# [Pt(PPh_3_)_4_]-Catalyzed Selective Diboration of Symmetrical and Unsymmetrical 1,3-Diynes

**DOI:** 10.1021/acs.joc.2c00844

**Published:** 2022-08-02

**Authors:** Jakub Szyling, Aleksandra Szymańska, Adrian Franczyk, Jędrzej Walkowiak

**Affiliations:** † Center for Advanced Technology, Adam Mickiewicz University in Poznan, Uniwersytetu Poznanskiego 10, 61-614 Poznan, Poland; ‡ Faculty of Chemistry, Adam Mickiewicz University in Poznan, Uniwersytetu Poznanskiego 8, 61-614 Poznan, Poland

## Abstract

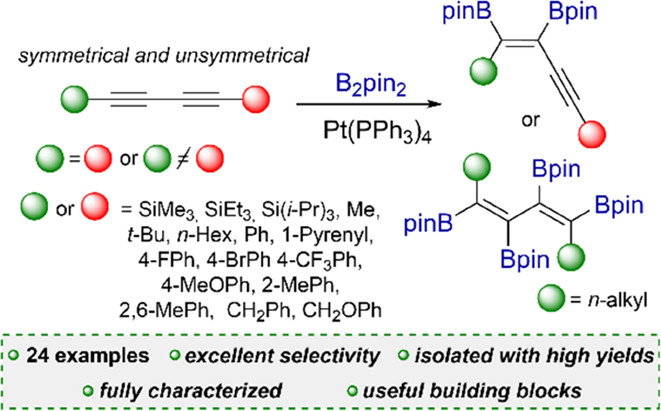

A straightforward, efficient, and selective method for the preparation of novel boryl-functionalized enynes or dienes via [Pt(PPh_3_)_4_]-catalyzed diboration of a broad spectrum of symmetrical and unsymmetrical 1,3-diynes was developed. The catalytic cycle of diboration was proposed on the basis of low-temperature ^31^P NMR studies. An alternative isolation method via product condensation on a cold finger was developed, which, in contrast to previous literature reports, eliminates the need for the additional transformation of rapidly decomposing enynyl pinacol boronates to more stable silica-based column chromatography derivatives during the separation step. To prove the efficiency of this simple catalytic protocol, bisboryl-functionalized enynes were synthesized in a gram scale and tested as useful building blocks in advanced organic transformations, such as hydrosilylation and Suzuki and sila-Sonogashira couplings. The presence of silyl, boryl, as well as other functions like halogen or alkoxy in their structures builds a new class of multifunctionalized enynes that might be modified in various chemical reactions.

## Introduction

Unsaturated organoboron compounds are powerful reagents in modern organic synthesis because of their low toxicity, high stability, and unique reactivity.^1−4^ The presence of unsaturated carbon–carbon bond(s) and the boryl moiety in their structure makes them unique building blocks in new C–C bond-forming catalytic processes,^5^ asymmetric transformations,^6^ and stereoselective demetallation protocols.^7,8^ The versatility of boron-containing molecules entails their application in chemical sensors and material science.^9−11^ In this class of organoboronates, π-conjugated C–C bond systems are extremely attractive, for example, enynyl or dienyl boronates, which were applied in the preparation of many biologically active or natural compounds (e.g., *chalcomoracin*, *muberrofuran C*, *apoptolidin A*, and (*+*)–*fostriecin*).^12−16^ Therefore, the development of efficient and selective methods for the synthesis of such molecules, which possess easy-to-modify boryl groups and conjugated chain, is of great importance.

Transition-metal (TM)-catalyzed hydroboration and diboration are the most important and commonly used reactions to incorporate boryl moiety(ies) into isolated or π-conjugated C–C bonds.^17−19^ These 100% atom-economy efficiency transformations are more compatible with the green chemistry principles than similar substitution reactions.

Both hydroboration and diboration are well established for alkenes/alkynes^20−25^ and also for more challenging allenes,^26−28^ 1,3-dienes,^29−32^ and 1,3-enynes functionalization.^33−35^ In contrast to this, the addition of boryl group(s) to 1,3-diynes is practically unexplored. The reason for this is possible over-reduction, formation of a mixture of various products and their isomers, and problems in selective activation of the one C≡C bond, which often requires the application of precisely designed metal complexes.

**Scheme 1:**
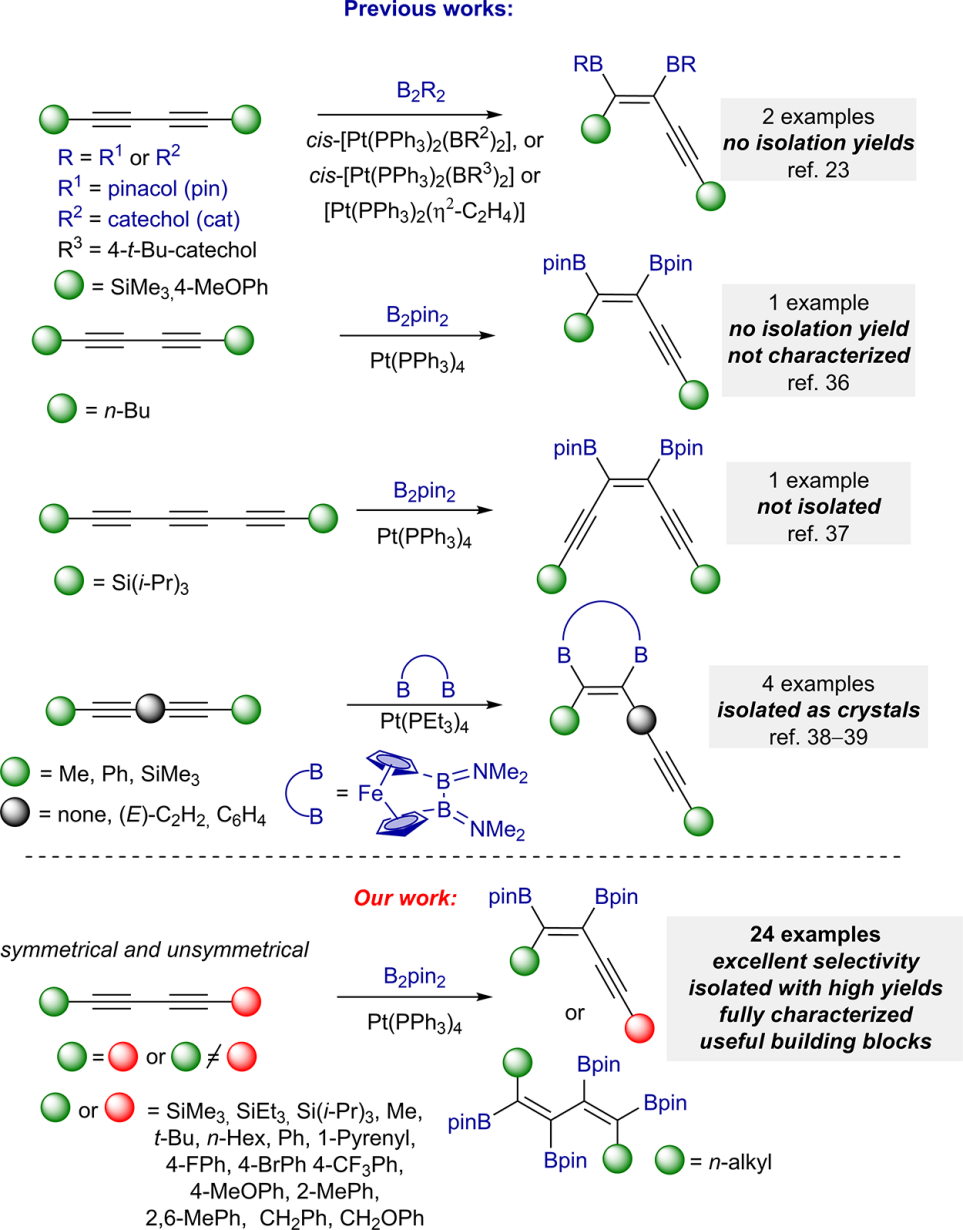
TM-Catalyzed Diboration of 1,3-Diynes and 1,3,5-Triyne

The first application of 1,3-diynes in TM-borylative functionalization was described by Marder, who used *cis*-bis(phosphine)platinum(II) bis(boryl) complexes in the diboration of a series of monoynes and two symmetrical diynes: 1,4-bis(trimethylsilyl)buta-1,3-diyne and 1,4-bis(4-methoxyphenyl)buta-1,3-diyne.^23^ The products of this reaction were characterized directly from the crude reaction mixture. No isolation procedure was described despite the fact that only symmetrical diynes were used. There are two examples of the application of the Pt(PPh_3_)_4_ complex in the diboration of dodeca-5,7-diyne^36^ and 1,6-bis(tri*iso*propylsilyl)hexa-1,3,5-triyne.^37^ In these cases, products were obtained as the reaction mixture or as intermediates. The products were not isolated and characterized, which make these protocols useless in the synthesis of bisboryl-functionalized enynes. The triyne diboration product was applied in further transformations without the isolation step because of its instability on silica-based separation methods. The diboration of diynes, where C≡C bonds were separated with alkyl, alkenyl, or aryl spacers, was carried out using [2]boraferrocenophanes. These reagents might be considered as alkynes because the bonds are separated and therefore their reactivity is different from conjugated 1,3-diynes. The products were isolated by crystallization in relatively low yields (Scheme 1).^38,39^ The monoboryl-functionalized enynes were first reported in 2015 by Li who used bis(pinacolato)diboron in a protoboration reaction catalyzed by a generated in situ system composed of copper(I) chloride, tri(*p*-tolyl)phosphine, and sodium *tert*-butoxide.^40^ Recently, Ge and co-workers reported the first regiodivergent hydroboration of symmetrical and unsymmetrical 1,3-diynes catalyzed by a generated in situ cobalt system.^41^ Slightly later, our group developed the ruthenium-hydride-catalyzed hydroboration of symmetrical aryl-substituted 1,3-diynes by pinacol borane, leading to 2-borylsubstituted-1,3-enynes with excellent yields and selectivity.^42^ The obtained products, in these studies, were transformed to more stable derivatives: 1,8-diaminonaphthalene boronates or potassium trifluorobates, respectively, which were simpler for isolation, but the additional transformations were necessary in both cases.

Platinum catalysts are generally highly effective in the addition of H–M or M–M (M = metalloids (B, Si)) to unsaturated C–C bonds, which has been shown in numerous studies.^17,21,43,44^ Encouraged by (i) almost neglected scientific reports regarding the diboration of 1,3-diynes, especially significantly more challenging unsymmetrically substituted diynes, (ii) challenges with the isolation of enynyl pinacol boronates without their transformations into more stable derivatives, (iii) our experience in the functionalization of diynes via hydroboration and hydrosilylation reactions,^42,45−47^ and (iv) the principle to find simply and commercially available catalysts that might be directly used in this transformation in each synthetic laboratory, we sought to investigate the diboration of symmetrical and unsymmetrical 1,3-diynes.

Thus, our work describes for the first time a simple and straightforward process leading to bisboryl-functionalized unsymmetrical-substituted enynes using commercially available and easy-to-handle [Pt(PPh_3_)_4_] catalyst, which is an important advantage of the described method. Various symmetrical and unsymmetrical diynes were used as reagents, building a library of advanced molecules that might be functionalized in various transformations. Moreover, a new separation method of sensitive bisborylsubstituted enynes that does not require any additional reactions was developed. In contrast to previous methods, the obtained products were fully characterized, synthesized in a gram scale, and used in many organic transformations.

## Results and Discussion

In the initial stage of our study, we tested several platinum catalysts: PtO_2_, Pt/C, Pt_2_(dvs)_3_ (Karstedt’s catalyst), H_2_PtCl_6_, and [Pt(PPh_3_)_4_] in order to investigate their activity with the addition of cheap and air-stable bis(pinacolato)diboron (**1**) to the C≡C bonds in symmetrically substituted 1,3-diynes with different substituents: 1,4-bis(trimethylsilyl)buta-1,3-diyne (**2a**), hexa-2,4-diyne (**2d**), and 1,4-diphenylbuta-1,3-diyne (**2g**). The heterogeneous catalysts (PtO_2_ and Pt/C) did not show activity under the applied reaction conditions (Table 1, entries 1–3). Slightly better results, although still unsatisfactory, were observed for molecular catalysts (Pt_2_(dvs)_3_ and H_2_PtCl_6_), which are highly active with the addition of Si–H to the C≡C bonds (Table 1, entries 4–6).^48^ The highest activity toward the B–B bond addition to **2a** was shown by relatively air-stable [Pt(PPh_3_)_4_], which led to a monoadduct (**3a**) with excellent yield (Table 1, entry 8). The addition of the phosphine ligand Xphos had a negative influence on the conversion and reaction selectivity (Table 1, entry 7). After selecting [Pt(PPh_3_)_4_] as a promising catalyst, the reaction conditions were optimized. Lowering the temperature from 110 to 80 °C resulted in the exclusive formation of **3a** (Table 1, entry 10). Moreover, the reaction time was reduced from 24 to 18 h without any changes in the process efficiency (Table 1, entry 11). The diboration of nonhindered and aliphatic 2,4-hexadiyne (**2d**) led to a mixture of both products (**3d**/**4d** = 70/30). However, significant dilution (10-fold) of the reaction mixture resulted in better selectivity toward monoaddition (**3d**/**4d** = 81/19). The exclusive formation of monoadducts **3d** was observed when a seven-fold excess of **2d** toward **1** was used (Table 1, entries 13–15). Moreover, for **2d**, it was also possible to obtain tetraboryl-substituted diene **4d** by using three-fold excess of **1** toward **2d** (Table 1, entries 16–17). The application of aromatic 1,4-diphenylbuta-1,3-diyne (**2g**) resulted in the complete conversion of substrates. However, traces of hydroboration products (**5**) were also formed unexpectedly (Table 1, entry 18). Replacing toluene with tetrahydrofuran led to selective diboration of one C≡C bond toward **3g**, while the second C≡C bond was not modified (Table 1, entry 19).

**Table 1:**
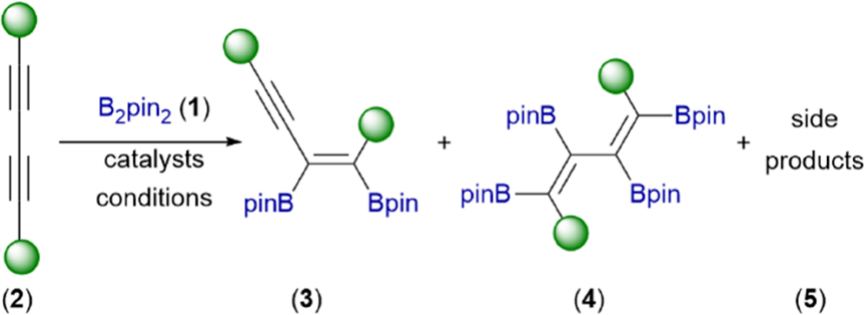

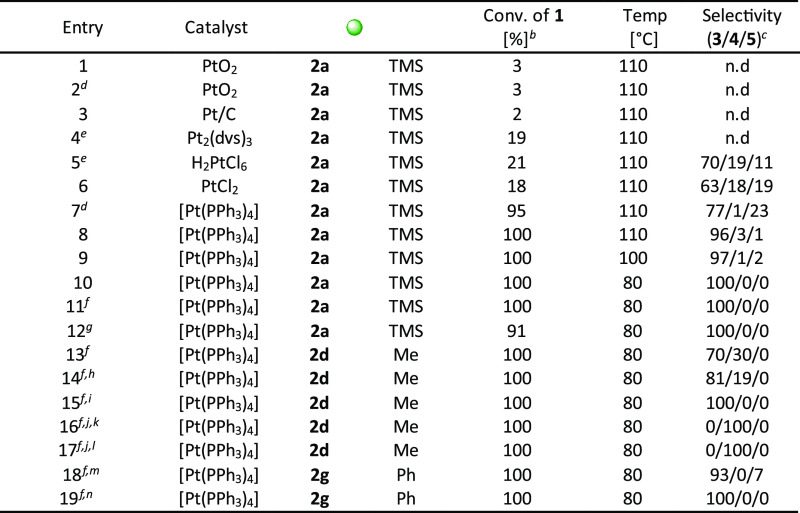
Catalysts and Conditions Screening for Selective Diboration of 1,4-Bis(trimethylsilyl)buta-1,3-diyne (**2a**), Hexa-2,4-diyne (**2d**) and 1,4-Diphenylbuta-1,3-diyne (**2g**)^a^

a Reaction conditions: [Pt]:[**1**]:[**2**] = 0.01:1:1, toluene (0.125 M), inert atmosphere, 24 h.

b Determined by GC–MS analysis.

c Determined by GC–MS and ^1^H NMR analyses.

d Xphos used as a ligand (1 mol %).

e Pt = 10^–3^ per Pt atom.

f 18 h.

g 12 h.

h Toluene 0.0125 M.

i [Pt]:[**1**]:[**2**] = 0.01:1:7.

j Calculated based on **2d** conv.

k [Pt]:[**1**]:[**2**] = 0.01:5:1.

l [Pt]:[**1**]:[**2**] = 0.01:3:1.

m Hydroboration products as side products.

n Reaction carried out in THF (0.125 M). Side products were identified as a mixture of hydroboration and undefined byproducts.

**Scheme 2:**
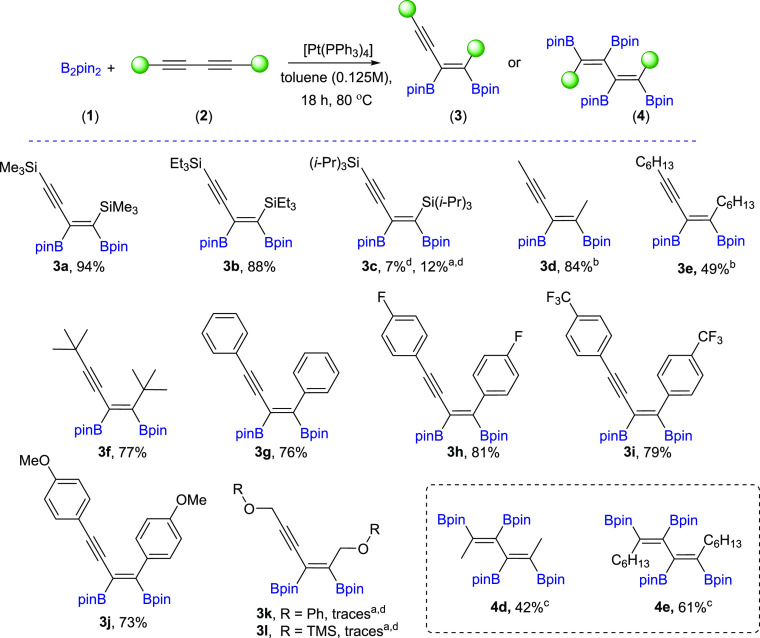
Scope of Products Obtained in the [Pt(PPh_3_)_4_]-Catalyzed Diboration or Tetraboration of Symmetrical 1,3-Diynes **2a–l**. Standard Reaction Conditions: [Pt(PPh_3_)_4_]:[**1**]:[**2a–l**] = 0.01:1:1, Toluene (**3a–f**, **3k–l**) or THF (**3g–j**) (0.125 M), 80 °C, 18 h, Ar. Isolated Yields Are Presented 110 °C, 48 h. [Pt]:[**1**]:[**2**] = 0.01:1:7. [Pt]:[**1**]:[**2**] = 0.01:3:1. Product yield based on GC–MS analysis.

With the selected [Pt(PPh_3_)_4_] catalyst and optimized reaction conditions in hand, we studied the scope of 1,3-diynes for platinum(0)-catalyzed diboration. Scheme 2 summarizes the results obtained for the functionalization of symmetrically 1,4-substituted-1,3-diynes (**2a–2l**). Generally, silyl-substituted 1,3-diynes with sterically unhindered groups such as −SiMe_3_ (**2a**) and −SiEt_3_ (**2b**) were smoothly converted to corresponding monoadducts, whereas the presence of bulky −Si(*i*-Pr)_3_ substituents resulted in a very low yield of **3c**, even at 110 °C and for 48 h. The diboration of linear hexadeca-7,9-diyne (**2e**) yielded the formation of mono- and bisadducts **3e**/**4e** = 75/25 under standard reaction conditions ([Pt(PPh_3_)_4_]:[**1**]:[**2e**] = 0.01:1:1, toluene, 80 °C, 18 h, Ar). However, similar to **2d**, depending on the ratio of the substrates, the di- or tetraboryl-substituted products (**3e** and **4e**, respectively) were selectively obtained. The 7-fold excess of **2e** toward **1** led to diboration product **3e**, whereas 3-fold excess of **1** toward **2e** led to tetraborylated diene **4e**. Alkyl-substituted 1,3-diyne with steric *t*-Bu moieties (**2f**) showed excellent selectivity toward monoaddition. Thus, the formation of boryl-functionalized enynes or dienes was only possible for symmetrical *n*-alkyl 1,3-diynes. For silyl, *t*-Bu, or aryl substituents, the diene formation was not observed under standard conditions. Aryl-substituted 1,3-diynes with different groups attached to the phenyl ring (−F, −CF_3_, −OMe) (**2h–j**) reacted in the presence of [Pt(PPh_3_)_4_] with B_2_pin_2_ (**1**) in THF, providing corresponding enynyl boronates (**3h–j**) with excellent selectivity and high isolation yields. Surprisingly, 1,3-diynes with phenoxy or silyloxy substituents (**2k–l**) were not active, even under harsher reaction conditions (110 °C, 48 h).

**Scheme 3:**
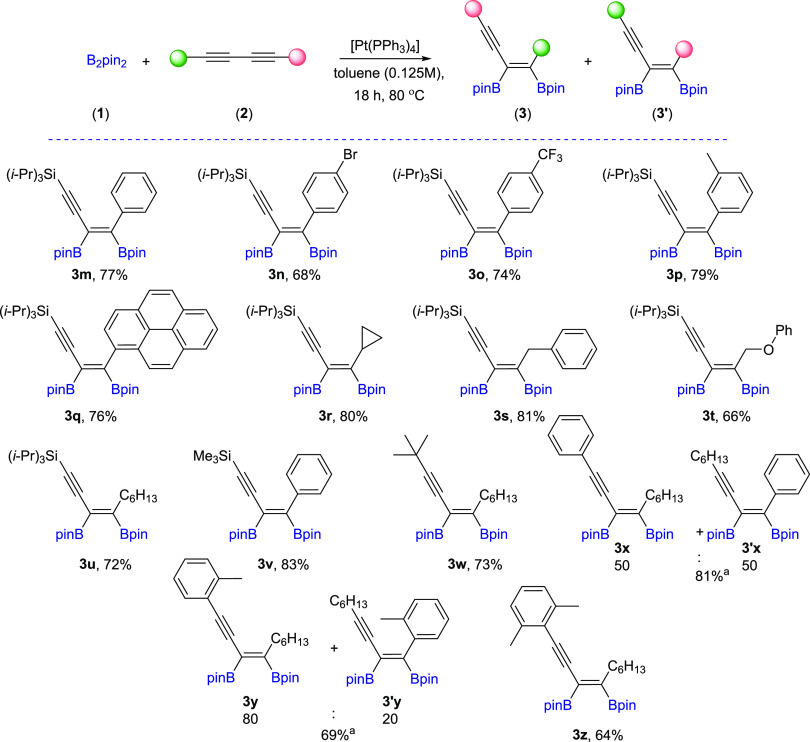
Scope of Products Obtained in the [Pt(PPh_3_)_4_]-Catalyzed Diboration of Unsymmetrical 1,3-Diynes **2m–z**. Reaction Conditions: [Pt(PPh_3_)_4_]:[**1**]:[**2m–z**] = 0.01:1:1, Toluene (0.125 M), 80 °C, 18 h, Ar. Isolated Yields Are Presented Isolated as the reaction mixture.

In the next step of our study, we synthesized various unsymmetrical substituted 1,3-diynes with silyl, aryl, or alkyl groups (**2m–z**), among which **2p–t** and **2y–z** are new compounds, and tested their reactivity in the diboration process in the presence of [Pt(PPh_3_)_4_] (Scheme 3). The 1,3-diyne with a bulky −Si(*i*-Pr_3_) group on the one side and a phenyl ring on the other (**2m**) gave a monoaddition product of **3m** with high isolation yield. Comparable results were obtained for 1,3-diynes with *para*-Br or *para*-CF_3_ or *meta*-Me substituted phenyl rings (**3n–p**). The presence of the polycyclic aromatic hydrocarbon structure (**2q**) or cyclopropyl (**2r**) moiety in the diyne is also acceptable for the proposed protocol, leading to interesting building blocks (**3q–r**). Incorporation of the methylene spacer between the sp-carbon and phenyl ring in the 1,3-diyne structure gave the corresponding enynyl boronates (**3s**) with a high isolation yield and without loss of selectivity. Satisfying results were also obtained for 1,3-diyne with phenoxy and −Si(*i*-Pr)_3_ groups (**2t**). The chemical shifts in ^29^Si NMR and correlative ^1^H–^13^C HSQC and HMBC NMR spectra confirmed that the addition of the B–B bond occurred at the triple bond situated further from the silyl group. The presence of −Si(*i*-Pr)_3_ promoted selective monoaddition of B_2_pin_2_ to **2u** with an *n*-hexyl group. Replacing −Si(*i*-Pr)_3_ with a smaller −SiMe_3_ group in **2v** had no influence on the reaction efficiency and smoothly led to **3v** with good isolation yield because the silyl moiety acts as the directing group.^40,49^ The developed protocol is also suitable for the diboration of alkyl–alkyl substituted unsymmetrical 1,3-diynes, with *t*-Bu and *n*-hex moieties (**3w**). The diboration of **2x** yielded an equivalent mixture of regioisomers (**3x**/**3′x** = 50:50). The incorporation of one small methyl group in the *meta* position into the phenyl ring resulted in an increase in selectivity toward the formation of **3y** (**3y**/**3′y** = 80:20). The application of the 2,6-dimethyl-substituted phenyl ring in diyne (**2z**) forced the selective formation of **3z**. All of the isolated unsymmetrical-substituted enynyl boronates (**3m–z**) are new compounds, which due to the presence of unsaturated bonds, as well as boryl, silyl, alkoxy, or halogen groups, constitute extremely attractive building blocks in organic synthesis (in addition, coupling and demetallation reactions), which will be discussed later in this article.

**Scheme 4:**
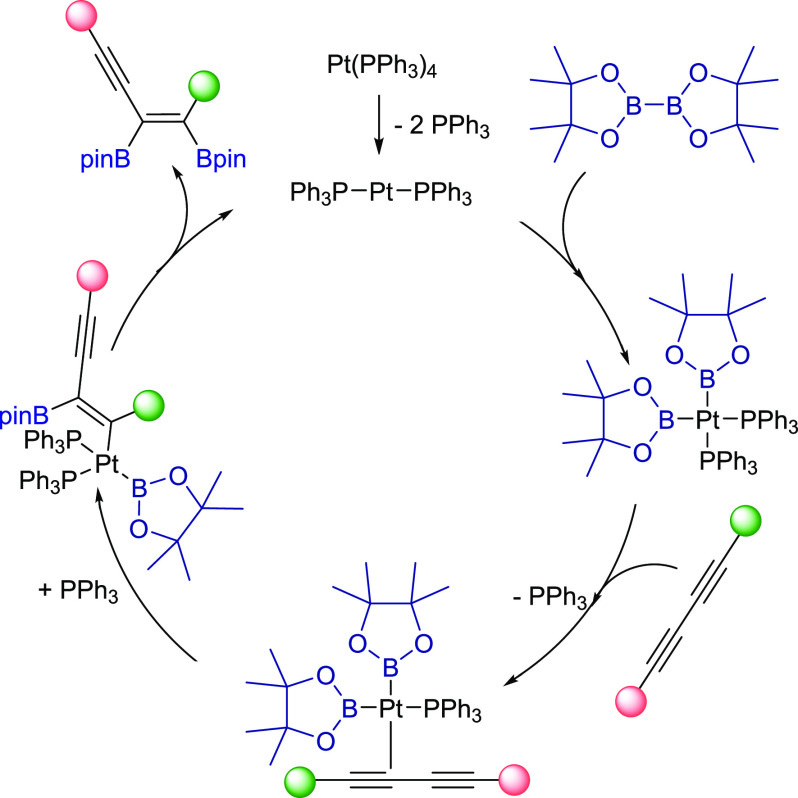
Proposed Catalytic Cycle for 1,3-Diynes Diboration with B_2_pin_2_ Catalyzed by [Pt(PPh_3_)_4_]

In the next stage of our study, we investigated the mechanism of [Pt(PPh_3_)_4_] diboration of 1,3-diynes (**2**) with B_2_pin_2_ (**1**). In the first step of the catalytic cycle, oxidative addition of the B–B bond to the metal center proceeded similarly to the diboration of alkynes.^22^ The low-temperature ^31^P NMR revealed the formation of a new signal at 28.6 ppm and its satellites at 33.3 and 23.9 ppm with coupling constant *J*_Pt–P_ = 1517 Hz, which corresponds to the *cis*-coordinated phosphines to the Pt(II) atom. Subsequently, similar to alkyne diboration, coordination of the C≡C bond and insertion into one of the Pt–B bonds occurred. Reductive elimination released bisboryl-functionalized enyne and regenerated the initial catalyst (Scheme 4). To prove the *cis*-addition of B_2_pin_2_ (**1**) to diyne, the selective 1D NOESY NMR spectrum was carried out for product **3h** (see the SI).

**Scheme 5:**
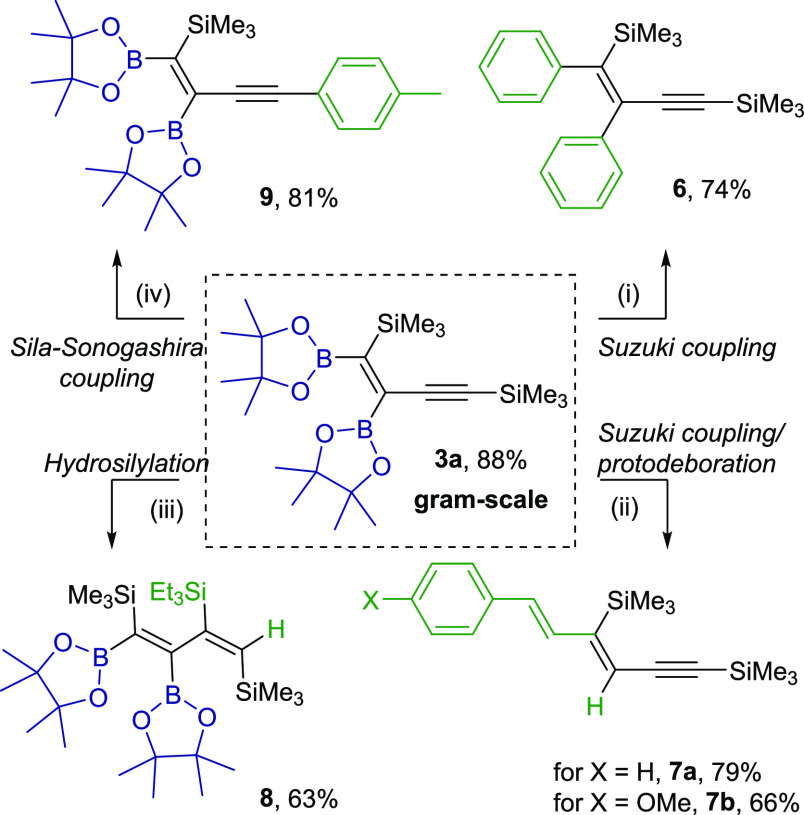
Application of **3a** as a Building Block in Catalytic Transformations Reaction conditions: for (i) and (ii): Pd(PPh_3_)_4_ (5 mol %), THF, 3 M Cs_2_CO_3_, 60 °C, 18 h, argon, for (i) iodobenzene 2.4 equiv, for (ii) (*E*)-styryl iodides 1.2 equiv (iii):[Et_3_SiH]:[**3a**]:[Pt_2_(dvs)_3_] = [1.2]:[1]:[10^–3^ per Pt atom], toluene (1 M), 100 °C, 24 h. (iv): [4-iodotoluene]:[**3a**]:[CuI]:[Pd(PPh_3_)_4_] = [1.1]:[1]:[0.5]:[0.05], DMF, 80 °C, 18 h.

Generally, unsaturated organoboron compounds are air-stable and easy to handle molecules.^50^ However, enynyl pinacol boronates, in contrast to nonconjugated alkenyl boronates, are unstable or stable to a very limited extent with silica-based purification in air. Because of this fact, in most previous reports, these compounds were converted to more stable versions on silica-based column chromatography 1,8-diaminonaphthalene boronates or trifluoroborate salts.^41,42^ Herein, we present a simple and convenient method for the isolation of the enynyl pinacol boronates based on the condensation of the product on a cold finger under vacuum (see the SI). This protocol could be adopted for most products because the postreaction workup only requires separation of the single product from the catalyst and solvent because of the high regio- and stereoselectivity of the developed process. The products were isolated in moderately high or high yields as solids or oils. Among 26 synthesized boryl-functionalized enynes or dienes, 24 are new compounds. In contrast to the previous report utilizing [Pt(PPh_3_)_2_(C_2_H_4_)] or *cis*-bis(phosphine)platinum(II) bis(boryl) complexes in diynes diboration,^23^ in the presented protocol, comprehensive studies with a wide range of symmetrical and unsymmetrical 1,3-diynes with the application of commercially available and easy-to-handle catalysts were described. Moreover, this is the first and most efficient method for preparing pinacolborane derivatives without the need to convert Bpin to more stable derivatives and the first studies which fully characterize obtained products. Such a collection of bisborylfunctionalized enynes, strengthened by the simple isolation method and straightforward procedure using a commercially available catalyst, builds a new class of multifuctionalized compounds that might be used in a wide range of organic transformations. To prove the utility of the [Pt(PPh_3_)_4_]-catalyzed diboration of 1,3-diynes, we performed a gram-scale reaction of **2a** with **1**. After 18 h, a complete conversion of the substrate was observed with excellent selectivity. Product **3a** was easily isolated in a high yield (1.17 g, 88%) on a cold finger condenser as a white solid. The presence of the C=C and C≡C bonds, boryl, and silyl moieties makes product **3a** an extremely attractive building block in the organic synthesis. To verify this, the transformations of each reactive group were demonstrated. Depending on the reaction conditions, the boryl moieties could be selectively utilized in Suzuki coupling or Suzuki coupling/protodeboration sequence, giving products **6** and **7** with high isolated yields. Similarly, the different reactivity of double and triple C–C bonds, as well as Csp^2^–SiMe_3_ and Csp–SiMe_3_ bonds, gave the unreported trisilyl-substituted diene **8** in hydrosilylation reaction or product **9** in sila-Sonogashira coupling with good yields (63 and 81%, respectively) (Scheme 5).

## Conclusions

In conclusion, we have comprehensively described for the first time an efficient and selective method for the synthesis and isolation of bisboryl-functionalized enynes through [Pt(PPh_3_)_4_]-catalyzed diboration of 1,3-diynes. The protocol is suitable for either symmetrical or unsymmetrical substituted 1,3-diynes with various functional groups such as aryl, silyl, halogen, and trifluoromethane. For *n*-alkyl-substituted 1,3-diynes, it is also possible to obtain selectively tetraboryl-functionalized dienes using an excess of bis(pinacolato)diboron. Moreover, in contrast to previous reports, enynyl boronates were isolated with high yields and fully characterized without the need for their transformation to more stable on silica-based column chromatography derivatives. No need for the application of specially designed TM complexes (as in the previously described examples), the equimolar ratio of reagents, high process stero- and regioselectivity, and simple separation method, constitute advantages of this new protocol that might be applied in every synthetic laboratory focusing on the synthesis of *fine chemicals*. The products, of which 24 have been reported for the first time because of the presence of unsaturated C–C triple and double bonds, boryl groups and various other functions (e.g., silyl, alkoxy, halogen) create an important class of building blocks, which might be applied in the synthesis of advanced products. Their reactivity might be distinguished by differences in the reactivity of these functional groups, selection of an appropriate catalyst, and different reactivity of the C=C and C≡C bonds.

## Experimental Section

### General Information

^1^H, ^11^B, ^13^C, and ^29^Si NMR spectra were recorded at 25 °C on Bruker UltraShield 300, 400, or 600 MHz with a number of scans (NS) for ^1^H NMR = 16, ^13^C NMR = 512 or 1024 (unless otherwise stated). Chemical shifts were reported in ppm with the reference to the residue portion solvent peak (^1^H, ^13^C NMR) or BF_3_-Et_2_O and TMS for ^11^B and ^29^Si, respectively. Chloroform-d_1_ or toluene-d_8_ were used as solvents and for internal deuterium lock. The multiplicities were reported as follows: singlet (s), doublet (d), doublet of doublets (dd), multiplet (m), triplet (t), pentet (p), and doublet of doublets of triplets (ddt). The mass spectra of the products were obtained by gas chromatography–mass spectrometry (GC–MS) analysis on a Bruker Scion 436-GC with a 30 m Varian DB-5 0.25 mm capillary column and a Scion SQ-MS mass spectrometry detector. Two temperature programs were used: (a) 60 °C (3 min), 10 °C/min, 250 °C (30 min), (b) 100 °C (3 min), 10 °C/min, 280 °C (44.5 min). FT-IR spectra were measured on a Nicolet iS50 FT-IR spectrometer (Thermo Scientific) equipped with a built-in ATR accessory with ATR diamond unit. In all experiments, 16 scans at a resolution of 2 cm^–1^ were performed. Elemental analyses were performed using the Vario EL III instrument.

### General Procedures

All manipulations were performed using standard Schlenk’s techniques, unless otherwise stated. For the procedures of the synthesis of starting materials and functionalization of product **3a**, see the SI.

#### Synthesis of Symmetrical 1,3-Diynes (**2a–l**)

Symmetrical 1,3-diynes were prepared according to the following procedure:

The CuCl (0.1 mmol) was placed in a round-bottom bulb equipped with a condenser and magnetic stirring bar. Subsequently, toluene (10 mL), piperidine (0.15 mmol), and alkyne (5 mmol) were placed in the reaction vessel. The reaction was performed at 80 °C using oil bath as a heat source for 18 h in an air atmosphere. Afterward, the reaction mixture was cooled, and all volatiles were removed under vacuum. The crude residue was dissolved in hexanes (with a small amount of dichloromethane if necessary) and purified (see the SI). The synthesis of **2e** was performed in a Rotaflo-type Schlenk vessel because of the low boiling point of the initial alkyne.

#### Preparation of 2,2,11,11-Tetramethyl-3,10-dioxa-2,11-disiladodeca-5,7-diyne (**2l**)

Hexa-2,4-diyne-1,6-diol (5 mmol) was placed in a three-neck round-bottom flask equipped with a stirring bar and a reflux condenser under an argon atmosphere. Subsequently, dry THF (10 mL) and hexamethyldisilazane (6 mmol) were added. The reaction was allowed to continue at 70 °C using oil bath as a heat source until no further evidence of ammonia was observed. The excess of HMDS was easily removed under vacuum. The product was characterized by GC–MS, FT-IR and ^1^H, ^13^C and ^29^Si NMR analyses.

#### Synthesis of Unsymmetrical 1,3-Diynes (**2m–z**)

The unsymmetrical 1,3-diynes were prepared according to the literature^51^ with some modifications:

CuCl was dissolved in a 2:3 mixture by volume of *n*-BuNH_2_:H_2_O (5 mL/mmol alkyne), and the solution was cooled to 0 °C in an ice bath. Hydroxylamine hydrochloride was slowly added until trace amounts of copper(II) were reduced, and the color of the solution changed from blue to colorless. Alkyne bromide and alkyne were dissolved in dichloromethane (5 mL/mmol alkyne), and this solution was added to the reaction flask at once. The biphasic mixture was vigorously stirred overnight under an argon atmosphere. Subsequently, the organic layer was removed and washed with portions of saturated aq. NH_4_Cl until these portions no longer took on a blue color. The organic layer was dried (MgSO_4_) and concentrated by rotary evaporation. The crude residue was dissolved in hexanes and purified (see the SI).

#### Diboration of 1,3-Diynes (**2a–z**)

[Pt(PPh_3_)_4_] (0.0025 mmol), bis(pinacolato)diboron (0.25 mmol), diyne (0.25 mmol), and toluene (THF for **2g–j**; 2 mL, 0.125 M) were added into a Schlenk vessel under an argon atmosphere and stirred for 18 h at 80 °C using oil bath as a heat source. Afterward, the crude reaction mixture was analyzed by GC–MS and ^1^H NMR analyses and purified (see the SI). Products **3d–e** were prepared according to the abovementioned procedure with 7-fold excess of diyne toward bis(pinacolato)diboron, while for **4d–e**, the 3-fold excess of bis(pinacolato)diboron towards diyne was used.

#### (*Z*)-(1,2-Bis(4,4,5,5-tetramethyl-1,3,2-dioxaborolan-2-yl)but-1-en-3-yne-1,4-diyl)bis(trimethylsilane) (**3a**)


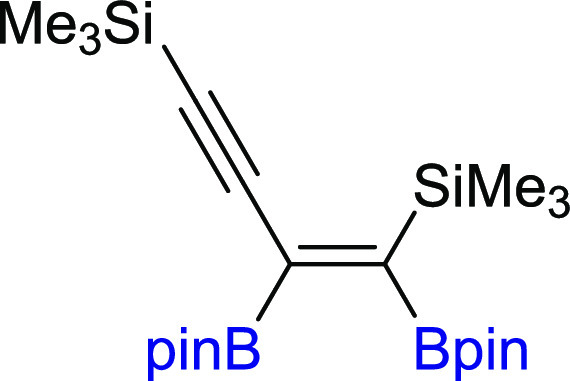
^1^H NMR (300 MHz, CDCl_3_, δ, ppm): 1.30 (s, 12H, C(CH_3_)_2_), 1.27 (s, 12H, C(CH_3_)_2_), 0.23 (s, 9H, Si(CH_3_)_3_), 0.16 (s, 9H, Si(CH_3_)_3_). ^13^C{^1^H} NMR (75 MHz, CDCl_3_, δ, ppm): 108.07 (C≡C), 103.0 (C≡C), 84.4 (C(CH_3_)_2_), 83.8 (C(CH_3_)_2_), 25.6 (C(CH_3_)_2_), 24.8 (C(CH_3_)_2_), −0.1 (Si(CH_3_)_3_), −0.5 (Si(CH_3_)_3_). Cα to boron atoms were not observed. ^29^Si NMR (79 MHz, CDCl_3_ δ, ppm): −6.12 (SiC=C), −18.66 (SiC≡C). ^11^B NMR (128 MHz, CDCl_3_, δ, ppm): 31.26, 28.47. MS (EI, *m*/*z*): 448(M^+^, 1), 443(2), 390(2), 333(2), 307(3), 249(2), 207(2), 175(3), 149(2), 84(100), 73(18), 55(10). FT-IR (cm^–1^): 2978, 1641, 1526, 1361, 1320, 1138, 1100, 980, 841, 755. White solid. Isolated yield: 94% (104 mg), for gram scale: isolated yield: 88% (1.18 g). Analytical data are in agreement with the literature.^21^

#### (*Z*)-(1,2-Bis(4,4,5,5-tetramethyl-1,3,2-dioxaborolan-2-yl)but-1-en-3-yne-1,4-diyl)bis(triethylsilane) (**3b**)


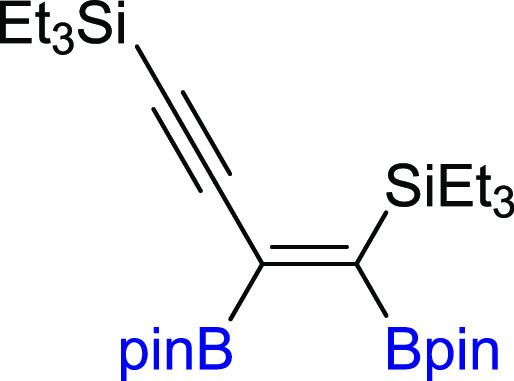
^1^H NMR (300 MHz, CDCl_3_, δ, ppm): 1.29 (s, 12H, C(CH_3_)_2_), 1.28 (s, 12H, C(CH_3_)_2_), 1.04–0.91 (m, 18H, Si(CH_2_CH_3_)_3_), 0.89–0.78 (m, 6H, Si(CH_2_CH_3_)_3_), 0.60 (q, *J*_H–H_ = 7.8 Hz, 6H, Si(CH_2_CH_3_)_3_). ^13^C{^1^H} NMR (75 MHz, CDCl_3_, δ, ppm): 109.3 (C≡C), 101.2 (C≡C), 84.2 (C(CH_3_)_2_), 83.7 (C(CH_3_)_2_), 25.5 (C(CH_3_)_2_), 24.9 (C(CH_3_)_2_), 7.9, 7.5, 4.6, 3.9. Cα to boron atoms were not observed. ^29^Si NMR (79 MHz, CDCl_3_ δ, ppm): 1.89 (SiC=C), −8.25 (SiC≡C). ^11^B NMR (128 MHz, CDCl_3_, δ, ppm): 31.27, 28.58. MS (EI, *m*/*z*): 503(M^+^-29, 6), 475(5), 375(2), 347(2), 319(3), 293(2), 265(2), 115(10), 84(100), 69(13), 55(21). FT-IR (cm^–1^): 2978, 2890, 1639, 1520, 1358, 1321, 1140, 1101, 981, 838, 756. Anal. calcd for C_28_H_54_B_2_O_4_Si_2_: C, 63.15; H, 10.22. Found: C, 62.98; H, 10.03. Colorless oil. Isolated yield: 88% (116 mg).

#### (*Z*)-(1,2-Bis(4,4,5,5-tetramethyl-1,3,2-dioxaborolan-2-yl)but-1-en-3-yne-1,4-diyl)bis(triisopropylsilane) (**3c**)


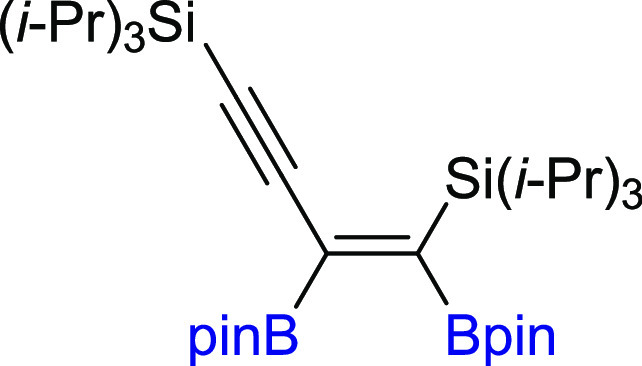
MS (EI, *m*/*z*): 573(M^+^-43, 18), 532(51), 489(14), 347(11), 282(14), 263(10), 211(9), 132(12), 115(21), 84(100), 73(18). Not isolated.

#### (*Z*)-2,2′-(Hex-2-en-4-yne-2,3-diyl)bis(4,4,5,5-tetramethyl-1,3,2-dioxaborolane) (**3d**)


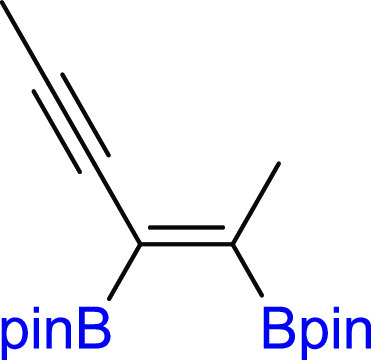
^1^H NMR (300 MHz, CDCl_3_, δ, ppm): 2.03 (s, 3H, CH_3_), 1.99 (s, 3H, CH_3_), 1.30 (s, 12H, C(CH_3_)_2_), 1.27 (s, 12H, C(CH_3_)_2_).^13^C{^1^H} NMR (75 MHz, CDCl_3_, δ, ppm): 95.71, 84.0 (C(CH_3_)_2_), 83.9 (C(CH_3_)_2_), 24.9 (C(CH_3_)_2_), 24.9 (C(CH_3_)_2_), 20.4, 5.2. Cα to boron atoms were not observed. ^11^B NMR (128 MHz, CDCl_3_, δ, ppm): 30.17. MS (EI, *m*/*z*): 332(M^+^, 1), 275(7), 254(2), 191(18), 175(5), 147(13), 84(100), 69(19), 55(13). FT-IR (cm^–1^): 2978, 2933, 1581, 1438, 1362, 1338, 1311, 1143, 1117, 963, 852, 723, 542. Anal. calcd for C_18_H_30_B_2_O_4_: C, 65.11; H, 9.11. Found: C, 65.17; H, 9.14. Yellow solid. Isolated yield: 84% (70 mg).

#### (*Z*)-2,2′-(Hexadec-7-en-9-yne-7,8-diyl)bis(4,4,5,5-tetramethyl-1,3,2-dioxaborolane) (**3e**)


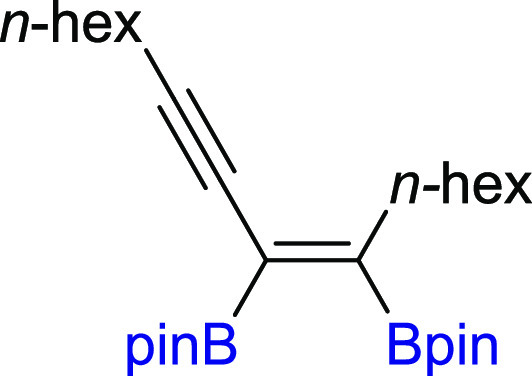
^1^H NMR (400 MHz, CDCl_3_, δ, ppm): 2.57–2.20 (m, 4H), 1.45–1.29 (m, 12H), 1.29 (s, 12H, C(CH_3_)_2_), 1.27 (s, 12H, C(CH_3_)_2_), 1.26–1.11 (m, 4H), 0.90–0.85 (m, 6H). ^13^C{^1^H} NMR (101 MHz, CDCl_3_, δ, ppm): 99.7, 83.9 (C(CH_3_)_2_), 83.7 (C(CH_3_)_2_), 35.0, 31.9, 31.7, 31.6, 29.7, 29.1, 28.7, 25.0 (C(CH_3_)_2_), 24.9 (C(CH_3_)_2_), 22.8, 20.2, 14.2. ^11^B NMR (128 MHz, CDCl_3_, δ, ppm): 30.90. MS (EI, *m*/*z*): 472(M^+^, 1), 415(2), 389(5), 389(5), 289(6), 254(2), 219(3), 175(5), 101(8), 84(100), 55(20). Anal. calcd for C_28_H_50_B_2_O_4_: C, 71.20; H, 10.67. Found: C, 71.07; H, 10.60. Pale yellow oil. Isolated yield: 49% (58 mg).

#### (*Z*)-2,2′-(2,2,7,7-Tetramethyloct-3-en-5-yne-3,4-diyl)bis(4,4,5,5-tetramethyl-1,3,2-dioxaborolane) (**3f**)


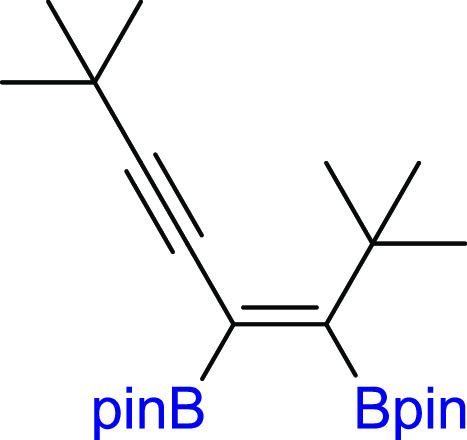
^1^H NMR (300 MHz, CDCl_3_, δ, ppm): 1.31 (s, 12H, C(CH_3_)_2_), 1.30 (s, 9H, (CH_3_)_3_) 1.27 (s, 12H, C(CH_3_)_2_), 1.22 (s, 9H, (CH_3_)_3_). ^13^C{^1^H} NMR (75 MHz, CDCl_3_, δ, ppm): 110.0, 83.9 (C(CH_3_)_2_), 83.8 (C(CH_3_)_2_),30.9, 29.7, 25.6 (C(CH_3_)_2_), 24.8 (C(CH_3_)_2_). Cα to boron atoms were not observed. ^11^B NMR (128 MHz, CDCl_3_, δ, ppm): 29.78. MS (EI, *m*/*z*): 332(M^+^, 1), 275(7), 254(2), 191(18), 175(5), 147(13), 84(100), 69(19), 55(13). FT-IR (cm^–1^): 2964, 2872, 1703, 1574, 1479, 1463, 1366, 1259, 1090, 1016, 800. Anal. calcd for C_24_H_42_B_2_O_4_: C, 69.26; H, 10.17. Found: C, 69.33; H, 10.11. Pale yellow solid. Isolated yield: 77% (80 mg).

#### (*Z*)-2,2′-(1,4-Diphenylbut-1-en-3-yne-1,2-diyl)bis(4,4,5,5-tetramethyl-1,3,2-dioxaborolane) (**3g**)


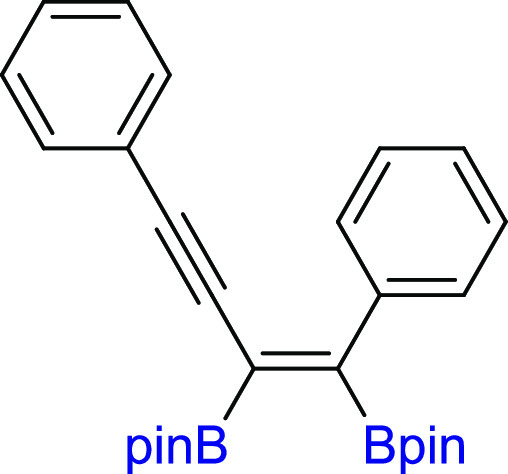
^1^H NMR (300 MHz, CDCl_3_, δ, ppm): 7.52–7.41 (m, 2H, Ph), 7.29–7.09 (m, 8H, Ph), 1.29 (s, 12H, C(CH_3_)_2_), 1.22 (s, 12H, C(CH_3_)_2_). ^13^C{^1^H} NMR (75 MHz, CDCl_3_, δ, ppm): 141.4, 131.8, 128.8, 128.1, 128.0, 127.6, 127.3, 124.3, 97.3, 90.6, 84.5 (C(CH_3_)_2_), 84.4 (C(CH_3_)_2_), 25.0 (C(CH_3_)_2_), 24.9 (C(CH_3_)_2_). Cα to boron atoms were not observed. ^11^B NMR (128 MHz, CDCl_3_, δ, ppm): 30.04. MS (EI, *m*/*z*): 456(M^+^, 3) 399(5), 373(4), 315(6), 273(8), 257(4), 229(10), 202(100), 129(10), 84(68), 69(21), 55(18). FT-IR (cm^–1^): 2977, 1442, 1358, 1318, 1262, 1205, 1138, 1068, 977, 847, 756, 690. Anal. calcd for C_28_H_34_B_2_O_4_: C, 73.72; H, 7.51. Found: C, 73.68; H, 7.48. Pale yellow solid. Isolated yield: 76% (86 mg).

#### (*Z*)-2,2′-(1,4-Bis(4-fluorophenyl)but-1-en-3-yne-1,2-diyl)bis(4,4,5,5-tetramethyl-1,3,2-dioxaborolane) (**3h**)


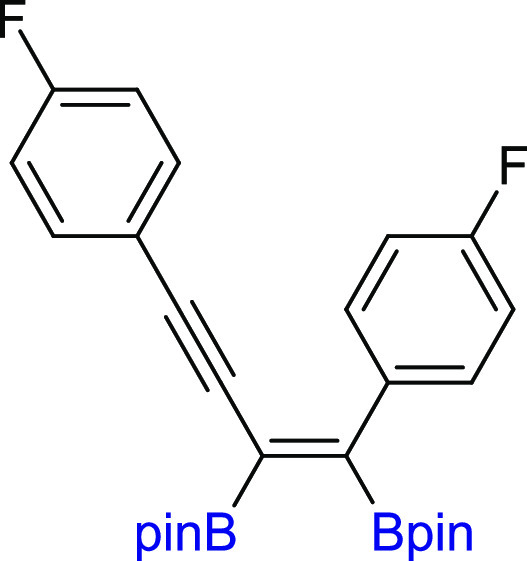
^1^H NMR (300 MHz, CDCl_3_, δ, ppm): 7.58–7.46 (m, 2H, Ph), 7.30–7.17 (m, 2H, Ph), 7.09–6.87 (m, 4H, Ph), 1.38 (s, 12H, C(CH_3_)_2_), 1.31 (s, 12H, C(CH_3_)_2_). ^13^C{^1^H} NMR (75 MHz, CDCl_3_, δ, ppm): 162.5 (d, *J*^1^_C–F_ = 244.7 Hz), 162.2 (d, *J*^1^_C–F_ = 245.9 Hz), 137.3 (d, *J*^4^_C–F_ = 3.5 Hz), 133.6 (d, *J*^3^_C–F_ = 8.3 Hz), 130.6 (d, *J*^3^_C–F_ = 7.9 Hz), 120.2 (d, *J*^4^_C–F_ = 3.6 Hz), 115.6 (d, *J*^2^_C–F_ = 22.1 Hz), 114.6 (d, *J*^2^_C–F_ = 21.3 Hz), 96.5 (C≡C), 90.0 (C≡C), 84.6 (C(CH_3_)_2_), 84.5 (C(CH_3_)_2_), 25.0 (C(CH_3_)_2_), 24.9 (C(CH_3_)_2_). Cα to boron atoms were not observed. ^11^B NMR (128 MHz, CDCl_3_, δ, ppm): 29.88. MS (EI, *m*/*z*): 492(M^+^, 3), 435(5), 335, 309(3), 265(3), 239(37), 201(4), 146(4), 85(100), 69(25), 55(14). FT-IR (cm^–1^): 2982, 1615, 1507, 1362, 1316, 1262, 1211, 1138, 1125, 1057, 956, 842. Anal. calcd for C_28_H_32_B_2_F_2_O_4_: C, 68.33; H, 6.55. Found: C, 68.38; H, 6.59. Yellowish solid. Isolated yield: 81% (99 mg).

#### (*Z*)-2,2′-(1,4-Bis(4-(trifluoromethyl)phenyl)but-1-en-3-yne-1,2-diyl)bis(4,4,5,5-tetramethyl-1,3,2-dioxaborolane) (**3i**)


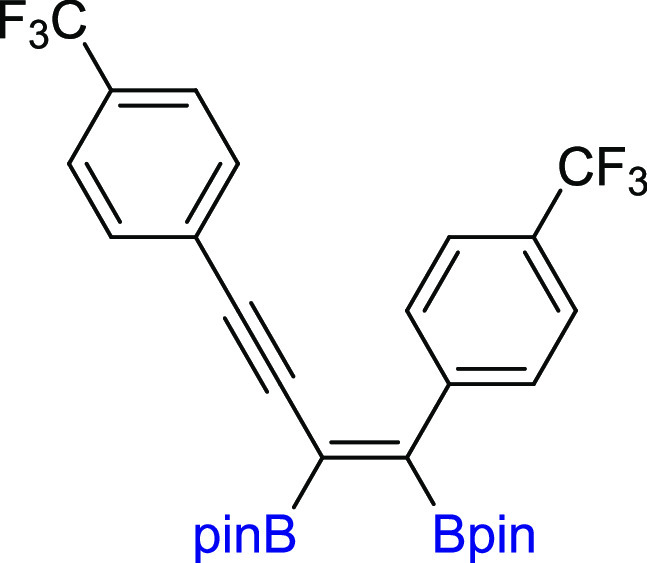
^1^H NMR (300 MHz, CDCl_3_, δ, ppm): 7.60 (s, 4H, Ph), 7.49 (d, *J*_H–H_ = 8.1 Hz, 2H, Ph), 7.28 (d, *J*_H–H_ = 8.2 Hz, 2H, Ph), 1.39 (s, 12H, C(CH_3_)_2_), 1.31 (s, 12H, C(CH_3_)_2_).^13^C{^1^H} NMR (75 MHz, CDCl_3_, δ, ppm): 131.9, 129.0, 125.2 (q, *J*^3^_C–F_ = 3.8 Hz), 124.8 (q, *J*^3^_C–F_ = 3.8 Hz), 84.9 (C(CH_3_)_2_), 84.8 (C(CH_3_)_2_), 25.0 (C(CH_3_)_2_), 24.9 (C(CH_3_)_2_). Cα to boron atoms were not observed. ^11^B NMR (128 MHz, CDCl_3_, δ, ppm): 29.94. MS (EI, *m*/*z*): 592(M+, 1) 573(1), 535(7), 451(7), 409(3), 347(2), 251(1), 197(1), 101(7), 84(100), 69(24), 57(41). FT-IR (cm^–1^): 2979, 1612, 1551, 1361, 1318, 1209, 1166, 1109, 1055, 1016, 841. Anal. calcd for C_30_H_32_B_2_F_6_O_4_: C, 60.85; H, 5.45. Found: C, 60.71; H, 5.47. White solid. Isolated yield: 79% (116 mg).

#### (*Z*)-2,2′-(1,4-Bis(4-methoxyphenyl)but-1-en-3-yne-1,2-diyl)bis(4,4,5,5-tetramethyl-1,3,2-dioxaborolane) (**3j**)


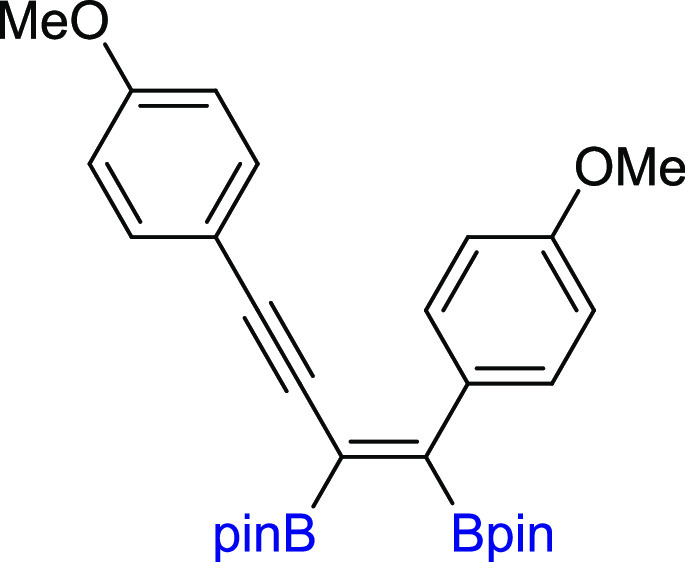
^1^H NMR (300 MHz, CDCl_3_, δ, ppm): 7.20–6.94 (m, 8H, Ph) 2.28 (s, 3H, OCH_3_), 1.97 (s, 3H, OCH_3_), 1.40 (s, 12H, C(CH_3_)_2_), 1.27 (s, 12H, C(CH_3_)_2_). ^13^C{^1^H} NMR (75 MHz, CDCl_3_, δ, ppm): 141.1, 128.7, 127.5, 127.3, 105.5, 103.7, 84.4 (C(CH_3_)_2_), 24.9 (C(CH_3_)_2_), 24.8 (C(CH_3_)_2_). Cα to boron atoms were not observed. ^11^B NMR (128 MHz, CDCl_3_, δ, ppm): 29.94. MS (EI, *m*/*z*): 484(M^+^-32, 2) 428(2), 401(10), 327(5), 301(4), 285(6), 257(6), 241(11), 230(100), 215(11), 115(17), 84(51), 69(24), 55(24). FT-IR (cm^–1^): 3008, 2978, 1601, 1560, 1358, 1319, 1203, 1161, 1021, 840, 821. Yellowish solid. Isolated yield: 73% (94 mg). Analytical data are in agreement with the literature.^21^

#### (*Z*)-(4-Phenyl-3,4-bis(4,4,5,5-tetramethyl-1,3,2-dioxaborolan-2-yl)but-3-en-1-yn-1-yl)trisopropylsilane (**3m**)


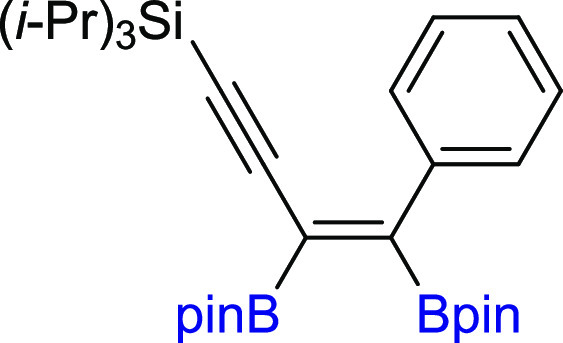
^1^H NMR (300 MHz, CDCl_3_, δ, ppm): 7.52–7.38 (m, 2H, Ph), 7.30–7.05 (m, 3H, Ph), 1.33 (s, 12H, C(CH_3_)_2_), 1.26 (s, 12H, C(CH_3_)_2_), 0.94 (s, 21H, Si(CH(CH_3_)_2_)_3_). ^13^C{^1^H} NMR (75 MHz, CDCl_3_, δ, ppm): 141.2, 128.8, 127.5, 127.0, 107.0 (C≡C), 101.1 (C≡C), 84.4 (C(CH_3_)_2_), 84.3 (C(CH_3_)_2_), 24.9 (C(CH_3_)_2_), 18.6, 11.4. Cα to boron atoms were not observed. ^29^Si NMR (79 MHz, CDCl_3_ δ, ppm): −2.54. ^11^B NMR (128 MHz, CDCl_3_, δ, ppm): 29.94. MS (EI, *m*/*z*): 536(M^+^, 1), 493(5), 393(54), 311(25), 255(7), 213(11), 115(10), 83(100), 55(46). FT-IR (cm^–1^): 2942, 2891, 2865, 1686, 1598, 1462, 1359, 1318, 1141, 1071, 996, 881, 674. Anal. calcd for C_31_H_50_B_2_O_4_Si: C, 69.41; H, 9.40. Found: C, 69.28; H, 9.37. Pale yellow oil. Isolated yield: 77% (103 mg).

#### (*Z*)-(4-(4-Bromophenyl)-3,4-bis(4,4,5,5-tetramethyl-1,3,2-dioxaborolan-2-yl)but-3-en-1-yn-1-yl)trisopropylsilane (**3n**)


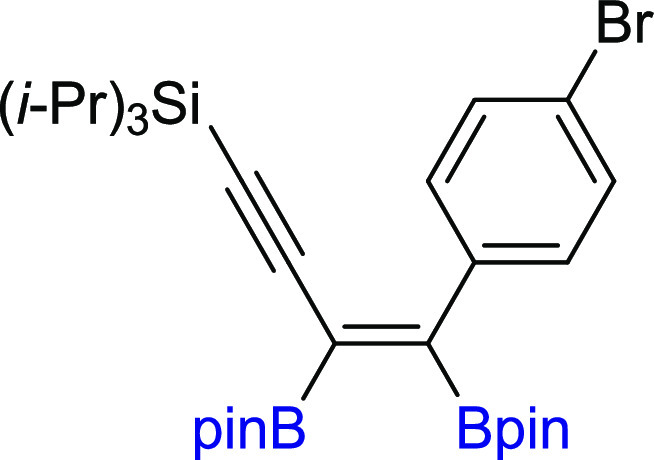
^1^H NMR (300 MHz, CDCl_3_, δ, ppm): 7.43–7.30 (m, 4H, Ph), 1.37 (s, 12H, C(CH_3_)_2_), 1.35 (s, 12H, C(CH_3_)_2_), 0.96 (s, 21H, Si(CH(CH_3_)_2_)_3_). ^13^C{^1^H} NMR (75 MHz, CDCl_3_, δ, ppm): 140.1, 130.7, 130.6, 121.1, 106.6 (C≡C), 102.6 (C≡C), 84.6 (C(CH_3_)_2_), 84.4 (C(CH_3_)_2_), 24.9 (C(CH_3_)_2_), 18.6, 11.4. Cα to boron atoms were not observed. ^29^Si NMR (79 MHz, CDCl_3_ δ, ppm): −2.26. ^11^B NMR (128 MHz, CDCl_3_, δ, ppm): 30.07. MS (EI, *m*/*z*): 614(M^+^, 2), 573(4), 557(5), 430(30), 391(2), 361(2), 334(2), 265(3), 195(3), 157(5), 115(7), 83(100), 55(31). FT-IR (cm^–1^): 2977, 2943, 2891, 2865, 1486, 1357, 1327, 1143, 1011, 983, 848. Anal. calcd for C_31_H_49_B_2_O_4_SiBr: C, 60.51; H, 8.03. Found: C, 60.56; H, 8.05. Pale yellow solid. Isolated yield: 68% (104 mg).

#### (*Z*)-(3,4-Bis(4,4,5,5-tetramethyl-1,3,2-dioxaborolan-2-yl)-4-(4-(trifluoromethyl)phenyl)but-3-en-1-yn-1-yl)trisopropylsilane (**3o**)


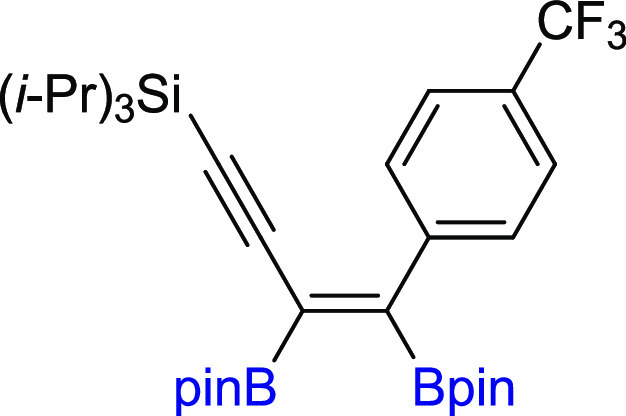
^1^H NMR (300 MHz, CDCl_3_, δ, ppm): 7.51 (s, 4H, Ph), 1.36 (s, 12H, C(CH_3_)_2_), 1.28 (s, 12H, C(CH_3_)_2_), 0.93 (s, 21H, Si(CH(CH_3_)_2_)_3_). ^13^C{^1^H} NMR (101 MHz, CDCl_3_, δ, ppm): 145.1, 129.1, 128.8 (q, *J*^2^_C–F_ = 32.2 Hz), 125.9, 124.6 (q, *J*^3^_C–F_ = 3.7 Hz), 123.2, 106.2 (C≡C), 103.1 (C≡C), 84.7 (C(CH_3_)_2_), 84.5 (C(CH_3_)_2_), 24.9 (C(CH_3_)_2_), 24.9 (C(CH_3_)_2_), 18.6, 11.3. Cα to boron atoms were not observed. ^29^Si NMR (79 MHz, CDCl_3_ δ, ppm): −2.13. ^11^B NMR (128 MHz, CDCl_3_, δ, ppm): 30.82. MS (EI, *m*/*z*): 561(M^+^-43, 33), 505(10), 461(15), 419(3), 379(4), 287(3) 115(6), 83(100), 73(13), 55(31). FT-IR (cm^–1^): 2979, 2943, 2866, 1615, 1555, 1464, 1361, 1323, 1164, 1141, 1067, 984, 882, 850. Anal. calcd for C_32_H_49_B_2_F_3_O_4_Si: C, 63.59; H, 8.17. Found: C, 63.64; H, 8.18. Pale yellow oil. Isolated yield: 74% (111 mg).

#### (*Z*)-(3,4-Bis(4,4,5,5-tetramethyl-1,3,2-dioxaborolan-2-yl)-4-(m-tolyl)but-3-en-1-yn-1-yl)triisopropylsilane (**3p**)


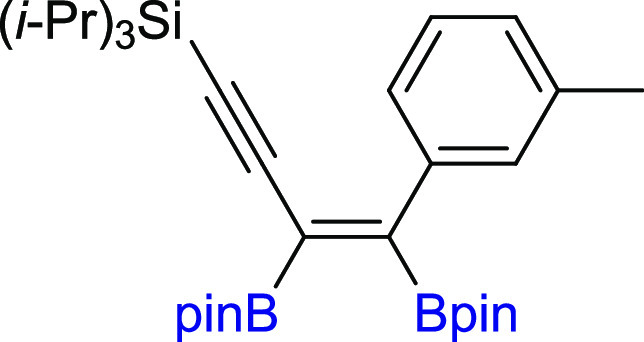
^1^H NMR (300 MHz, CDCl_3_, δ, ppm): 7.21–6.88 (m, 4H, Ph), 2.24 (s, 3H. PhCH_3_), 1.30 (s, 12H, C(CH_3_)_2_), 1.24 (s, 12H, C(CH_3_)_2_), 0.92 (s, 21H, Si(CH(CH_3_)_2_)_3_). ^13^C{^1^H} NMR (101 MHz, CDCl_3_, δ, ppm): 141.1, 136.7, 129.3, 127.8, 127.4, 125.6, 107.0 (C≡C), 100.6 (C≡C), 84.2 (C(CH_3_)_2_), 84.1 (C(CH_3_)_2_), 24.8 (C(CH_3_)_2_), 24.8 (C(CH_3_)_2_), 21.5 (PhCH_3_), 18.6 Si(CH(CH_3_)_2_)_3_), 11.3 Si(CH(CH_3_)_2_)_3_). Cα to boron atoms were not observed. ^29^Si NMR (79 MHz, CDCl_3_ δ, ppm): −2.51. ^11^B NMR (128 MHz, CDCl_3_, δ, ppm): 30.18. MS (EI, *m*/*z*): 550(M^+^, 2), 506(3), 492(4), 406(68), 324(34), 296(15) 252(16), 226(16), 209(11), 115(12), 83(100), 55(61). Anal. calcd for C_32_H_52_B_2_O_4_Si: C, 69.82; H, 9.52. Found: C, 69.92; H, 9.63. Pale yellow oil. Isolated yield: 79% (108 mg).

#### (*Z*)-Trisopropyl(4-(pyren-1-yl)-3,4-bis(4,4,5,5-tetramethyl-1,3,2-dioxaborolan-2-yl)but-3-en-1-yn-1-yl)silane (**3q**)


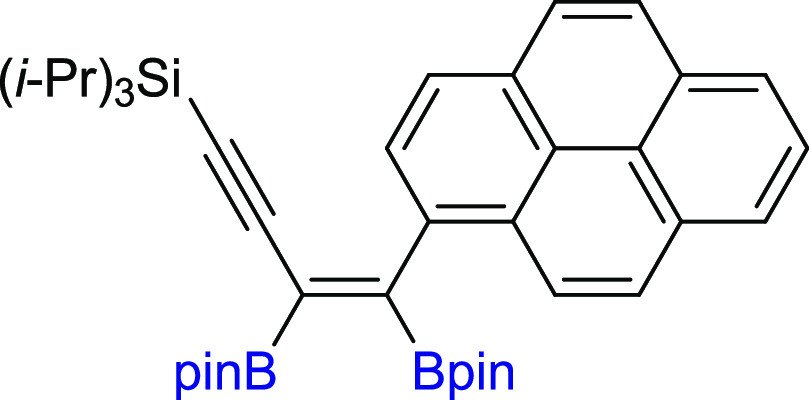
^1^H NMR (300 MHz, CDCl_3_, δ, ppm): 8.10–7.78 (m, 9H, Pyrene), 1.35 (s, 12H, C(CH_3_)_2_), 1.17 (s, 12H, C(CH_3_)_2_), 0.45 (s, 21H, Si(CH(CH_3_)_2_)_3_). ^13^C{^1^H} NMR (75 MHz, CDCl_3_, δ, ppm): 138.2, 131.4, 130.4, 128.1, 127.6, 126.7, 126.5, 126.4, 125.5, 125.2, 125.0, 124.6, 124.5, 106.7 (C≡C), 102.2 (C≡C), 84.5 (C(CH_3_)_2_), 84.4 (C(CH_3_)_2_), 24.7 (C(CH_3_)_2_), 24.8 (C(CH_3_)_2_), 18.2, 11.0. Cα to boron atoms were not observed. ^29^Si NMR (79 MHz, CDCl_3_ δ, ppm): −2.60. ^11^B NMR (128 MHz, CDCl_3_, δ, ppm): 31.07. Anal. calcd for C_41_H_54_B_2_O_4_Si: C, 74.55; H, 8.24. Found: C, 74.11; H, 8.14. Yellow-green oil. Isolated yield: 76% (125 mg).

#### (*Z*)-(4-Cyclopropyl-3,4-bis(4,4,5,5-tetramethyl-1,3,2-dioxaborolan-2-yl)but-3-en-1-yn-1-yl)triisopropylsilane (**3r**)


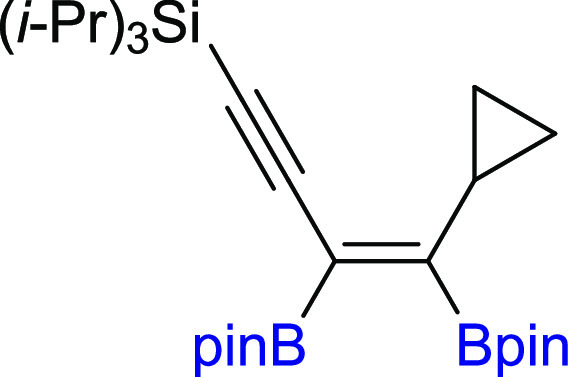
^1^H NMR (300 MHz, CDCl_3_, δ, ppm): 2.50–2.32 (m, 1H, CHCH_2_CH_2_), 1.26 (s, 12H, C(CH_3_)_2_), 1.26 (s, 12H, C(CH_3_)_2_), 1.06 (s, 21H, Si(CH(CH_3_)_2_)_3_), 0.90–0.77 (m, 4H, CHCH_2_CH_2_). ^13^C{^1^H} NMR (101 MHz, CDCl_3_, δ, ppm): 107.4 (C≡C), 98.3 (C≡C), 84.0 (C(CH_3_)_2_), 83.9 (C(CH_3_)_2_), 25.2 (C(CH_3_)_2_), 24.9 (C(CH_3_)_2_), 18.8 Si(CH(CH_3_)_2_)_3_), 11.6 Si(CH(CH_3_)_2_)_3_), 7.9. Cα to boron atoms were not observed. ^29^Si NMR (79 MHz, CDCl_3_ δ, ppm): −2.56. ^11^B NMR (128 MHz, CDCl_3_, δ, ppm): 29.69. MS (EI, *m*/*z*): 500(M^+^, 1), 457(2), 356(22), 275(18), 231(6), 203(7), 157(8), 115(11), 83(100), 55(56). Anal. calcd for C_28_H_50_B_2_O_4_Si: C, 67.21; H, 10.07. Found: C, 67.33; H, 9.98. Pale yellow oil. Isolated yield: 80% (99 mg).

#### (*Z*)-(5-Phenyl-3,4-bis(4,4,5,5-tetramethyl-1,3,2-dioxaborolan-2-yl)pent-3-en-1-yn-1-yl)trisopropylsilane (**3s**)


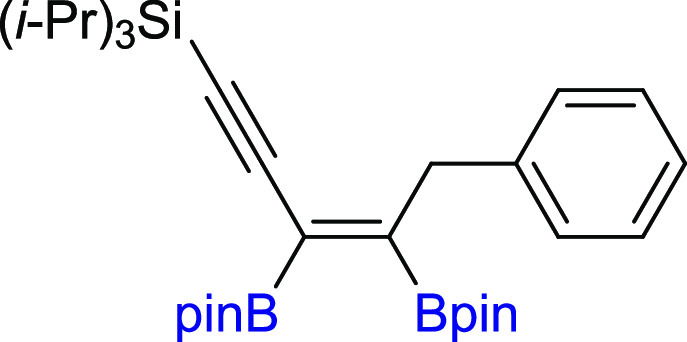
^1^H NMR (300 MHz, CDCl_3_, δ, ppm): 7.39–7.28 (m, 2H, Ph), 7.22–7.03 (m, 3H, Ph), 3.86 (s, 2H, CH_2_Ph), 1.30 (s, 12H, C(CH_3_)_2_), 1.13 (s, 12H, C(CH_3_)_2_), 1.08 (s, 21H, Si(CH(CH_3_)_2_)_3_). ^13^C{^1^H} NMR (75 MHz, CDCl_3_, δ, ppm): 140.5, 129.6, 128.1, 125.8, 106.7 (C≡C), 101.0 (C≡C), 84.1 (C(CH_3_)_2_), 84.0 (C(CH_3_)_2_), 40.9 (CH_2_Ph), 24.9 (C(CH_3_)_2_), 24.8 (C(CH_3_)_2_), 18.8, 11.5. Cα to boron atoms were not observed. ^29^Si NMR (79 MHz, CDCl_3_ δ, ppm): −2.21. ^11^B NMR (128 MHz, CDCl_3_, δ, ppm): 30.24. MS (EI, *m*/*z*): 550(M^+^, 3), 492(3), 450(1), 407(71), 367(4), 325(25), 281(12), 253(10), 195(6), 157(5), 115(8), 83(100), 55(45). FT-IR (cm^–1^): 2976, 2942, 2864, 1573, 1463, 1364, 1338, 1316, 1144, 1128, 1047, 882, 724, 675. Anal. calcd for C_32_H_52_B_2_O_4_Si: C, 69.82; H, 9.52. Found: C, 69.90; H, 9.48. Pale yellow oil. Isolated yield: 81% (111 mg).

#### (*Z*)-(5-Phenoxy-3,4-bis(4,4,5,5-tetramethyl-1,3,2-dioxaborolan-2-yl)pent-3-en-1-yn-1-yl)trisopropylsilane (**3t**)


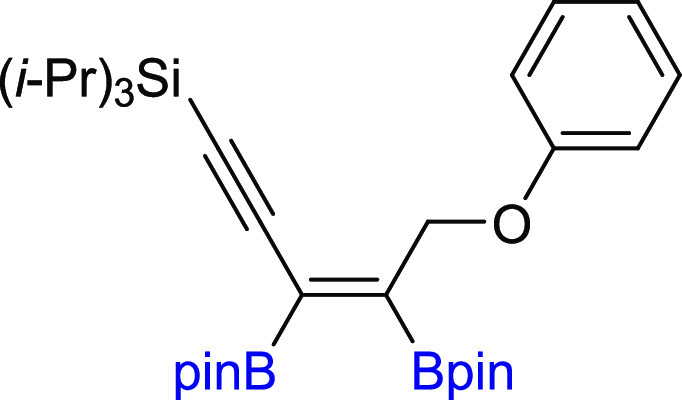
^1^H NMR (300 MHz, CDCl_3_, δ, ppm): 7.40–7.21 (m, 2H, Ph), 7.06–6.86 (m, 3H, Ph), 5.08 (s, 2H, CH_2_OPh), 1.35 (s, 12H, C(CH_3_)_2_), 1.33 (s, 12H, C(CH_3_)_2_), 1.14 (s, 21H, Si(CH(CH_3_)_2_)_3_). ^13^C{^1^H} NMR (151 MHz, CDCl_3_, δ, ppm): 158.8, 151.1 (Cα to boron), 129.4, 125.5 (Cα to boron), 120.5, 114.8, 105.2 (C≡C), 102.8 (C≡C), 84.4 (C(CH_3_)_2_), 84.3 (C(CH_3_)_2_), 25.0 (C(CH_3_)_2_), 24.9 (C(CH_3_)_2_), 18.8, 11.4. Cα to boron atoms were observed after 21,371 number of scans as weak broad peaks. ^29^Si NMR (79 MHz, CDCl_3_ δ, ppm): −1.82. ^11^B NMR (128 MHz, CDCl_3_, δ, ppm): 28.54. MS (EI, *m*/*z*): 566(M^+^, 3), 523(2), 423(10), 331(4), 253(6), 173(11), 115(8), 83(100), 55(45). FT-IR (cm^–1^): 2942, 2864, 1599, 1495, 1462, 1366, 1240, 1213, 1070, 1031, 882, 803, 751, 676, 541. Anal. calcd for C_32_H_52_B_2_O_5_Si: C, 67.85; H, 9.25. Found: C, 67.91; H, 9.31. Pale yellow oil. Isolated yield: 66% (93 mg).

#### (*Z*)-(3,4-Bis(4,4,5,5-tetramethyl-1,3,2-dioxaborolan-2-yl)dec-3-en-1-yn-1-yl)trisopropylsilane (**3u**)


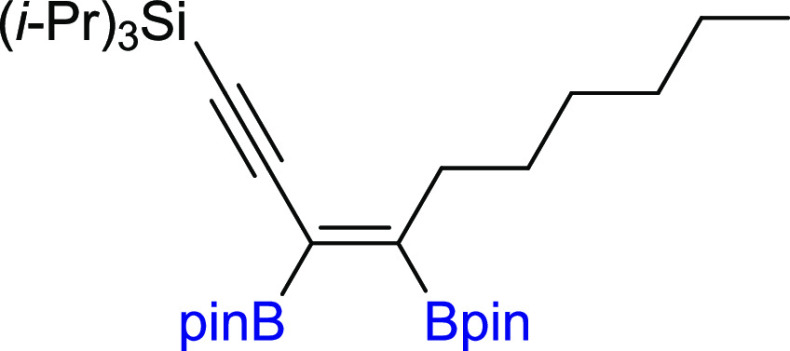
^1^H NMR (400 MHz, CDCl_3_, δ, ppm): 2.54–2.40 (m, 2H, C=CCH_2_), 1.44–1.36 (m, 3H), 1.30 (s, 12H, C(CH_3_)_2_), 1.29–1.24 (m, 17H), 1.06 (s, 21H, Si(CH(CH_3_)_2_)_3_), 0.86 (t, *J*_H–H_ = 6.8 Hz, 3H, CH_2_CH_3_). ^13^C{^1^H} NMR (101 MHz, CDCl_3_, δ, ppm): 106.3 (C≡C), 100.6 (C≡C), 84.0 (C(CH_3_)_2_), 83.9 (C(CH_3_)_2_), 35.2, 32.0, 29.8, 29.4, 24.9 (C(CH_3_)_2_), 24.9 (C(CH_3_)_2_), 22.8, 18.8, 18.7, 14.3, 11.5, 11.5. Cα to boron atoms were not observed. ^29^Si NMR (79 MHz, CDCl_3_ δ, ppm): −2.35. ^11^B NMR (128 MHz, CDCl_3_, δ, ppm): 30.77. MS (EI, *m*/*z*): 544(M^+^, 2), 529(1), 501(2), 401(56), 344(3), 319(25), 249(4), 157(6), 115(10), 83(100), 55(31). FT-IR (cm^–1^): 2943, 2866, 1716, 1462, 1683, 882, 677. Anal. calcd for C_31_H_58_B_2_O_4_Si: C, 68.38; H, 10.74. Found: C, 68.43; H, 10.72. Pale yellow oil. Isolated yield: 72% (97 mg).

#### (*Z*)-Trimethyl(4-phenyl-3,4-bis(4,4,5,5-tetramethyl-1,3,2-dioxaborolan-2-yl)but-3-en-1-yn-1-yl)silane (**3v**)


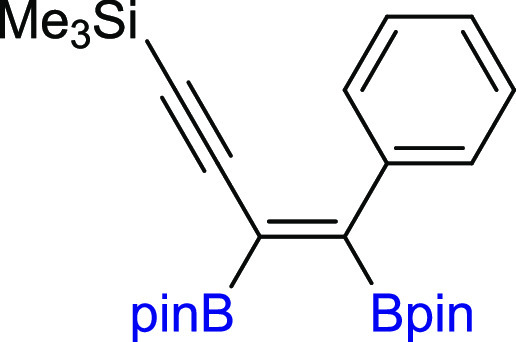
^1^H NMR (300 MHz, CDCl_3_, δ, ppm): 7.52–7.46 (m, 2H, Ph), 7.33–7.21 (m, 3H, Ph)1.35 (s, 12H, C(CH_3_)_2_), 1.29 (s, 12H, C(CH_3_)_2_), 0.05 (s, 9H, Si(CH_3_)_3_). ^13^C{^1^H} NMR (75 MHz, CDCl_3_, δ, ppm): 141.0, 128.7, 127.5, 127.3, 105.5 (C≡C), 103.7 (C≡C), 84.4 (C(CH_3_)_2_), 24.9 (C(CH_3_)_2_), 24.8 (C(CH_3_)_2_), −0.1 (Si(CH_3_)_3_. Cα to boron atoms were not observed. ^29^Si NMR (79 MHz, CDCl_3_ δ, ppm): −18.30. ^11^B NMR (128 MHz, CDCl_3_, δ, ppm): 29.98. MS (EI, *m*/*z*): 552(M^+^, 1), 473(1), 395(10), 337(1), 313(4), 295(5), 269(4), 254(3), 225(3), 198(7), 183(8), 143(5), 84(100), 73(20), 69(12), 55(12). FT-IR (cm^–1^): 2986, 1676, 1473, 1371, 1328, 1264, 1140, 981, 847, 757, 734, 701. Anal. calcd for C_25_H_38_B_2_O_4_Si: C, 66.39; H, 8.47. Found: C, 66.44; H, 8.50. Pale yellow oil. Isolated yield: 83% (93 mg).

#### (*Z*)-2,2′-(2,2-Dimethyldodec-5-en-3-yne-5,6-diyl)bis(4,4,5,5-tetramethyl-1,3,2-dioxaborolane) (**3w**)


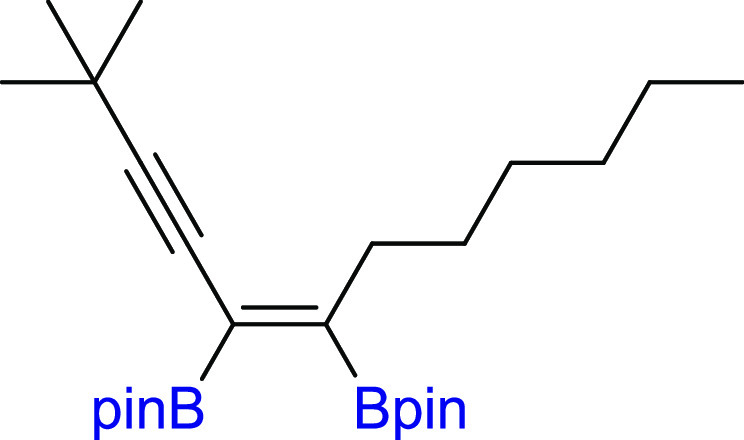
^1^H NMR (400 MHz, CDCl_3_, δ, ppm): 1.33–1.24 (m, 20H), 1.24 (s, 12H, C(CH_3_)_2_), 1.23 (s, 9H, C(CH_3_)_3_), 0.89–0.83 (m, 3H, CH_2_CH_3_). ^13^C{^1^H} NMR (101 MHz, CDCl_3_, δ, ppm): 108.9 (C≡C), 83.8 (C(CH_3_)_2_), 83.7 (C(CH_3_)_2_), 78.5 (C(CH_3_)_3_), 34.4, 32.0, 31.2, 29.6, 29.2, 28.6, 24.9 (C(CH_3_)_2_), 24.8 (C(CH_3_)_2_), 22.8, 14.3. Cα to boron atoms were not observed. ^11^B NMR (128 MHz, CDCl_3_, δ, ppm): 30.25. MS (EI, *m*/*z*): 444(M^+^, 1), 429(1), 387(2), 361(2), 329(2), 303(4), 261(3), 235(4), 191(6), 177(10), 133(7), 105(6), 83(100), 69(15), 55(20). FT-IR (cm^–1^): 2959, 2929, 2860, 1679, 1459, 1368, 1343, 1310, 1260, 1134, 966, 854, 542. Anal. calcd for C_26_H_46_B_2_O_4_: C, 70.29; H, 10.44. Found: C, 70.22; H, 10.39. Pale yellow oil. Isolated yield: 73% (81 mg).

#### (*Z*)-2,2′-(1-Phenyldec-3-en-1-yne-3,4-diyl)bis(4,4,5,5-tetramethyl-1,3,2-dioxaborolane) (**3x**) and (*Z*)-2,2′-(1-Phenyldec-1-en-3-yne-1,2-diyl)bis(4,4,5,5-tetramethyl-1,3,2-dioxaborolane) (**3′x**)


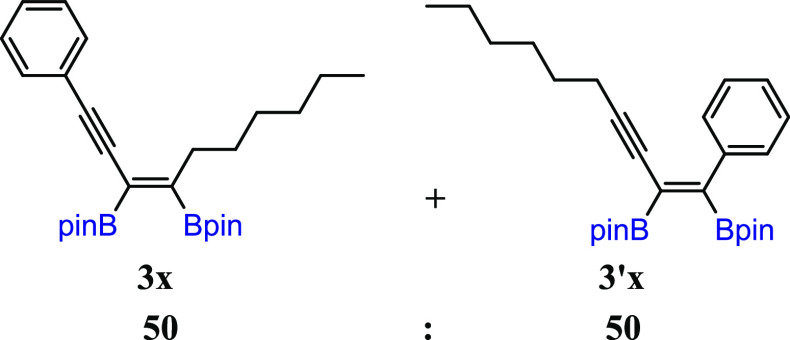
^1^H NMR (400 MHz, CDCl_3_, δ, ppm): 7.62–7.15 (m, 10H, Ph, (**3x** + **3′x**)), 2.61–2.55 (m, 2H, CCH_2_CH_2,_**3x**), 2.25 (t, *J*_H–H_ = 6.8 Hz, 2H, CCH_2_CH_2,_**3′x**), 1.54–1.39 (m, 6H, (**3x** + **3′x**)), 1.37 (s, 12H, C(CH_3_)_2_, **3x**), 1.36 (s, 12H, C(CH_3_)_2_, **3x**), 1.32 (s, 12H, C(CH_3_)_2_, **3′x**), 1.31 (s, 12H, C(CH_3_)_2_, **3′x**), 1.29–1.21 (m, 8H, (**3x** + **3′x**)), 1.00–0.80 (m, 6H, CH_2_CH_3_, (**3x** + **3′x**)). ^13^C{^1^H} NMR (101 MHz, CDCl_3_, δ, ppm): 141.5, 132.6, 132.3, 132.2, 132.1, 132.0, 131.6, 128.7, 128.7, 128.5, 128.4, 128.4, 128.2, 127.8, 127.5, 126.9, 124.6, 99.6, 98.0, 89.2, 84.2 (C(CH_3_)_2_), 84.2 (C(CH_3_)_2_), 84.1 (C(CH_3_)_2_), 83.9 (C(CH_3_)_2_), 83.1, 81.1, 35.3, 31.9, 31.5, 29.6, 29.1, 28.6, 28.5, 24.9 (C(CH_3_)_2_), 24.9 (C(CH_3_)_2_), 24.6 (C(CH_3_)_2_), 24.8 (C(CH_3_)_2_), 24.7, 22.7, 22.7, 20.2, 14.2, 14.2. Cα to boron atoms were not observed. MS (EI, *m*/*z*): for **3x** or **3′x**: 464(M^+^, 1), 407(2), 381(7), 324(3), 281(8), 253(6), 211(6), 181(17), 167(10), 128(9), 84(100), 55(15); for **3x** or **3′x**: 464(M^+^, 2), 407(6), 322(3), 253(3), 210(10), 195(6), 167(10), 141(5), 129(8), 84(100), 55(22). FT-IR (cm^–1^): 2955, 2928, 2857, 1687, 1598, 1581, 1490, 1449, 1371, 1313, 1263, 1241, 1142, 1120, 1007, 982, 852, 755, 722, 590, 541. Obtained and characterized as reaction mixture (**3x**/**3′x** = 50/50) in 81% yield (93 mg).

#### (*Z*)-2,2′-(1-(o-Tolyl)dec-3-en-1-yne-3,4-diyl)bis(4,4,5,5-tetramethyl-1,3,2-dioxaborolane) (**3y**) and (*Z*)-2,2′-(1-(o-Tolyl)dec-1-en-3-yne-1,2-diyl)bis(4,4,5,5-tetramethyl-1,3,2-dioxaborolane) (**3′y**)


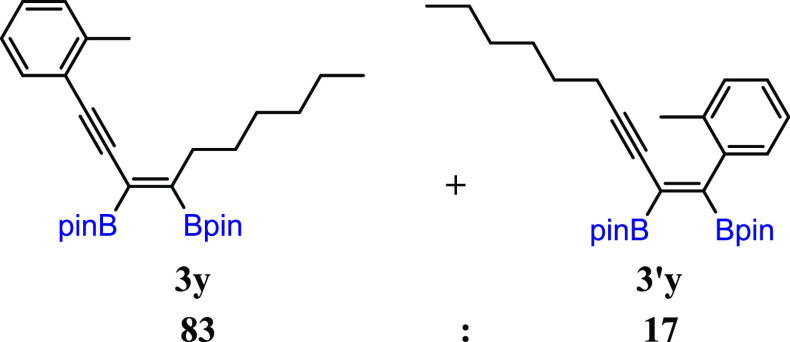
^1^H NMR (400 MHz, CDCl_3_, δ, ppm): 7.44–7.35 (m, 1H, Ph (**3x** + **3′x**)), 7.23–7.05 (m, 4H, Ph, (**3y** + **3′y**)), 2.63–2.51 (m, 2H, CCH_2_CH_2_, (**3y**)), 2.45 (s, 3H, PhCH_3_, **3x**)), 2.23 (s, 0.6H, PhCH_3_, **3′y**)), 2.13 (t, *J*_H–H_ = 6.7 Hz, 0.43H, CCH_2_CH_2_, (**3′y**)), 1.64–1.33 (m, 6H, (**3y** + **3′y**)), 1.33 (s, 12H, C(CH_3_)_2_, **3y**), 1.30 (s, 12H, C(CH_3_)_2_, **3x**), 1.26 (s, 3H, C(CH_3_)_2_, **3′y**), 1.23 (s, 3H, C(CH_3_)_2_, **3′y**), 1.01–0.78 (m, 4H, CH_2_CH_3_, (**3y** + **3′y**)). MS (EI, *m*/*z*): for **3y**: 478(M^+^, 7), 422(3), 395(6), 337(2), 308(2), 295(11), 267(6), 224(46), 195(13), 181(9), 101(8), 84(100), 55(26); for **3′y**: 478(M^+^, 1), 421(5), 395(2), 337(2), 308(3), 295(3), 267(3), 224(42), 195(19), 181(6), 101(5), 84(100), 55(24). FT-IR (cm^–1^): 2976, 2956, 2928, 2857, 1705, 1601, 1456, 1369, 1344, 1313, 1255, 1214, 1145, 1131, 850, 756. Obtained and characterized as reaction mixture (**3y**/**3′y** = 83/17) in 69% yield (82 mg).

#### (*Z*)-2,2′-(1-(2,6-Dimethylphenyl)dec-3-en-1-yne-3,4-diyl)bis(4,4,5,5-tetramethyl-1,3,2-dioxaborolane) (**3z**)


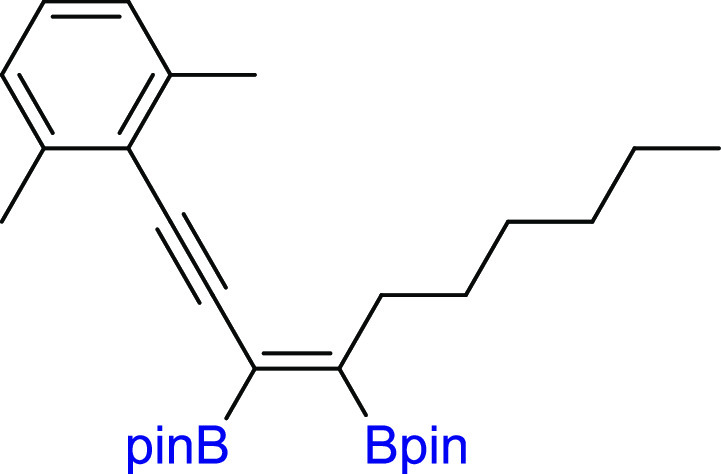
^1^H NMR (600 MHz, CDCl_3_, δ, ppm): 7.10–7.05 (m, 1H, Ph), 7.05–7.00 (m, 2H, Ph), 2.62–2.55 (m, 2H, (pin)BC=CB(*pin*)CH_2_CH_2_), 2.45 (s, 6H, PhCH_3_), 1.48 (m, 2H, (pin)BC=CB(*pin*)CH_2_CH_2_), 1.38–1.33 (m, 2H), 1.33 (s, 12H, C(CH_3_)_2_), 1.30 (s, 12H, C(CH_3_)_2_), 1.30–1.25 (m, 4H), 0.88–0.83 (m, 3H). ^13^C{^1^H} NMR (151 MHz, CDCl_3_, δ, ppm): 155.9 ((pin)BC=CB(*pin*)CH_2_CH_2_) based on HMBC NMR), 140.4, 127.5, 126.6, 125.9 ((pin)BC=CB(*pin*)CH_2_CH_2_) based on HMBC NMR), 124.3, 97.9, 96.2, 84.1, 83.9, 35.7, 32.0, 29.9, 29.3, 25.0, 24.8, 22.7, 21.3, 14.2. Cα to boron atoms were observed after 5556 number of scans as weak broad peaks. ^11^B NMR (128 MHz, CDCl_3_, δ, ppm): 30.93. MS (EI, *m*/*z*): 492(M^+^, 9), 435(13), 361(7), 309(13), 253(11), 238(100), 196(17), 167(12), 118(6), 84(91), 55(30). Anal. calcd for C_30_H_46_B_2_O_4_: C, 73.19; H, 9.42. Found: C, 73.27; H, 9.45. Pale yellow oil. Isolated yield: 64% (78 mg).

#### 2,2′,2″,2‴-((2*Z*,4*Z*)-Hexa-2,4-diene-2,3,4,5-tetrayl)tetrakis(4,4,5,5-tetramethyl-1,3,2-dioxaborolane) (**4d**)


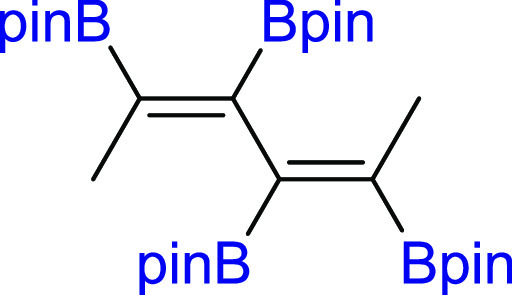
^1^H NMR (400 MHz, CDCl_3_, δ, ppm): 1.63 (s, 6H, CH_3_), 1.29 (s, 24H, C(CH_3_)_2_), 1.22 (s, 24H, C(CH_3_)_2_). ^13^C{^1^H} NMR (101 MHz, CDCl_3_, δ, ppm): 83.3 (C(CH_3_)_2_), 83.2 (C(CH_3_)_2_), 25.0 (C(CH_3_)_2_), 24.9 (C(CH_3_)_2_), 24.9 (C(CH_3_)_2_), 18.5. ^11^B NMR (128 MHz, CDCl_3_, δ, ppm): 30.13. MS (EI, *m*/*z*): 571(M^+^-15, 0.1), 527(1), 445(4), 345(5), 328(6), 287(5), 244(5), 205(4), 163(4), 83(100), 69(28), 55(40). Anal. calcd for C_30_H_54_B_4_O_8_: C, 61.49; H, 9.29. Found: C, 61.55; H, 9.34. White solid. Isolated yield: 42% (61 mg).

#### 2,2′,2″,2‴-((7-Z,9-Z)-Hexadeca-7,9-diene-7,8,9,10-tetrayl)tetrakis(4,4,5,5-tetramethyl-1,3,2-dioxaborolane) (**4e**)


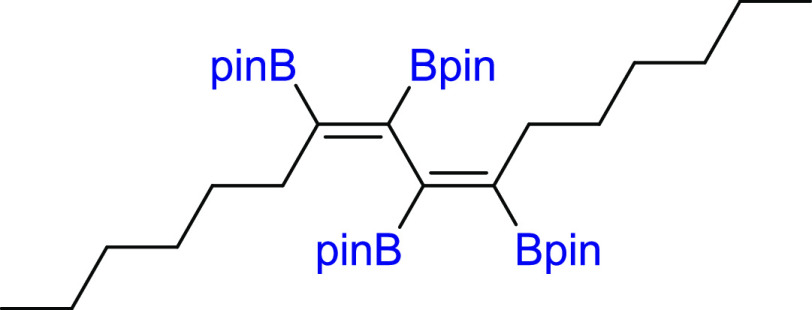
^1^H NMR (400 MHz, CDCl_3_, δ, ppm): 2.19–1.89 (m, 4H), 1.38–1.30 (m, 4H), 1.29 (s, 24H, C(CH_3_)_2_), 1.27–1.21 (m, 12H), 1.20 (s, 12H, C(CH_3_)_2_), 1.20 (s, 12H, C(CH_3_)_2_), 0.85 (t, *J*_H–H_ = 6.8 Hz, 6H). ^13^C{^1^H} NMR (101 MHz, CDCl_3_, δ, ppm): 83.0 (C(CH_3_)_2_), 82.9 (C(CH_3_)_2_), 33.3, 32.0, 29.7, 28.7, 25.0 (C(CH_3_)_2_), 24.9 (C(CH_3_)_2_), 24.7 (C(CH_3_)_2_), 24.6 (C(CH_3_)_2_), 22.6, 14.2, 14.1, 1.0. ^11^B NMR (128 MHz, CDCl_3_, δ, ppm): 31.10. MS (EI, *m*/*z*): 669(M^+^-57, 1), 626(3), 568(5), 526(5), 442(3), 389(9), 289(6), 175(6), 129(17), 83(100), 55(55). Anal. calcd for C_40_H_74_B_4_O_8_: C, 66.15; H, 10.27. Found: C, 66.55; H, 10.34. White solid. Isolated yield: 61% (110 mg).

#### (*Z*)-(1,2-Diphenylbut-1-en-3-yne-1,4-diyl)bis(trimethylsilane) (**6**)


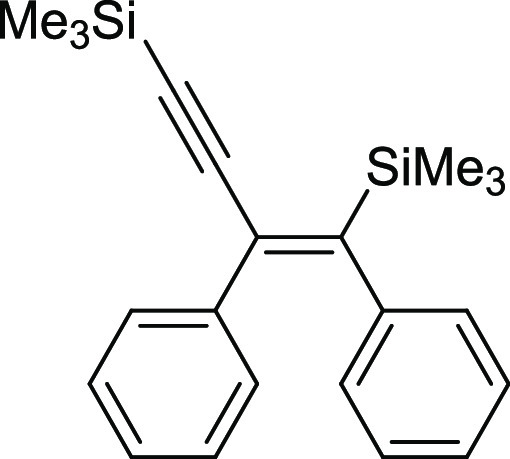
^1^H NMR (400 MHz, CDCl_3_, δ, ppm): 7.17–7.00 (m, 8H, Ph), 6.84–6.73 (m, 2H, Ph), 0.24 (s, 9H, Si(CH_3_)_3_), 0.23 (s, 9H, Si(CH_3_)_3_. ^13^C{^1^H} NMR (101 MHz, CDCl_3_, δ, ppm): 154.7, 143.1, 139.6, 133.2, 129.5, 128.1, 127.9, 127.5, 126.8, 125.5, 107.5 (C≡C), 99.7 (C≡C), −0.2 (Si(CH_3_), −0.4 (Si(CH_3_). ^29^Si NMR (79 MHz, CDCl_3_ δ, ppm): −4.06 (C=CSiMe_3_), −18.16 (C≡CSiMe_3_). MS (EI, *m*/*z*): 348(M+, 52), 333(12), 275(35), 260(12), 245(11), 179(12), 155(58), 135(), 73(100). Anal. calcd for C_22_H_28_Si_2_: C, 75.79; H, 8.10. Found: C, 75.89; H, 8.17. Pale yellow oil. Isolated yield: 74% (25 mg).

#### ((3*Z*,5*E*)-6-Phenylhexa-3,5-dien-1-yne-1,4-diyl)bis(trimethylsilane) (**7a**)


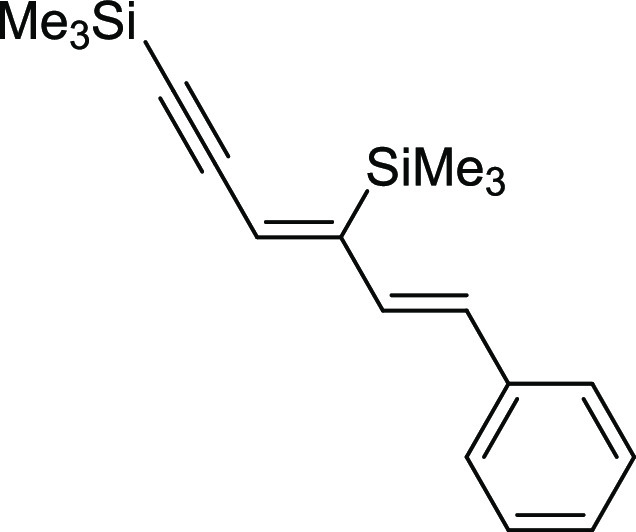
^1^H NMR (400 MHz, CDCl_3_, δ, ppm): 7.43–7.37 (m, 2H, Ph), 7.32 (t, *J*_H–H_ = 7.6 Hz, 2H, Ph), 7.27–7.19 (m, 1H, Ph), 6.85 (d, *J*_H–H_ = 14.8 Hz, 1H, CH=CH), 6.66 (d, *J*_H–H_ = 16.0 Hz, 1H, CH=CH), 6.35 (s, 1H, =CH), 0.35 (s, 9H, (Si(CH_3_)), 0.22 (s, 9H, (Si(CH_3_)).^13^C{^1^H} NMR (101 MHz, CDCl_3_, δ, ppm): 154.2 (C=CH), 137.6, 133.3, 130.3, 128.8, 127.8, 126.7, 119.9 (C=CH), 105.9 (C≡C), 102.2 (C≡C), −0.1 (Si(CH_3_), −0.2 (Si(CH_3_). ^29^Si NMR (79 MHz, CDCl_3_ δ, ppm): −4.12 (C=CSiMe_3_), −18.27 (C≡CSiMe_3_). MS (EI, *m*/*z*): 298(M^+^, 4), 283(18), 209(11), 195(20), 155(58), 128(4), 73(100). Anal. calcd for C_18_H_26_Si_2_: C, 72.41; H, 8.78. Found: C, 72.30; H, 8.82. Pale yellow oil. Isolated yield: 79% (23 mg).

#### ((3*Z*,5*E*)-6-(4-Methoxyphenyl)hexa-3,5-dien-1-yne-1,4-diyl)bis(trimethylsilane) (**7b**)


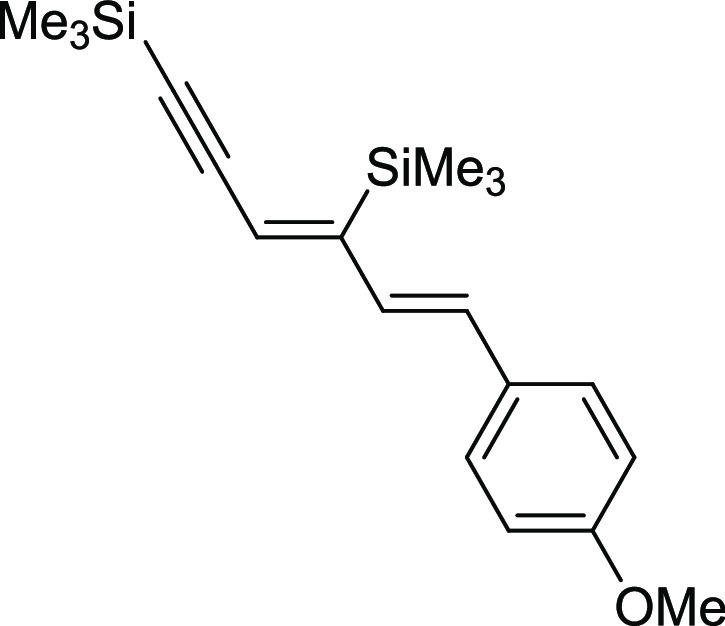
^1^H NMR (400 MHz, CDCl_3_, δ, ppm): 7.36 (d, *J*_H–H_ = 8.8 Hz, 2H, Ph), 6.88 (d, *J*_H–H_ = 8.8 Hz, 2H, Ph), 6.75 (d, *J*_H–H_ = 16.0 Hz, 1H, CH=CH), 6.65 (d, *J*_H–H_ = 16.0 Hz, 1H, CH=CH), 6.33 (s, 1H, =CH), 3.83 (s, 3H, OCH_3_), 0.36 (s, 9H, Si(CH_3_)_3_), 0.23 (s, 9H, Si(CH_3_)_3_).^13^C{^1^H} NMR (101 MHz, CDCl_3_, δ, ppm): 159.5, 154.4, 144.5, 131.2, 130.4, 129.8, 127.9, 127.4, 119.0, 114.2, 106.1 (C≡C), 101.8 (C≡C), 55.5 (OCH_3_), −0.1 (Si(CH_3_), −0.1 (Si(CH_3_). ^29^Si NMR (79 MHz, CDCl_3_ δ, ppm): −4.18 (C=CSiMe_3_), −18.38 (C≡CSiMe_3_). MS (EI, *m*/*z*): 328(M^+^, 8), 313(12), 239(7), 225(21), 209(6), 165(7), 115(6), 73(100). Anal. calcd for C_19_H_28_OSi_2_: C, 69.45; H, 8.59. Found: C, 69.55; H, 8.64. Pale yellow oil. Isolated yield: 66% (21 mg).

#### ((1*Z*,3*E*)-1,2-Bis(4,4,5,5-tetramethyl-1,3,2-dioxaborolan-2-yl)-3-(triethylsilyl)buta-1,3-diene-1,4-diyl)bis(trimethylsilane) (**8**)


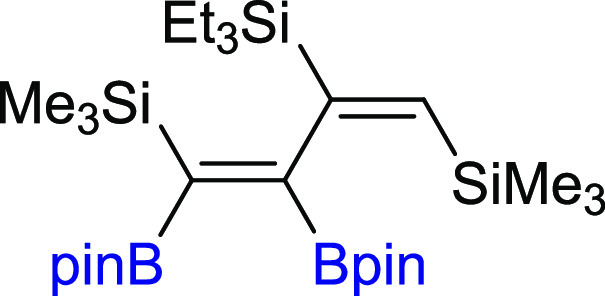
^1^H NMR (300 MHz, CDCl_3_, δ, ppm): 7.38–7.06 (m, 1H), 1.20 (s, 12H, C(CH_3_)_2_), 1.17 (s, 12H, C(CH_3_)_2_), 0.95 (t, *J*_H–H_ = 7.8 Hz, 9H, Si(CH_2_CH_3_)_3_), 0.65–0.56 (m, 6H, SiCH_2_CH_3_)_3_), 0.12 (s, 9H, SiCH_3_)_3_), 0.08 (s, 9H, SiCH_3_)_3_). ^13^C{^1^H} NMR (101 MHz, CDCl_3_, δ, ppm): 128.4, 126.4, 82.7 (C(CH_3_)_2_), 82.6 (C(CH_3_)_2_), 25.4 (C(CH_3_)_2_), 25.2 (C(CH_3_)_2_), 25.1 (C(CH_3_)_2_), 24.7 (C(CH_3_)_2_), 7.6, 4.7, 0.7, 0.0. ^11^B NMR (128 MHz, CDCl_3_, δ, ppm): 34.41. ^29^Si NMR (79 MHz, CDCl_3_ δ, ppm): 3.77, 3.32, −3.74. MS (EI, *m*/*z*): 564(M^+^, 21), 549(3), 423(6), 397(6), 319(6), 293(11), 267(7), 231(9), 175(11), 115(59), 84(100), 59(34). Anal. calcd for C_16_H_34_Si_3_: C, 61.85; H, 11.03. Found: C, 61.77; H, 10.98. Pale yellow oil. Isolated yield: 63% (20 mg).

#### (*Z*)-(1,2-Bis(4,4,5,5-tetramethyl-1,3,2-dioxaborolan-2-yl)-4-(p-tolyl)but-1-en-3-yn-1-yl)trimethylsilane (**9**)


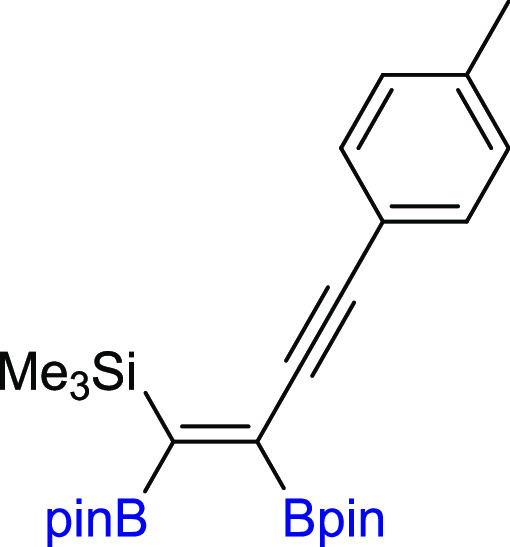
^1^H NMR (300 MHz, CDCl_3_, δ, ppm): 7.32 (d, 2H, *J*_H–H_ = 8.1 Hz), 7.10 (d, 2H, *J*_H–H_ = 7.8 Hz), 2.34 (s, 3H, CH_3_), 1.34 (s, 12H, C(CH_3_)_2_), 1.31 (s, 12H, C(CH_3_)_2_), 0.29 (s, 9H, SiCH_3_)_3_). ^13^C{^1^H} NMR (101 MHz, CDCl_3_, δ, ppm): 138.1, 131.4, 129.1, 121.4, 97.7 (C≡C), 92.5 (C≡C) 84.5 (C(CH_3_)_2_), 83.8 (C(CH_3_)_2_), 25.6 (C(CH_3_)_2_), 24.9 (C(CH_3_)_2_), 21.6 (PhCH_3_), −0.2 (Si(CH_3_)_3_). ^11^B NMR (128 MHz, CDCl_3_, δ, ppm): 29.51 (bs). ^29^Si NMR (79 MHz, CDCl_3_ δ, ppm): −6.34. MS (EI, *m*/*z*): 466(M^+^, 1), 451(1), 351(2), 308(4), 212(40), 197(27), 173(13), 84(100), 69(35). Anal. calcd for C_26_H_40_B_2_O_4_Si: C, 66.97; H, 8.65. Found: C, 67.01; H, 8.69. Pale yellow oil. Isolated yield: 81% (31 mg).

## Supporting Information Available

Purification procedures; starting material and **3a** functionalization synthetic protocols; and NMR spectra of all synthesized compounds (PDF)
