# Supplementary material for: [Pt(PPh3)4]-Catalyzed Selective Diboration of Symmetrical and Unsymmetrical 1,3-Diynes
Source: J Org Chem. 2022 Aug 2;87(16):10651–63. doi: 10.1021/acs.joc.2c00844 (PMC9396666; doi:10.1021/acs.joc.2c00844)
Supplement: Supplementary file 1 — jo2c00844_si_001.pdf [file jo2c00844_si_001.pdf]

## Supporting Information

# [Pt(PPh$_3$)$_4$]-catalyzed selective diboration of symmetrical and unsymmetrical 1,3-diynes

Jakub Szyling,*,†,‡ Aleksandra Szymańska,†,‡ Adrian Franczyk,† Jędrzej Walkowiak*,†

†Center for Advanced Technology, Adam Mickiewicz University in Poznan, Uniwersytetu Poznanskiego 10, 61-614 Poznan, Poland

‡Faculty of Chemistry, Adam Mickiewicz University in Poznan, Uniwersytetu Poznanskiego 8, 61-614 Poznan, Poland

*E-mail: j.szyling@amu.edu.pl , jedrzej.walkowiak@amu.edu.pl

**Outline**

1. Materials ........ S2
2. General information ........ S2
 2.1. Products purification ........ S2
  2.1.1. 1,3-Diynes (**2a**-**z**) ........ S2
  2.1.2. Bisboryl-functionalized enynes (**3a**-**z**) ........ S3
3. General procedures for starting materials and product **3a** functionalization ........ S3
 3.1. Synthesis of alkynyl bromides ........ S3
 3.2. Suzuki coupling (for products **6**-**7**) ........ S4
 3.3. Hydrosilylation (for product **8**) ........ S4
 3.4. Sila-Sonogashira (for product **9**) ........ S4
 3.5. Mechanistic studies ........ S4
4. Starting materials and diynes characterization ........ S4
5. NMR spectra. ........ S10
6. References ........ S97

## 1. Materials

Phenylacetylene (98%, Sigma-Aldrich), 1-bromo-4-ethynylbenzene (97%, Sigma-Aldrich), 1-ethynyl-4-fluorobenzene (99%, Sigma-Aldrich), 1-ethynyl-4-methoxybenzene (97%, Sigma-Aldrich), 1-ethynyl-4-(trifluoromethyl)benzene (98%, Sigma-Aldrich), 1-ethynyl-2-methylbenzene (97%, Sigma-Aldrich), 2-ethynyl-1,3-dimethylbenzene (abcr, 97%) 1-octyne (97%, Sigma-Aldrich), ethynyltrimethylsilane (98%, abcr), ethynyltriethylsilane (97%, Sigma-Aldrich) (triisopropylsilyl)acetylene (97%, Sigma-Aldrich), 3-phenyl-1-propyne (97%, Sigma-Aldrich), 3-phenoxy-1-propyne (90%, Sigma-Aldrich), prop-2-yn-1-ol (99%, Sigma-Aldrich), 3,3-dimethylbut-1-yne (98%, Sigma-Aldrich), iodobenzene (98%, Sigma-Aldrich), triethylsilane 99%, Sigma-Aldrich), 1,4-diphenylbuta-1,3-diyne (99%, Sigma-Aldrich), hexa-2,4-diyne (99%, abcr), 1,4-bis(trimethylsilyl)buta-1,3-diyne (98%, Sigma-Aldrich), 1,6-diphenoxyhexa-2,4-diyne (98%, Alfa Aesar), bis(pinacolato)diboron (99%, TCI), N-bromosuccinimide (98%, Sigma-Aldrich), hydroxylamine hydrochloride (98%, abcr), tributylamine (99%, Sigma-Aldrich), piperidine (99%, TCI), ammonium chloride (99%, Avantor Performance Materials Poland), 1,1,1,3,3,3-hexamethyldisilazane (99%, Sigma-Aldrich), silver nitrate (99%, Sigma-Aldrich), 2-dicyclohexylphosphino-2′,4′,6′-triisopropylbiphenyl (98%, Sigma-Aldrich), magnesium sulfate (anhydrous, 99%, Sigma-Aldrich), cesium carbonate (99%, Sigma-Aldrich), hexamethyldisilazane (98%, abcr), silica gel (MN-Kieselgel 60, 0.04-0.063 mm (230-400 mesh ASTM; Sigma-Aldrich)) were used as received. Toluene, tetrahydrofurane (THF), *n*-hexane, hexanes, ethyl acetate, acetone, were purchased from Avantor Performance Materials Poland. The solvents used in the reactions (toluene and THF) were dried, deoxygenated (SP5-800 MBraun) and stored over molecular sieves 4 Å under argon atmosphere. Argon (99,999%) was purchased from Messer. Platinum(IV) oxide (surface area ≥60 $m^2/g$, Sigma-Aldrich), platinum on carbon (10 wt. % loading, Sigma-Aldrich), platinum(0)-1,3-divinyl-1,1,3,3-tetramethyldisiloxane complex solution in xylene (Pt ~2 %, Sigma-Aldrich), hexachloroplatinic acid hexahydrate (Sigma-Aladrich), platinum(II) chloride (99.9%, Sigma-Aldrich), tetrakis(triphenylphosphine)palladium(0) (99%, Sigma-Aladrich) were used as received. Tetrakis(triphenylphosphine)platinum(0) was synthesized according to the literature.[1]

## 2. General information:

### 2.1. Products purification

#### 2.1.1. 1,3-Diynes (2a-z)

The UV-absorbing products (1,3-diynes) were purified on silica by flash chromatography (Biotage IsoleraOne chromatograph) with UV detector ($\lambda 1$ = 255 nm, $\lambda 2$= 280 nm). Purification details: cartridge 10 g, flow rate: 12 mL/min, length: 10 CV (CV = column volume), phase: *n*-hexane/dichloromethane (step 1: *n*-hexane 100% by 4 CV, step 2: gradient 10%/CV by 4 CV, step 3: *n*-hexane 50% by 2 CV). The non-aromatic products (1,3-diynes) were purified on silica using standard column chromatography using *n*-hexane/dichloromethane (95/5–7/3) as eluents. Products were characterized by GC-MS, $^{1}H$, $^{13}C$, $^{29}Si$ NMR, FT-IR analyses.

**2.1.2. Bisboryl-functionalized enynes (3a-z)**

The reaction mixture was evaporated to remove all volatiles. Subsequently, the crude product was dissolved in *n*-pentane and filtered through the syringe filter (0.2 μm). After evaporation of *n*-pentane, the product was heated (approx. 70-130 °C) and condensed at cold-finger trap under vacuum (<$10^{-3}$ mbar) (see Scheme 1). The products were obtained as solids (symmetrical substituted 1,3-diynes, excluding **3b**) or oils (unsymmetrical substituted 1,3-diynes). Products **3n** and **3q** could not be condensed on a cold finger trap and were analyzed after the filtration step.

*Purification of **3h-i**:* The reaction mixture was evaporated to remove all volatiles. Subsequently, the crude product was dissolved in *n*-pentane and filtered through the syringe filter (0.2 μm) and placed in the freezer (-18 °C) in 24 hours. The **3h-i** precipitate as white solids.

*Purification of **3d-e** and **4d-e**:* The reaction mixture was evaporated to remove all volatiles. Subsequently, the crude product was dissolved in *n*-pentane and filtered through the syringe filter (0.2 μm) and excess of diyne or $B_2pin_2$ was removed after the filtration step by heating at 70-110 °C under vacuum ($10^{-3}$ mbar). For **3d-e**, the crude products were heated (approx. 90-150 °C) and condensed at cold-finger trap under vacuum (<$10^{-3}$ mbar). For, **4d-e** the products were filtered *via* thick (approx. 3 cm) pad of Florisil and analyzed.

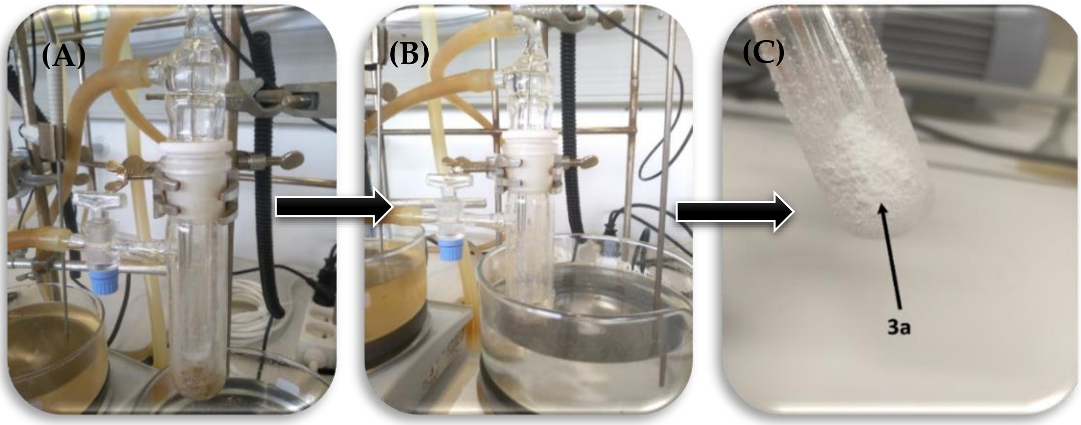


**Scheme S1**. Isolation of bisboryl-functionalized enynes. (**A**) – Crude product after filtration and solvent evaporation. (**B**) – Purification setup under reduced pressure and elevated temperature. (**C**) – condensed product (**3a**) on a cold finger trap.

## 3. General procedures for starting materials and product 3a functionalization

*3.1. Synthesis of alkynyl bromides*

The alkynyl bromides were prepared according to the literature with some modification[2]:

To a solution of alkyne (5 mmol) in acetone (50 mL), N-bromosuccinimide (6 mmol) and silver nitrate (0.5 mmol) were successively added. The reaction mixture was stirred without light access at room temperature over 18 h before adding water (100 mL). The resulting mixture was extracted with hexanes (3 x 100 mL) and the combined organic layers were washed with brine (100 mL), dried over $MgSO_4$, filtered through a pad of silica and concentrated to give a colourless or slightly yellow liquids.

**Caution:** All synthesized alkynyl bromides are strong lachrymators. The isolation should be performed under the hood.

*3.2 Suzuki coupling (for products **6**-**7**)*

[Pd($PPh_3$)$_4$] (0.005 mmol), **3a** (0.1 mmol), aryl iodide (0.24 mmol) (for **6**) or alkenyl iodide (0.12 mmol) (for **7a**-**b**) were placed in the Schlenk vessel and evacuated. Then the toluene (1 mL) and aqueous solution of $Cs_2CO_3$ (3M, 1mL) were added under argon atmosphere and stirred for 18 h at 60 °C using oil bath as a heat source. Afterwards, crude reaction mixture was analyzed by GC-MS and $^1$H NMR analyzes and purified according to the procedure in 2.1.1. subsection.

*3.3 Hydrosilylation (for product **8**)*

To the one-neck round bottom flask, **3a** (0.1 mmol), toluene (0.1 mL), triethylsilane (0.12 mmol) were added and heated to 100 °C (oil bath). Subsequently, the $Pt_2(dvs)_3$ ($10^{-4}$ mmol) was added and the reaction mixture was stirred for 24 h. Afterwards, crude reaction mixture was analyzed by GC-MS and $^1$H NMR analyzes and purified according to the procedure in 2.1.2. subsection.

*3.4 Sila-Sonogashira (for product **9**)*

Pd($PPh_3$)$_4$] (0.005 mmol), CuI (0.05 mmol), **3a** (0.1 mmol) and aryl iodide (0.11 mmol) were placed in the Schlenk vessel and evacuated. Then the dry DMF (1 mL) was added under argon atmosphere and stirred for 18 h at 80 °C (oil bath). Afterwards, crude reaction mixture was analyzed by GC-MS and $^1$H NMR analyzes and purified according to the procedure in 2.1.1. subsection.

*3.5 Mechanistic studies*

To the Young's NMR tube the **1** (15,8 mg, 0.0625 mmol) and Pt($PPh_3$)$_4$ (7,8mg, 0.00625 mmol) were added and kept under the vacuum for 1 h. After that, the 0.7 mL of dry toluene-d8 was added and heated over 1.5 h at 100 °C (oil bath) under Ar atmosphere. The mixture was cooled down to -50 °C (NMR chiller) and low temperature $^{31}$P NMR was performed.

**4. Starting materials and diynes characterization**

*(Bromoethynyl)benzene*

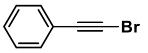


**$^1$H NMR** (300 MHz, $CDCl_3$, δ, ppm): 7.54 – 7.43 (m, 2H, Ph), 7.40 – 7.28 (m, 3H, Ph). **$^{13}$C{$^1$H} NMR** (75 MHz, $CDCl_3$, δ, ppm): 132.1, 128.8, 128.4, 122.7, 80.2 (C≡C-Br), 49.9 (C≡C-Br). **MS** (EI, m/z): 182 ($M^+$+2, 100), 180($M^+$, 97), 101(93), 75(48), 62(5), 51(8). Pale yellow liquid. Isolated yield: 91% (0.82 g). Analytical data are in agreement with the literature.[2]

*1-Bromooct-1-yne*

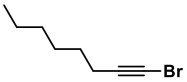


**$^1$H NMR** (300 MHz, $CDCl_3$, δ, ppm): 2.20 (t, $J_{H-H}$ = 7.0 Hz, 2H, $CH_2$C≡C), 1.51 (p, $J_{H-H}$ = 6.8 Hz, 2H), 1.38 – 1.24 (m, 6H), 0.89 (t, $J_{H-H}$ = 6.8 Hz, 3H, $CH_2CH_3$). **MS** (EI, m/z): 161($M^+$-28 ($C_2H_5$•), 4), 159($M^+$-30($C_2H_5$•), 4), 147(4), 145(4), 132(7), 119(8), 117(9), 109(15), 79(41), 67(100). Colorless liquid. Isolated yield: 93% (0.88 g). Analytical data are in agreement with the literature.[2]

*(Bromoethynyl)triisopropylsilane*

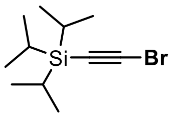

**$^1$H NMR** (300 MHz, $CDCl_3$, δ, ppm): 1.08 (s, 21H, $(Si(C\underline{H}(C\underline{H}_3)_2)_3)$. **$^{13}C\{^1H\}$ NMR** (75 MHz, $CDCl_3$, δ, ppm): 83.6 ($\underline{C}$≡C-Br), 61.9 (C≡$\underline{C}$-Br), 18.6 $(Si(\underline{C}H(CH_3)_2)_3)$, 11.4 $(Si(CH(\underline{C}H_3)_2)_3)$. **MS** (EI, m/z): 262 ($M^+$+2, 5) 260 ($M^+$, 5), 219(83), 217(83), 191(43), 189(42), 163(74), 161(68), 149(100), 147(93), 137(22), 109(31), 95(23), 69(17), 53(22). Colorless liquid. Isolated yield: 91% (1.18 g). Analytical data are in agreement with the literature.[3]

*1,4-Bis(triethylsilyl)buta-1,3-diyne* (**2b**)

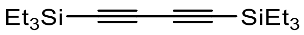


**$^1$H NMR** (300 MHz, $CDCl_3$, δ, ppm): . 1.00 (t, $J_{H\text{-}H}$ = 7.9 Hz, 18H, $Si(CH_2C\underline{H}_3)_3$), 0.64 (t, $J_{H\text{-}H}$ = 7.9 Hz, 12H, $Si(C\underline{H}_2CH_3)_3$). **$^{13}C\{^1H\}$ NMR** (101 MHz, $CDCl_3$, δ, ppm): 89.4 (C≡C), 83.3 (C≡C), 7.5, 4.3. **$^{29}$Si NMR** (79 MHz, $CDCl_3$ δ, ppm): -5.84. **MS** (EI, m/z): 278($M^+$, 7), 249(100), 221(94), 193(38), 165(27), 137(20), 109(11), 82(17), 68(8). **FT-IR** ($cm^{-1}$): 2955, 2936, 2912, 2875, 2064, 1457, 1004, 723, 695, 620. Colorless liquid. Isolated yield: 90% (1.24 g). Analytical data are in agreement with the literature.[4]

*1,4-Bis(triisopropylsilyl)buta-1,3-diyne* (**2c**)

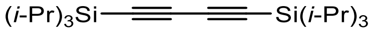


**$^1$H NMR** (300 MHz, $CDCl_3$, δ, ppm): 1.09 (s, 42H, $Si(C\underline{H}(C\underline{H}_3)_2)_3$). **$^{13}C\{^1H\}$ NMR** (101 MHz, $CDCl_3$, δ, ppm): 90.3 (C≡C), 81.7 (C≡C), 18.7, 11.5. **$^{29}$Si NMR** (79 MHz, $CDCl_3$ δ, ppm): -0.86. **MS** (EI, m/z): 362($M^+$, 10), 319(100), 291(23), 277(26), 263(15), 249(29), 235(15), 207(11). 193(13), 179(11), 165(14), 151(12), 137(16), 82(31), 59(17). **FT-IR** ($cm^{-1}$): 3055, 3031, 2112, 1587, 1477, 1436, 825, 755, 719, 691. White solid. Isolated yield: 81% (1.46 g). Analytical data are in agreement with the literature.[4]

*Hexadeca-7,9-diyne* (**2e**)

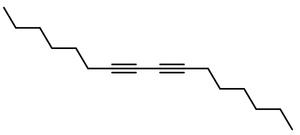

**$^1$H NMR** (300 MHz, $CDCl_3$, δ, ppm): 2.24 (t, $J_{H\text{-}H}$ = 6.9 Hz, 4H, $C\underline{H}_2C≡CC≡CC\underline{H}_2$), 1.57 – 1.45 (m, 4H), 1.40 – 1.22 (m, 12H), 0.88 (t, $J_{H\text{-}H}$ = 6.8 Hz, 6H, $CH_2C\underline{H}_3$). **$^{13}C\{^1H\}$ NMR** (101 MHz, $CDCl_3$, δ, ppm): 77.7 (C≡C), 65.4 (C≡C), 31.4, 28.7, 28.5, 22.7, 19.3, 14.2. **MS** (EI, m/z): 189($M^+$-29, 10), 161(4), 147(11), 133(22), 119(39), 105(58), 91(100), 79(65), 67(54), 55(30). **FT-IR** ($cm^{-1}$): 2956, 2928, 2858, 1466, 1426, 1323, 725. Colorless oil. Isolated yield: 90% (0.98 g). Analytical data are in agreement with the literature.[5]

*2,2,7,7-Tetramethylocta-3,5-diyne* (**2f**)

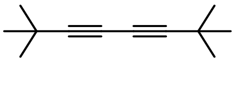

**$^1$H NMR** (300 MHz, $CDCl_3$, δ, ppm): 1.23 (s, 18H, $C\underline{H}_3)_3)_2$). **$^{13}C\{^1H\}$ NMR** (101 MHz, $CDCl_3$, δ, ppm): 86.4 (C≡C), 63.8 (C≡C), 30.8, 28.1. **MS** (EI, m/z): 162($M^+$, 74), 147(50), 132(10), 119(100), 105(92), 91(89), 77(36), 55(24). **FT-IR** ($cm^{-1}$): 2968, 2864, 2141, 1465, 1361, 1239, 1198, 677. White solid. Isolated yield: 87% (0.7 g). Analytical data are in agreement with the literature.[6]

*1,4-Bis(4-fluorophenyl)buta-1,3-diyne* (**2h**)

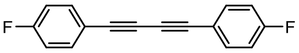

**$^{1}$H NMR** (300 MHz, $CDCl_3$, δ, ppm): 7.58 – 7.43 (m, 4H, Ph), 7.11 – 6.96 (m, 4H, Ph). **$^{13}$C{$^{1}$H} NMR** (101 MHz, $CDCl_3$, δ, ppm): 163.2 (d, $J^{1}_{C\text{-}F}$ = 251.6 Hz), 134.7 (d, $J^{3}_{C\text{-}F}$ = 8.6 Hz), 117.9 (d, $J^{4}_{C\text{-}F}$ = 3.6 Hz), 116.1 (d, $J^{2}_{C\text{-}F}$ = 22.3 Hz), 80.6 (C≡C), 73.7 (C≡C). **MS** (EI, m/z): 238($M^+$, 100), 168(6), 119(14). **FT-IR** ($cm^{-1}$): 1887, 1594, 1499, 1215, 1157, 1093, 825, 695, 524. White solid. Isolated yield: 83% (0.98 g). Analytical data are in agreement with the literature.[7]

*1,4-Bis(4-(trifluoromethyl)phenyl)buta-1,3-diyne* (**2i**)

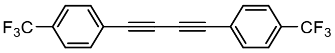

**$^{1}$H NMR** (300 MHz, $CDCl_3$, δ, ppm): 7.70 – 7.55 (m, 8H, Ph). **$^{13}$C{$^{1}$H} NMR** (75 MHz, $CDCl_3$, δ, ppm): 132.7, 131.3 (q, $J^{2}_{C\text{-}F}$ = 32.9 Hz), 125.6 (q, $J^{3}_{C\text{-}F}$ = 3.7 Hz), 125.4, 122.0, 81.1, 75.8. **$^{29}$Si NMR** (79 MHz, $CDCl_3$ δ, ppm): 18.79. **MS** (EI, m/z): 338($M^+$, 100), 319(13), 288(9), 249(5), 199(4), 169(4), 144(5), 119(3). **FT-IR** ($cm^{-1}$): 1607, 1306, 1166, 1121, 1104, 1062, 835. White solid. Isolated yield: 91% (1.53 g). Analytical data are in agreement with the literature.[8]

*1,4-Bis(4-methoxyphenyl)buta-1,3-diyne* (**2j**)

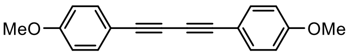

**$^{1}$H NMR** (300 MHz, $CDCl_3$, δ, ppm): 7.46 (d, $J_{H\text{-}H}$ = 8.8 Hz, 4H, Ph), 6.85 (d, $J_{H\text{-}H}$ = 8.9 Hz, 4H, Ph), 3.82 (s, 6H, OC$\underline{H}_3$).**$^{13}$C{$^{1}$H} NMR** (101 MHz, $CDCl_3$, δ, ppm): 160.4, 134.2, 114.3, 114.1, 81.4 (C≡C), 73.06 (C≡C), 55.47 (O$\underline{C}H_3$). **MS** (EI, m/z): 262($M^+$, 100), 247(43), 219(15), 203(7), 176(13), 150(5), 131(3). **FT-IR** ($cm^{-1}$): 3002, 2975, 2138, 1597, 1561, 1503, 1292, 1253, 1181, 1165, 1026, 840, 820. Pale yellow solid. Isolated yield: 88% (1.15 g). Analytical data are in agreement with the literature.[7]

*1,6-Bis(trimethylsiloxy)hexa-2,4-diyne* (**2l**)

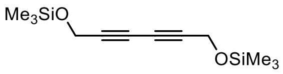

**$^{1}$H NMR** (300 MHz, $CDCl_3$, δ, ppm): 4.34 (s, 4H, CC$\underline{H}_2$O), 0.16 (s, 18H, OSi(C$\underline{H}_3$)$_3$ ).**$^{13}$C{$^{1}$H} NMR** (75 MHz, $CDCl_3$, δ, ppm): 77.5, 69.4, 51.5, -0.3. **$^{29}$Si NMR** (79 MHz, $CDCl_3$ δ, ppm): 22.26. **MS** (EI, m/z): 254($M^+$, 23), 239(7), 209(25), 179(73), 147(28), 73(100). **FT-IR** ($cm^{-1}$): 2958, 2856, 1362, 1250, 1078, 834, 748. Brown oil. Isolated yield: 77% (0.97 g). Despite previous report, **2l** is fully characterized for the first time.[9]

*(Phenylbuta-1,3-diyn-1-yl)trisopropylsilane* (**2m**)

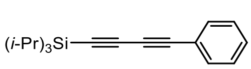

**$^{1}$H NMR** (300 MHz, $CDCl_3$, δ, ppm): 7.55 – 7.27 (m, 5H, Ph), 1.12 (s, 21H, Si(C$\underline{H}$(C$\underline{H}_3$)$_2$)$_3$). **$^{13}$C{$^{1}$H} NMR** (75 MHz, $CDCl_3$, δ, ppm): 132.8, 129.4, 128.5, 121.7, 89.6 (C≡C), 88.0 (C≡C), 75.7 (C≡C), 74.8 (C≡C), 18.7, 11.6. **$^{29}$Si NMR** (79 MHz, $CDCl_3$ δ, ppm): -0.65. **MS** (EI, m/z): 282($M^+$, 8), 239(98), 211(44), 197(40), 183(54), 169(100), 159(21), 153(27), 91(20) 59(10). **FT-IR** ($cm^{-1}$): 2942, 2890, 2865, 2204, 2101, 1488, 1461, 1070, 1018, 995, 881, 752, 729, 675, 602. Colorless oil. Isolated yield: 75% (1.05 g). Analytical data are in agreement with the literature.[10]

*((4-Bromophenyl)buta-1,3-diyn-1-yl)trisopropylsilane* (**2n**)

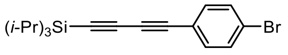

**$^1$H NMR** (300 MHz, $CDCl_3$, δ, ppm): 7.49 – 7.43 (m, 2H, Ph), 7.39 – 7.33 (m, 2H, Ph), 1.11 (s, 21H, $Si(C\underline{H}(C\underline{H}_3)_2)_3$). **$^{13}$C{$^1$H} NMR** (75 MHz, $CDCl_3$, δ, ppm): 134.2, 131.9, 123.9, 120.7, 89.4 (C≡C), 89.0 (C≡C), 75.9 (C≡C), 74.5 (C≡C), 18.7, 11.4. **$^{29}$Si NMR** (79 MHz, $CDCl_3$ δ, ppm): -0.46. **MS** (EI, m/z): 362($M^+$, 10), 360($M^+$-2, 12), 319(100), 317(98), 291(33), 289(33), 277(28), 275(26), 249(61), 247(61), 153(16), 151(17), 109(10), 59(8). **FT-IR** ($cm^{-1}$): 2942, 2890, 2864, 2102, 1485, 1462, 1070, 1009, 881, 820, 745, 676, 613. **Anal. Calcd for** $C_{19}H_{25}BrSi$: C, 63.15; H, 6.97. Found: C, 63.09; H, 6.93. White solid. Isolated yield: 74% (1.33 g). Analytical data are in agreement with the literature.[11]

*Trisopropyl((4-(trifluoromethyl)phenyl)buta-1,3-diyn-1-yl)silane* (**2o**)

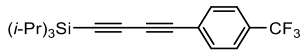


**$^1$H NMR** (300 MHz, $CDCl_3$, δ, ppm): 7.68 – 7.50 (m, 4H, Ph), 1.12 (s, 21H, $Si(C\underline{H}(C\underline{H}_3)_2)_3$). **$^{13}$C{$^1$H} NMR** (101 MHz, $CDCl_3$, δ, ppm): 133.1, 131.1, 130.8, 125.5 (q, $J^3{}_{C\text{-}F}$ = 3.8 Hz), 122.5, 89.9 (C≡C), 89.1 (C≡C) , 74.0 (C≡C), 18.7, 11.4. **$^{29}$Si NMR** (79 MHz, $CDCl_3$ δ, ppm): -0.28. **MS** (EI, m/z): 350($M^+$, 5), 307(100), 279(38), 265(26), 251(55), 237(82), 197(12), 175(17), 151(7), 137(8), 125(6). **FT-IR** ($cm^{-1}$): 2944, 2892, 2867, 2104, 1614, 1462, 1318, 1168, 1129, 1105, 1067, 1015, 881, 839, 676, 659, 613, 595. **Anal. Calcd for** $C_{20}H_{25}F_3Si$: C, 68.54; H, 7.19. Found: C, 68.58; H, 7.21. Colorless oil. Isolated yield: 81% (1.42 g). Analytical data are in agreement with the literature.[11]

*Triisopropyl(m-tolylbuta-1,3-diyn-1-yl)silane* (**2p**)

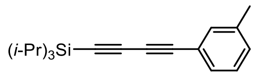


**$^1$H NMR** (400 MHz, $CDCl_3$, δ, ppm): 7.35 – 7.29 (m, 2H, Ph), 7.23 – 7.14 (m, 2H, Ph), 2.33 (s, 3H $PhC\underline{H}_3$), 1.12 (s, 21H, $Si(C\underline{H}(C\underline{H}_3)_2)_3$). **$^{13}$C{$^1$H} NMR** (101 MHz, $CDCl_3$, δ, ppm): 138.3, 133.4, 130.3, 129.9, 128.4, 121.5, 89.8 (C≡C), 87.7 (C≡C), 76.0 (C≡C), 74.5 (C≡C), 21.3 ($Ph\underline{C}H_3$), 18.7, 11.5. **$^{29}$Si NMR** (79 MHz, $CDCl_3$ δ, ppm): -0.70. **MS** (EI, m/z): 296($M^+$, 12), 253(98), 225(44), 211(41), 197(50), 183(100), 167(24), 143(24), 98(19), 59(10). **Anal. Calcd for** $C_{20}H_{28}Si$: C, 81.01; H, 9.52. Found: C, 81.19; H, 9.60. Yellow oil. Isolated yield: 77% (1.14 g).

*((4,6-Dihydropyren-1-yl)buta-1,3-diyn-1-yl)triisopropylsilane* (**2q**)

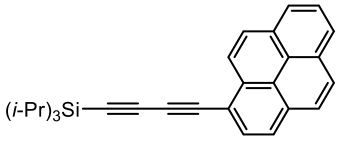


**$^1$H NMR** (300 MHz, $CDCl_3$, δ, ppm): 8.58 (d, $J_{H\text{-}H}$ = 9.1 Hz, 1H), 8.28 – 7.99 (m, 8H), 1.18 (s, 21H, $Si(C\underline{H}(C\underline{H}_3)_2)_3$). **$^{13}$C{$^1$H} NMR** (101 MHz, $CDCl_3$, δ, ppm): 133.7, 132.0, 131.3, 131.2, 131.0, 129.0, 128.9, 127.3, 126.5, 126.1, 126.0, 125.5, 124.6, 124.5, 124.3, 116.0, 90.0 (C≡C), 89.7 (C≡C), 80.2 (C≡C), 75.1 (C≡C), 18.8, 11.6. **$^{29}$Si NMR** (79 MHz, $CDCl_3$ δ, ppm): -0.57. **FT-IR** ($cm^{-1}$): 2940, 2889, 2863, 2191, 2089, 1459, 1069, 1016, 993, 880, 844, 670. **Anal. Calcd for** $C_{29}H_{32}Si$: C, 85.23; H, 7.89. Found: C, 84.98; H, 7.77. Yellow solid. Isolated yield: 48% (0.97g). Analytical data are in agreement with the literature.[12]

*(Cyclopropylbuta-1,3-diyn-1-yl)triisopropylsilane* (**2r**)

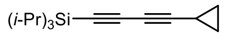


**$^1$H NMR** (300 MHz, $CDCl_3$, δ, ppm): 1.37 – 1.28 (m, 1H, $C\underline{H}$), 1.07 (s, 21H, $Si(C\underline{H}(C\underline{H}_3)_2)_3$), 0.85 – 0.78 (m, 4H, $CH_2CH_2$).**$^{13}$C{$^1$H} NMR** (75 MHz, $CDCl_3$, δ, ppm): 90.4 (C≡C), 81.9 (C≡C), 79.7 (C≡C), 61.5 (C≡C), 18.7, 11.5, 8.9, 0.2. **$^{29}$Si NMR** (79 MHz, $CDCl_3$ δ, ppm): -1.15. **MS** (EI, m/z): 246($M^+$, 4), 203(93), 175(42), 161(50), 147(48), 133(100), 118(17), 93(27), 59(23). **Anal. Calcd for** $C_{20}H_{28}Si$: C, 77.97; H, 10.63. Found: C, 78.08; H, 10.72. Yellow oil. Isolated yield: 83% (1.02 g).

*(5-Phenylpenta-1,3-diyn-1-yl)triisopropylsilane* (**2s**)

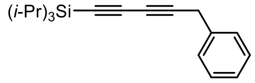

**$^1$H NMR** (300 MHz, $CDCl_3$, δ, ppm): 6.75 – 6.58 (m, 5H), 3.09 (s, 2H, ≡$C\underline{H}_2$Ph), 1.11 (s, 21H, Si($C\underline{H}(C\underline{H}_3)_2)_3$). **$^{13}C\{^1H\}$ NMR** (75 MHz, $CDCl_3$, δ, ppm): 135.4, 128.8, 128.2, 127.1, 90.0 (C≡C), 81.5 (C≡C), 75.8 (C≡C), 67.9 (C≡C), 25.8, 18.7, 11.4. **$^{29}$Si NMR** (79 MHz, $CDCl_3$ δ, ppm): -0.93. **MS** (EI, m/z): 296($M^+$, 4), 253(100), 225(30), 211(27), 197(29), 183(58), 155(14), 115(7), 98(5), 59(11). **FT-IR** ($cm^{-1}$): 2942, 2890, 2865, 2225, 2105, 1495, 1455, 1188, 1073, 995, 882, 727, 675, 611. **Anal. Calcd for** $C_{20}H_{28}Si$: C, 81.01; H, 9.52. Found: C, 80.98; H, 9.53. Yellow oil. Isolated yield: 73% (1.08 g).

*(5-Phenoxypenta-1,3-diyn-1-yl)trisopropylsilane* (**2t**)

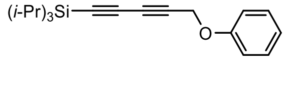

**$^1$H NMR** (300 MHz, $CDCl_3$, δ, ppm): 7.42 – 7.31 (m, 2H, Ph), 7.08 – 6.96 (m, 3H, Ph) 4.80 (s, 2H, ≡$C\underline{H}_2$OPh), 1.11 (s, 21H, Si($C\underline{H}(C\underline{H}_3)_2)_3$). **$^{13}C\{^1H\}$ NMR** (75 MHz, $CDCl_3$, δ, ppm): 157.7, 129.7, 125.3, 121.8, 115.0, 88.8 (C≡C), 85.5 (C≡C), 72.5 (C≡C), 71.5 (C≡C), 56.4, 18.6, 11.4. **$^{29}$Si NMR** (79 MHz, $CDCl_3$ δ, ppm): -0.45. **MS** (EI, m/z): 312($M^+$, 26), 269(100), 241(50), 225(29), 213(24), 199(38), 185(20), 173(30), 151(81), 137(32), 121(22), 106(34), 92(19), 59(25). **FT-IR** ($cm^{-1}$): 2943, 2891, 2865, 2225, 2106, 1598, 1588, 1494, 1461, 1211, 1172, 1032, 1015, 994, 881, 801, 750, 676. **Anal. Calcd for** $C_{20}H_{28}OSi$: C, 76.86; H, 9.03. Found: C, 76.88; H, 9.01. Yellow oil. Isolated yield: 69% (1.07 g).

*Deca-1,3-diyn-1-yltrisopropylsilane* (**2u**)

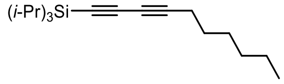

**$^1$H NMR** (300 MHz, $CDCl_3$, δ, ppm): 2.28 (d, $J_{H\text{-}H}$ = 7.0 Hz, 2H, ≡$C\underline{H}_2$), 1.59 – 1.18 (m, 8H), 1.08 (s, 21H, Si($C\underline{H}(C\underline{H}_3)_2)_3$), 0.89 (t, $J_{H\text{-}H}$ = 6.8 Hz, 3H, $CH_2C\underline{H}_3$). **$^{13}C\{^1H\}$ NMR** (75 MHz, $CDCl_3$, δ, ppm): 90.3 (C≡C), 80.1 (C≡C), 79.1 (C≡C), 65.9 (C≡C), 31.4, 28.6, 28.3, 22.6, 19.4, 18.7, 14.2, 11.4. **$^{29}$Si NMR** (79 MHz, $CDCl_3$ δ, ppm): -1.14. **MS** (EI, m/z): 290($M^+$, 3), 247(100), 219(38), 205(33), 191(28), 177(52), 163(5), 149(9), 137(11), 109(13), 95(10), 83(15), 59(20). **FT-IR** ($cm^{-1}$): 2941, 2865, 2223, 2104, 1462, 1181, 995, 881, 675. **Anal. Calcd for** $C_{19}H_{34}Si$: C, 78.54; H, 11.79. Found: C, 78.50; H, 11.81. Pale yellow oil. Isolated yield: 82% (1.19g). Analytical data are in agreement with the literature.[13]

*Trimethyl(phenylbuta-1,3-diyn-1-yl)silane* (**2v**)

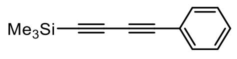

**$^1$H NMR** (300 MHz, $CDCl_3$, δ, ppm): 7.62 – 7.43 (m, 2H, Ph), 7.43 – 7.18 (m, 3H, Ph), 0.24 (s, 9H, Si($C\underline{H}_3)_3$). **$^{13}C\{^1H\}$ NMR** (75 MHz, $CDCl_3$, δ, ppm): 132.8, 129.5, 128.6, 121.5, 90.8 (C≡C), 88.0 (C≡C), 76.9 (C≡C), 74.3 (C≡C), -0.2 (Si($\underline{C}H_3)_3$). **$^{29}$Si NMR** (79 MHz, $CDCl_3$ δ, ppm): -16.1. **MS** (EI, m/z): 198($M^+$, 26), 183(100), 167(3), 153(5), 129(8). **FT-IR** ($cm^{-1}$): 2959, 2205, 2104, 1489, 1442, 1250, 837, 751, 686, 632. Pale yellow oil. Isolated yield: 66% (0.65 g). Analytical data are in agreement with the literature.[14]

*2,2-Dimethyldodeca-3,5-diyne* (**2w**)

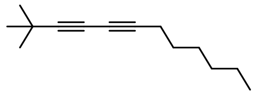

$^{1}$**H NMR** (300 MHz, $CDCl_3$, δ, ppm): 2.25 (d, $J_{H-H}$ = 7.0 Hz, 2H, ≡CC$\underline{H}_2$), 1.59 – 1.24 (m, 8H), 1.23 (s, 9H, C($\underline{H}_3$)$_3$), 0.88 (t, $J_{H-H}$ = 6.8 Hz, 3H, $CH_2C\underline{H}_3$). $^{13}$**C{$^{1}$H} NMR** (75 MHz, $CDCl_3$, δ, ppm): 85.2 (C≡C), 79.0 (C≡C), 65.1 (C≡C), 64.0 (C≡C), 31.5, 30.8, 28.7, 28.5, 28.1, 22.7, 19.4, 14.2. **MS** (EI, m/z): 175(M$^{+}$-15, 20), 161(20), 147(19), 133(), 119(70), 105(100), 91(67), 79(38), 67(22), 55(25). **FT-IR** (cm$^{-1}$): 2966, 2930, 2861, 1706, 1456, 1363, 1281, 1201, 1169. **Anal. Calcd for** $C_{14}H_{22}$: C, 88.35; H, 11.65. Found: C, 88.38; H, 11.62. Yellow oil. Isolated yield: 89% (0.84 g) Despite previous report, **2v** is fully characterized for the first time.[15]

*Deca-1,3-diyn-1-ylbenzene* (**2x**)

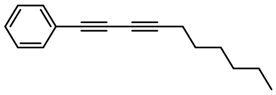

$^{1}$**H NMR** (300 MHz, $CDCl_3$, δ, ppm): 7.51 – 7.27 (m, 5H, Ph), 2.36 (d, $J_{H-H}$ = 7.0 Hz, 2H, ≡CC$\underline{H}_2$), 1.65 – 1.25 (m, 8H), 0.91 (t, $J_{H-H}$ = 6.7 Hz, 3H, $CH_2C\underline{H}_3$). $^{13}$**C{$^{1}$H} NMR** (75 MHz, $CDCl_3$, δ, ppm): 132.6, 128.9, 128.5, 122.3, 85.1 (C≡C), 74.8 (C≡C), 74.6 (C≡C), 65.2 (C≡C), 31.4, 28.7, 28.4, 22.7, 19.7, 14.2. **MS** (EI, m/z): 210(M$^{+}$, 53), 195(16), 181(72), 165(76), 153(53), 139(100), 126(39), 115(39), 91(22), 79(14), 63(8). **FT-IR** (cm$^{-1}$): 2955, 2929, 2858, 1489, 1422, 752, 687. Yellow oil. Isolated yield: 88% (0.92 g). Analytical data are in agreement with the literature.[16]

*1-(Deca-1,3-diyn-1-yl)-2-methylbenzene* (**2y**)

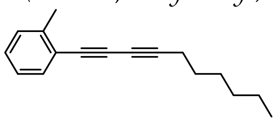

$^{1}$**H NMR** (300 MHz, $CDCl_3$, δ, ppm): 7.37 (dd, $J_{H-H}$ = 7.6, 1.4 Hz, 1H, Ph), 7.20 – 6.99 (m, 3H, Ph), 2.37 (s, 3H, PhC$\underline{H}_3$), 2.30 (d, $J_{H-H}$ = 7.0 Hz, 2H, ≡CC$\underline{H}_2$), 1.58 – 1.41 (m, 2H), 1.39 – 1.12 (m, 6H) 0.83 (t, $J_{H-H}$ = 6.7 Hz, 3H, $CH_2C\underline{H}_3$). $^{13}$**C{$^{1}$H} NMR** (75 MHz, $CDCl_3$, δ, ppm): 141.7, 133.1, 129.6, 128.7, 125.7, 122.1, 85.5 (C≡C), 78.1 (C≡C), 73.9 (C≡C), 65.3 (C≡C), 31.5, 28.7, 28.4, 22.7, 20.8, 19.8, 14.2. **MS** (EI, m/z): 224(M$^{+}$, 78), 209(14), 195(62), 181(26), 165(83), 152(100), 139(30), 129(23), 115(39), 105(7), 91(15), 79(18), 67(24) 51(7). **FT-IR** (cm$^{-1}$): 2954, 2929, 2857, 1484, 1456, 753, 713. **Anal. Calcd for** $C_{17}H_{20}$: C, 91.01; H, 8.99. Found: C, 90.99; H, 9.01. Pale-orange oil. Isolated yield: 85% (0.95 g).

*2-(Deca-1,3-diyn-1-yl)-1,3-dimethylbenzene* (**2z**)

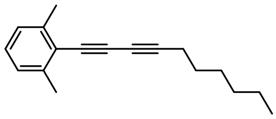

$^{1}$**H NMR** (600 MHz, $CDCl_3$, δ, ppm): 7.14 – 7.09 (m, 1H, Ph), 7.03 (m, 2H, Ph), 2.45 (s, 6H, PhC$\underline{H}_3$), 2.39 (t, $J_{H-H}$ = 7.2 Hz, 2H), 1.60 (p, $J_{H-H}$ = 7.2 Hz, 2H), 1.48 – 1.41 (m, 2H), 1.37 – 1.29 (m, 4H), 0.92 (t, $J_{H-H}$ = 7.0 Hz, 3H). $^{13}$**C{$^{1}$H} NMR** (151 MHz, $CDCl_3$, δ, ppm): 142.0, 128.2, 126.8, 122.1, 86.1 (C≡C), 82.3 (C≡C), 72.7 (C≡C), 65.3 (C≡C), 31.5, 28.8, 28.4, 27.4, 22.7, 21.2, 19. 9, 14.2. **MS** (EI, m/z): 238(M$^{+}$, 100), 223(10), 209(39), 195(20), 180(27), 164(56), 152(39), 115(16), 91(15), 79(26). **FT-IR** (cm$^{-1}$): 2954, 2927, 2858, 1466, 1377, 769. **Anal. Calcd for** $C_{18}H_{22}$: C, 90.70; H, 9.30. Found: C, 91.04; H, 8.96. Colourless oil. Isolated yield: 88% (1.04 g).

## 4. NMR spectra

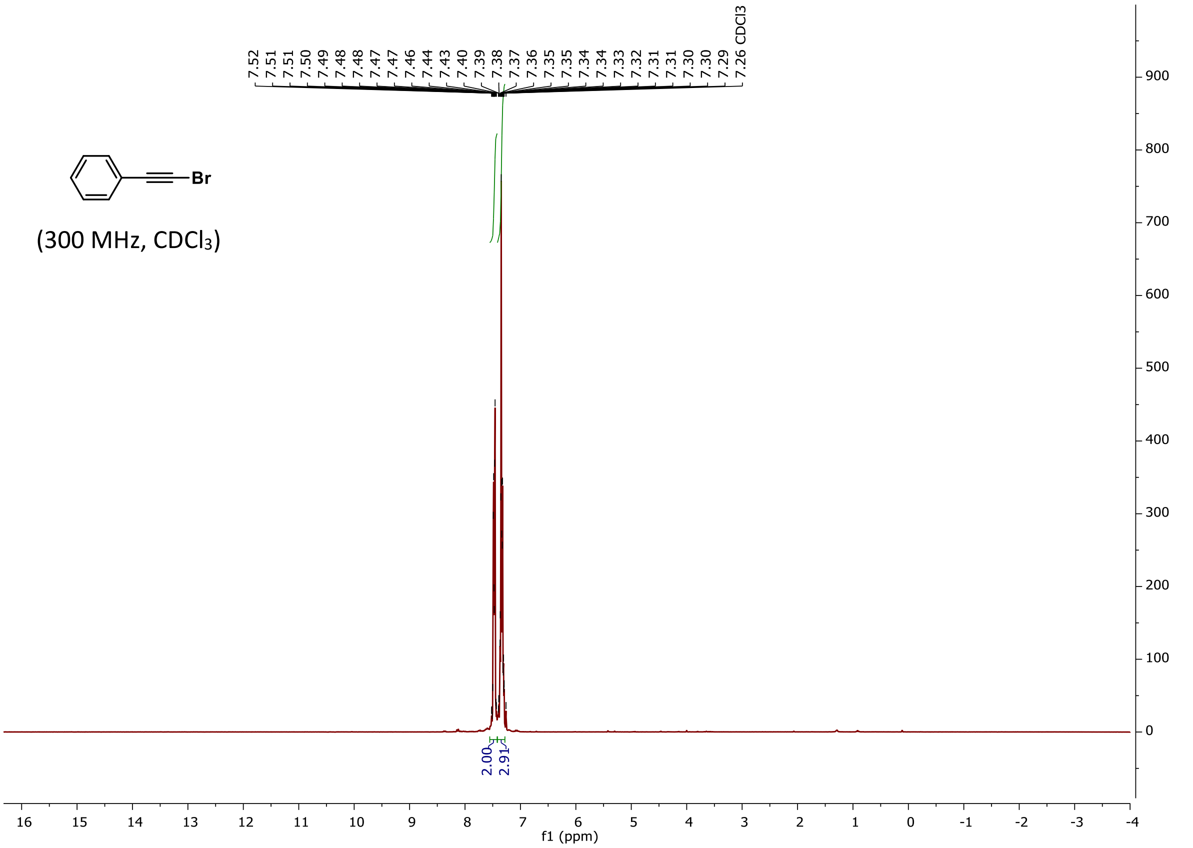


**Figure S1**. $^{1}H$ NMR spectrum of (bromoethynyl)benzene.

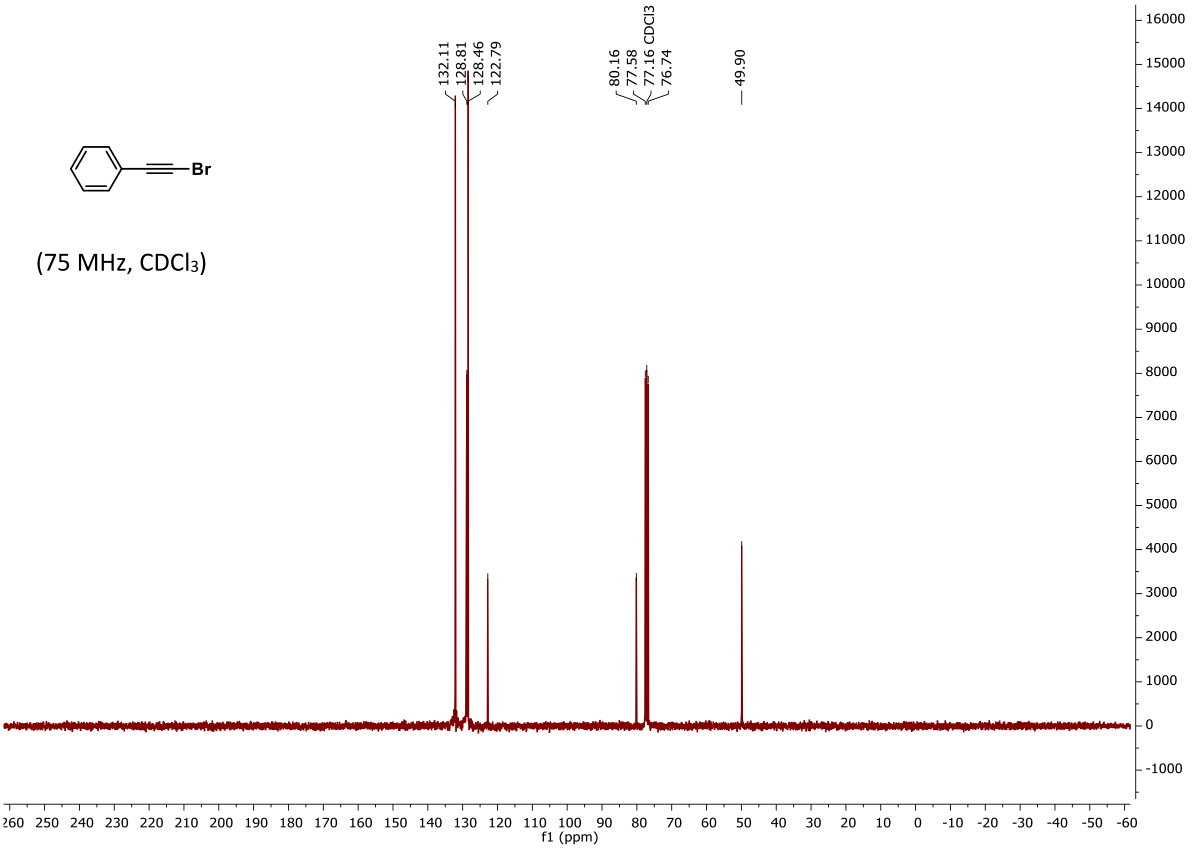


**Figure S2**. $^{13}C$ NMR spectrum of (bromoethynyl)benzene.

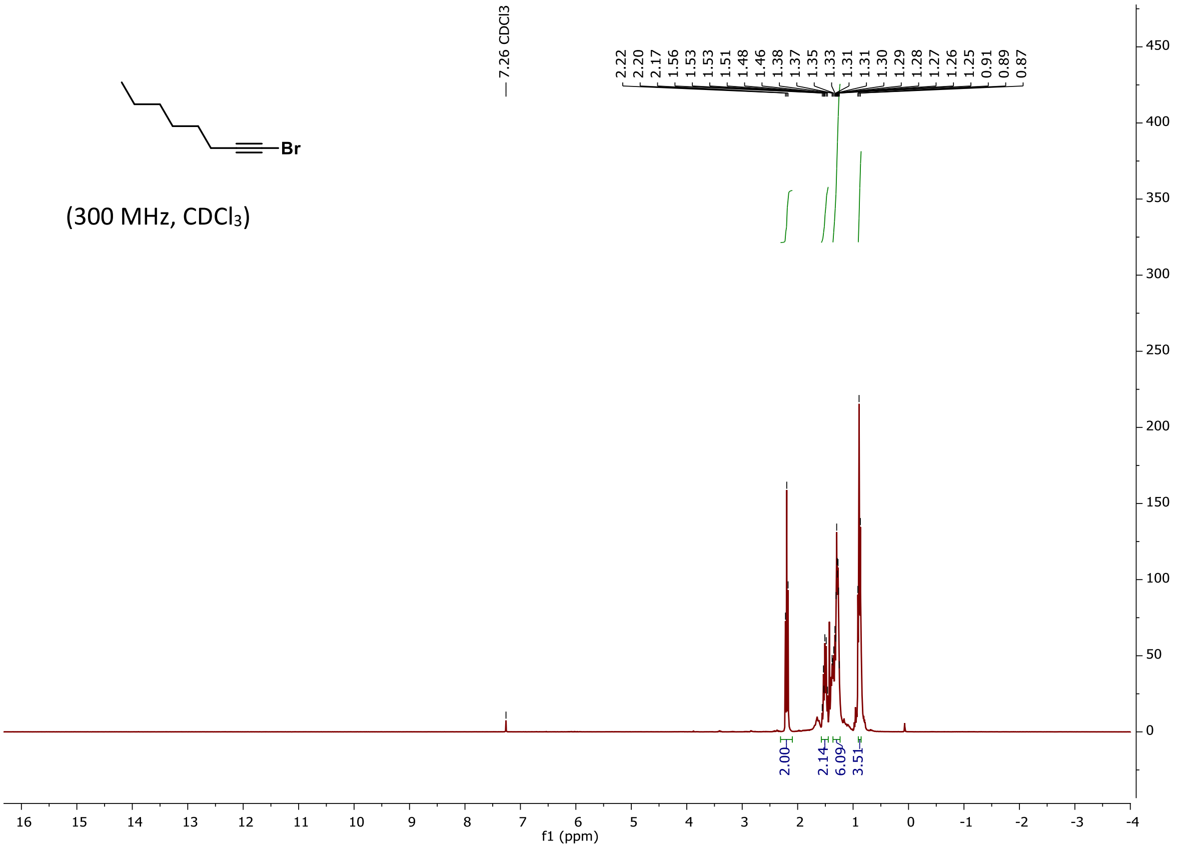


**Figure S3**. ¹H NMR spectrum of *1-bromooct-1-yne.*

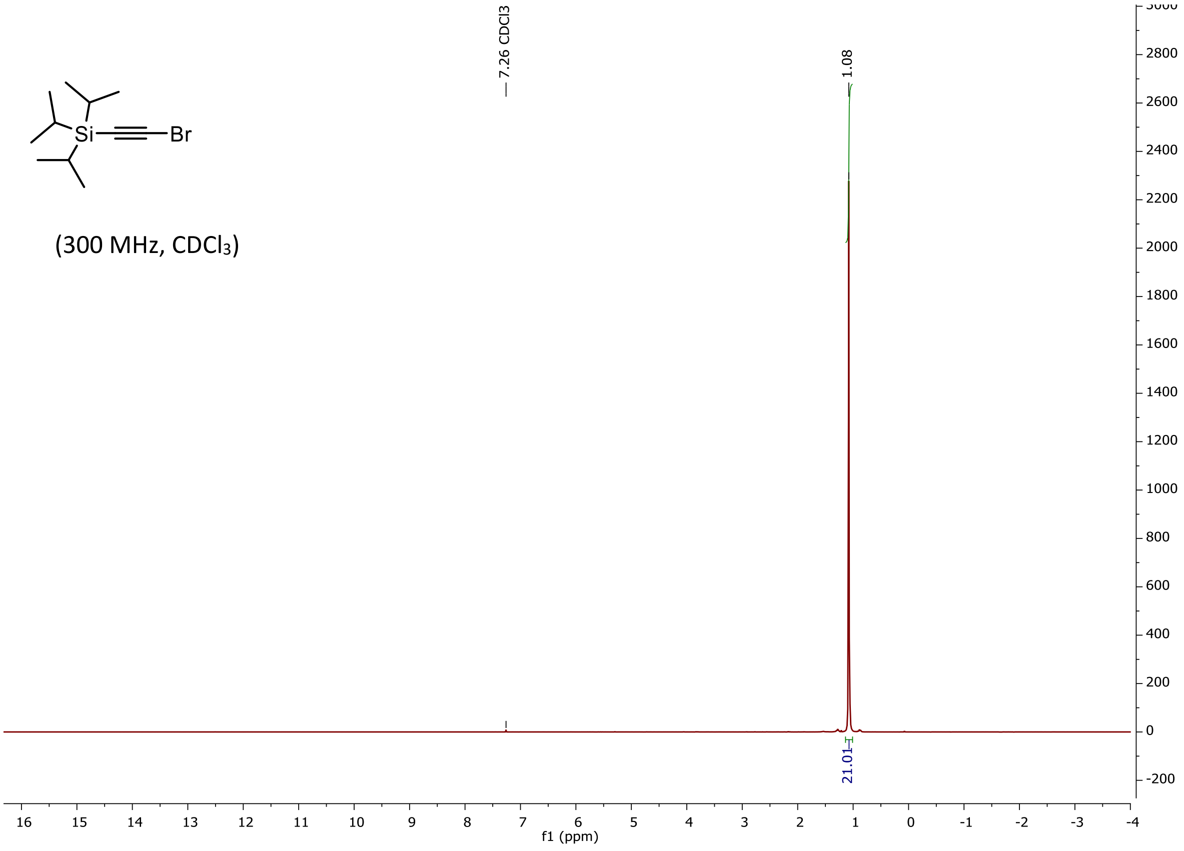


**Figure S4**. ¹H NMR spectrum of *(bromoethynyl)triisopropylsilane.*

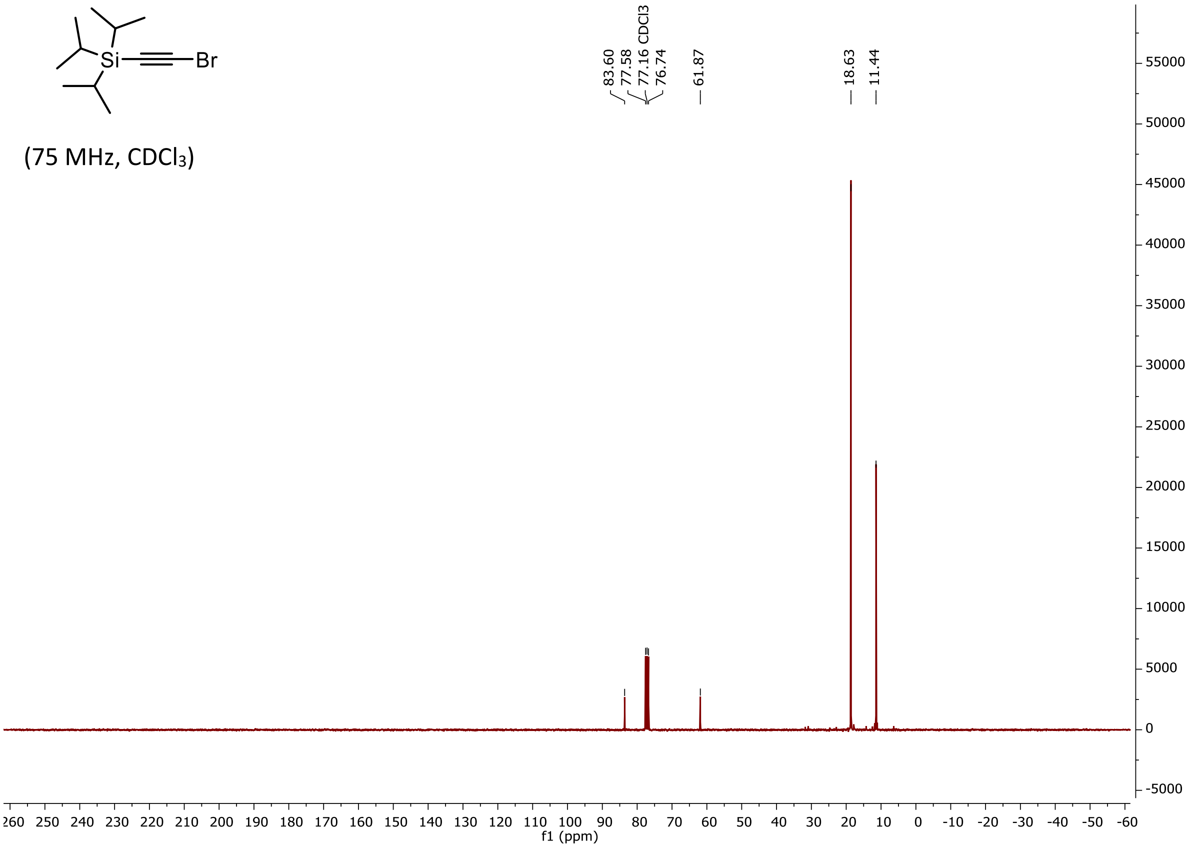


**Figure S5**. $^{13}$C NMR spectrum of *(bromoethynyl)triisopropylsilane.*

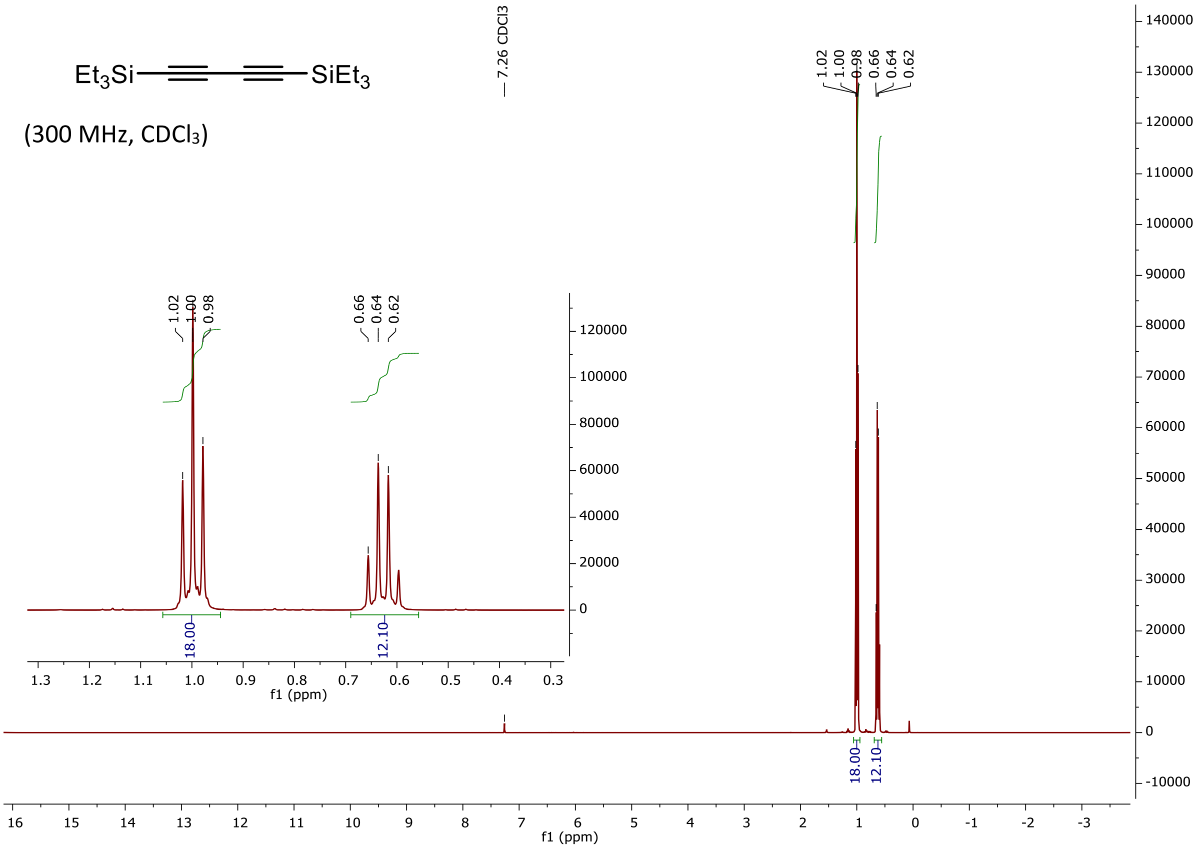


**Figure S6**. $^{1}$H NMR spectrum of **2b**.

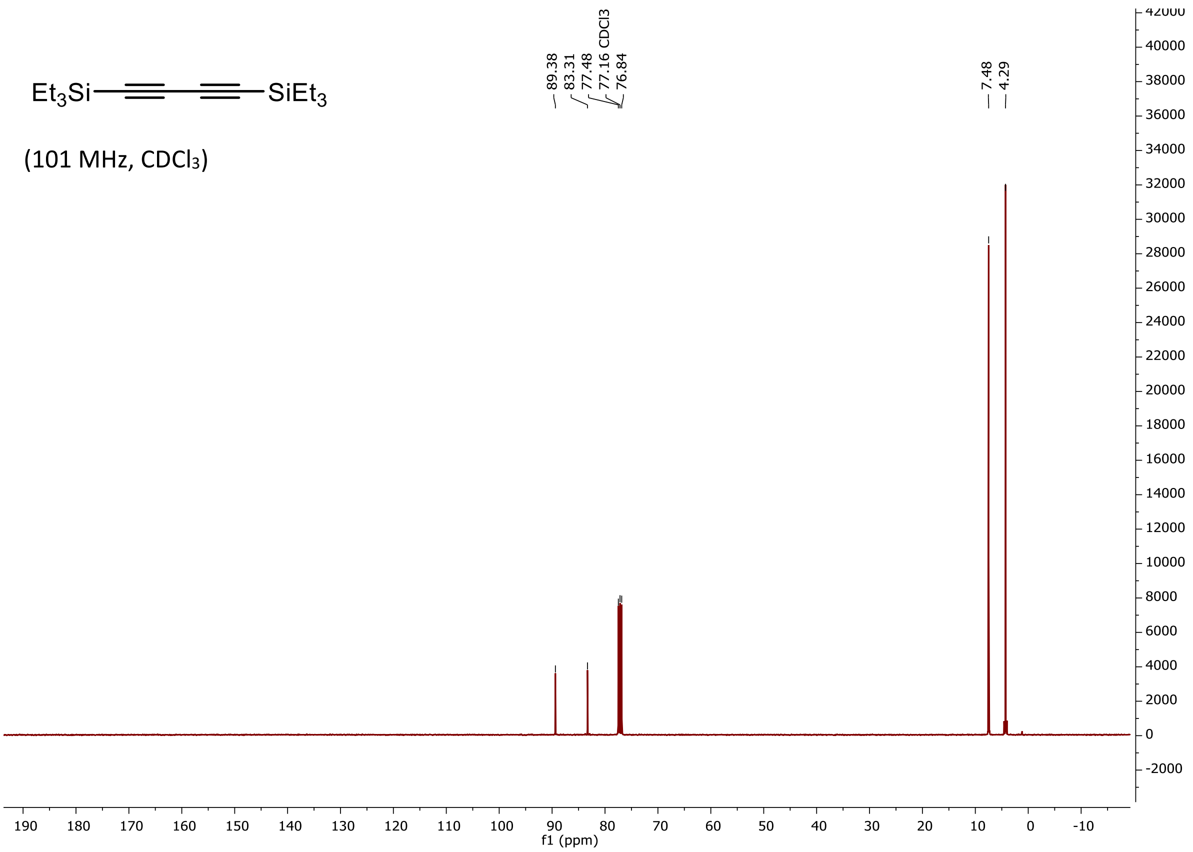


**Figure S7**. $^{13}$C NMR spectrum of **2b**.

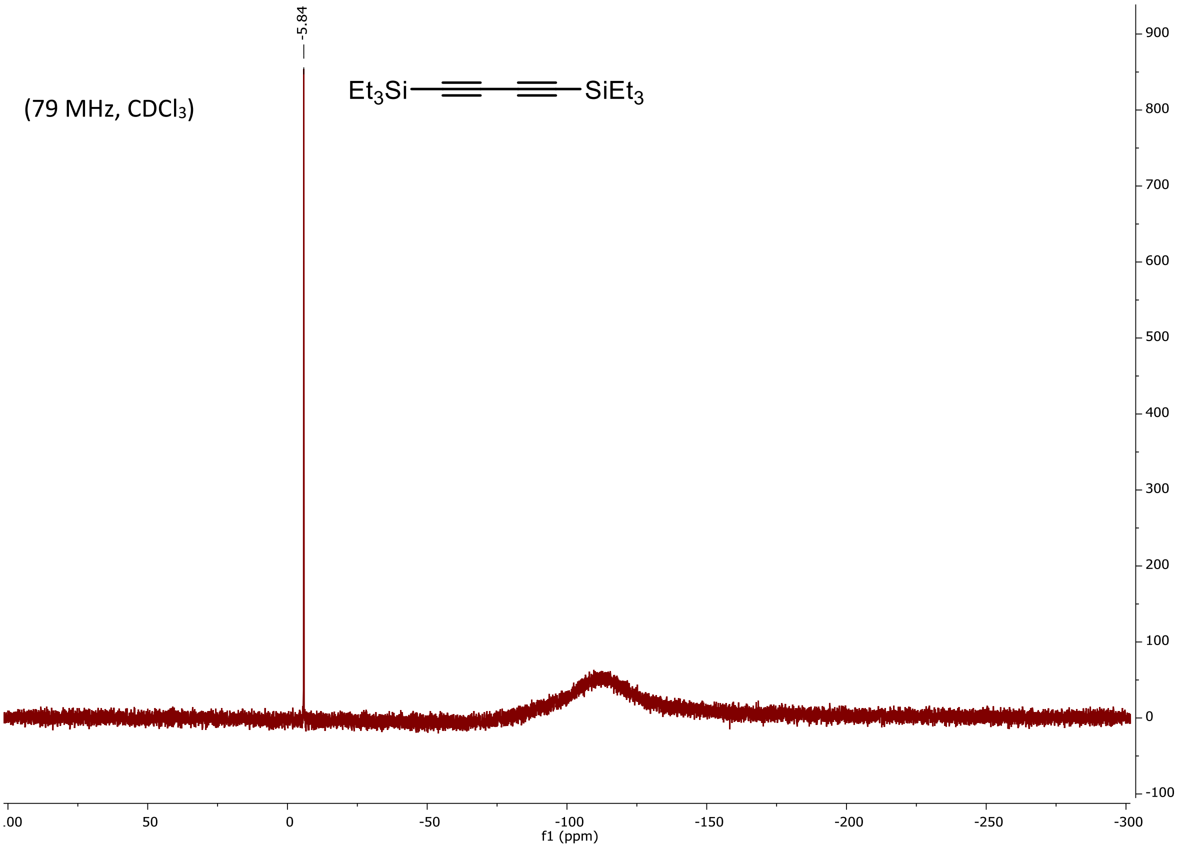


**Figure S8**. $^{29}$Si NMR spectrum of **2b**.

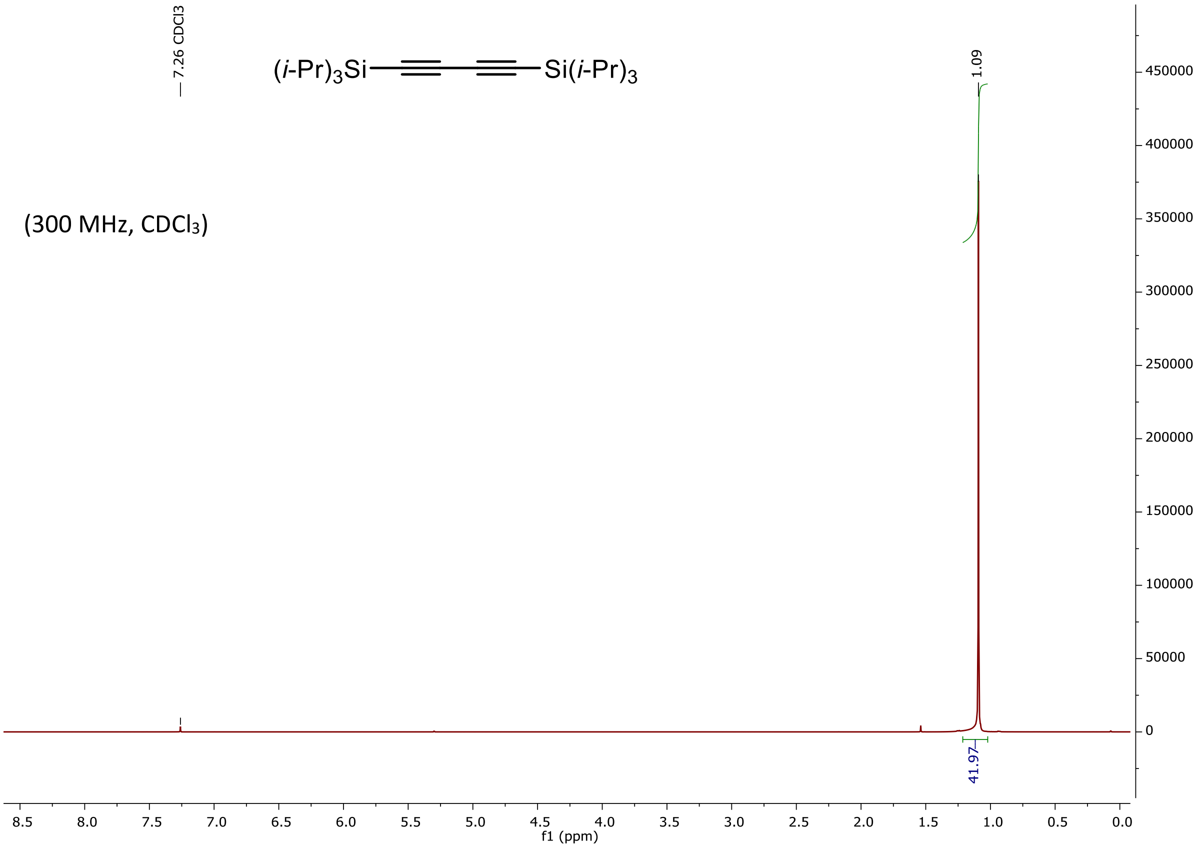


**Figure S9**. $^{1}$H NMR spectrum of **2c**.

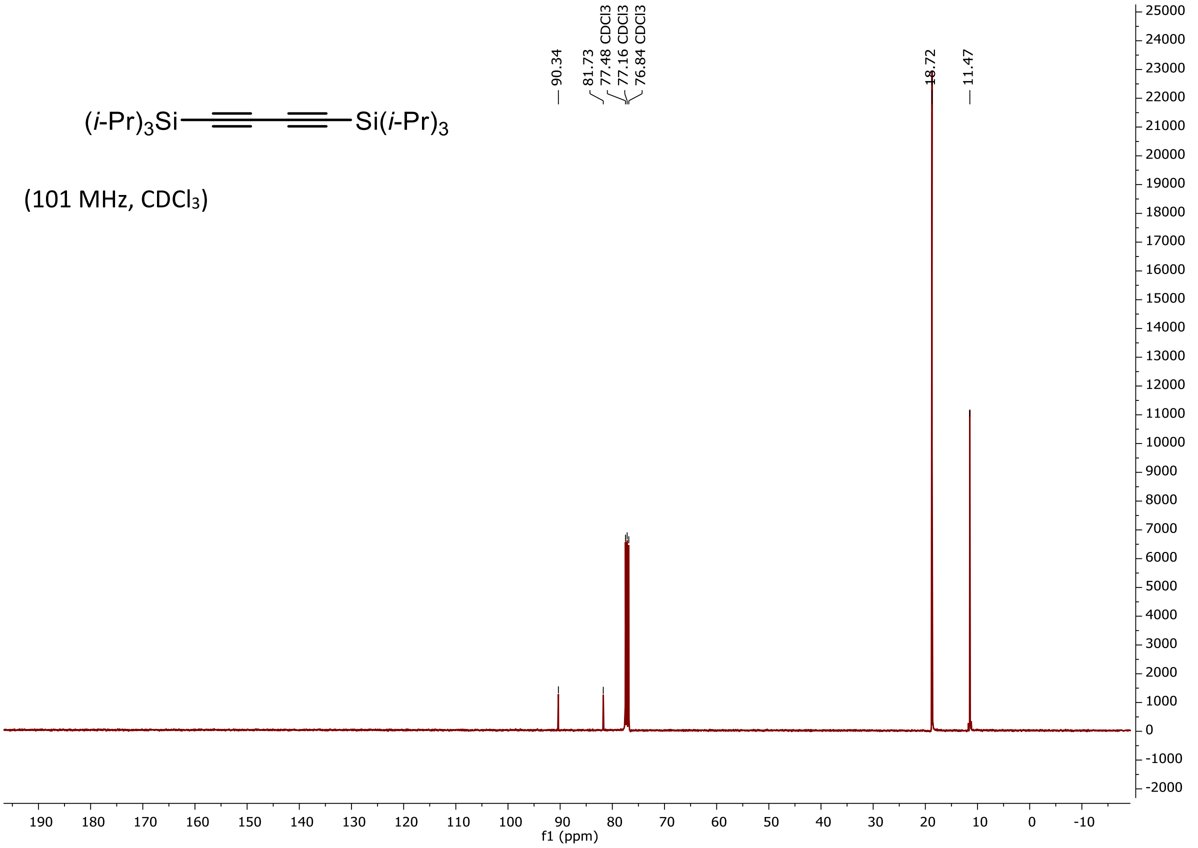


**Figure S10**. $^{13}$C NMR spectrum of **2c**.

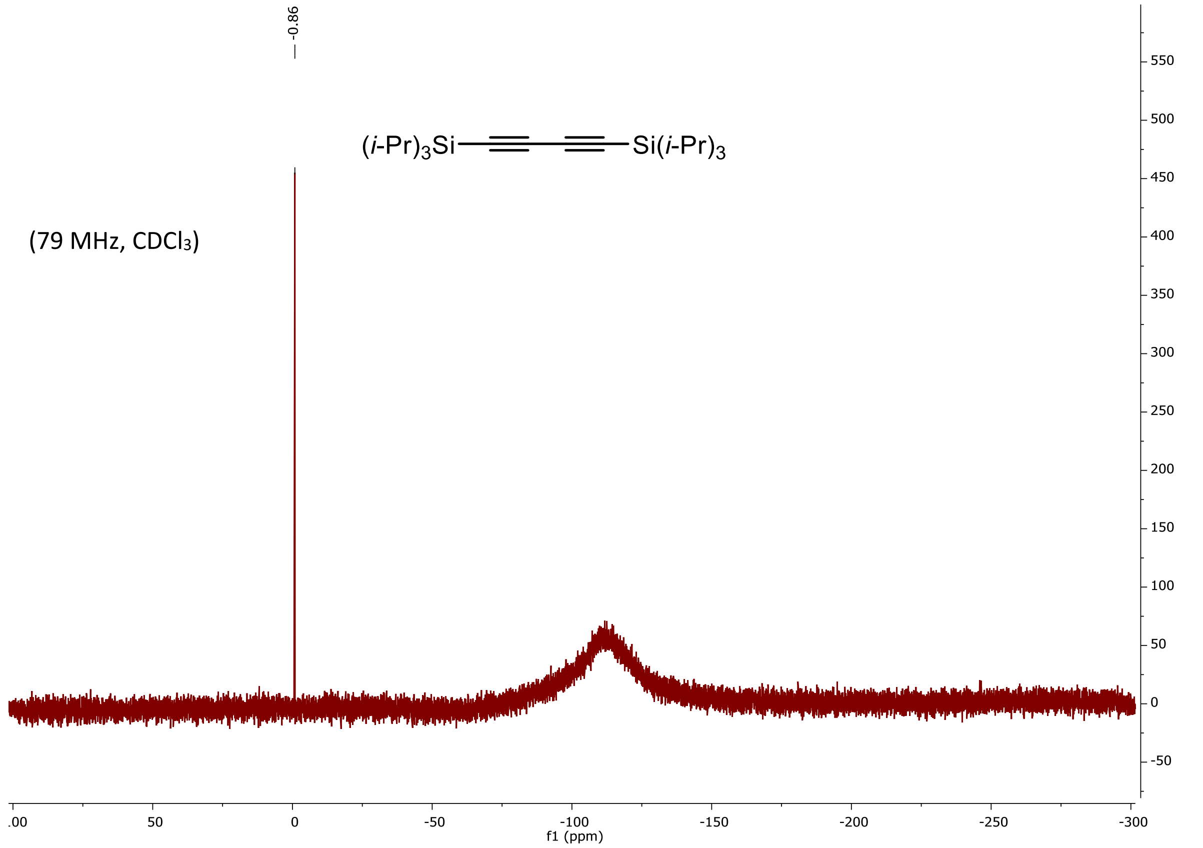


**Figure S11**. $^{29}$Si NMR spectrum of **2c**.

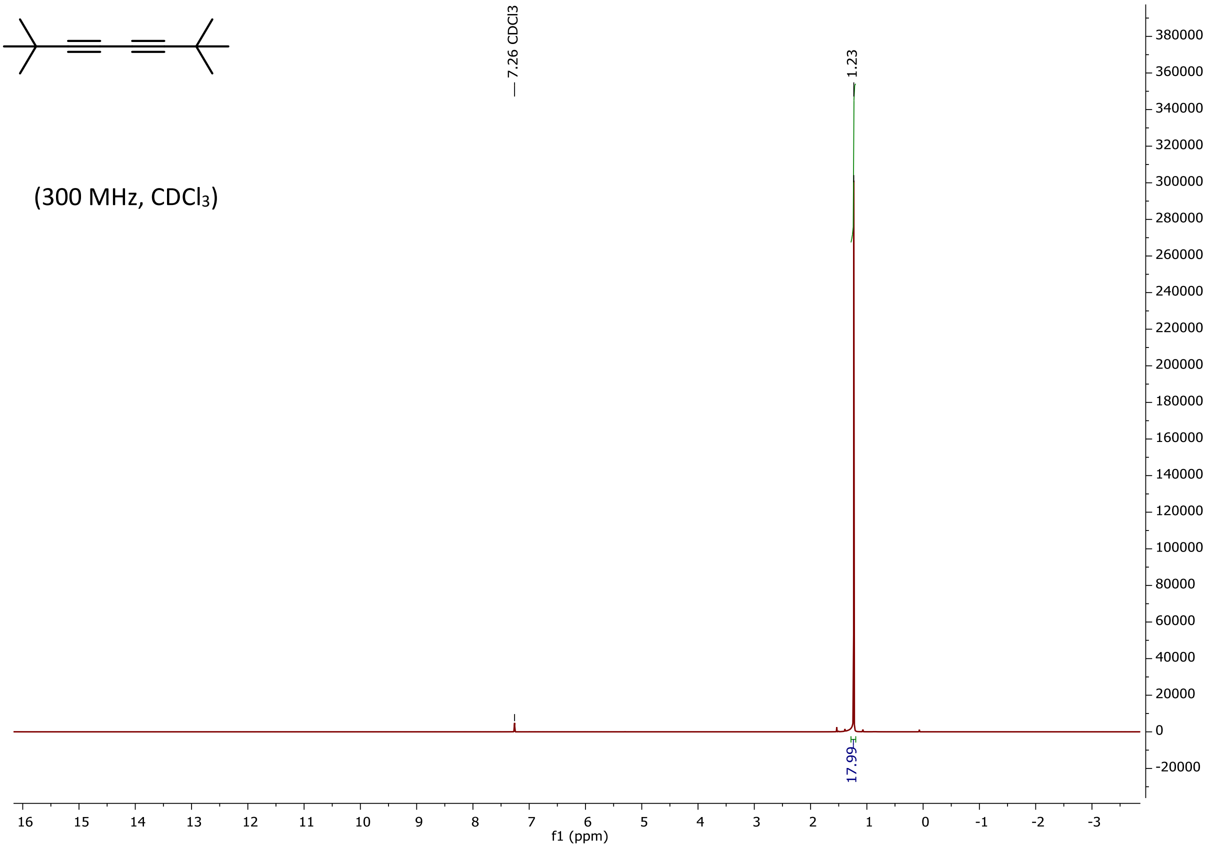


**Figure S12**. $^{1}$H NMR spectrum of **2f**.

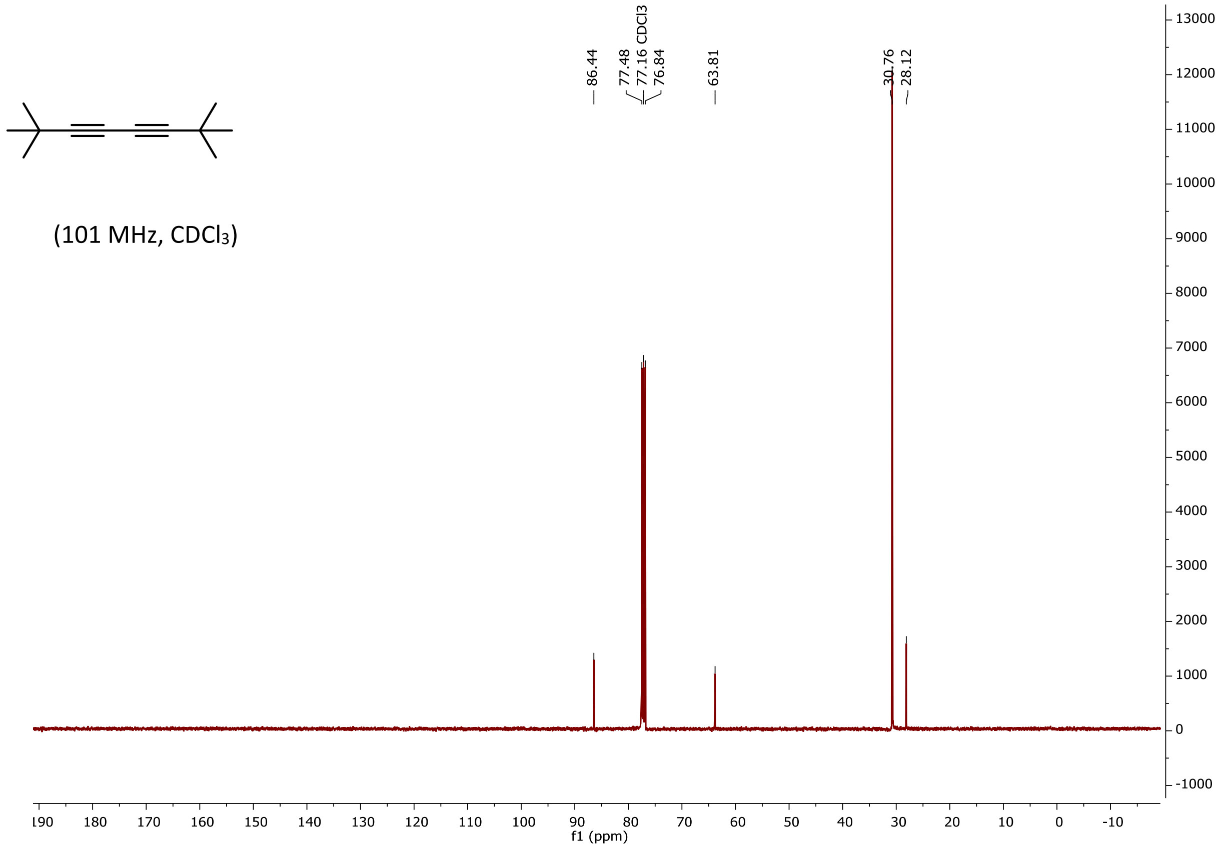


**Figure S13**. $^{13}$C NMR spectrum of **2f**.

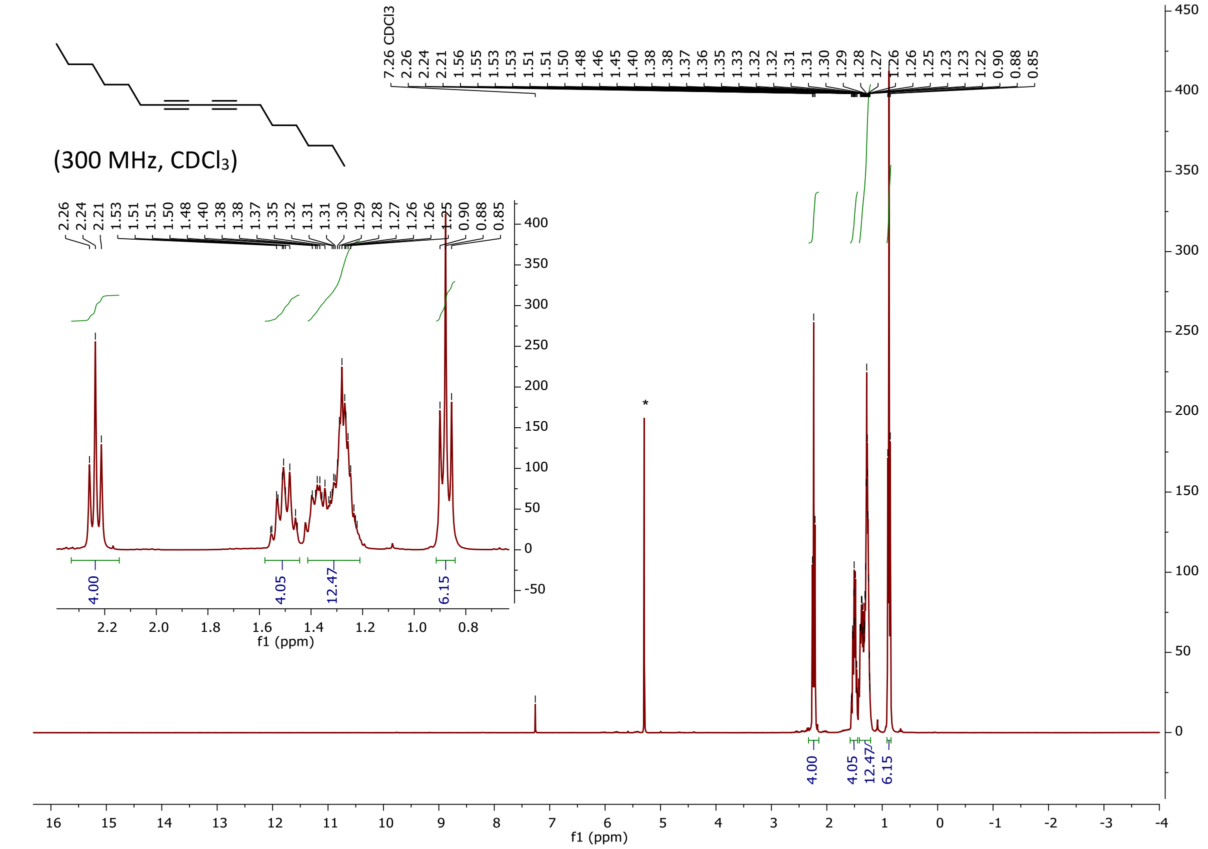


**Figure S14**. $^{1}$H NMR spectrum of **2e**. * - Traces of $CH_2Cl_2$

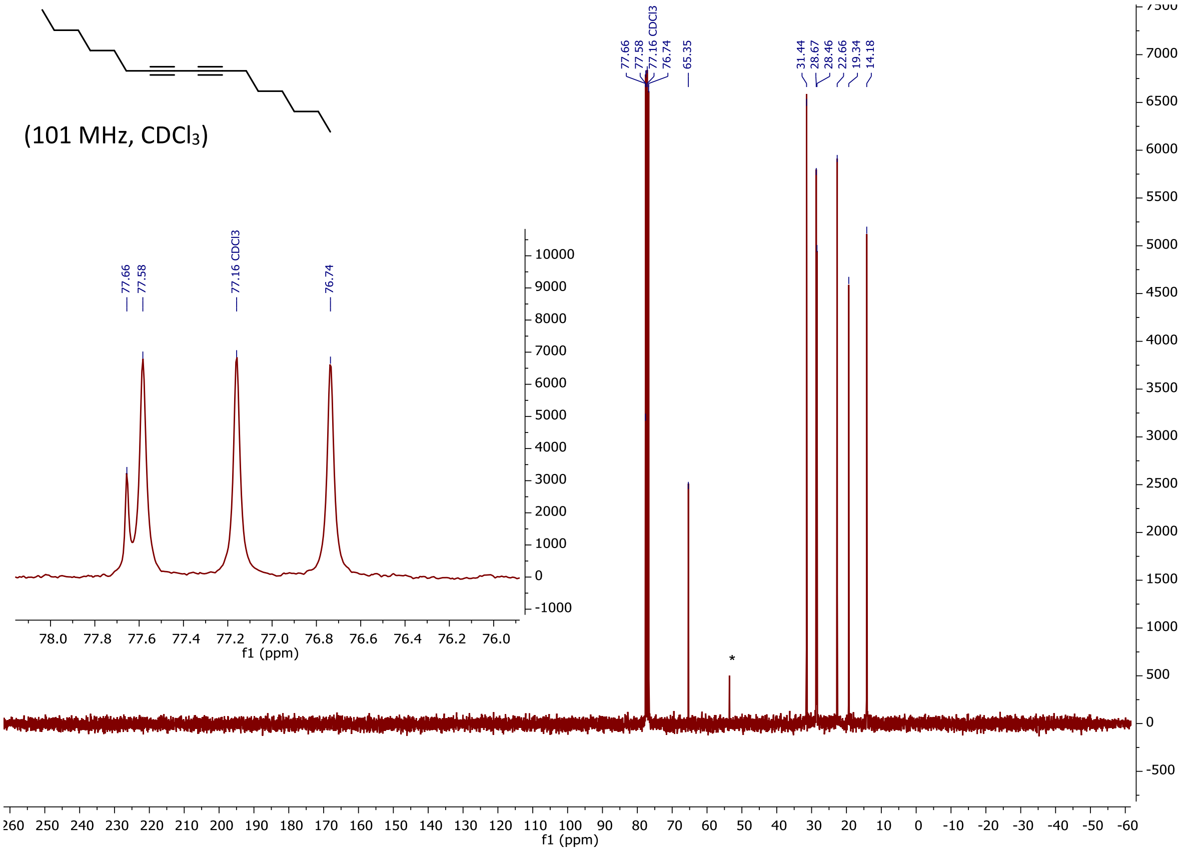


**Figure S15**. $^{13}$C NMR spectrum of **2e**. * - Traces of $CH_2Cl_2$

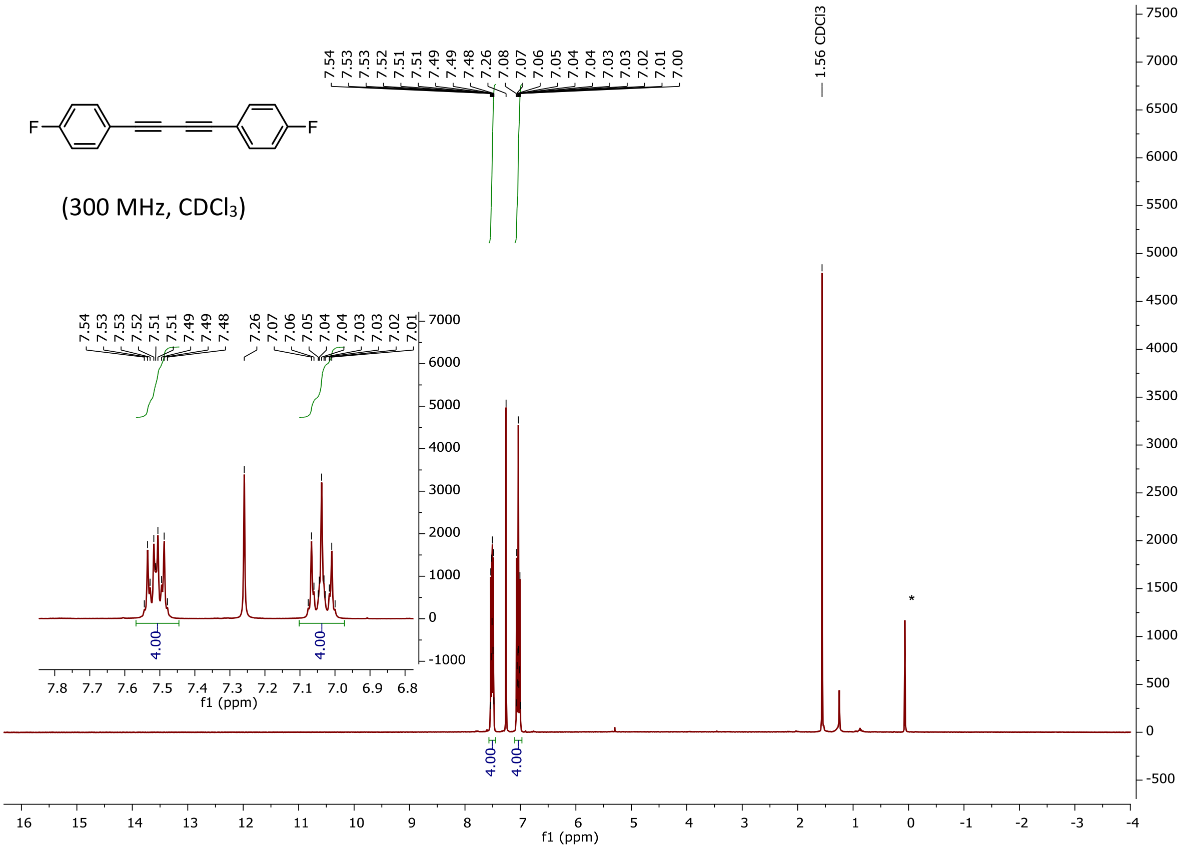


**Figure S16**. $^1$H NMR spectrum of **2h**. * - Grease.

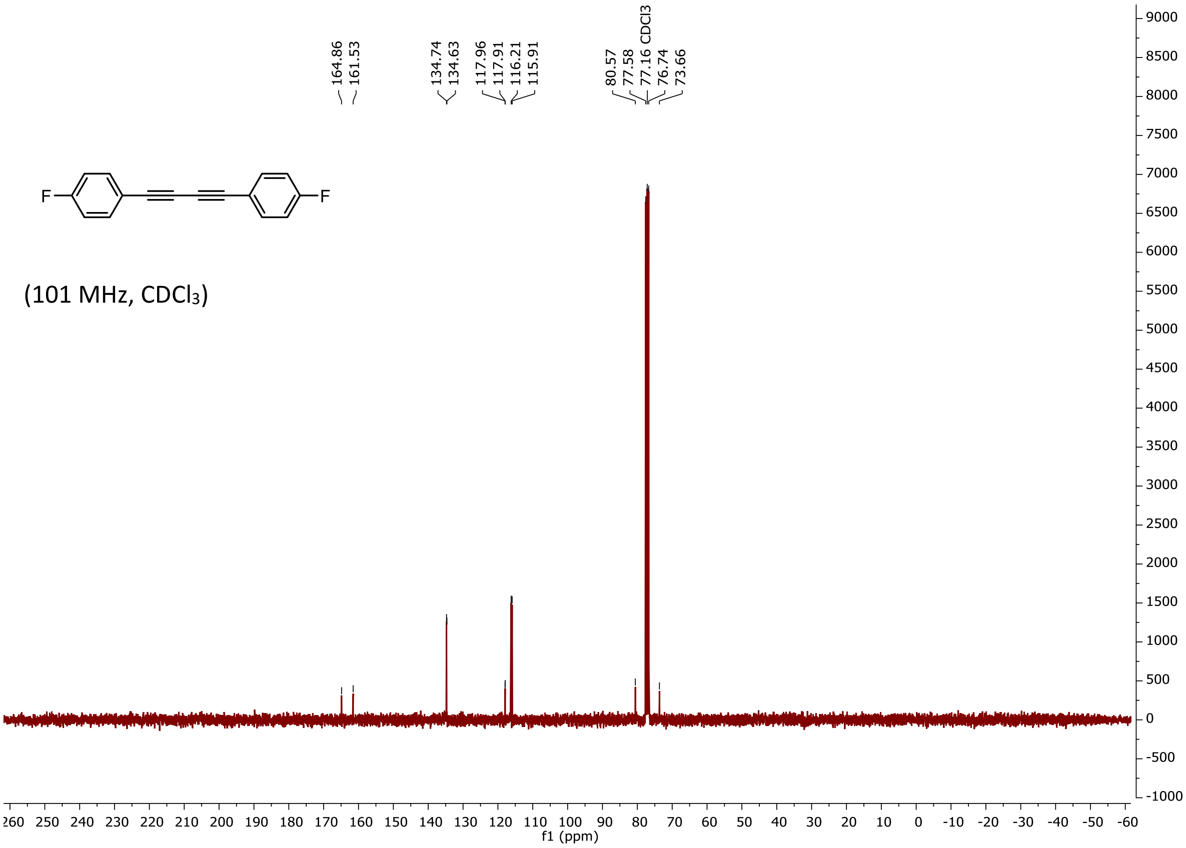


**Figure S17**. $^{13}C$ NMR spectrum of **2h**.

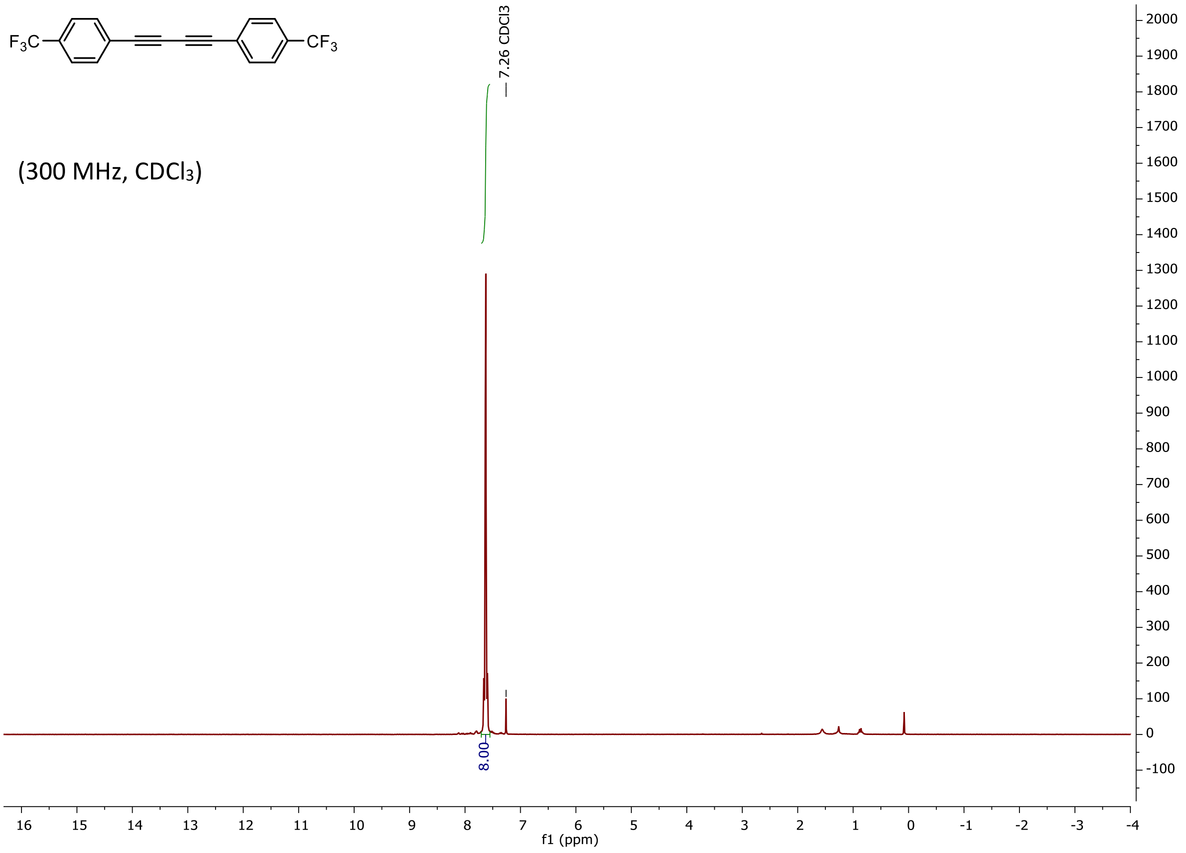


**Figure S18**. $^{1}H$ NMR spectrum of **2i**.

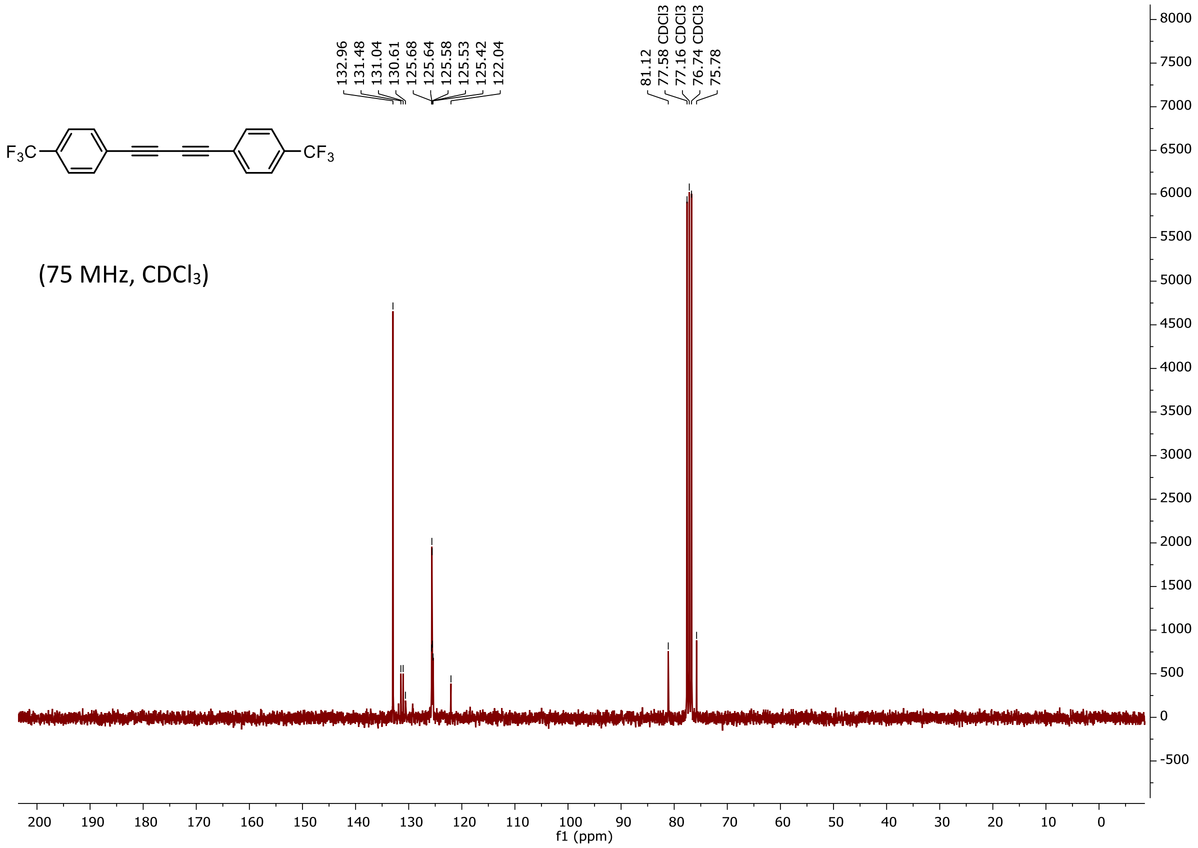


**Figure S19**. $^{13}$C NMR spectrum of **2i**.

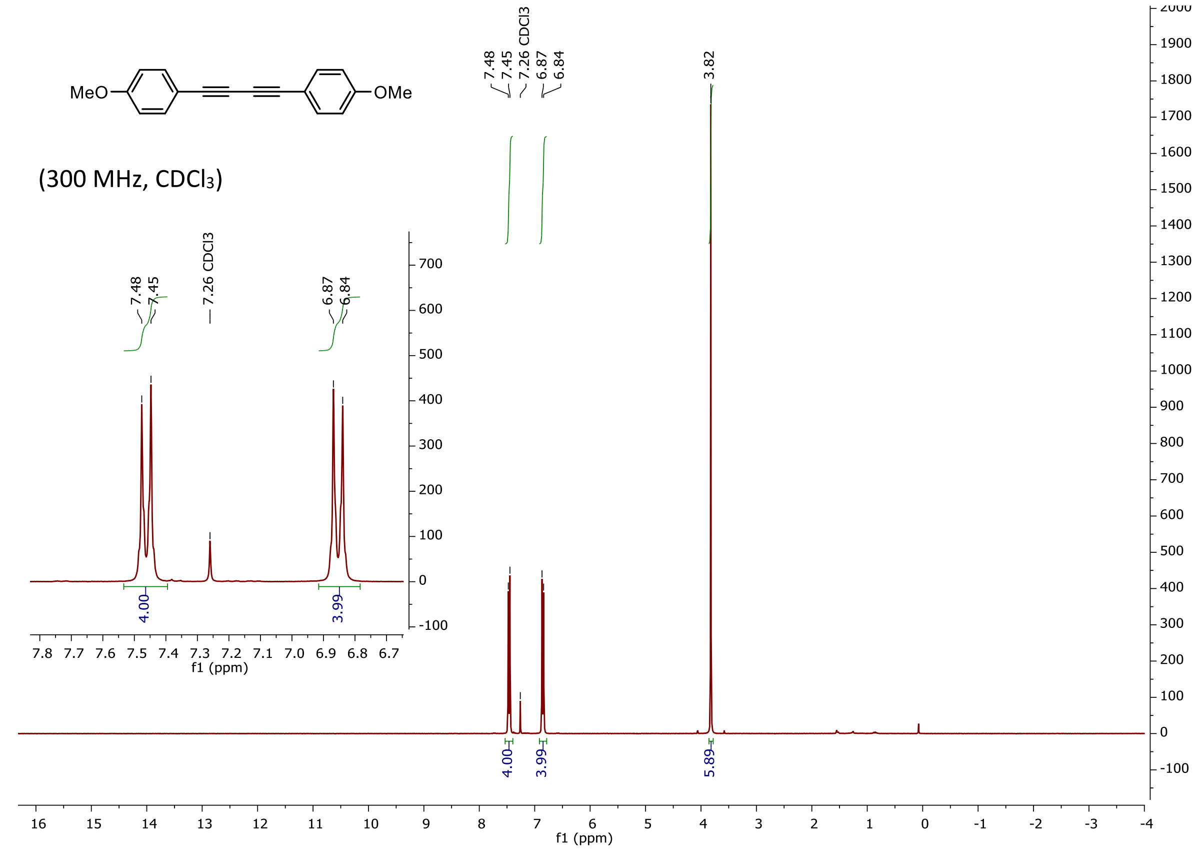


**Figure S20**. $^{1}$H NMR spectrum of **2j**.

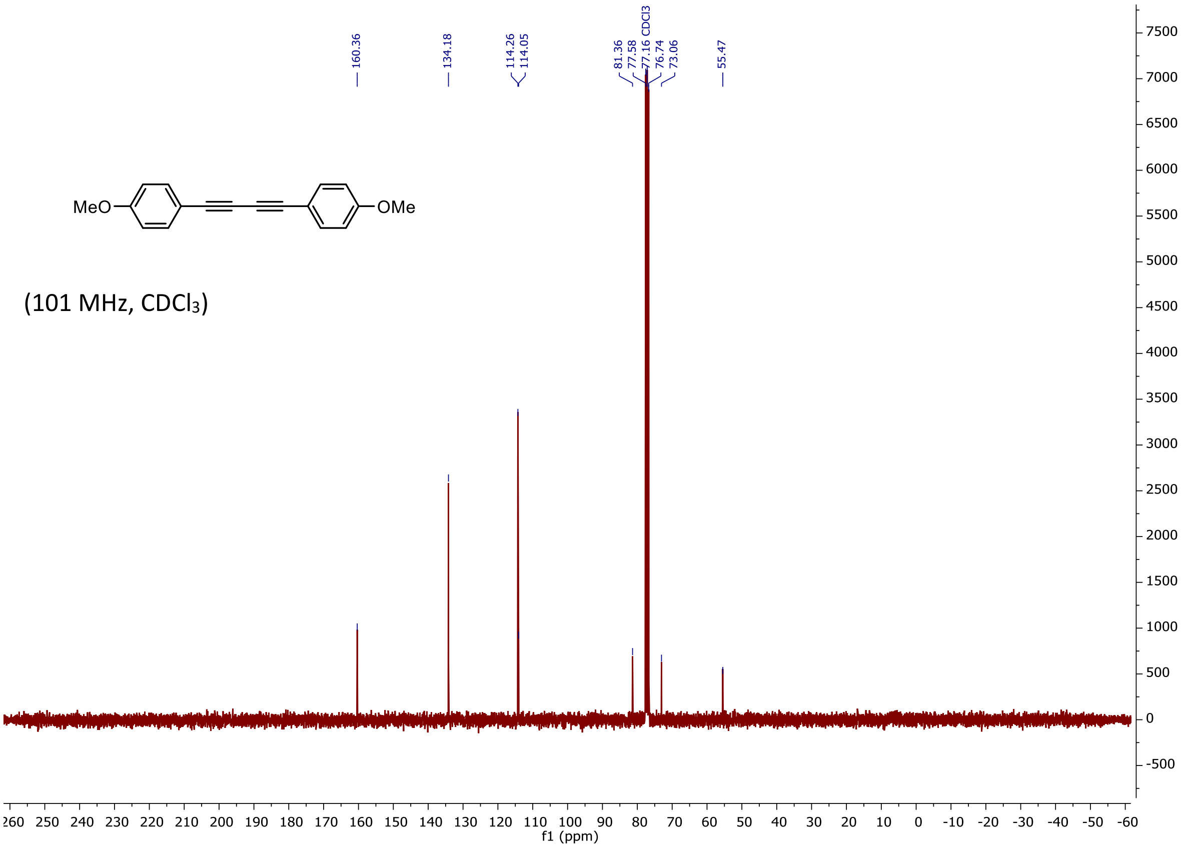


**Figure S21**. $^{13}$C NMR spectrum of **2j**.

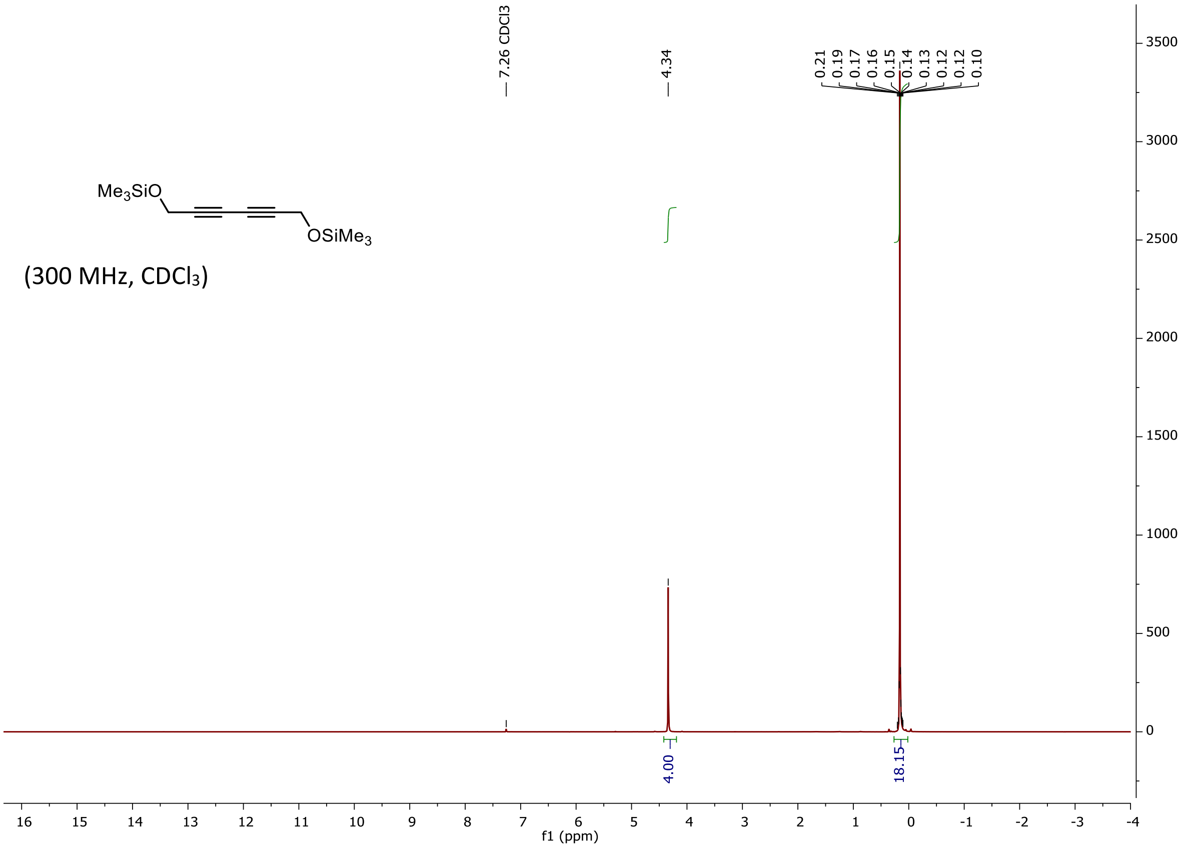


**Figure S22** $^{1}$H NMR spectrum of **2l**.

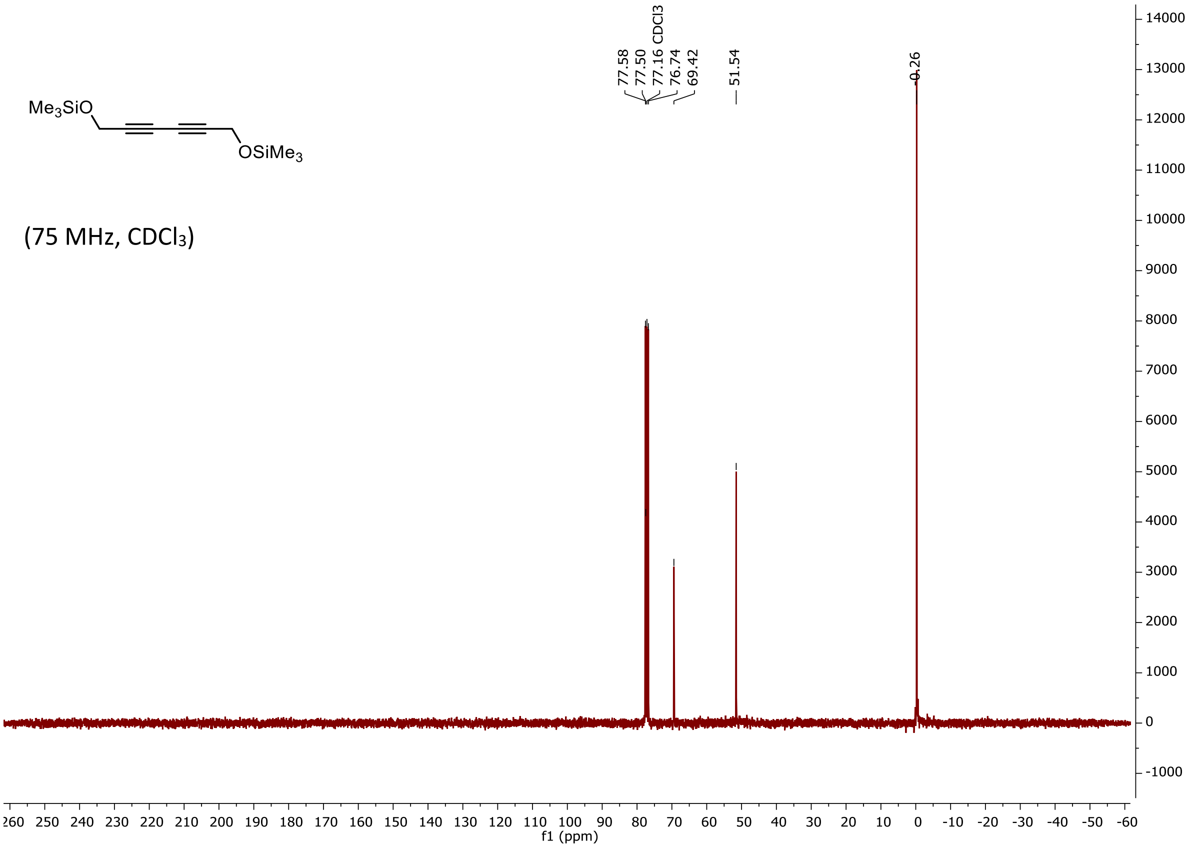


**Figure S23**. $^{13}$C NMR spectrum of **2l**.

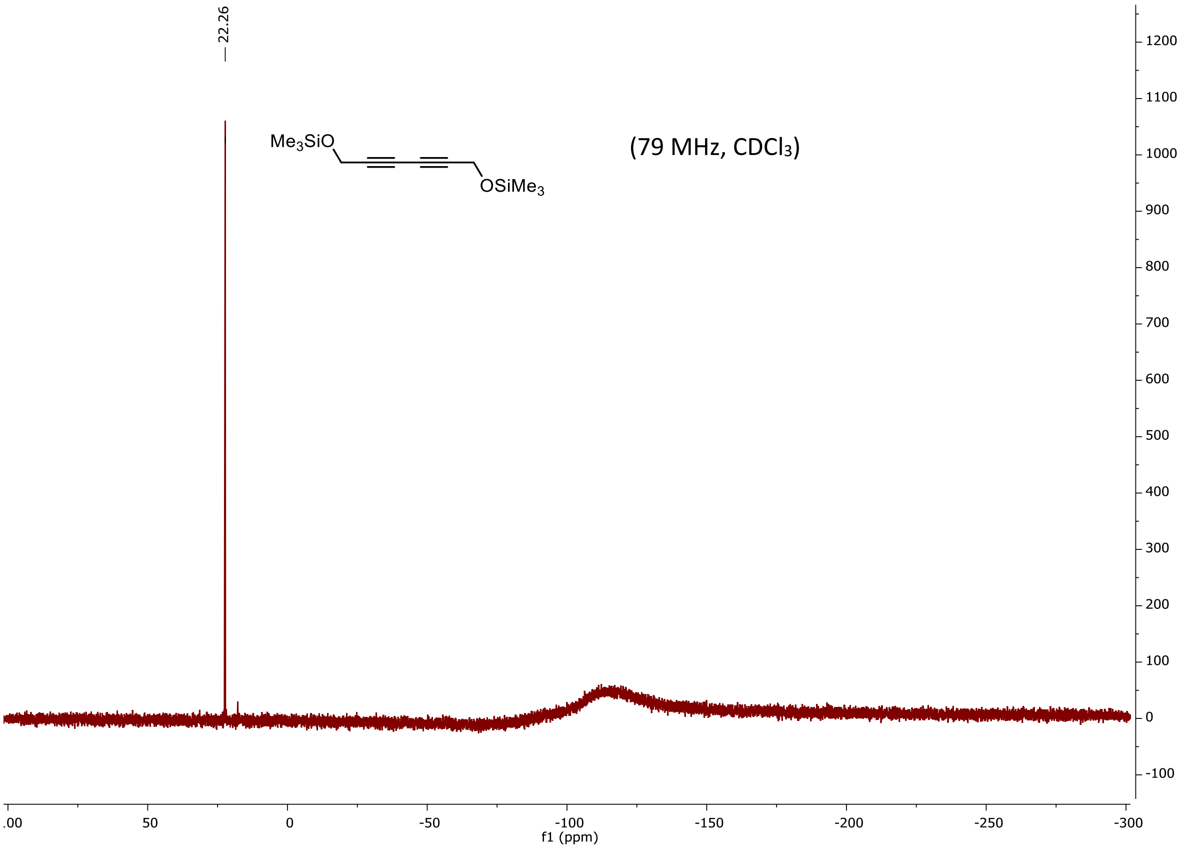


**Figure S24**. $^{29}$Si NMR spectrum of **2l**.

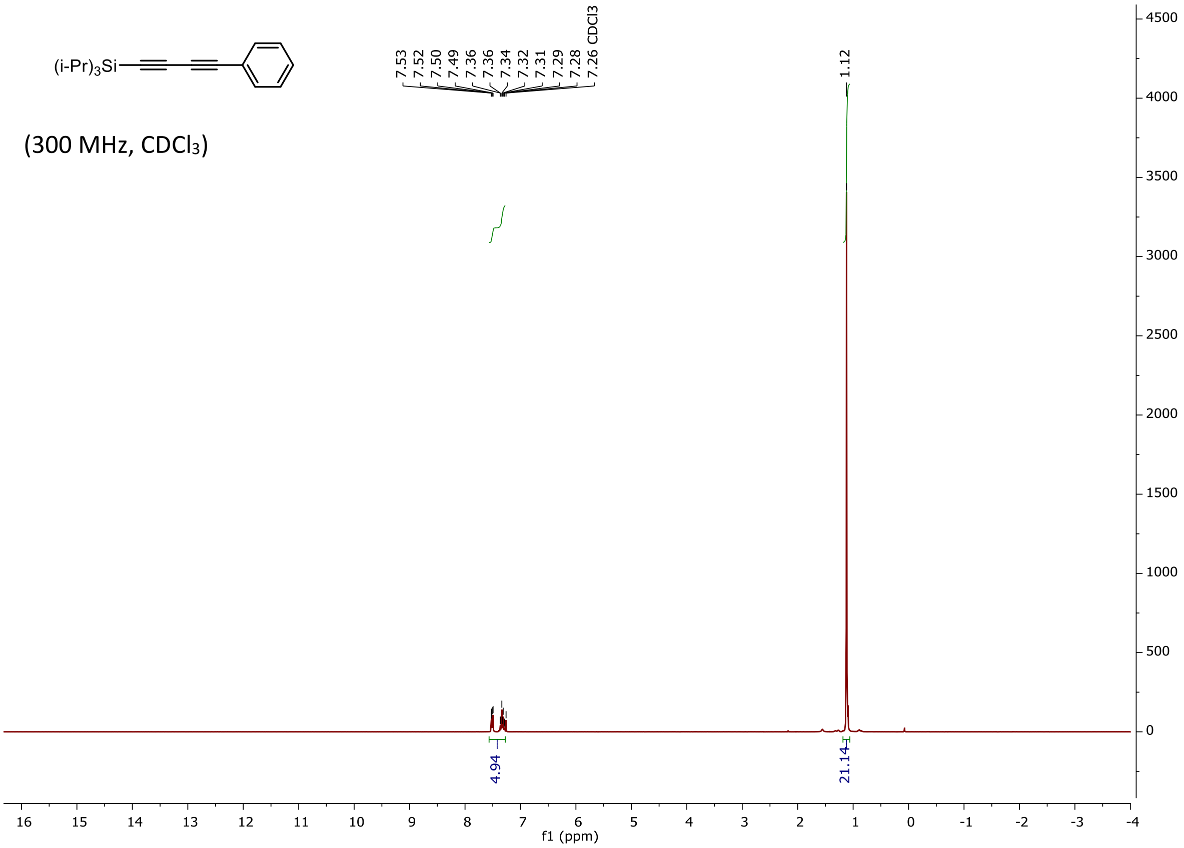


**Figure S25**. $^{1}$H NMR spectrum of **2m**.

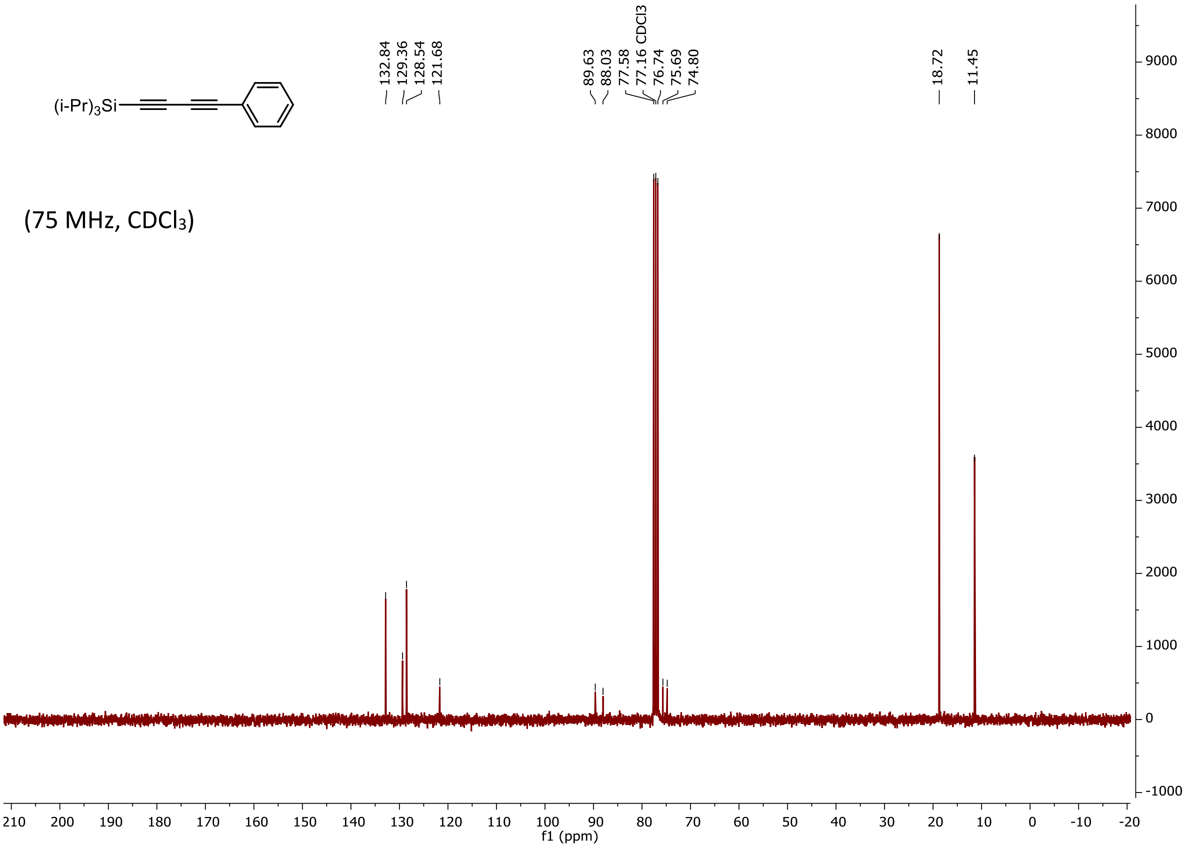


**Figure S26**. $^{13}$C NMR spectrum of **2m**.

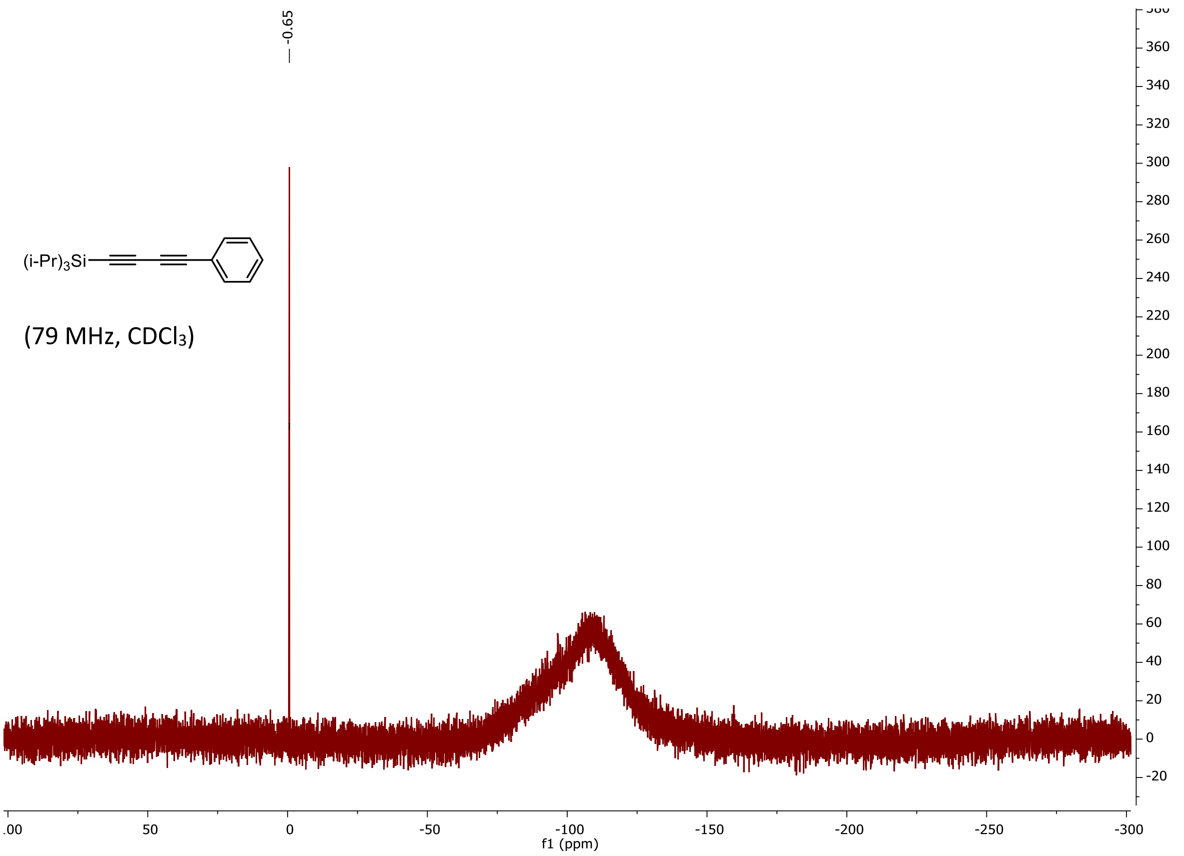


**Figure 27**. $^{29}$Si NMR spectrum of **2m**.

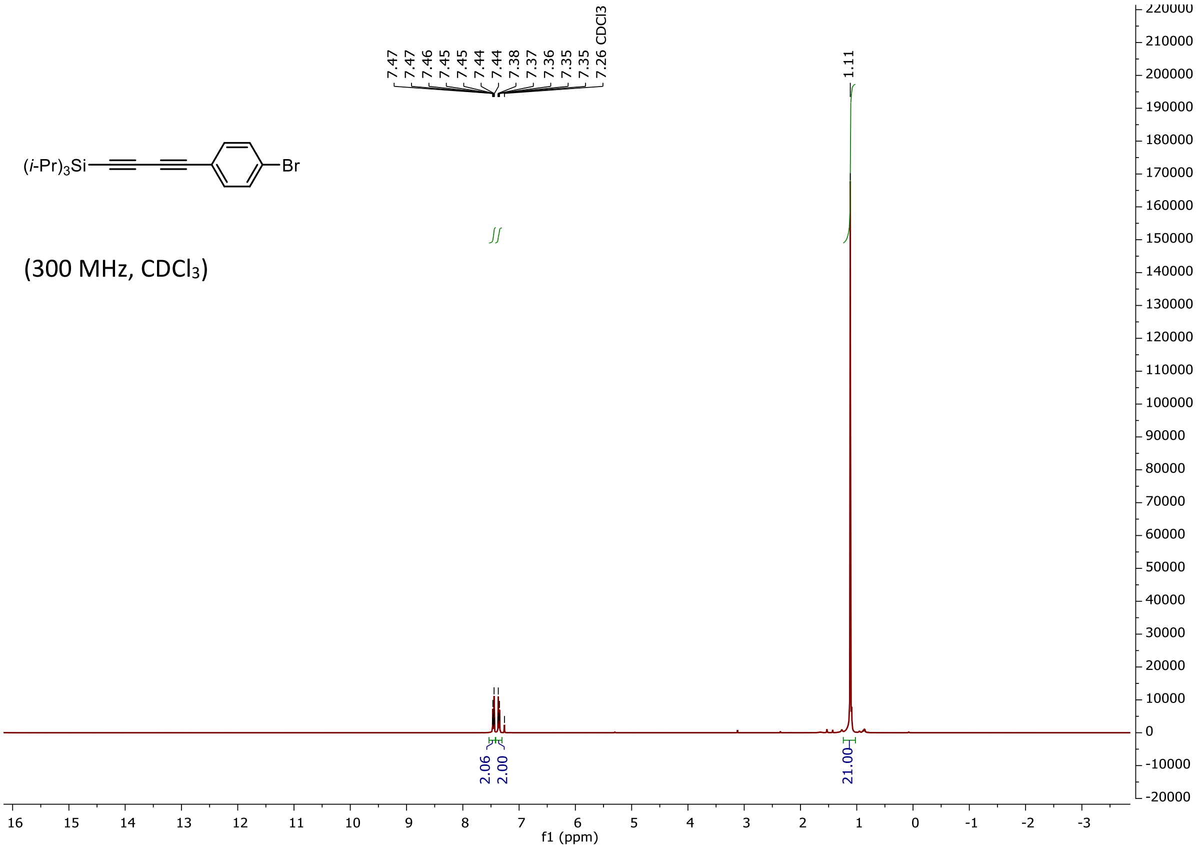


**Figure S28**. $^{1}$H NMR spectrum of **2n**.

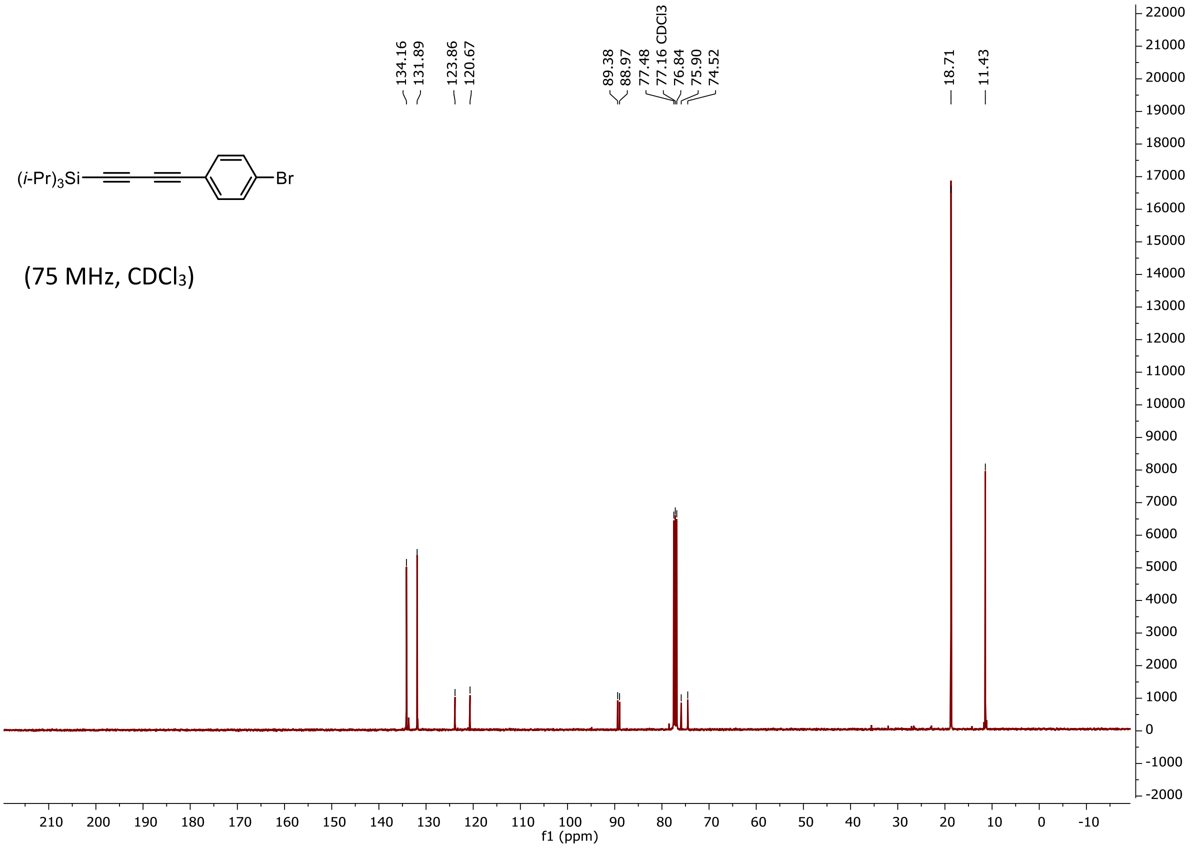


**Figure S29**. $^{13}$C NMR spectrum of **2n**.

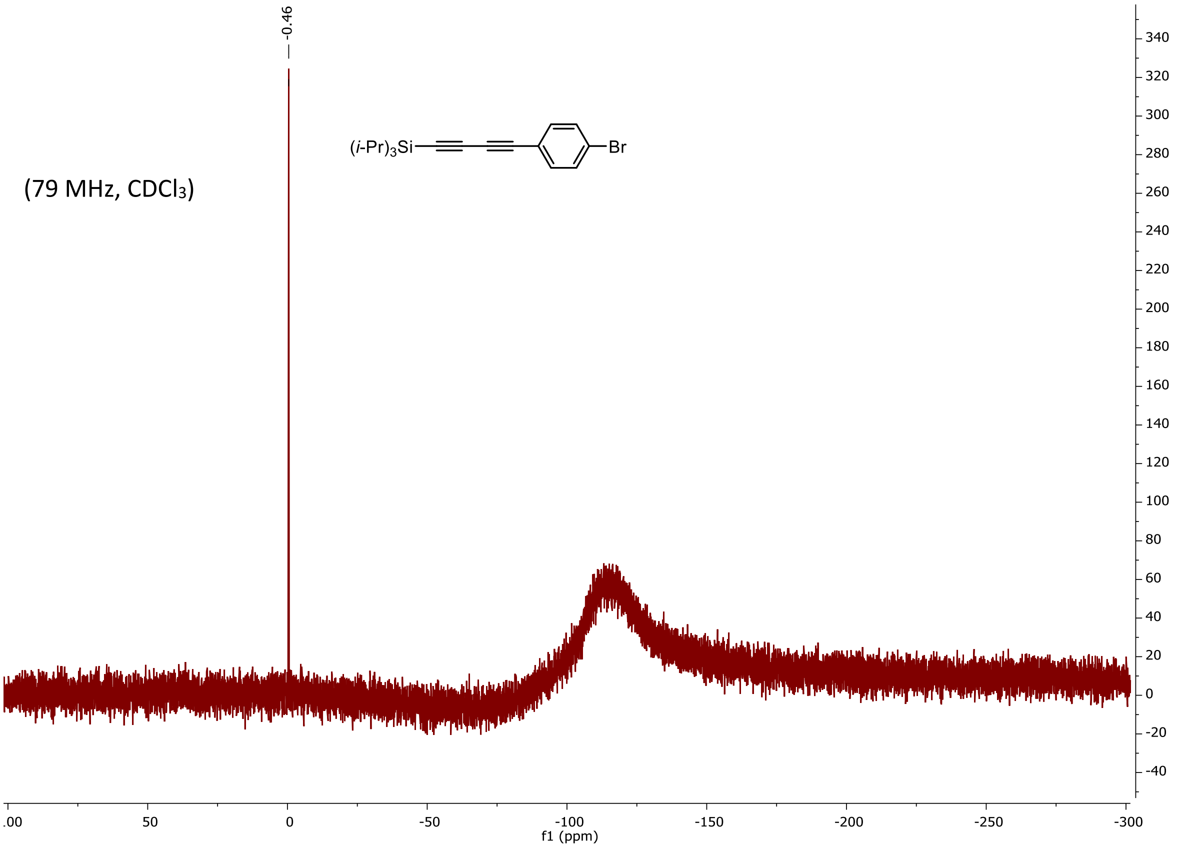


**Figure S30**. $^{29}$Si NMR spectrum of **2n**.

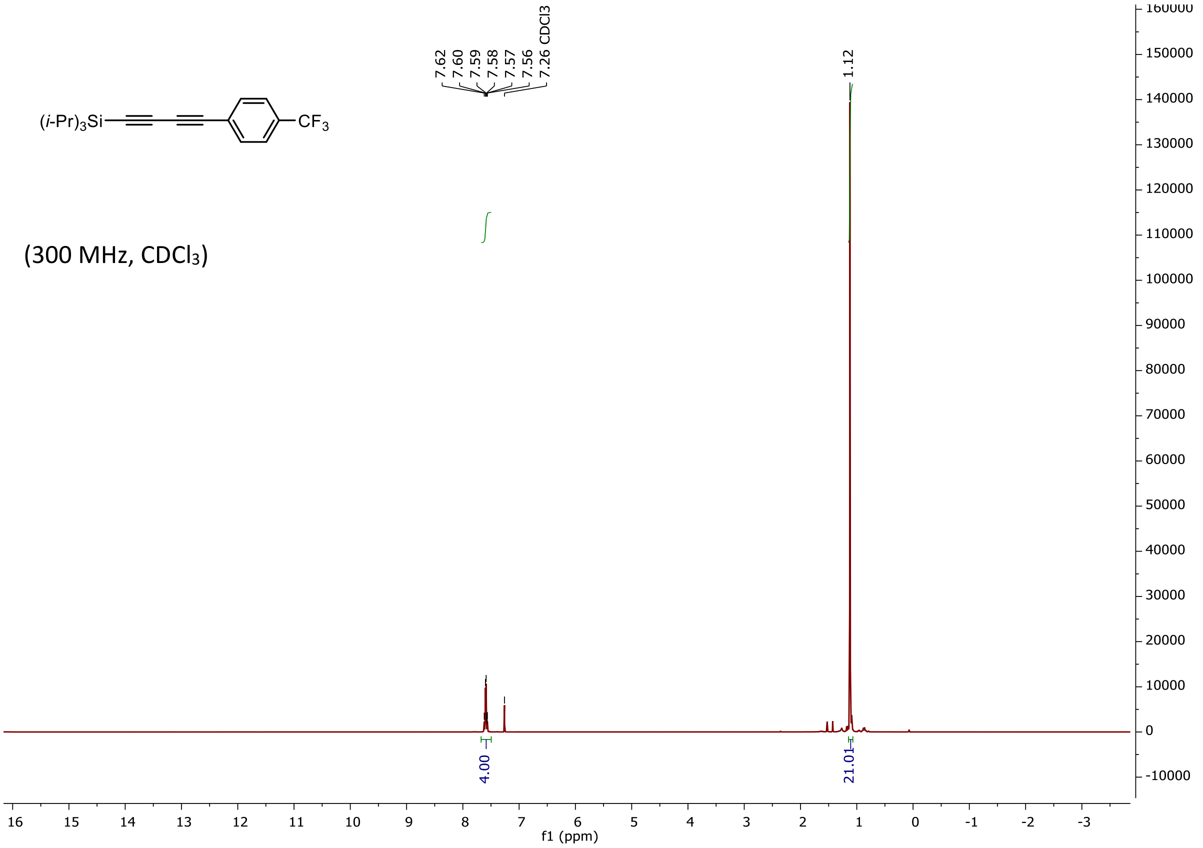


**Figure S31**. $^{1}$H NMR spectrum of **2o**.

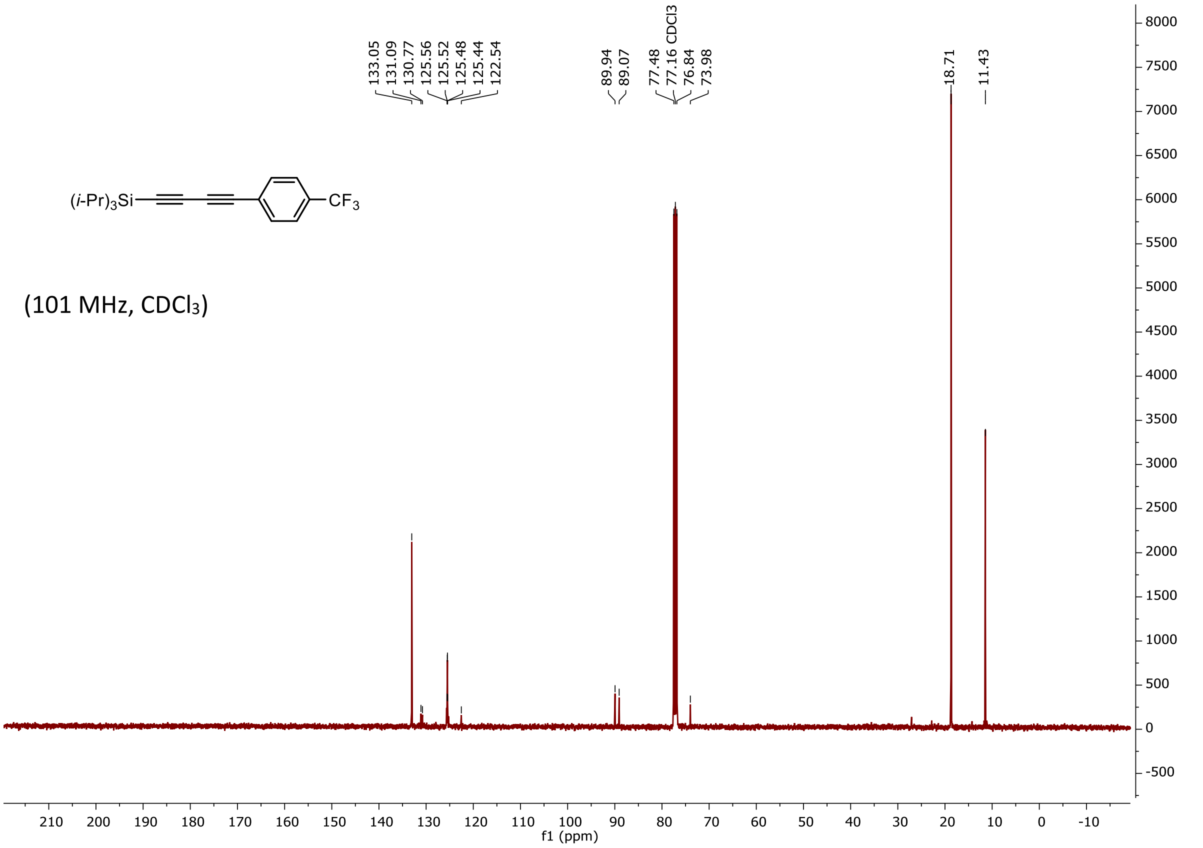


**Figure S32**. $^{13}$C NMR spectrum of **2o**.

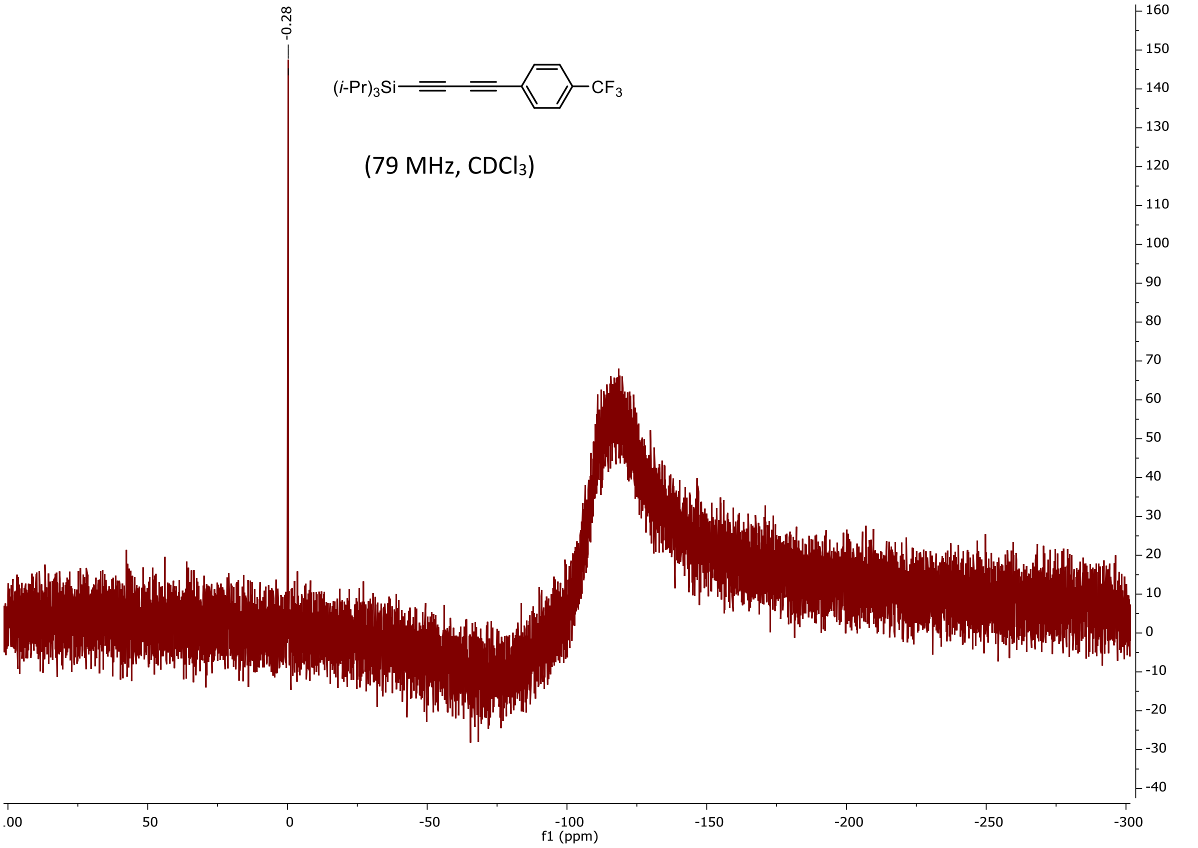


**Figure S33**. $^{29}$Si NMR spectrum of **2o**.

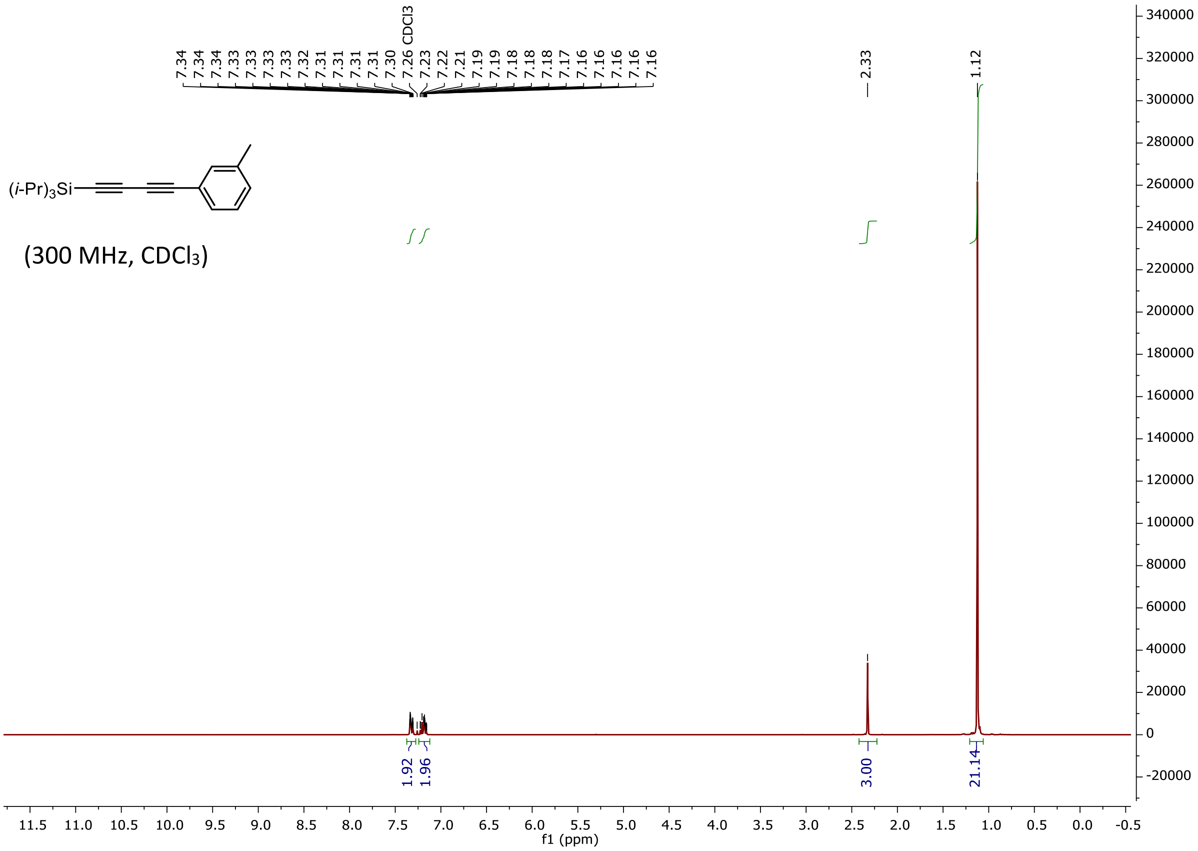


**Figure S34**. $^{1}$H NMR spectrum of (**2p**)

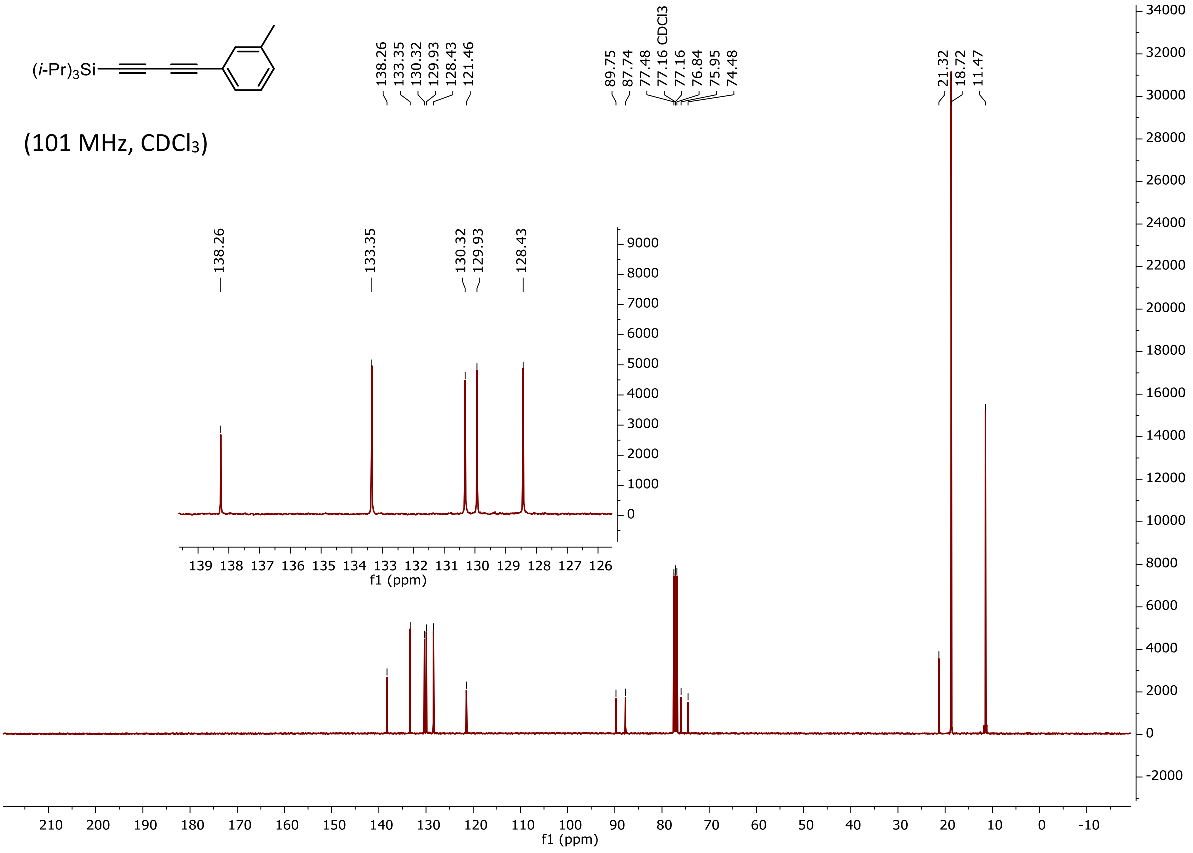


**Figure S35**. [11]C NMR spectrum of (**2p**)

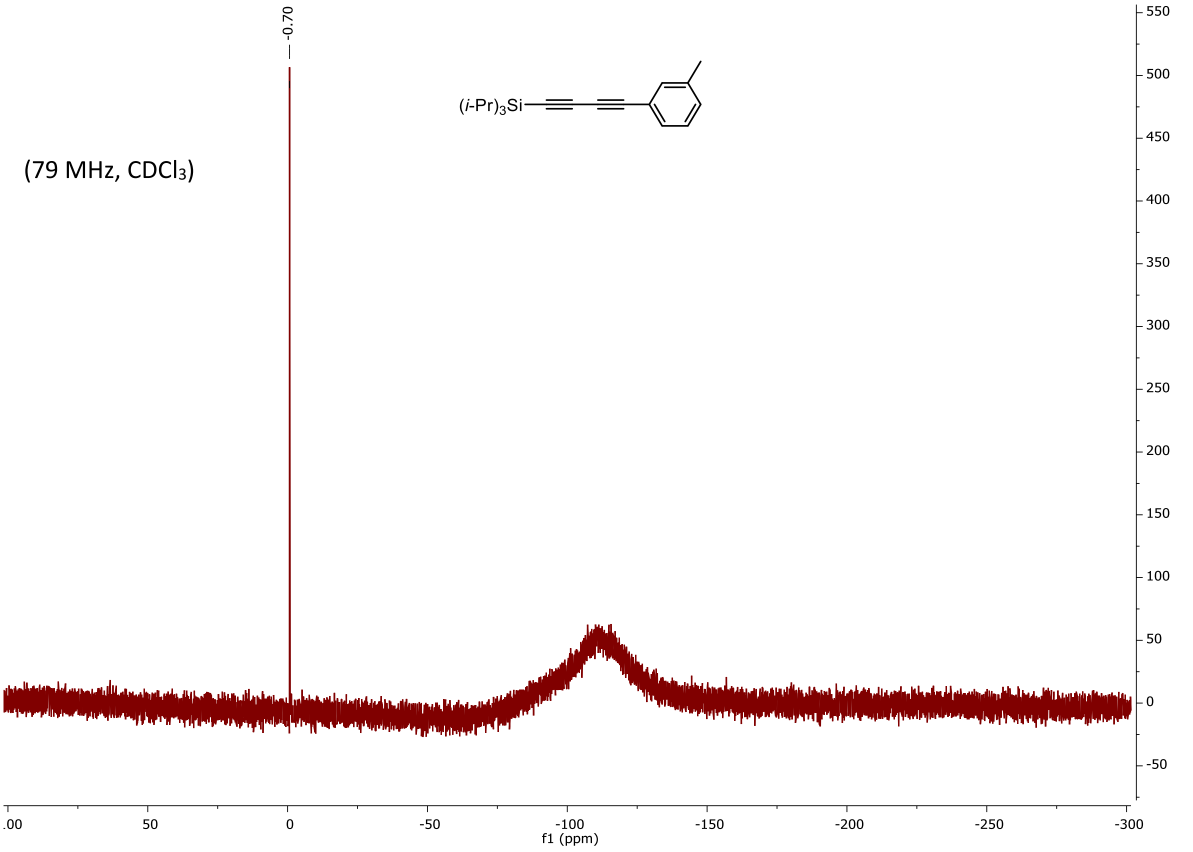


**Figure S36**. $^{29}$Si NMR spectrum of (**2p**)

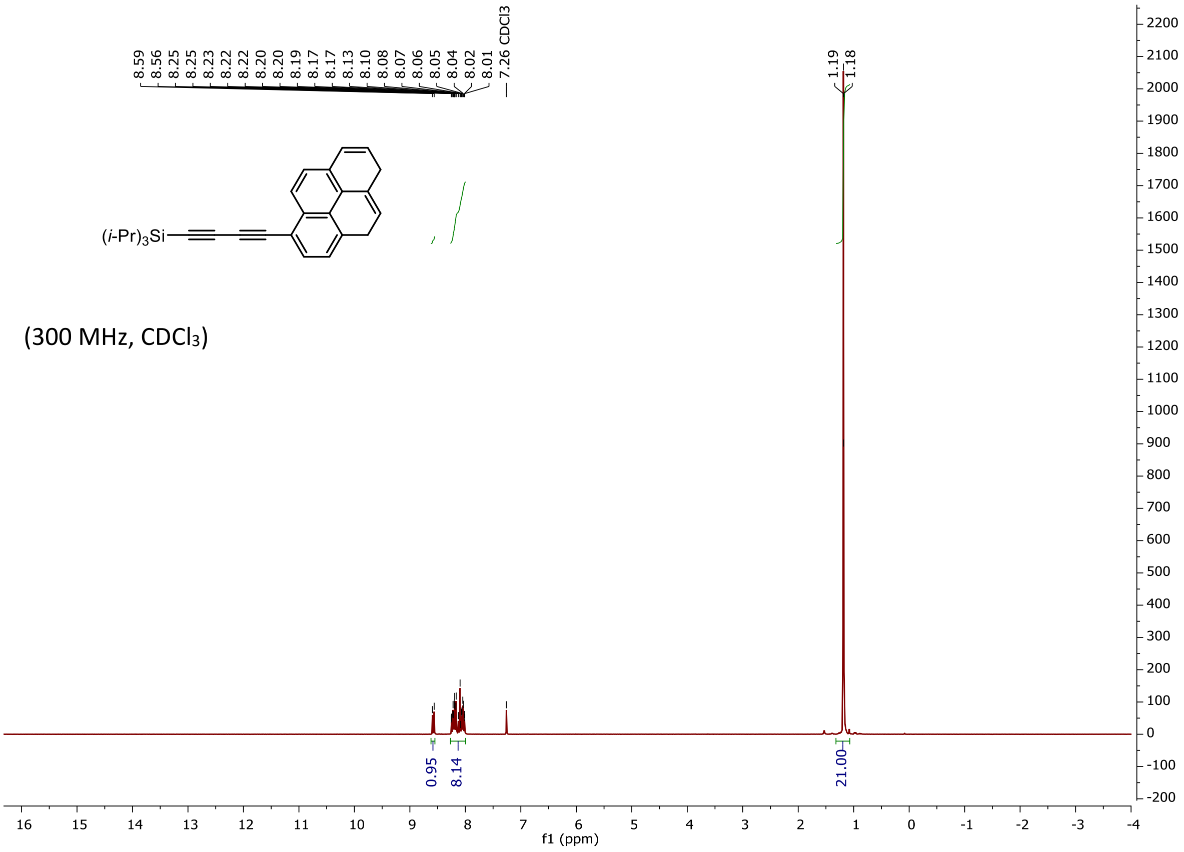


**Figure S37**. $^{1}$H NMR spectrum of **2q**.

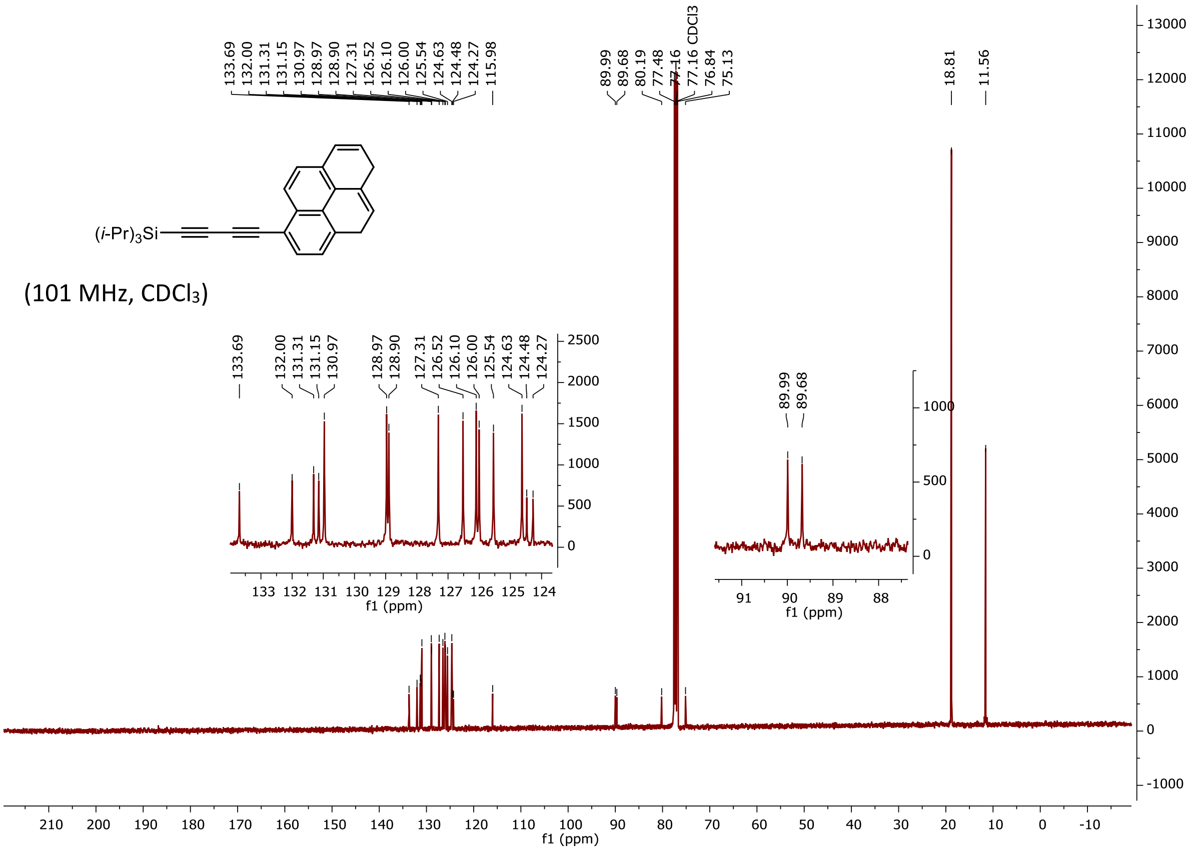


**Figure S38**. $^{13}$C NMR spectrum of **2q**.

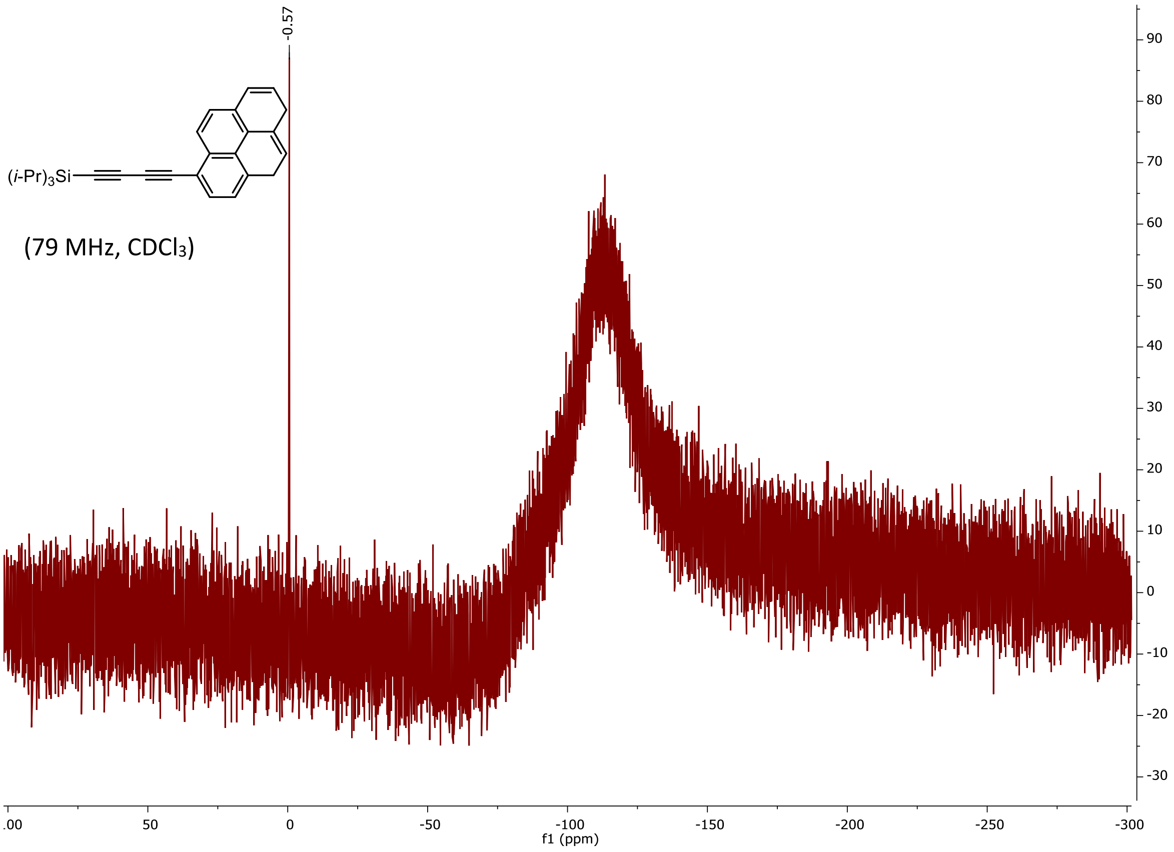


**Figure S39**. $^{29}$Si NMR spectrum of **2q**.

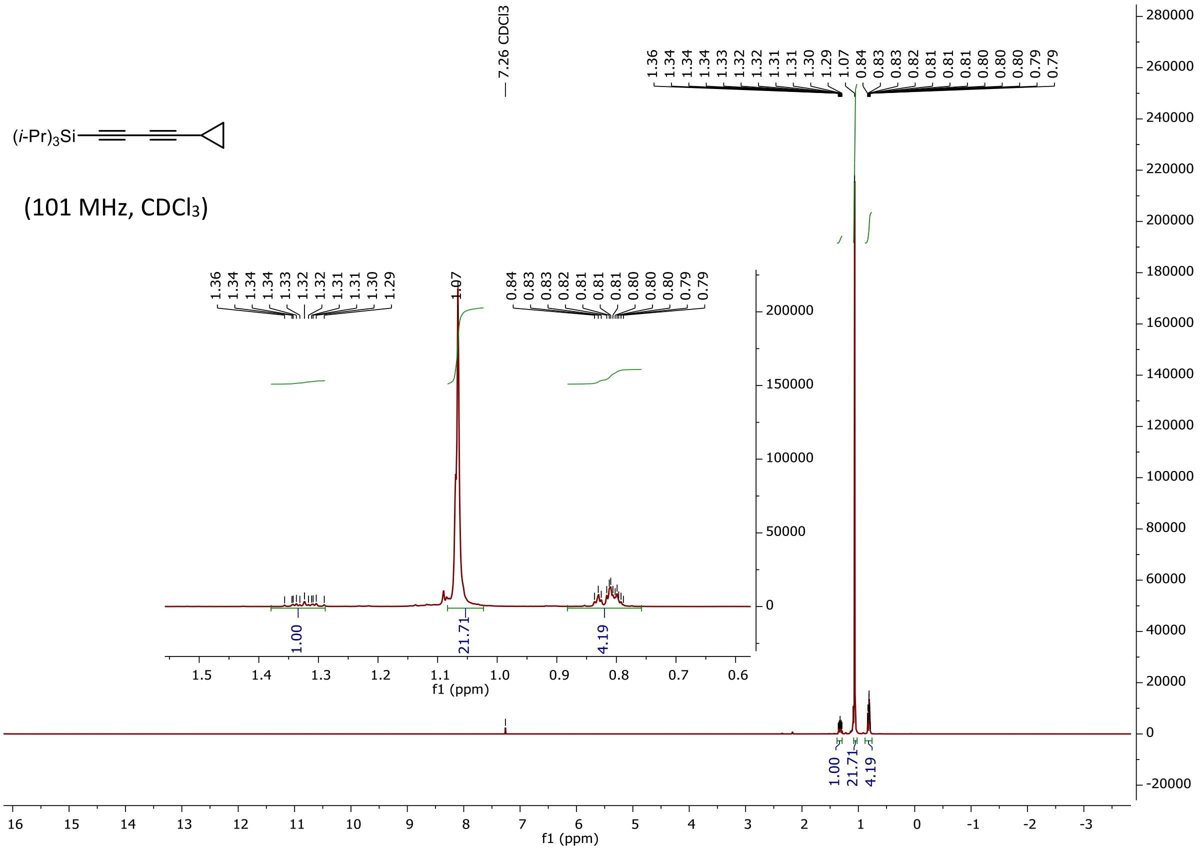


**Figure S40**. $^{1}$H NMR spectrum of (**2r**)

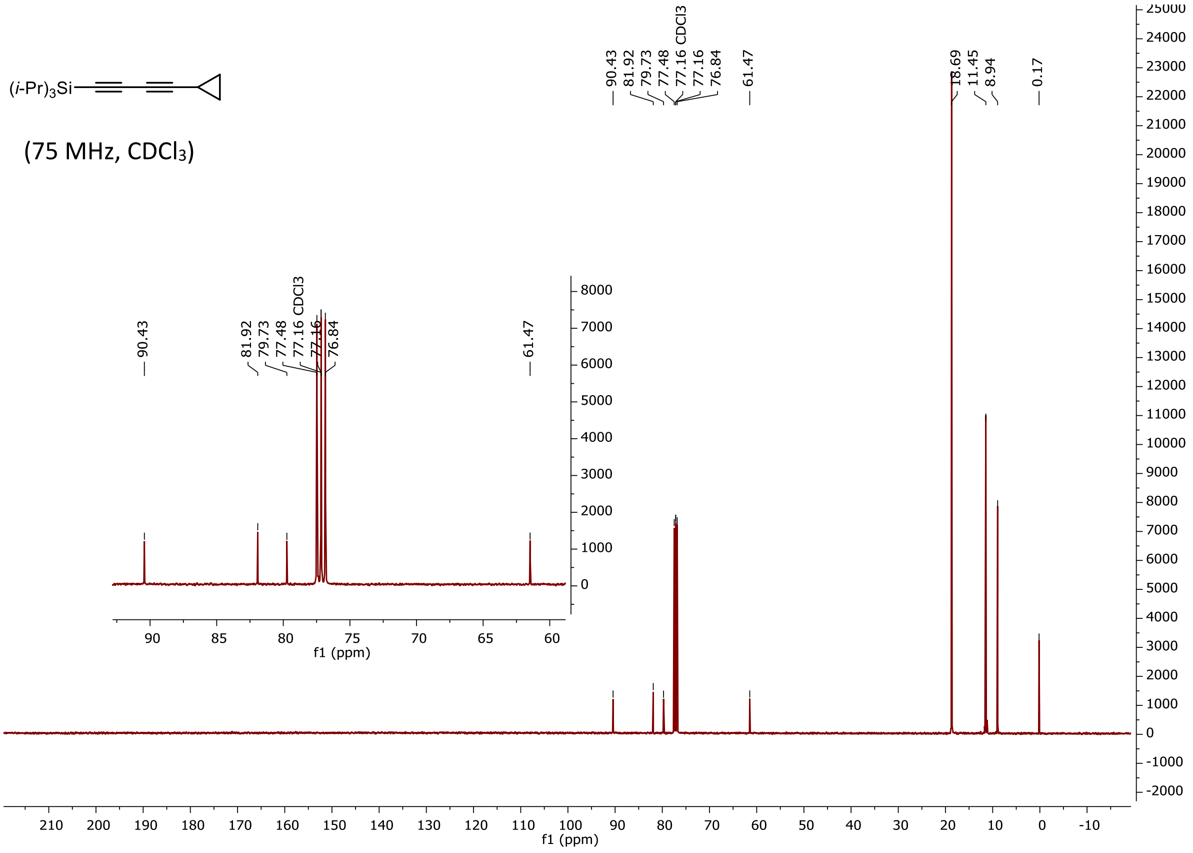


**Figure S41**. $^{11}$C NMR spectrum of (**2r**)

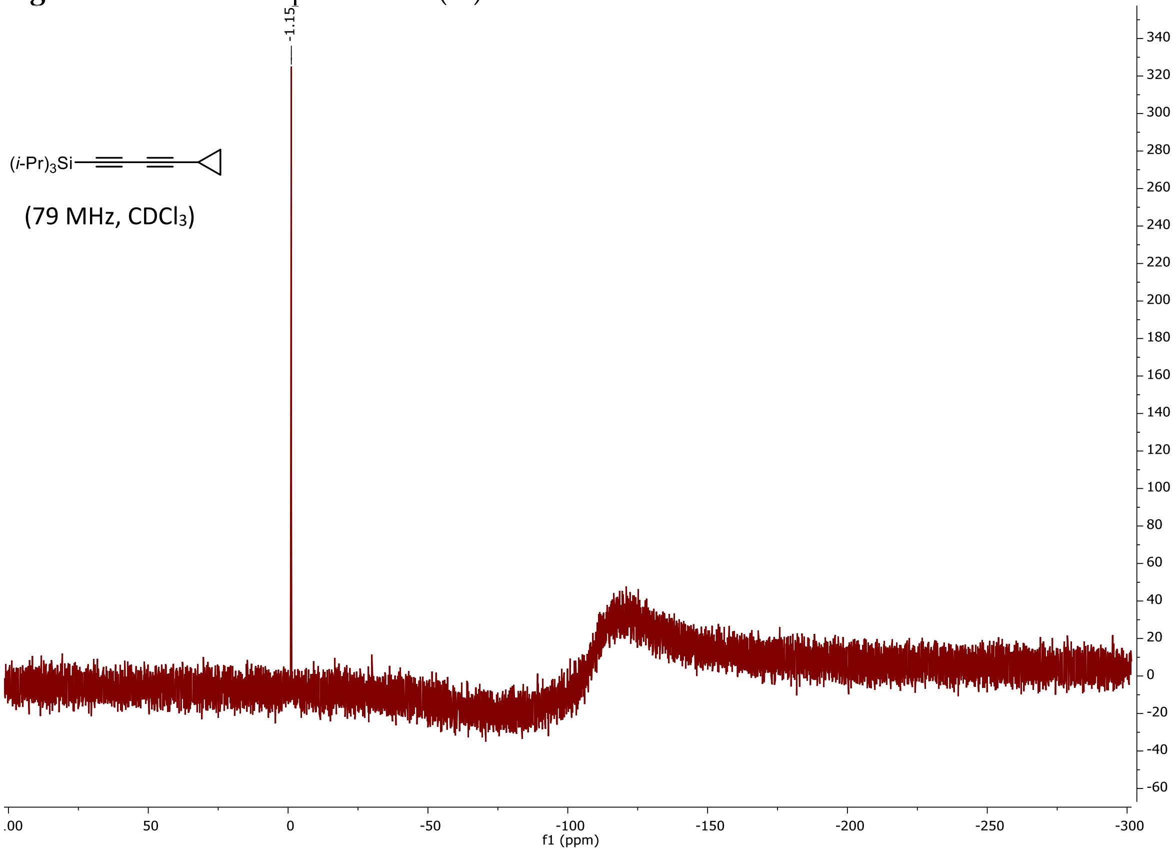


**Figure S42**. $^{29}$Si NMR spectrum of (**2r**)

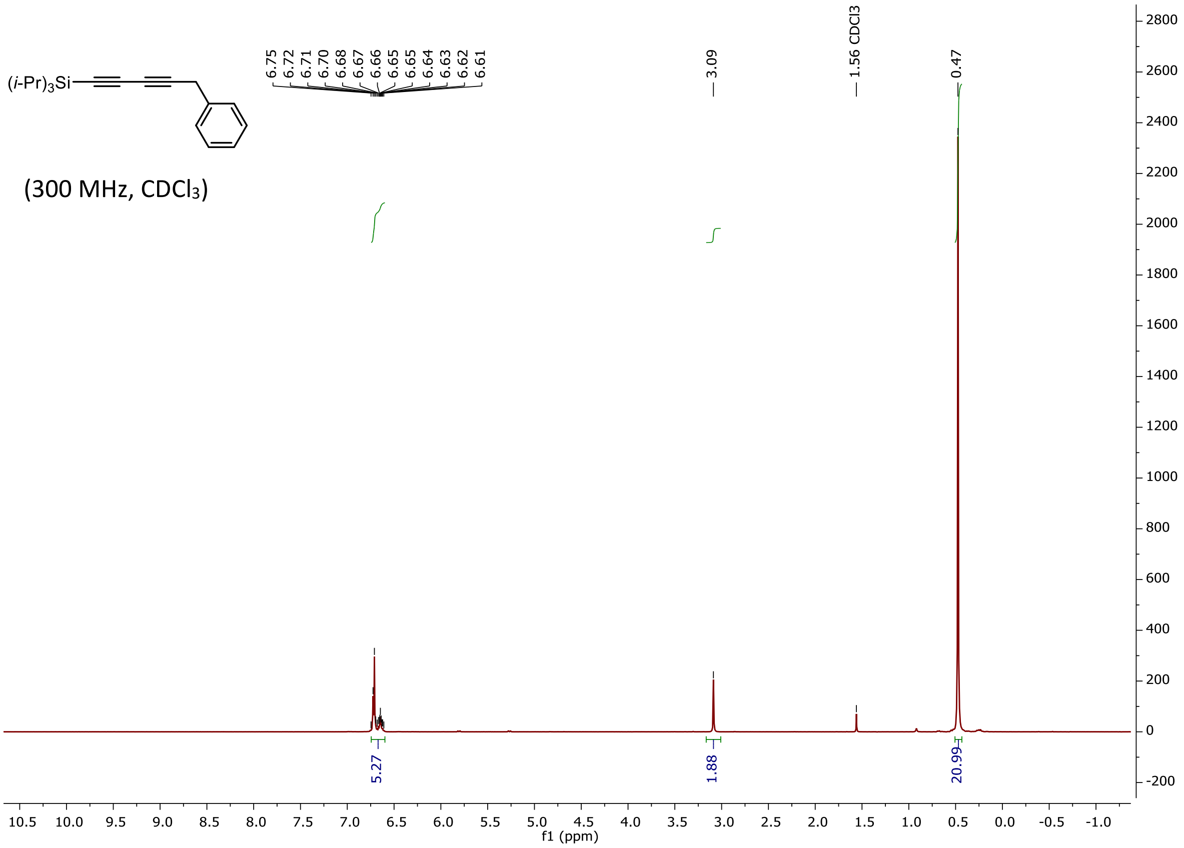


**Figure S43**. $^{1}$H NMR spectrum of **2s**.

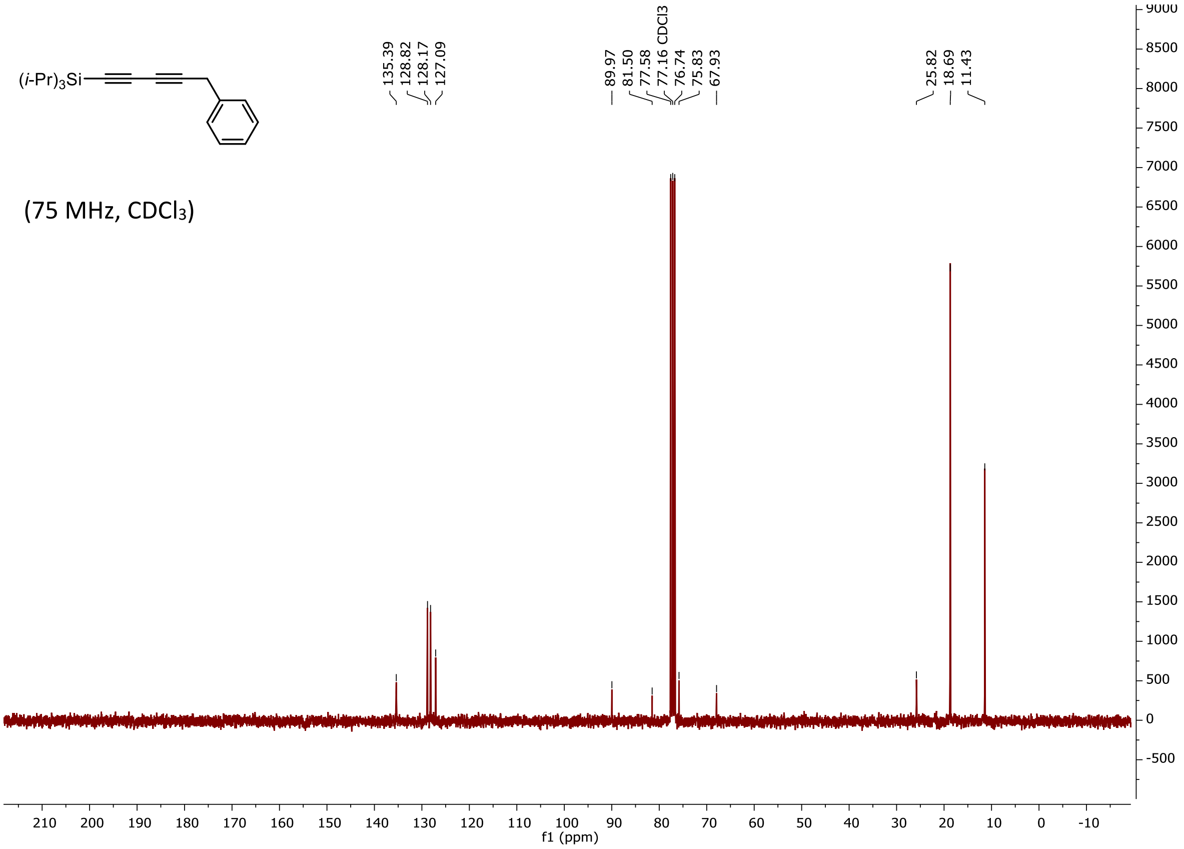


**Figure S44**. $^{13}$C NMR spectrum of **2s**.

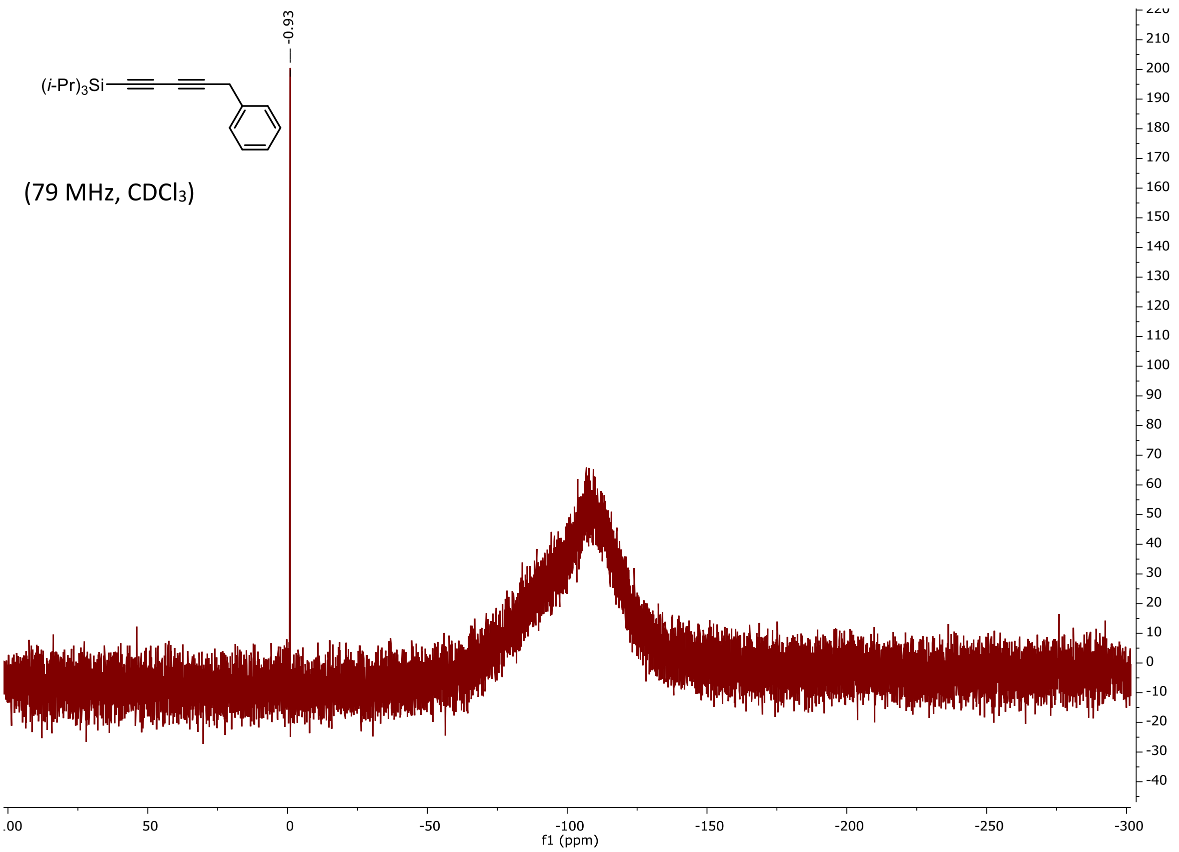


**Figure S45**. ²⁹SiNMR spectrum of **2s**.

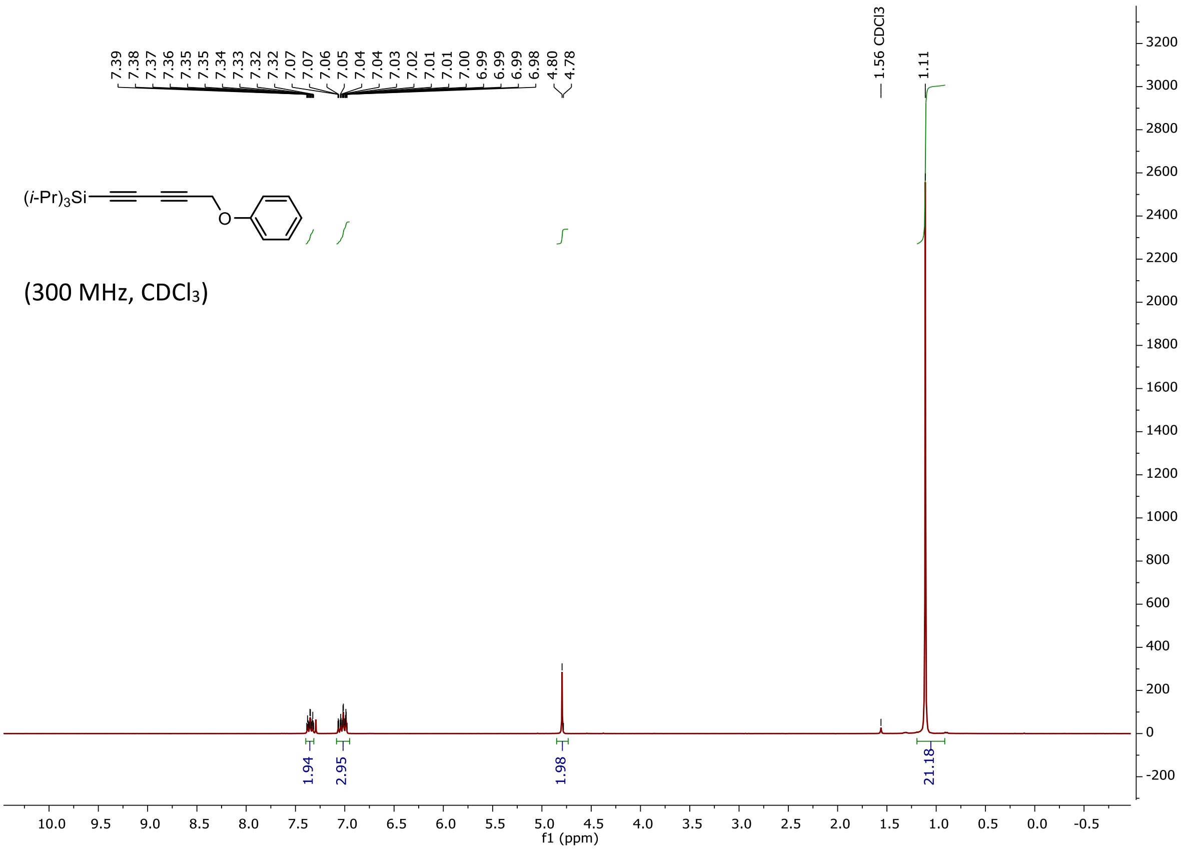


**Figure S46**. ¹H NMR spectrum of **2t**.

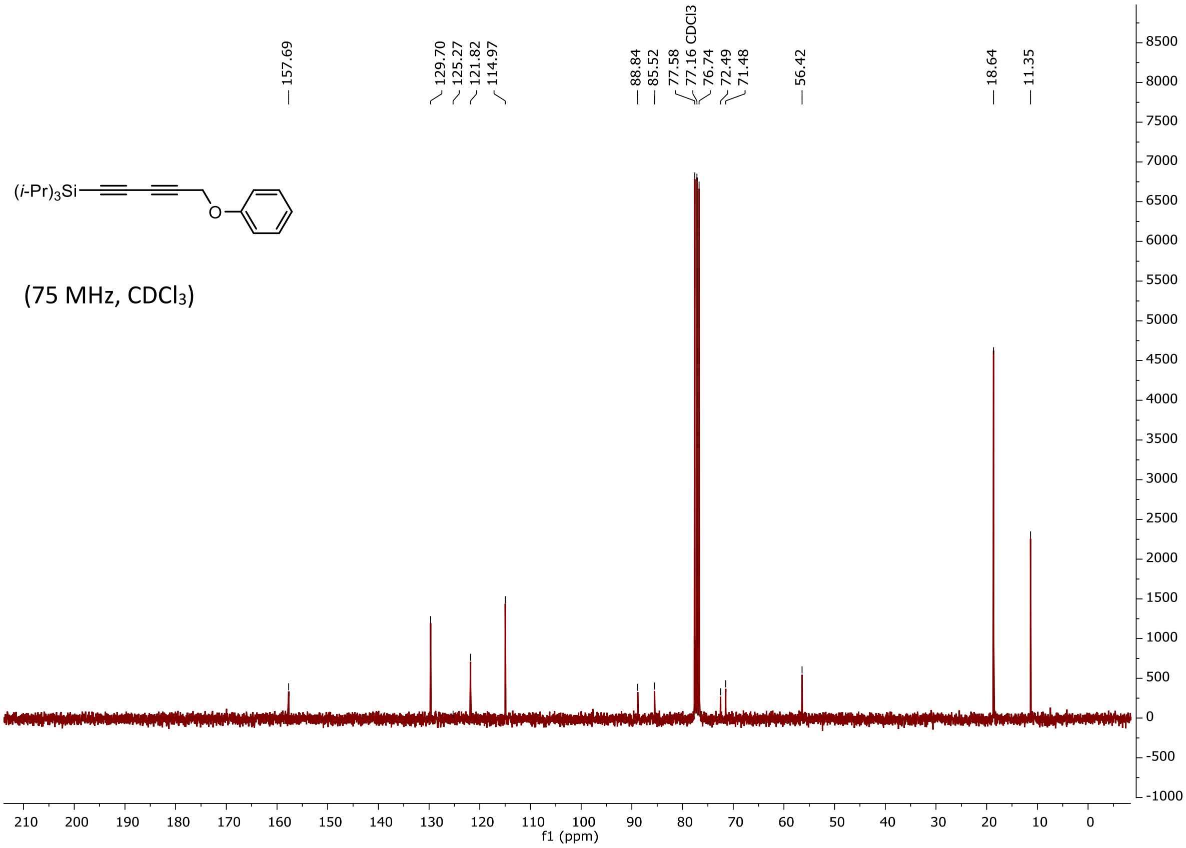


**Figure S47**. $^{13}$C NMR spectrum of **2t**.

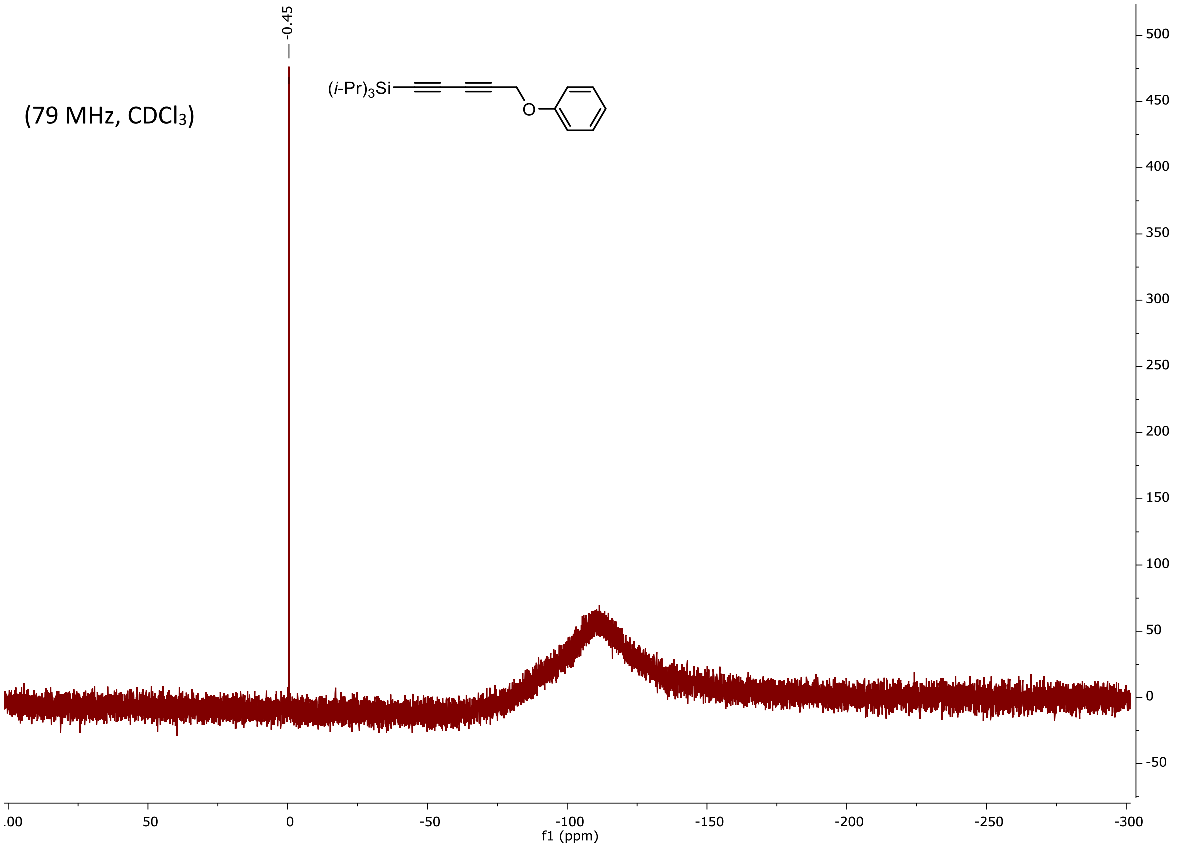


**Figure S48**. $^{29}$Si NMR spectrum of **2t**.

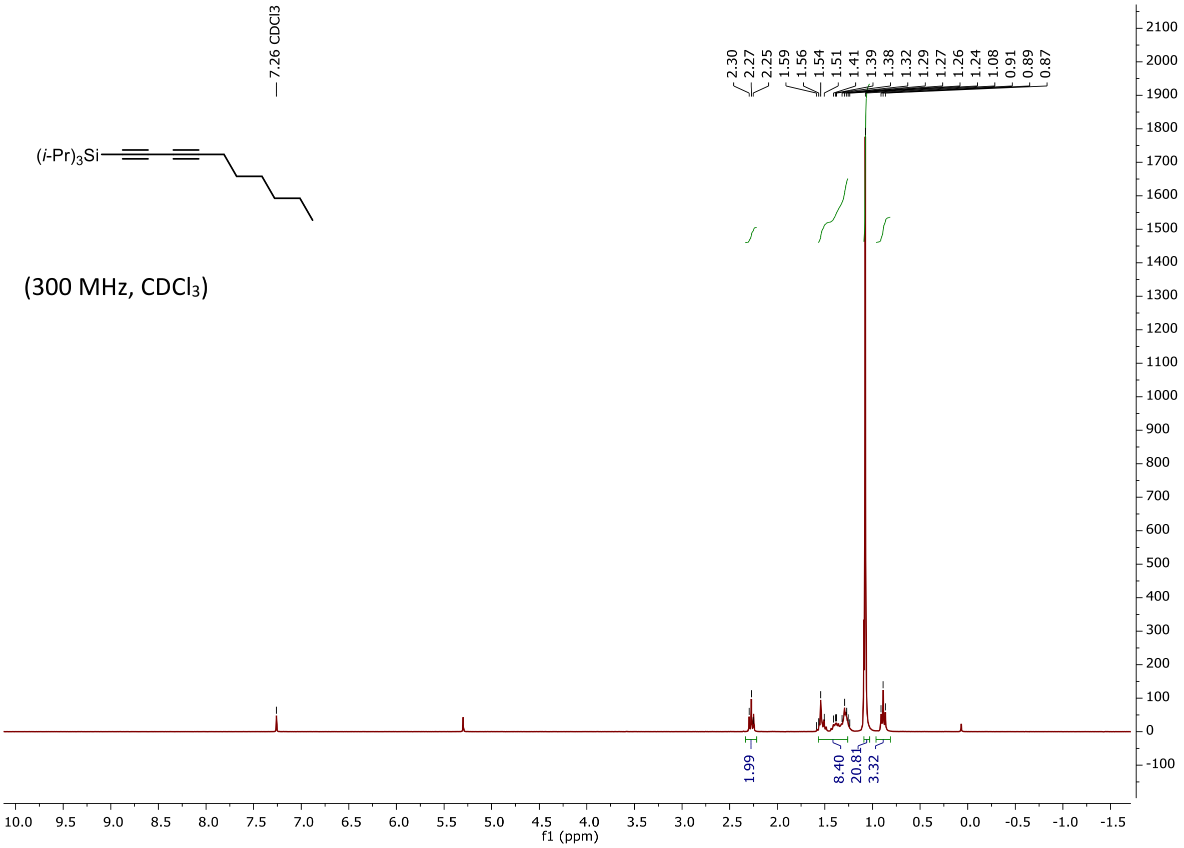


**Figure S49**. $^{1}$H NMR spectrum of **2u**.

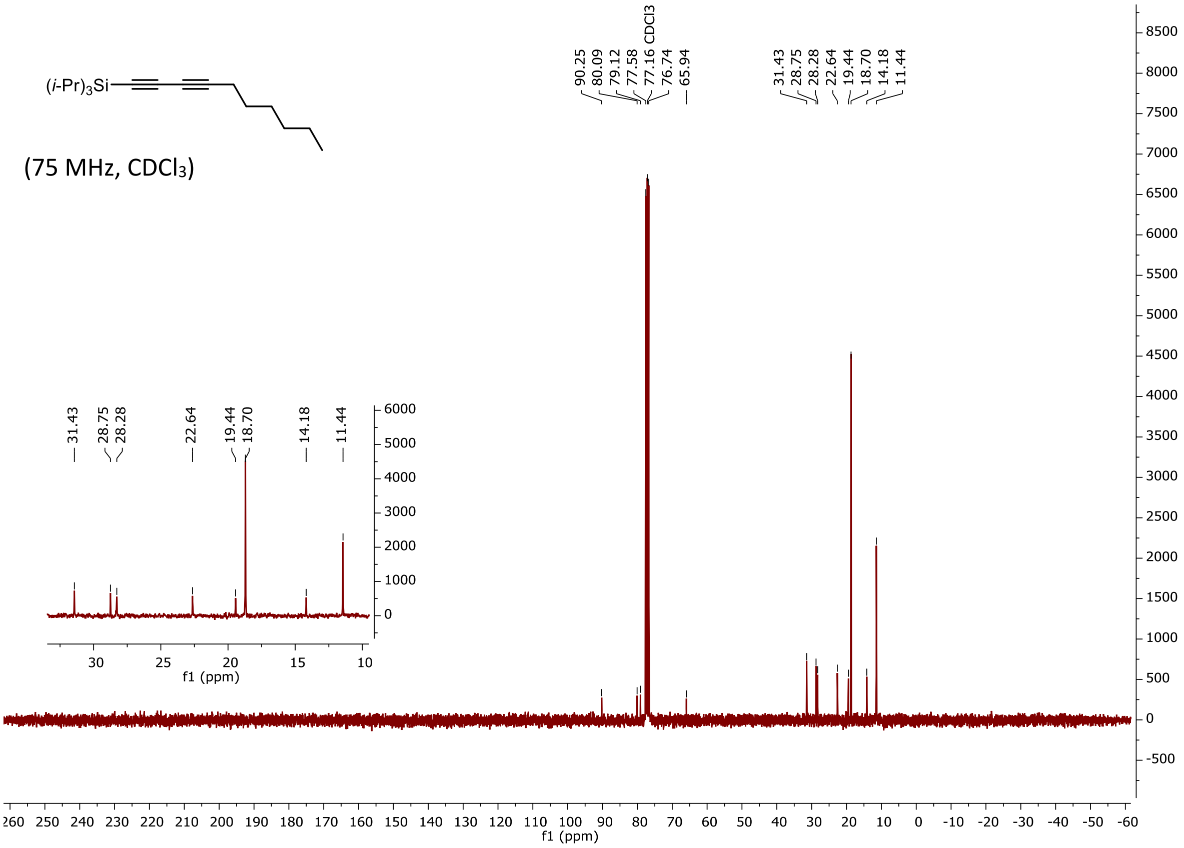


**Figure S50**. $^{13}$C NMR spectrum of **2u**.

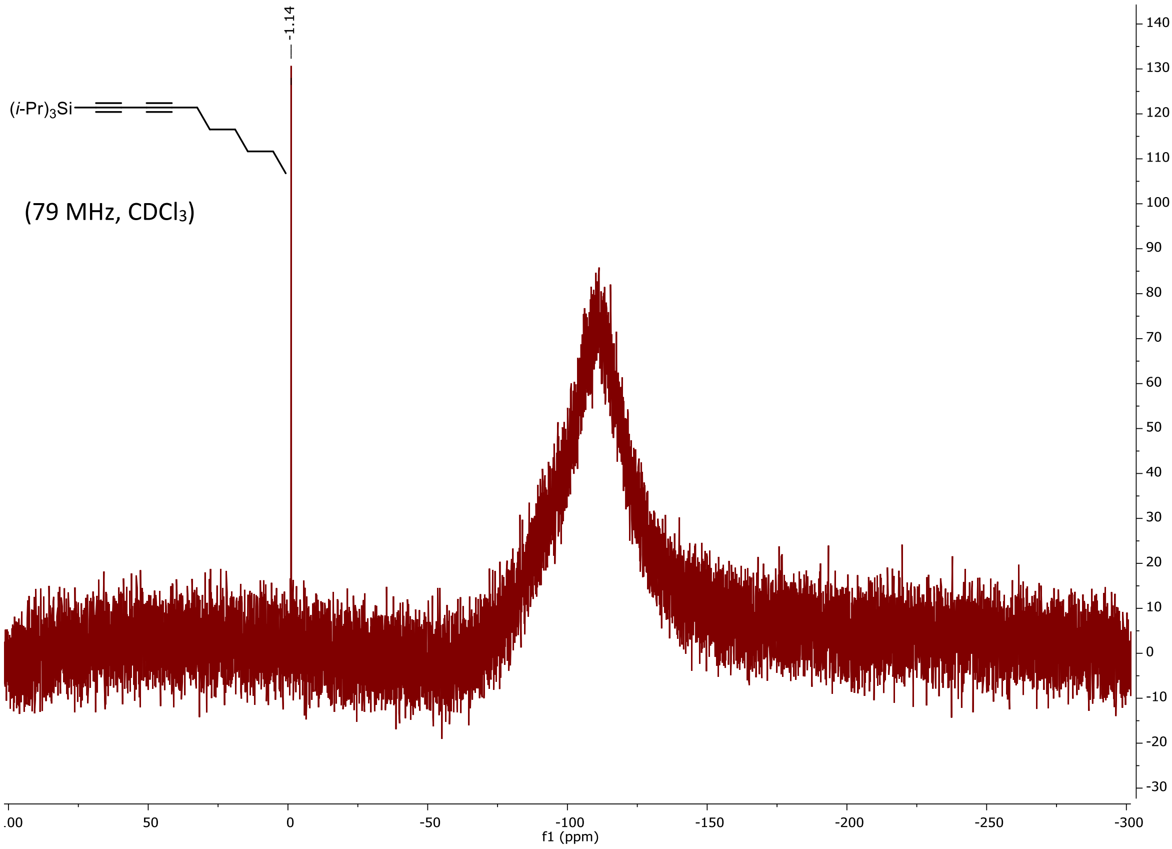


**Figure S51**. $^{29}$Si NMR spectrum of **2u**.

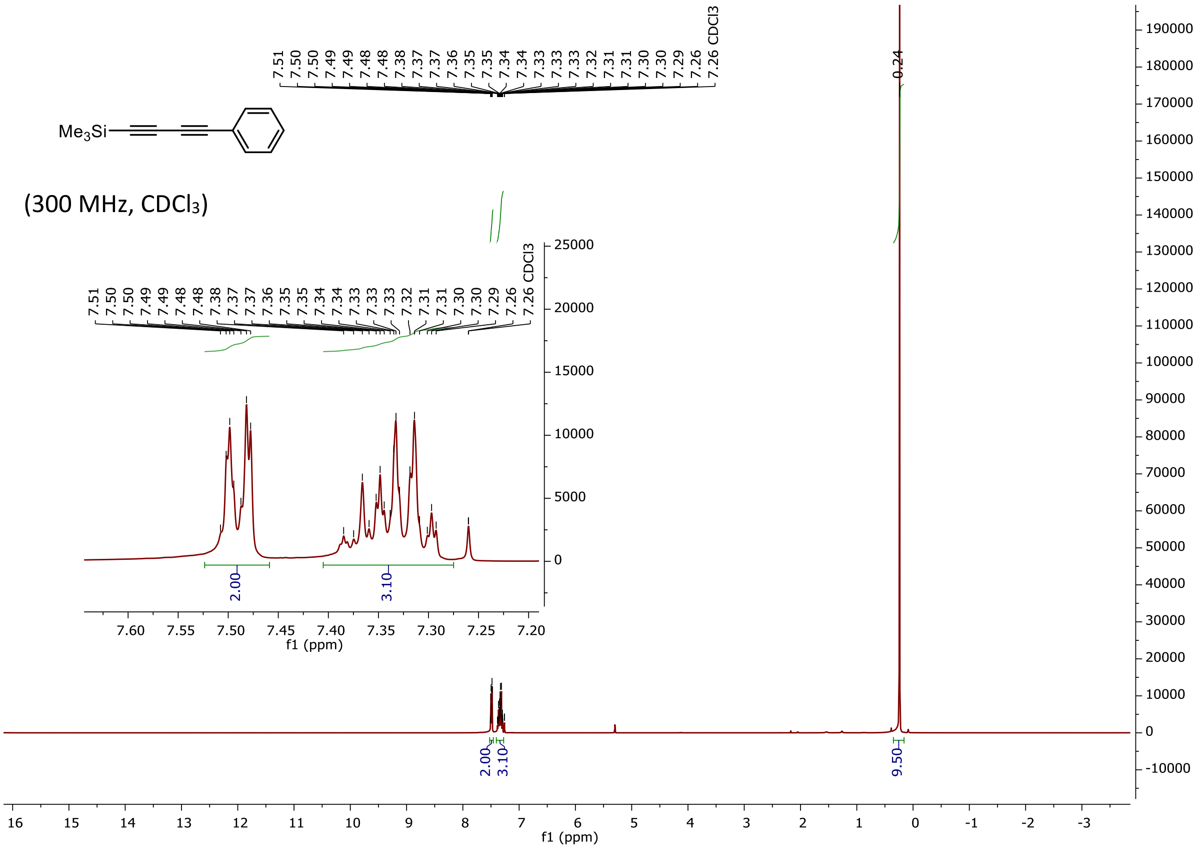


**Figure S52**. $^{1}$H NMR spectrum of **2v**.

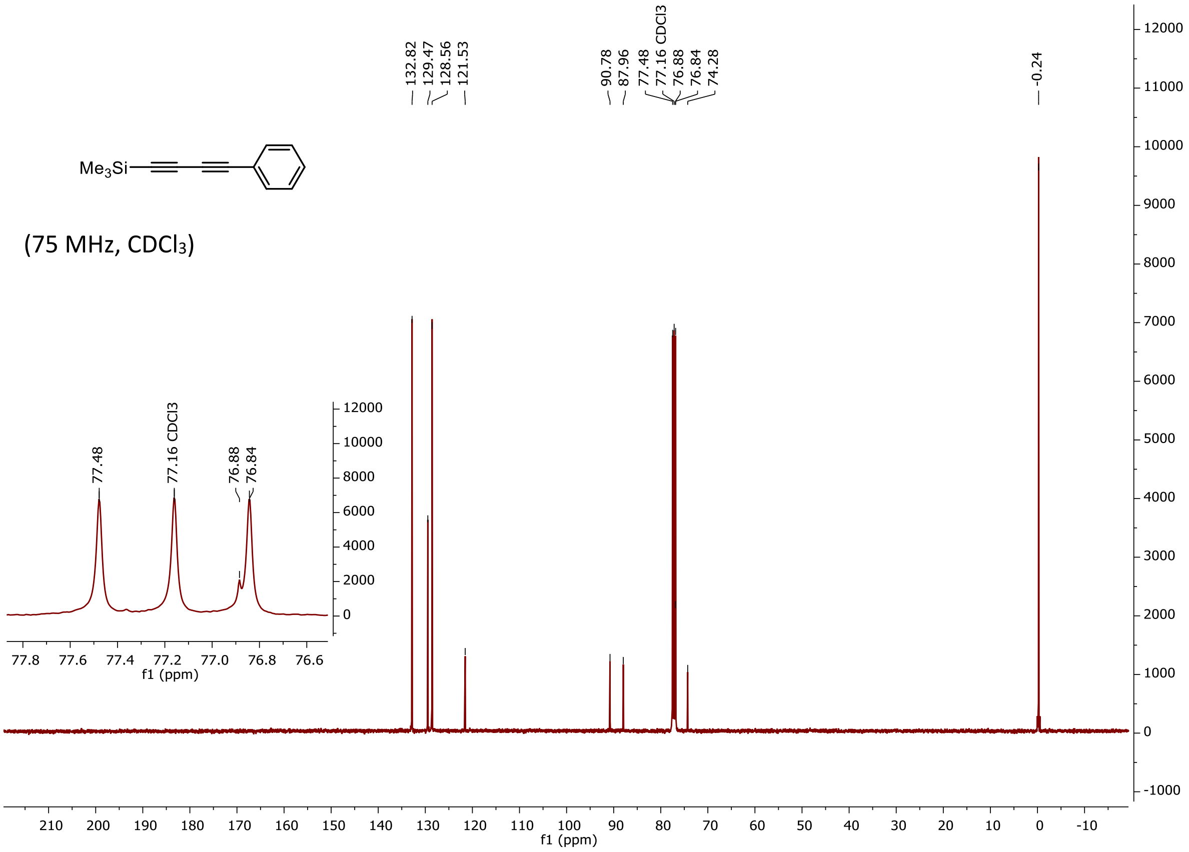


**Figure S53**. $^{13}$C NMR spectrum of **2v**.

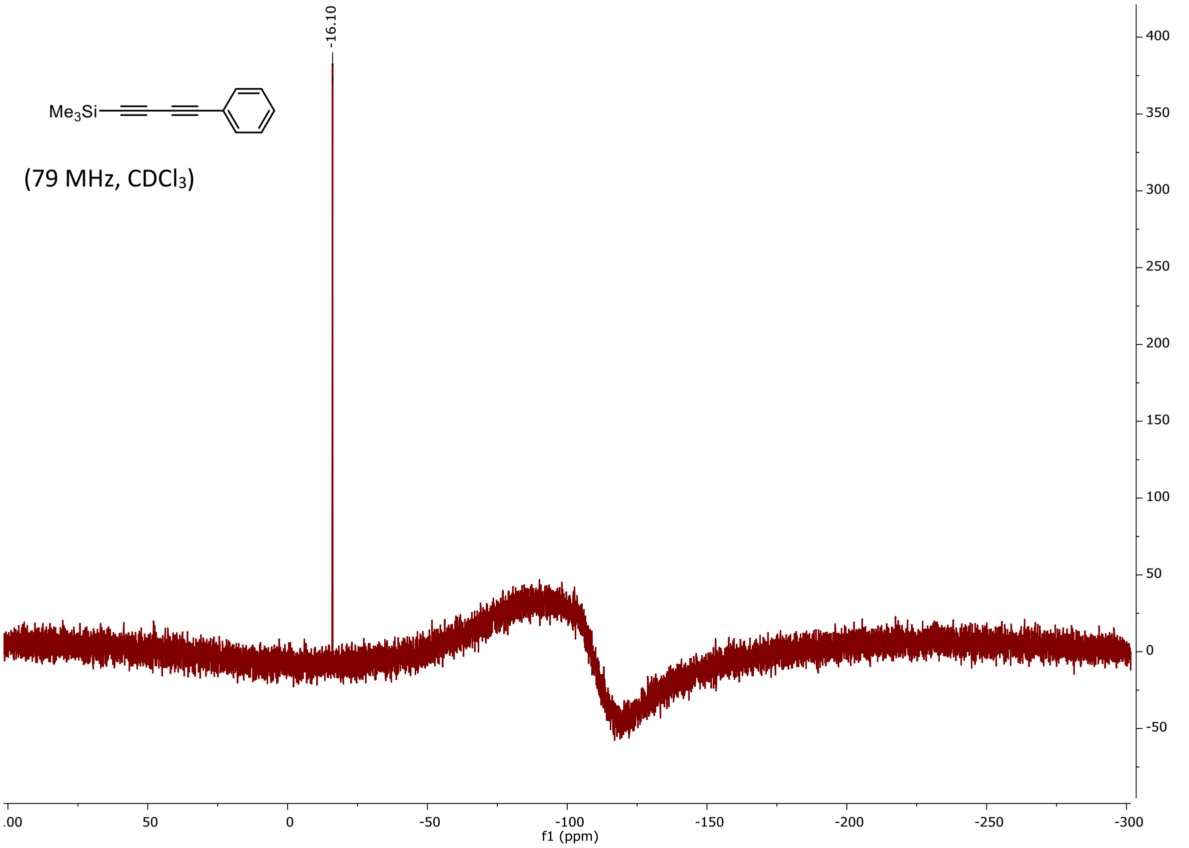


**Figure S54**. $^{29}$Si NMR spectrum of **2v**.

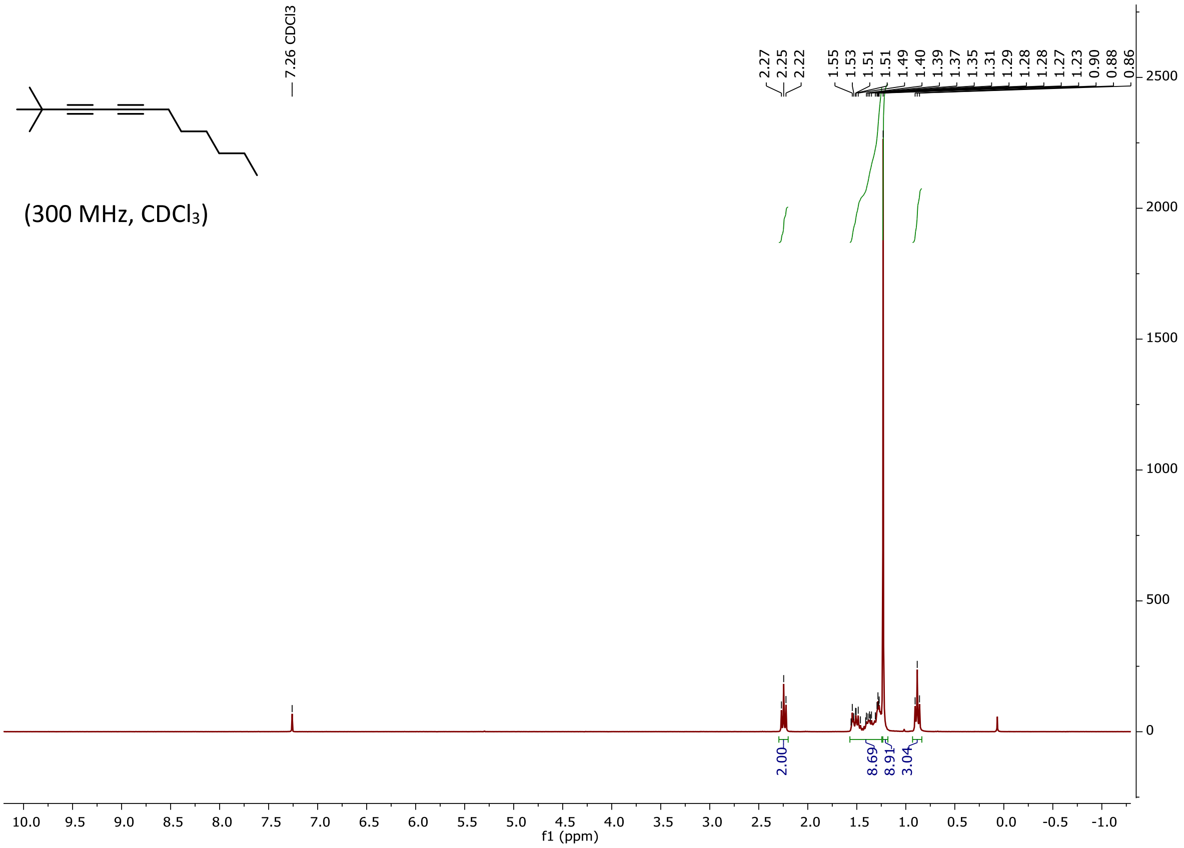


**Figure S55**. $^{1}$H NMR spectrum of **2w**.

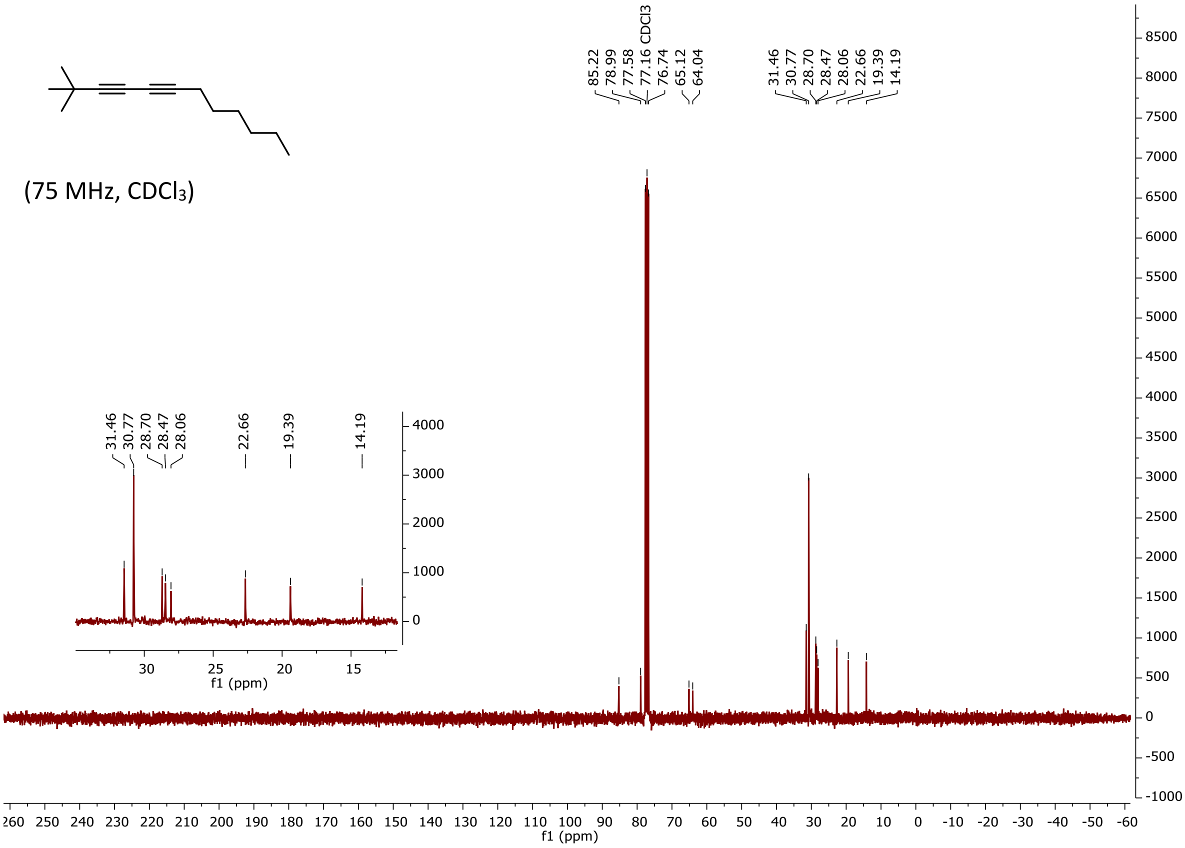


**Figure S56**. $^{13}$C NMR spectrum of **2w**.

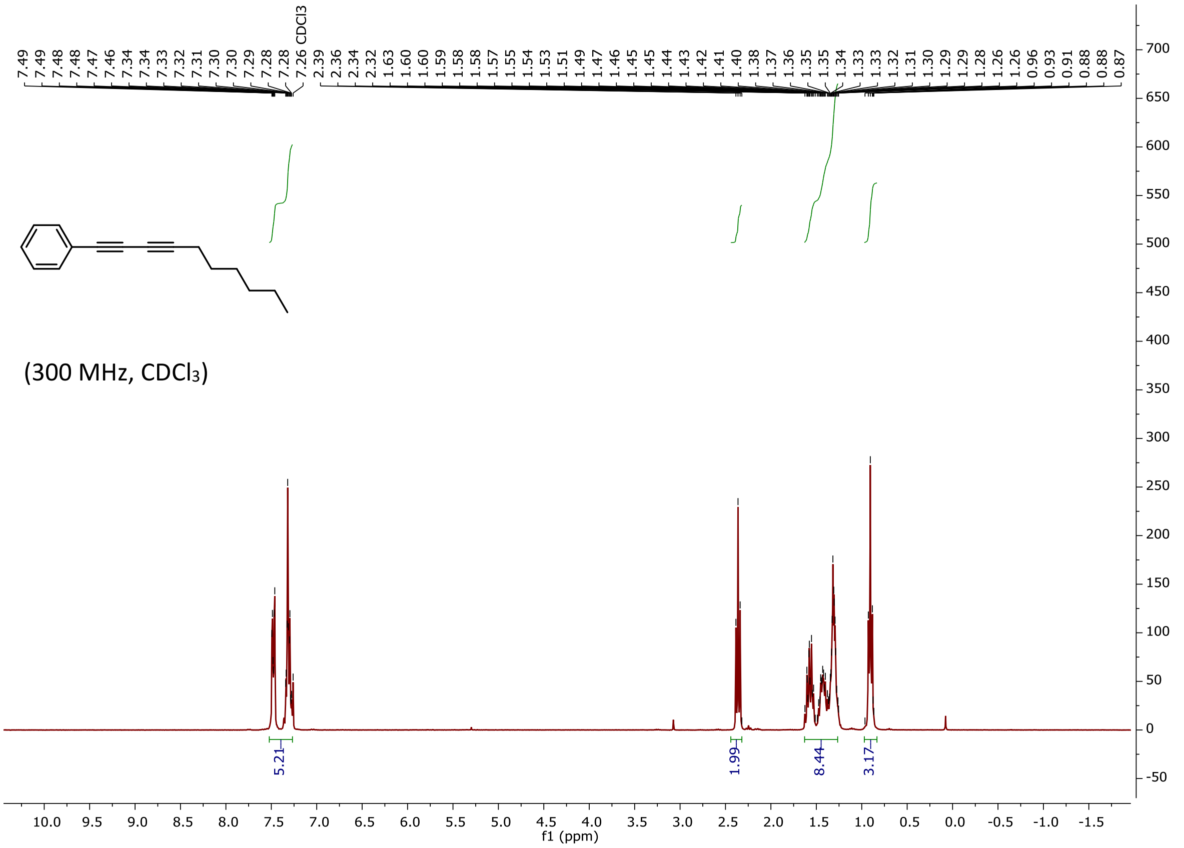


**Figure S57**. $^{1}$H NMR spectrum of **2x**.

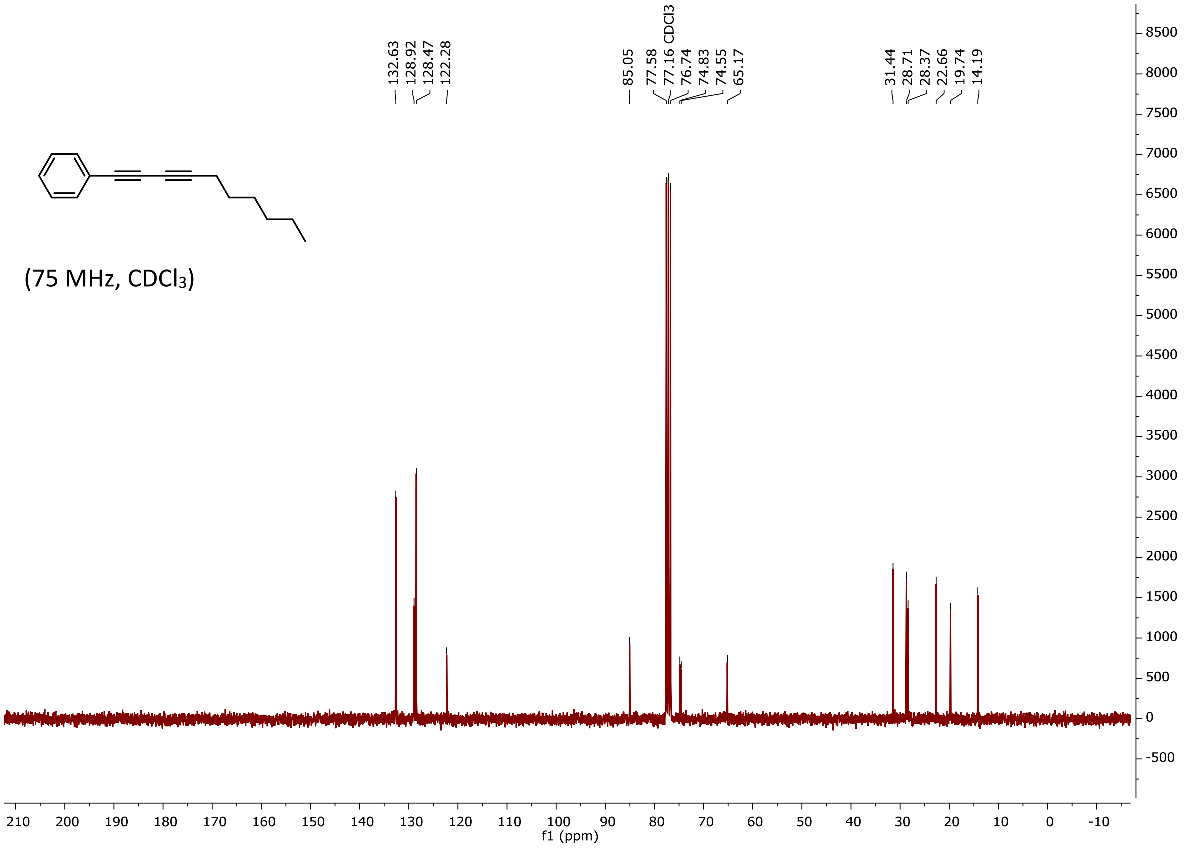


**Figure S58**. $^{13}$C NMR spectrum of **2x**.

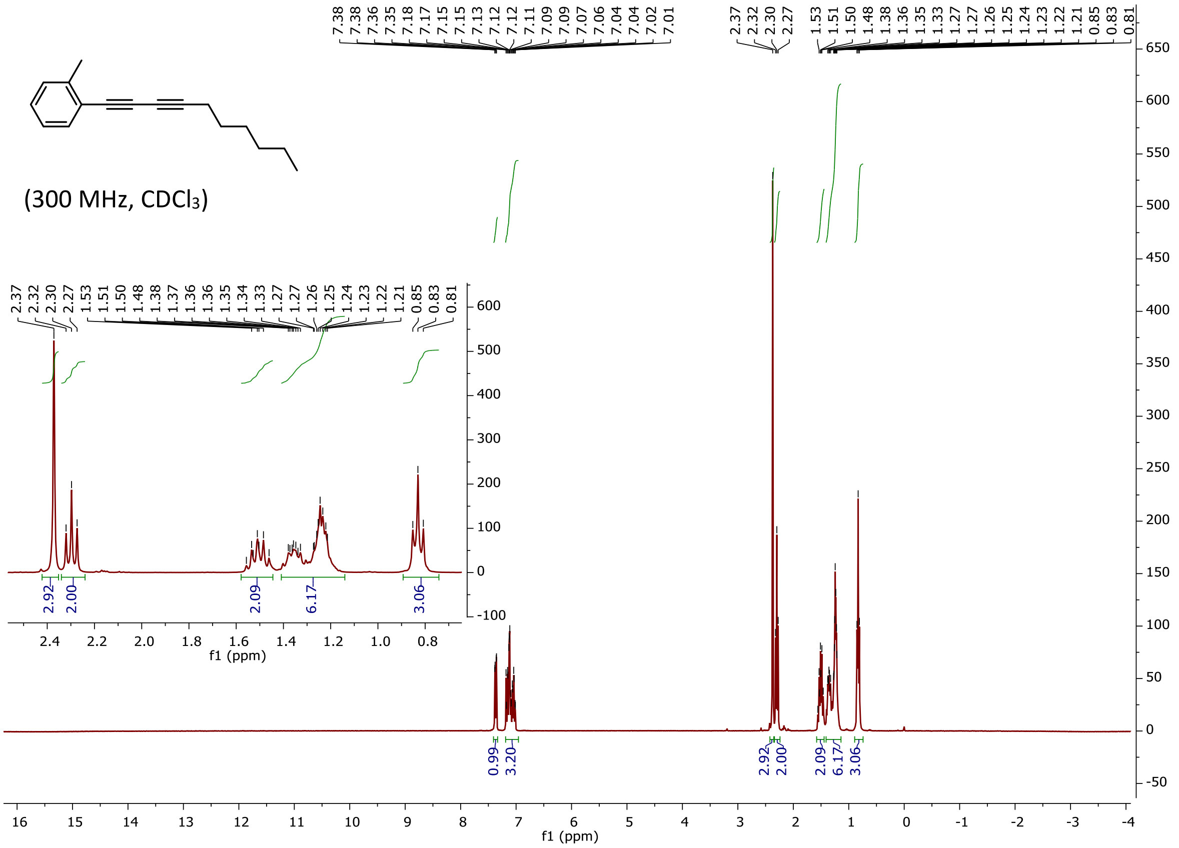


**Figure S59**. $^{1}$H NMR spectrum of **2y**.

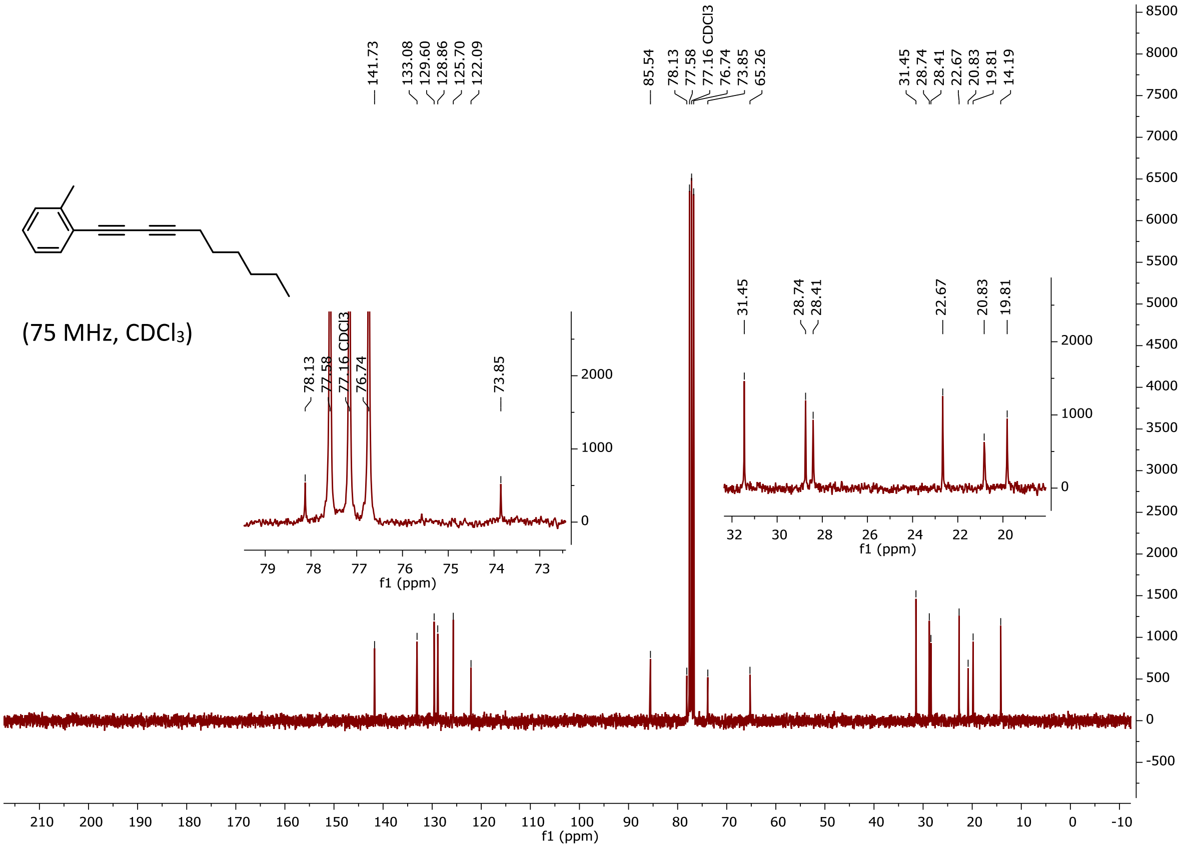


**Figure S60**. $^{13}$C NMR spectrum of **2y**.

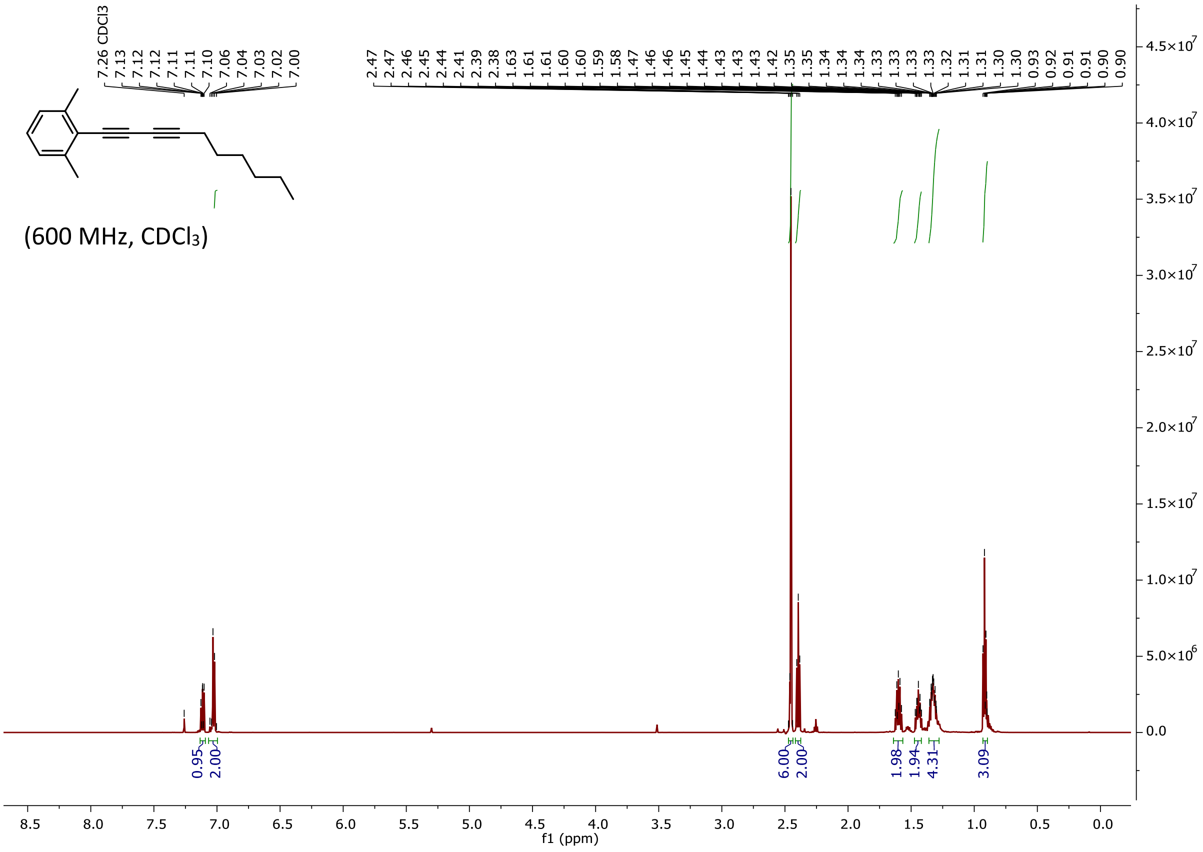


**Figure S61**. $^{1}$H NMR spectrum of **2z**.

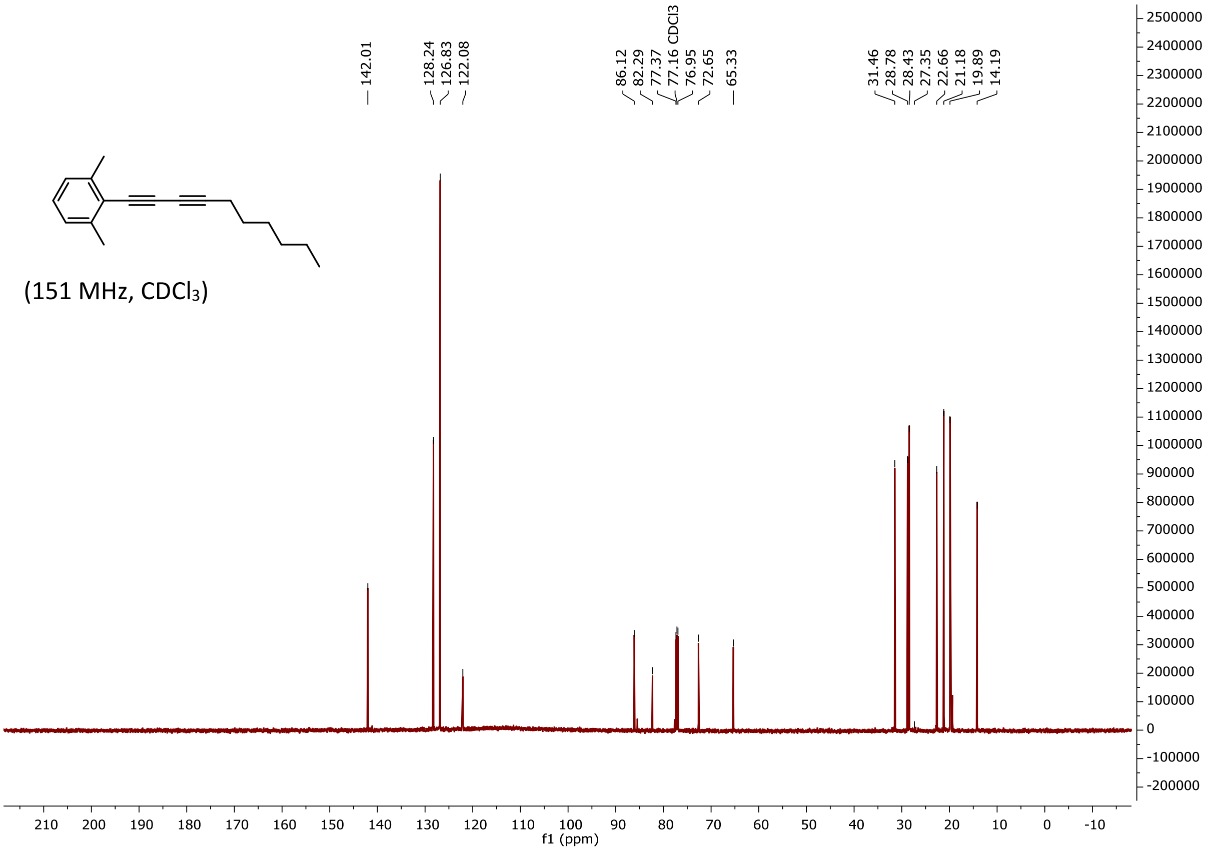


**Figure S62**. $^{13}$C NMR spectrum of **2z**.

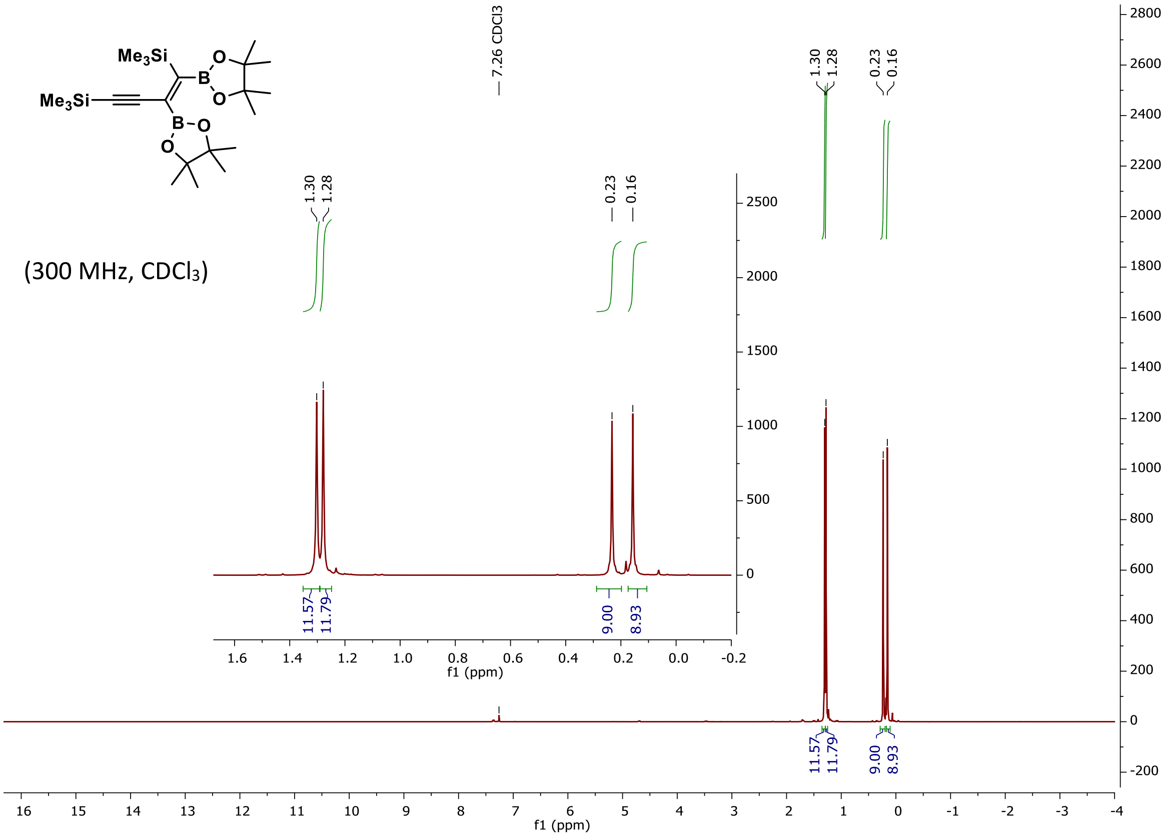


**Figure S63**. $^{1}$H NMR spectrum of **3a**.

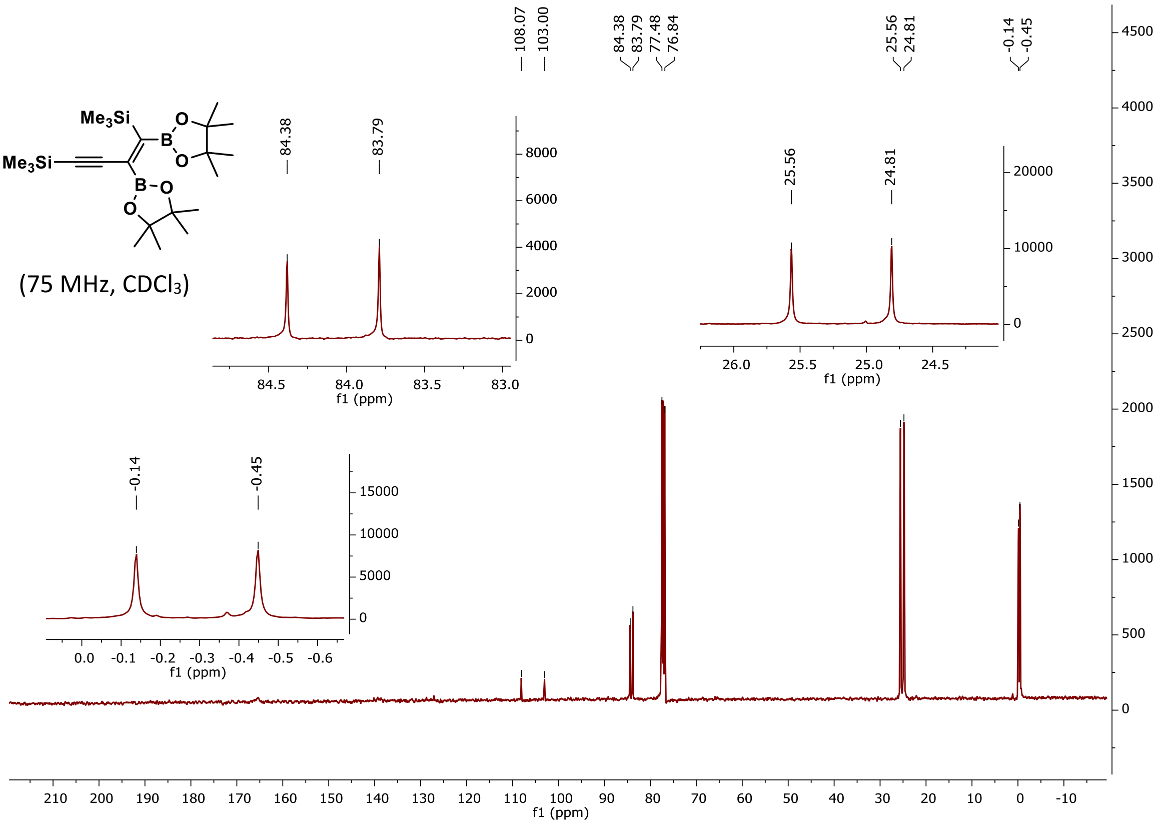


**Figure S64**. $^{13}$C NMR spectrum of **3a**.

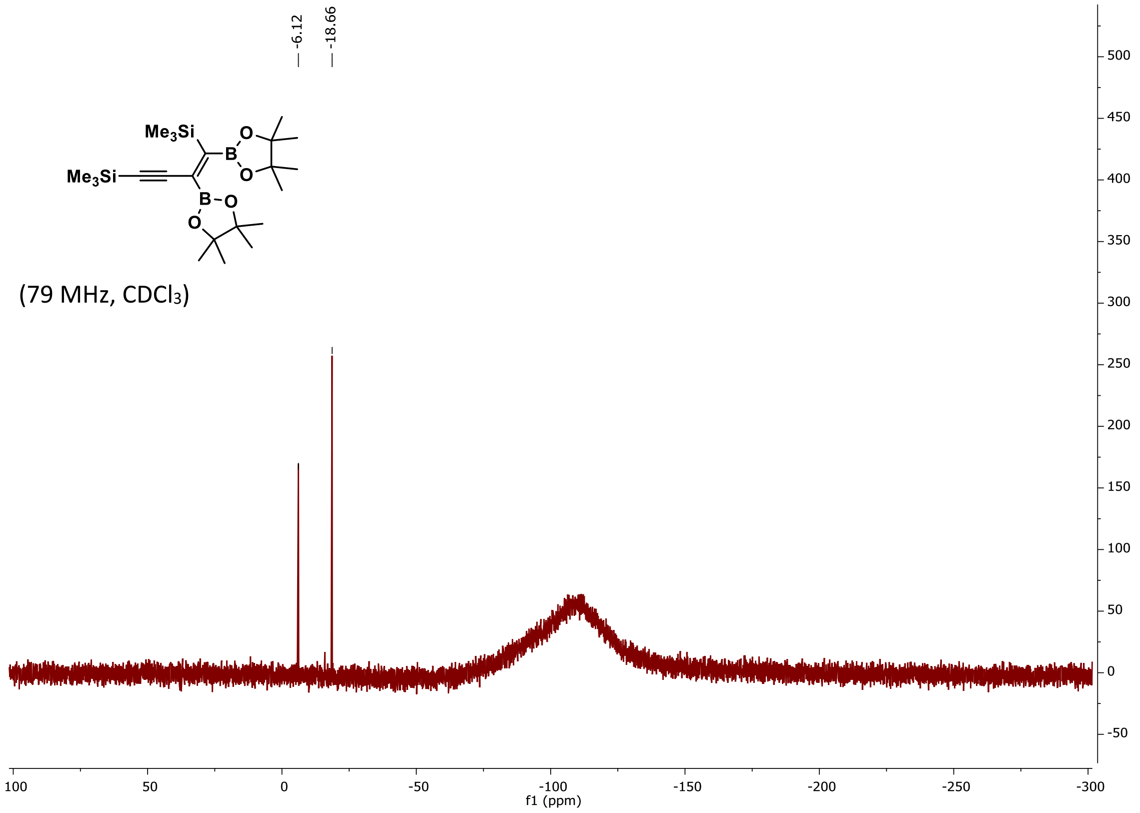


**Figure S65**. $^{29}$Si NMR spectrum of **3a**.

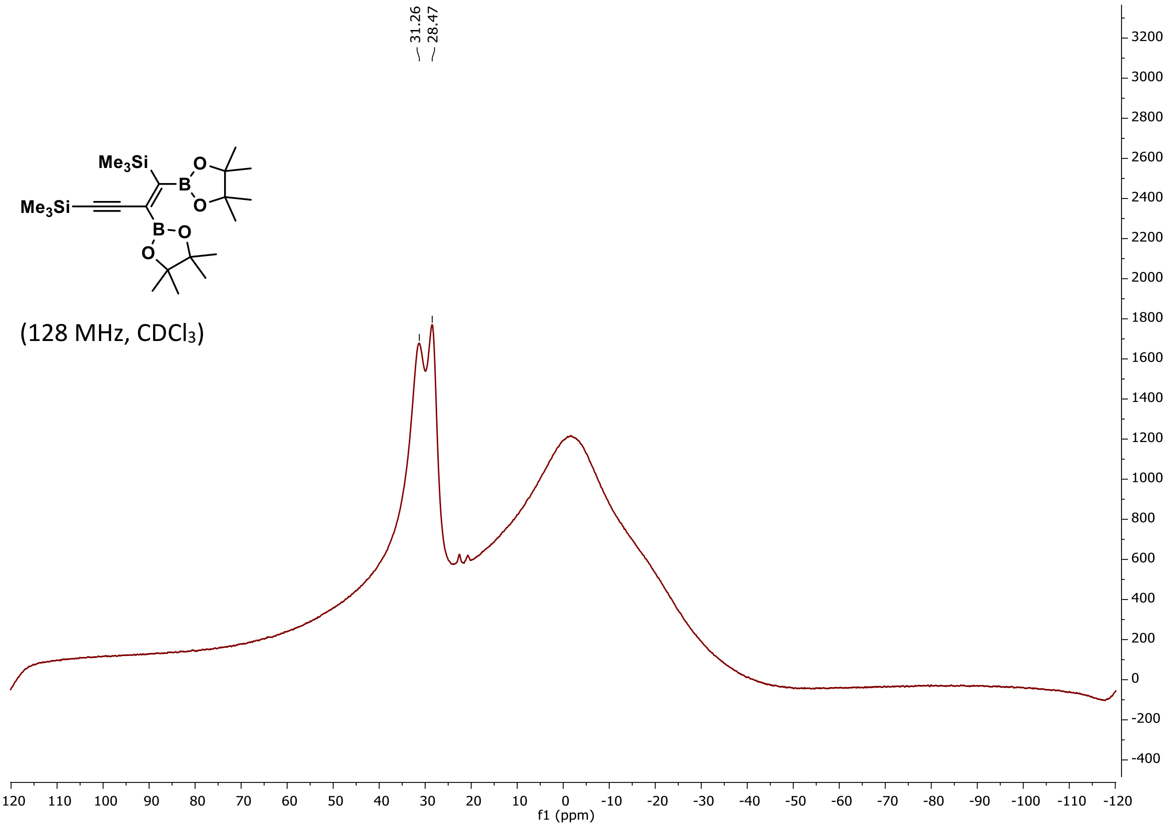


**Figure S66**. $^{11}$B NMR spectrum of **3a**.

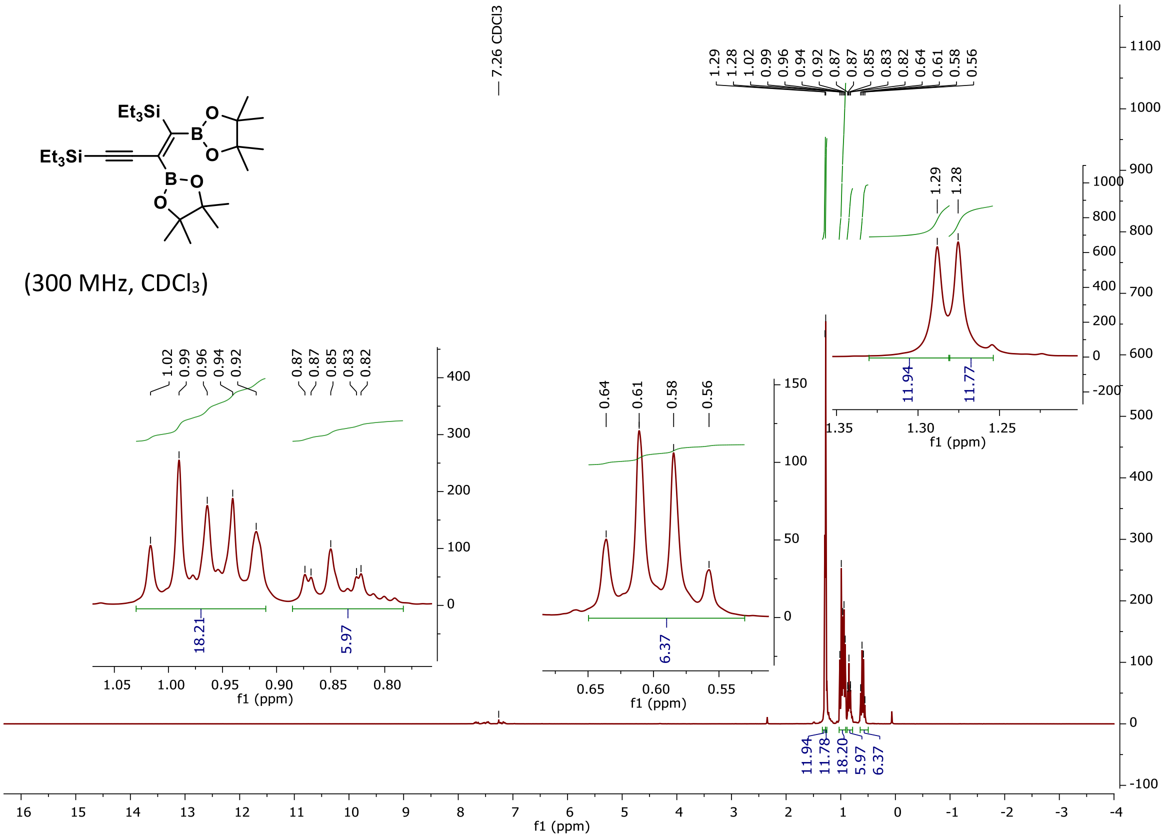


**Figure S67**. $^{1}$H NMR spectrum of **3b**.

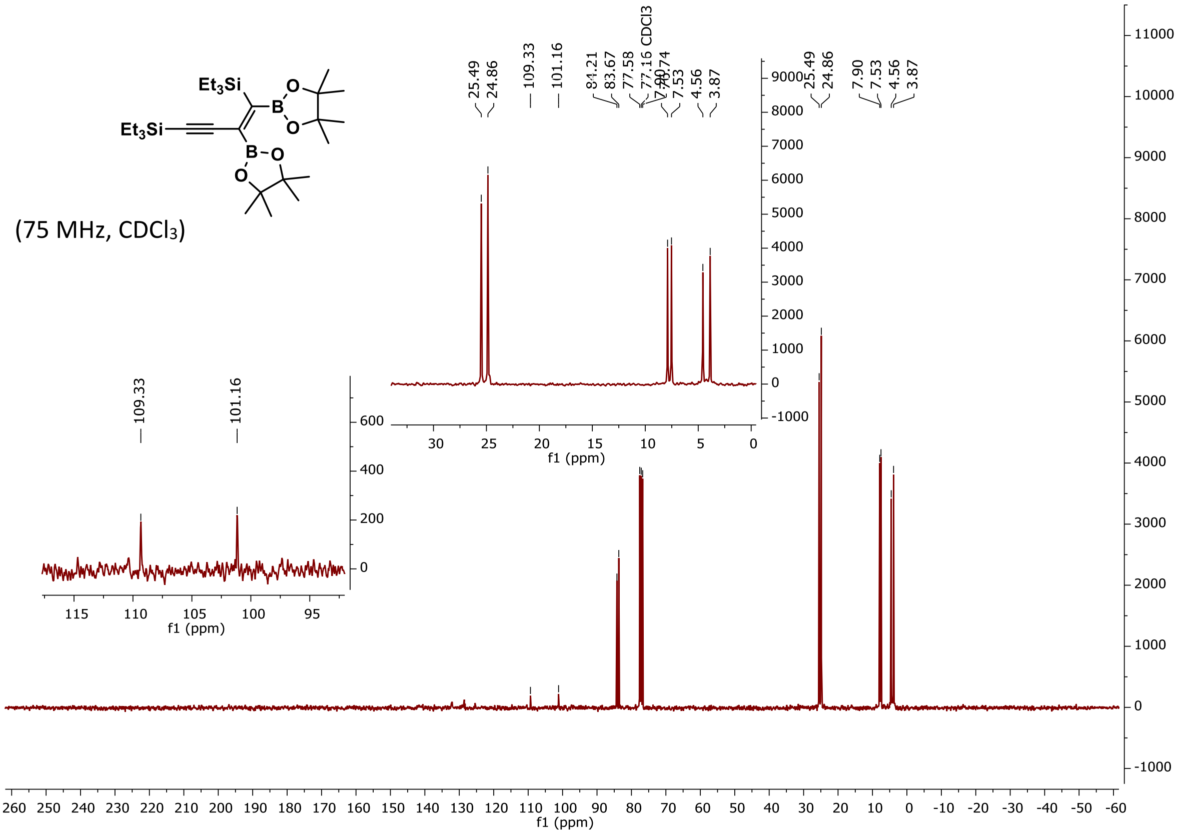


**Figure S68**. $^{13}$C NMR spectrum of **3b**.

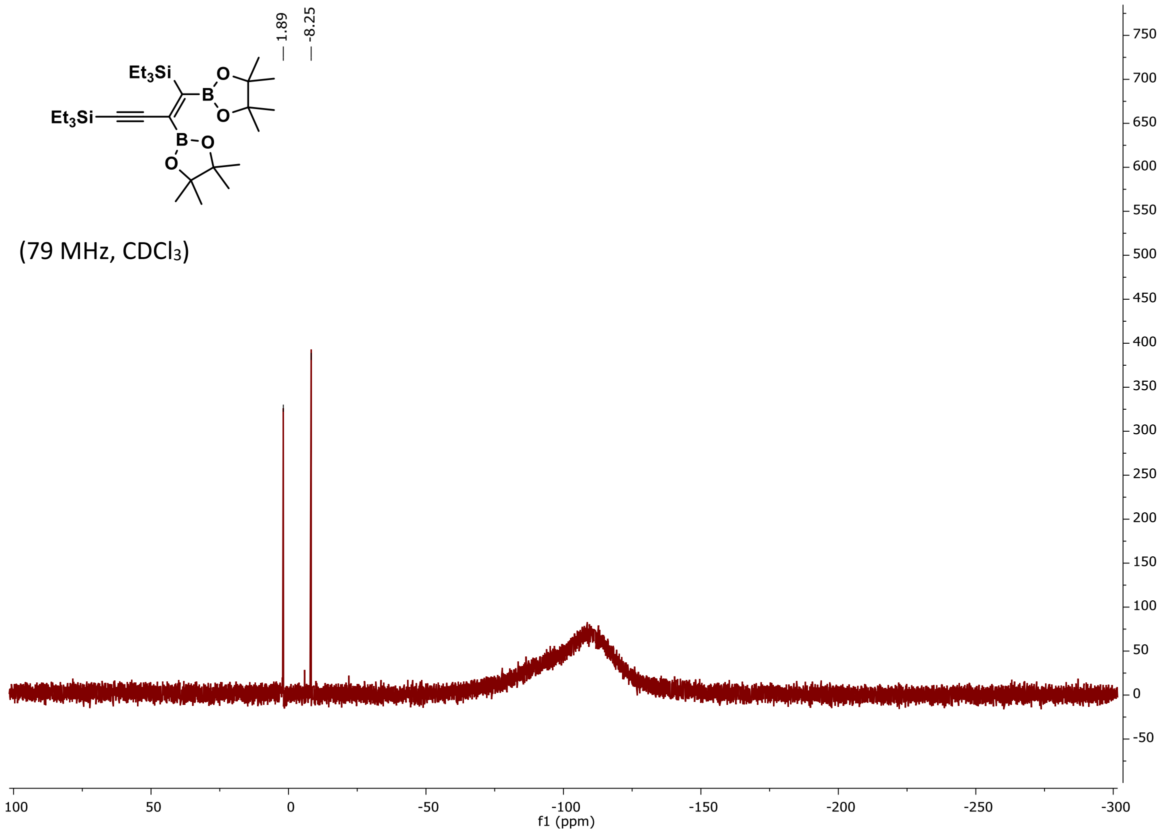


**Figure S69**. $^{29}$Si NMR spectrum of **3b**.

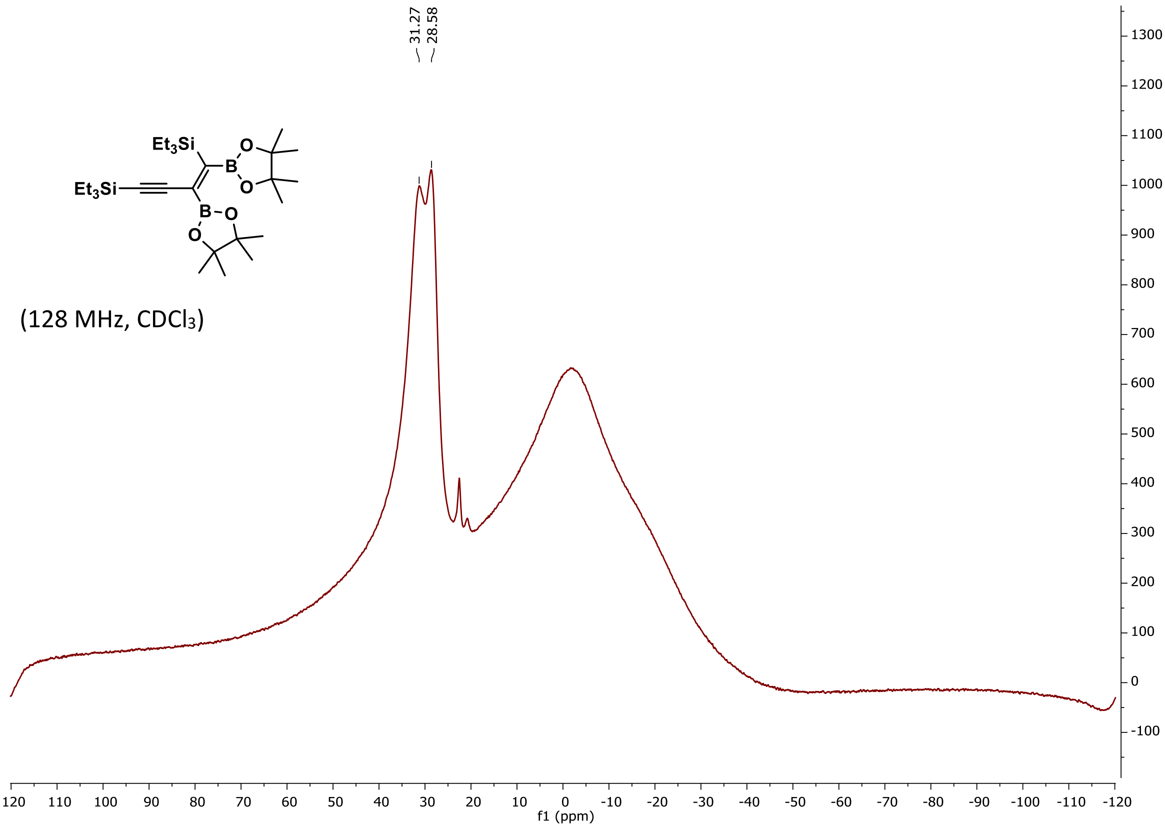


**Figure S70**. $^{11}$B NMR spectrum of **3b**.

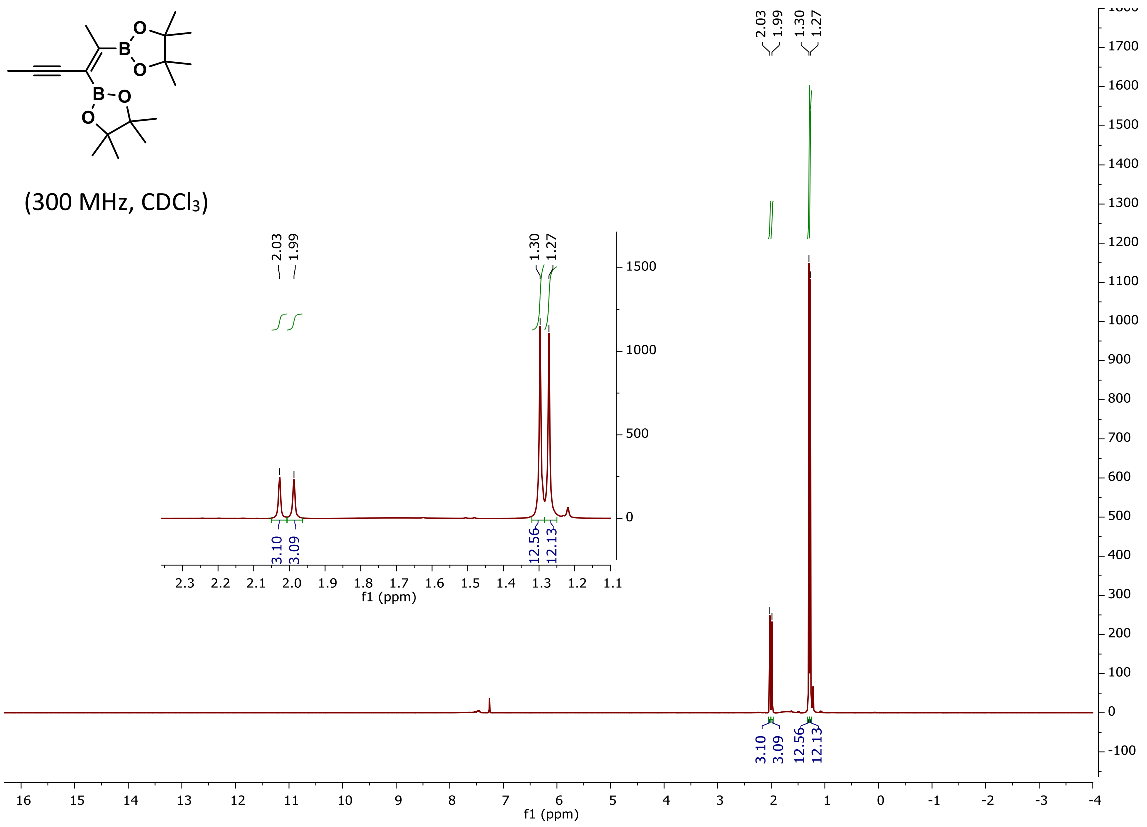


**Figure S71**. $^{1}$H NMR spectrum of **3d**.

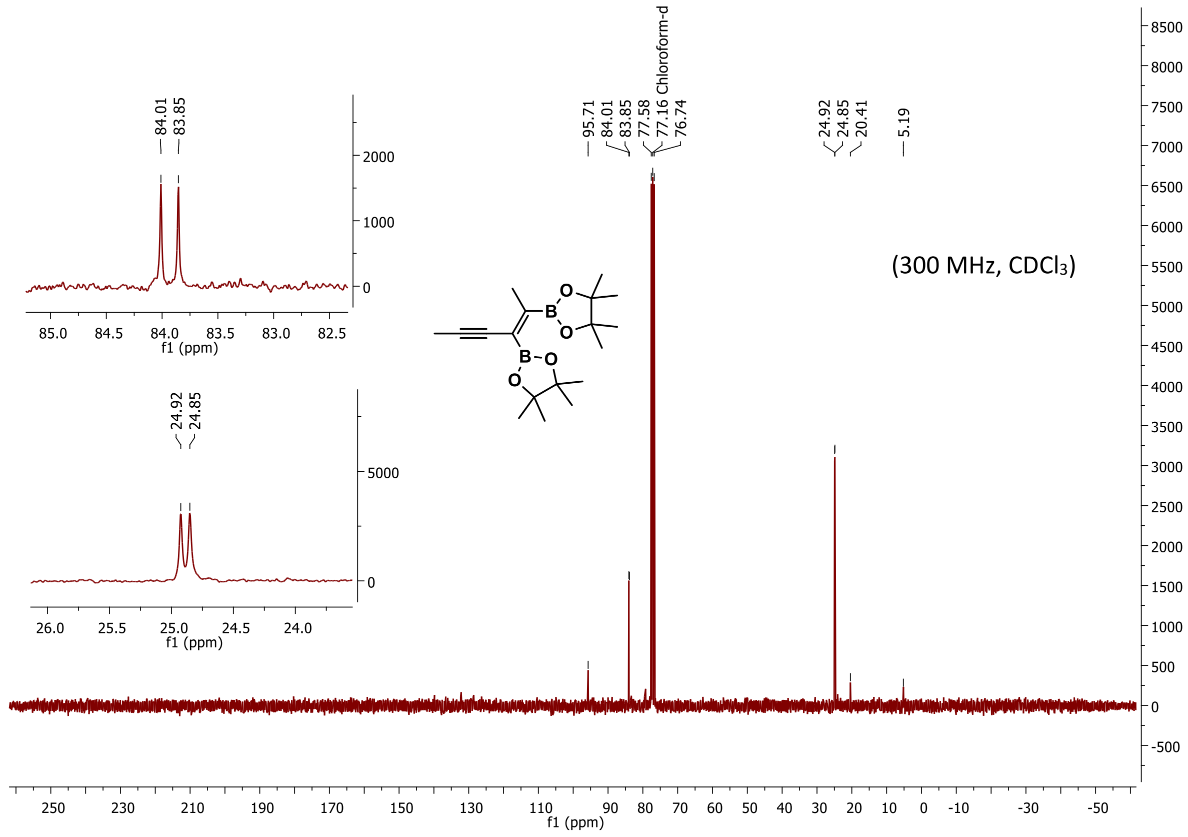


**Figure S72**. $^{13}$C NMR spectrum of **3d**.

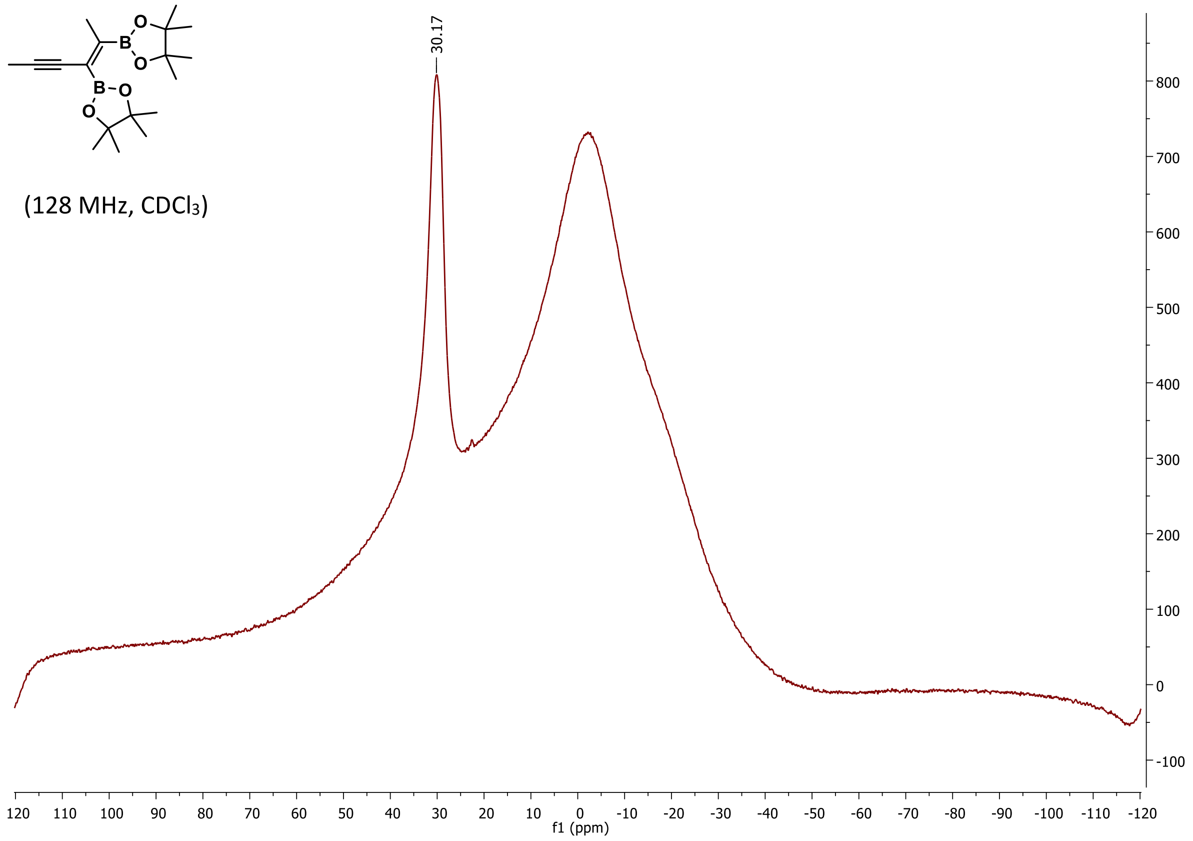


**Figure S73**. $^{11}$B NMR spectrum of **3d**.

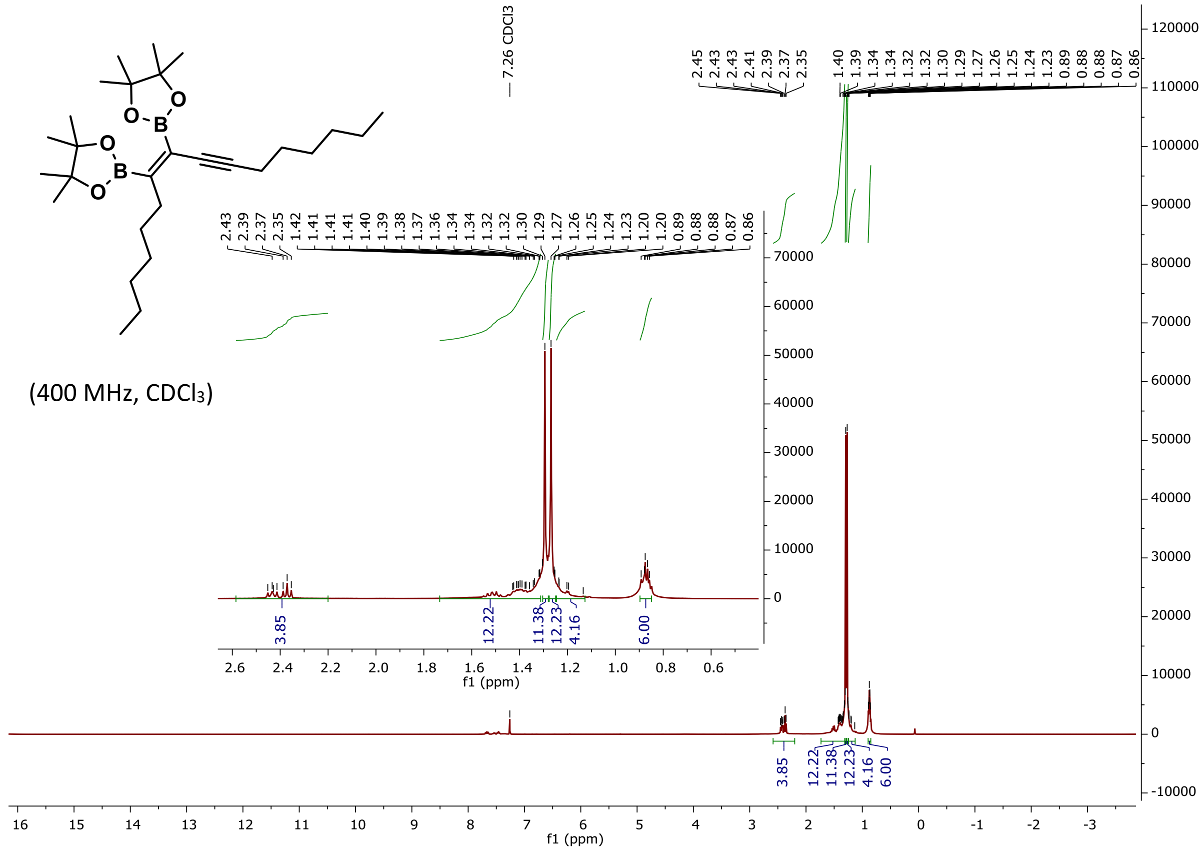


**Figure S74**. $^{1}$H NMR spectrum of **3e**.

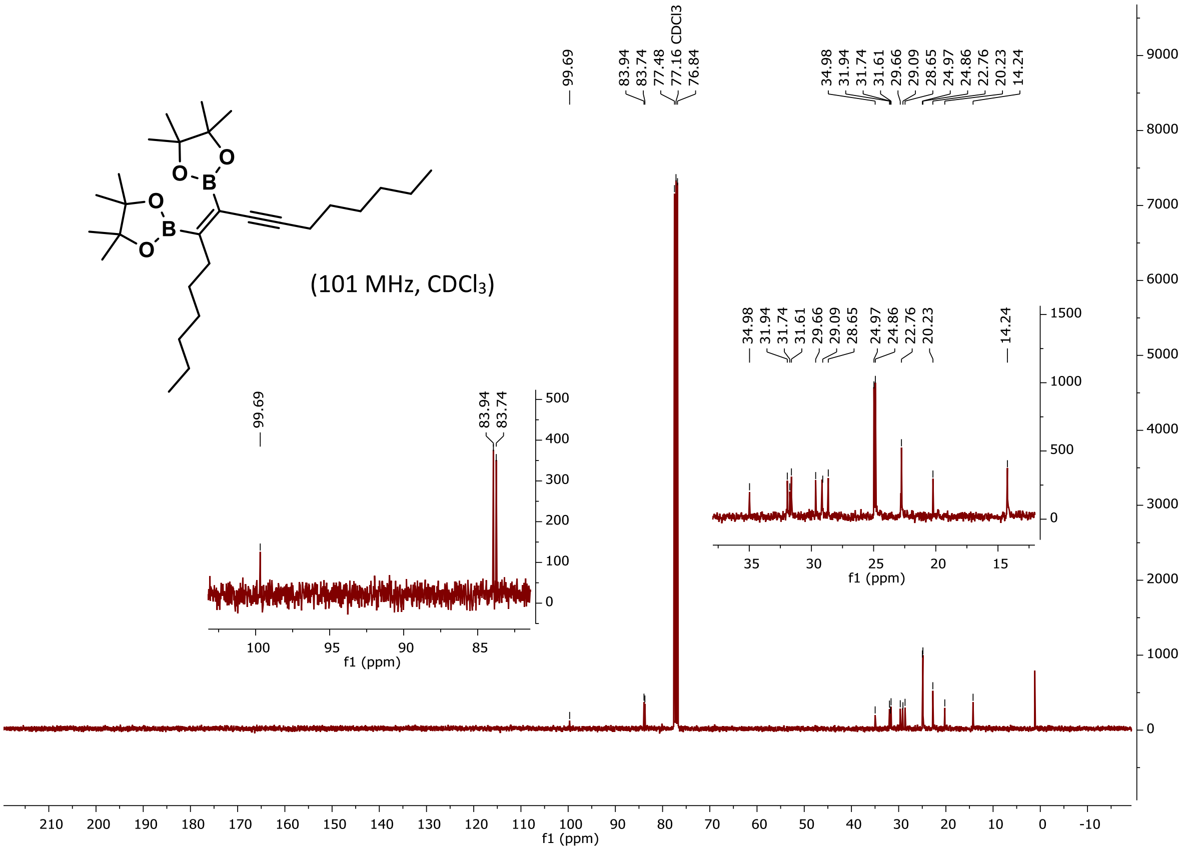


**Figure S75**. $^{13}$C NMR spectrum of **3e.**

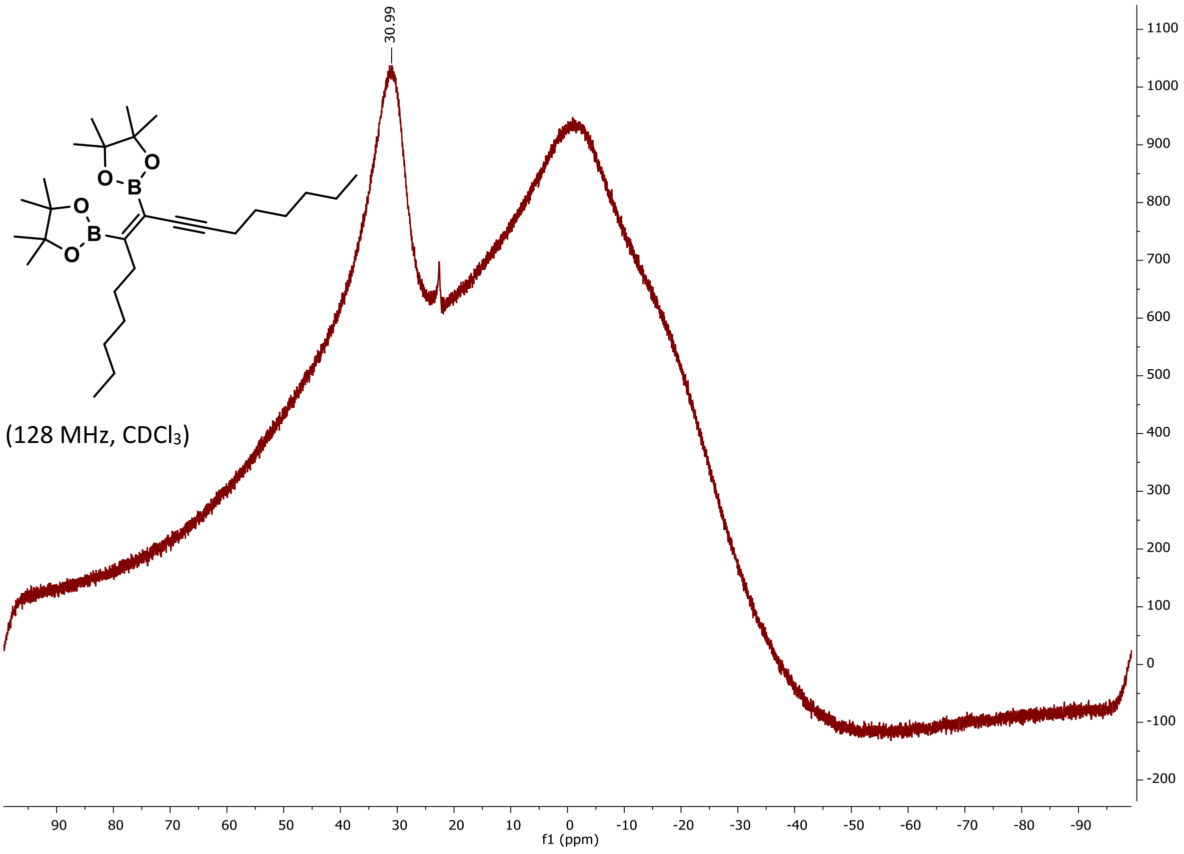


**Figure S76**. $^{13}$C NMR spectrum of **3e.**

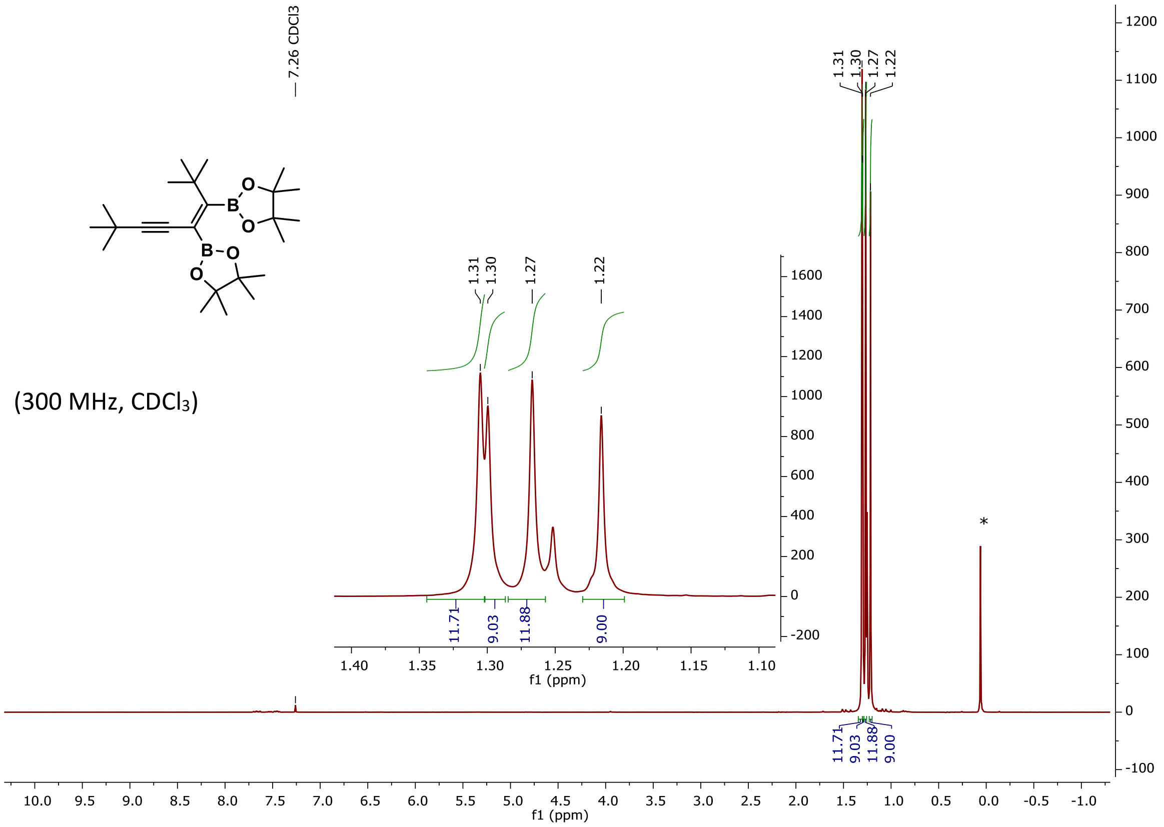


**Figure S77**. $^{1}$H NMR spectrum of **3f**. *-Grease.

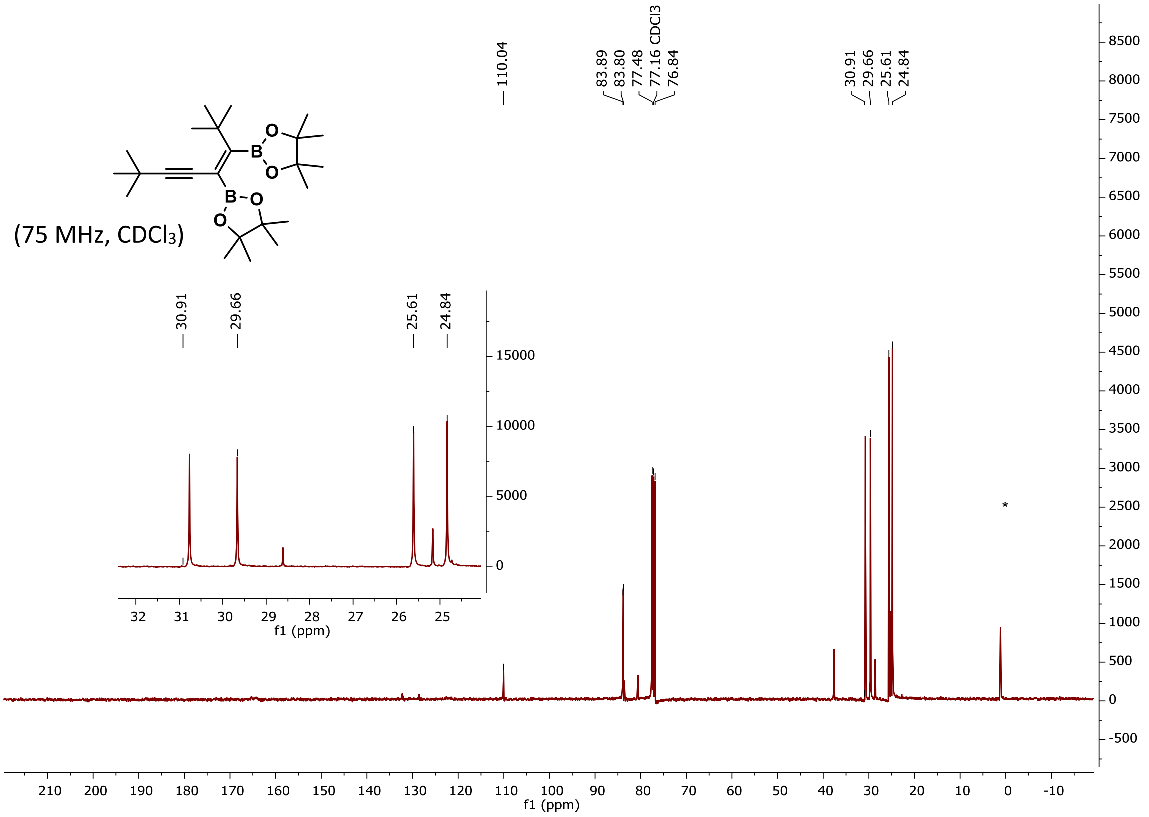


**Figure S78**. $^{13}$C NMR spectrum of **3f**. *-Grease

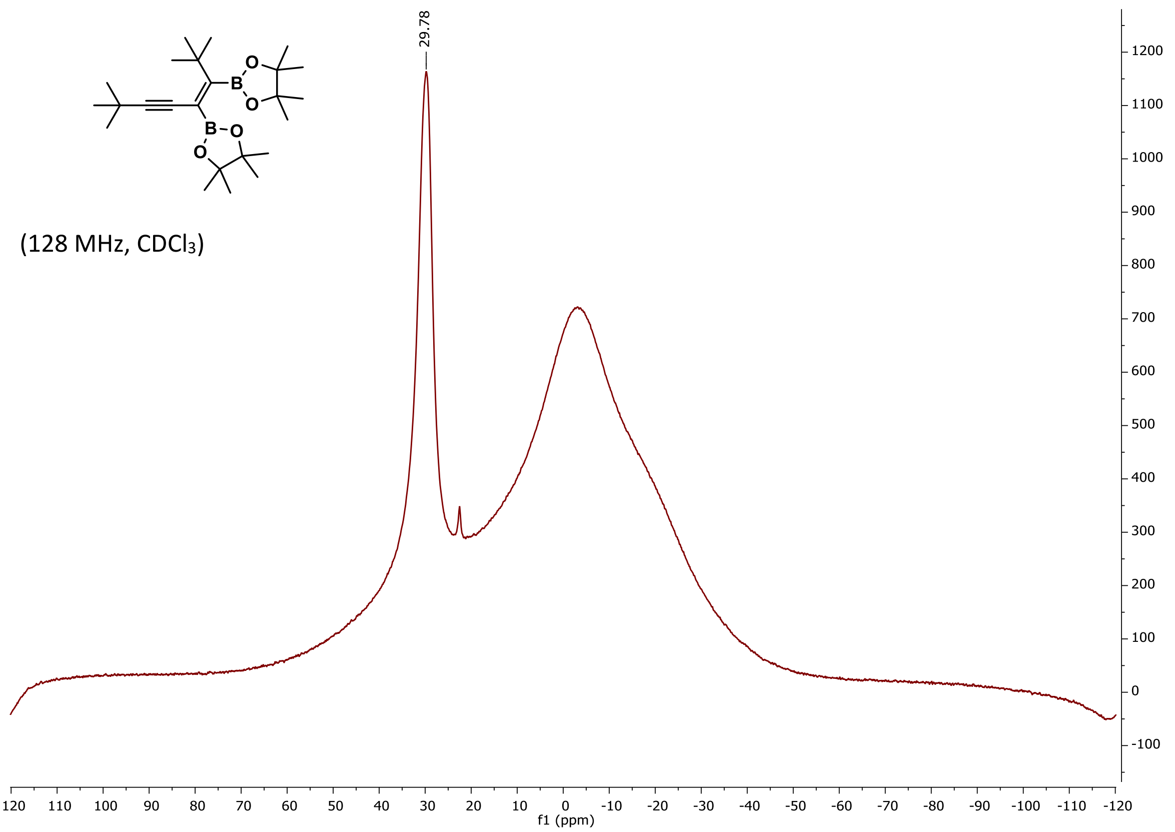


**Figure S79**. $^{11}$B NMR spectrum of **3f**.

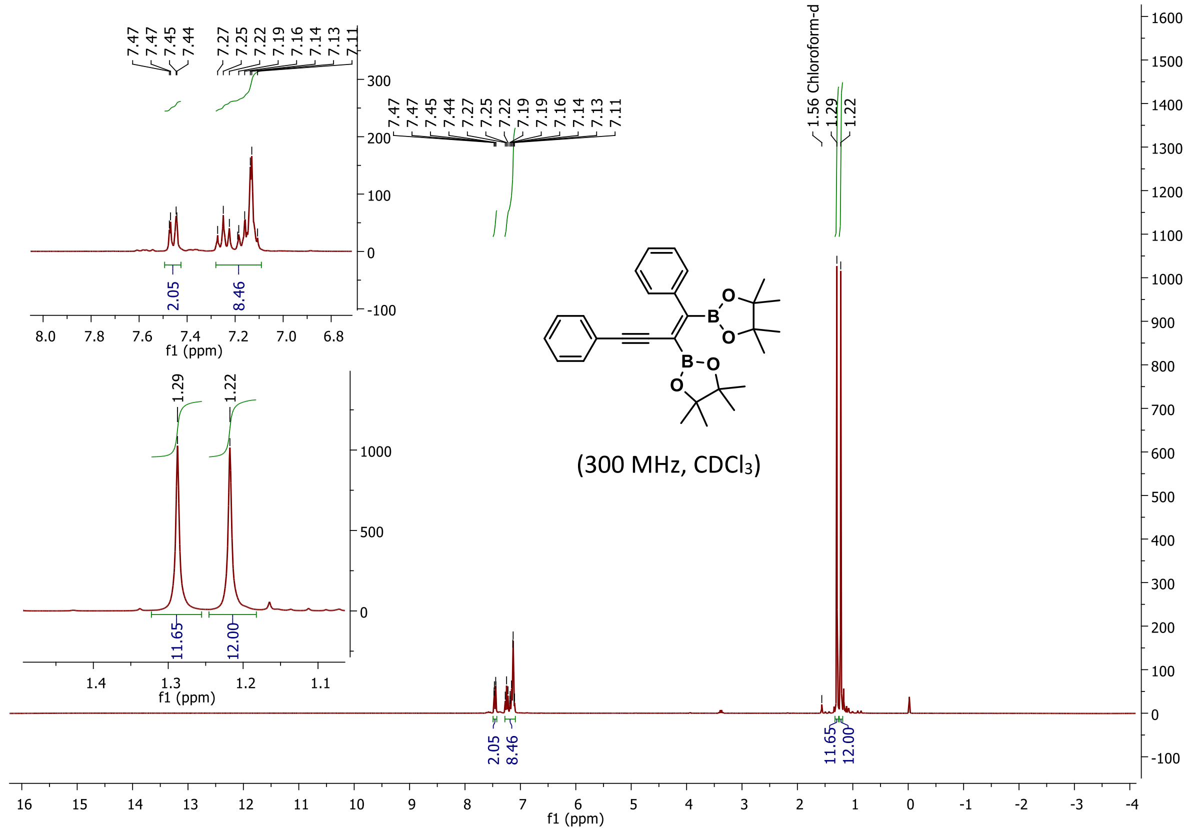


**Figure S80**. $^{1}$H NMR spectrum of **3g**.

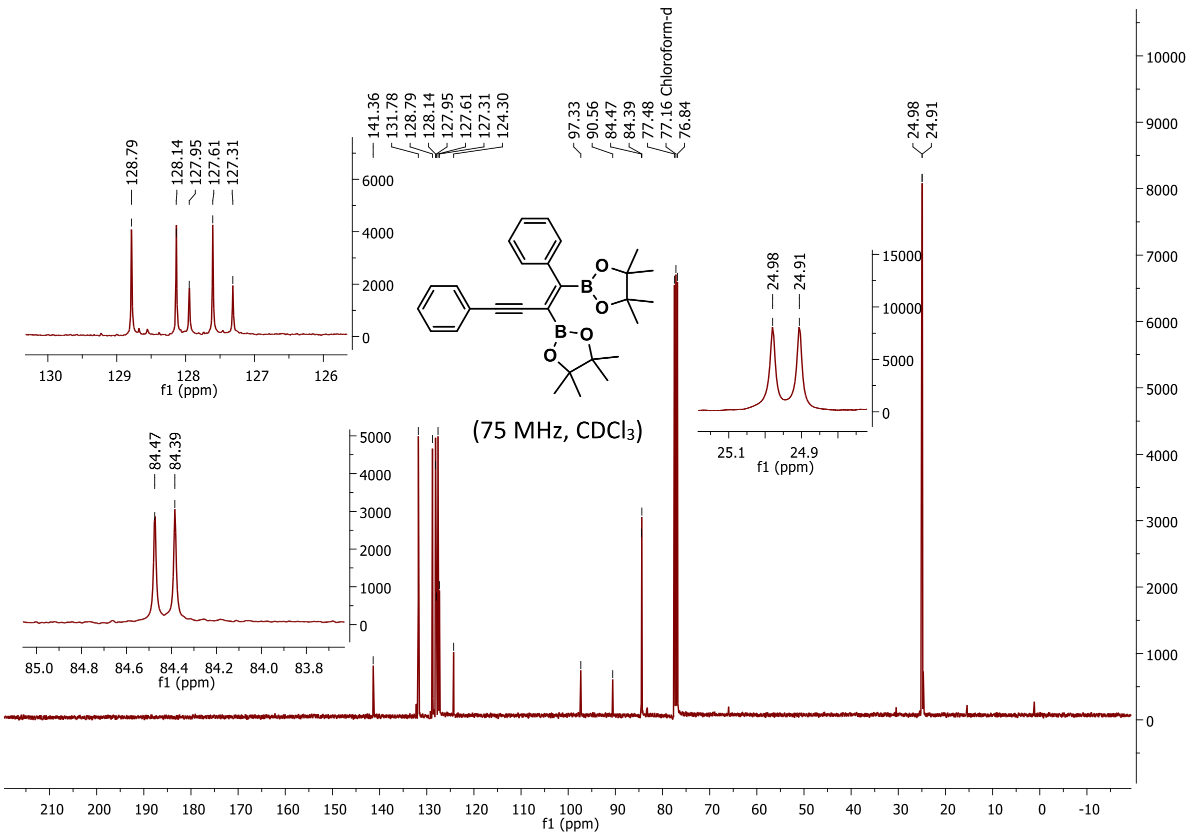


**Figure S81**. $^{13}$C NMR spectrum of **3g**.

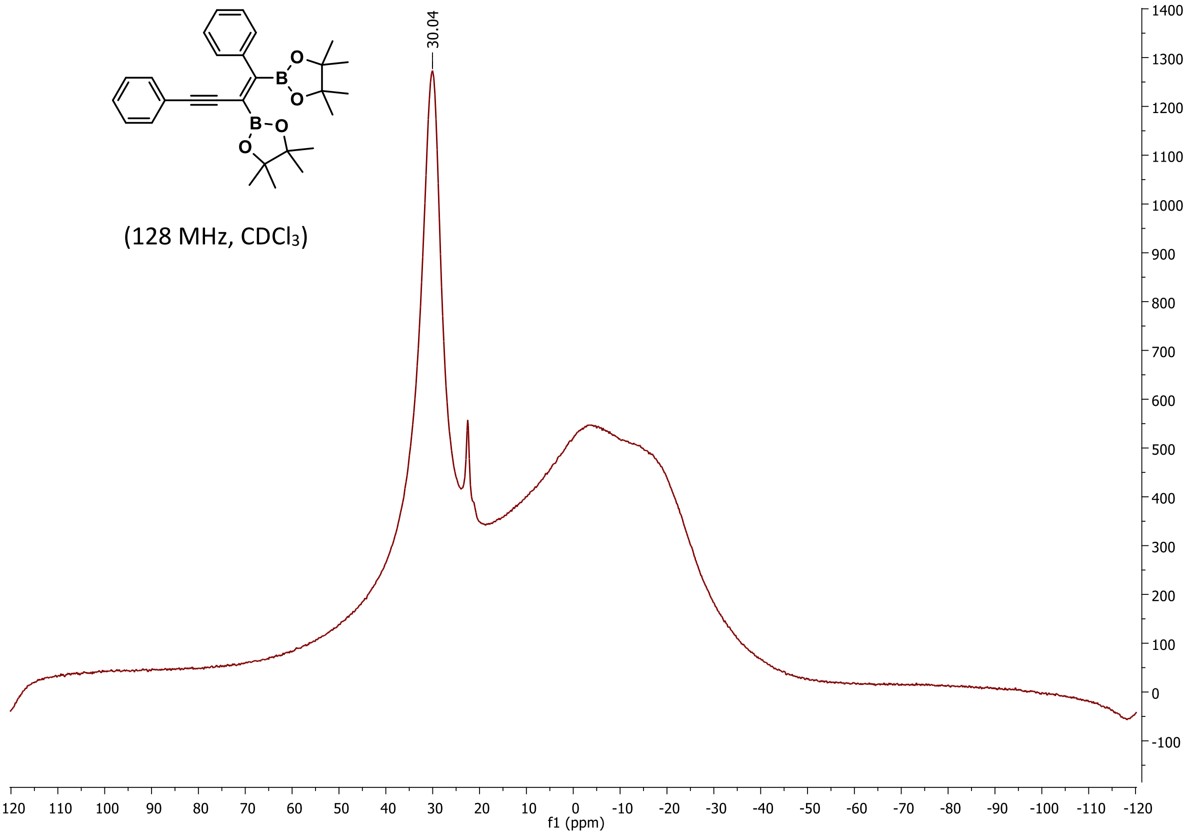


**Figure S82**. $^{11}$B NMR spectrum of **3g**.

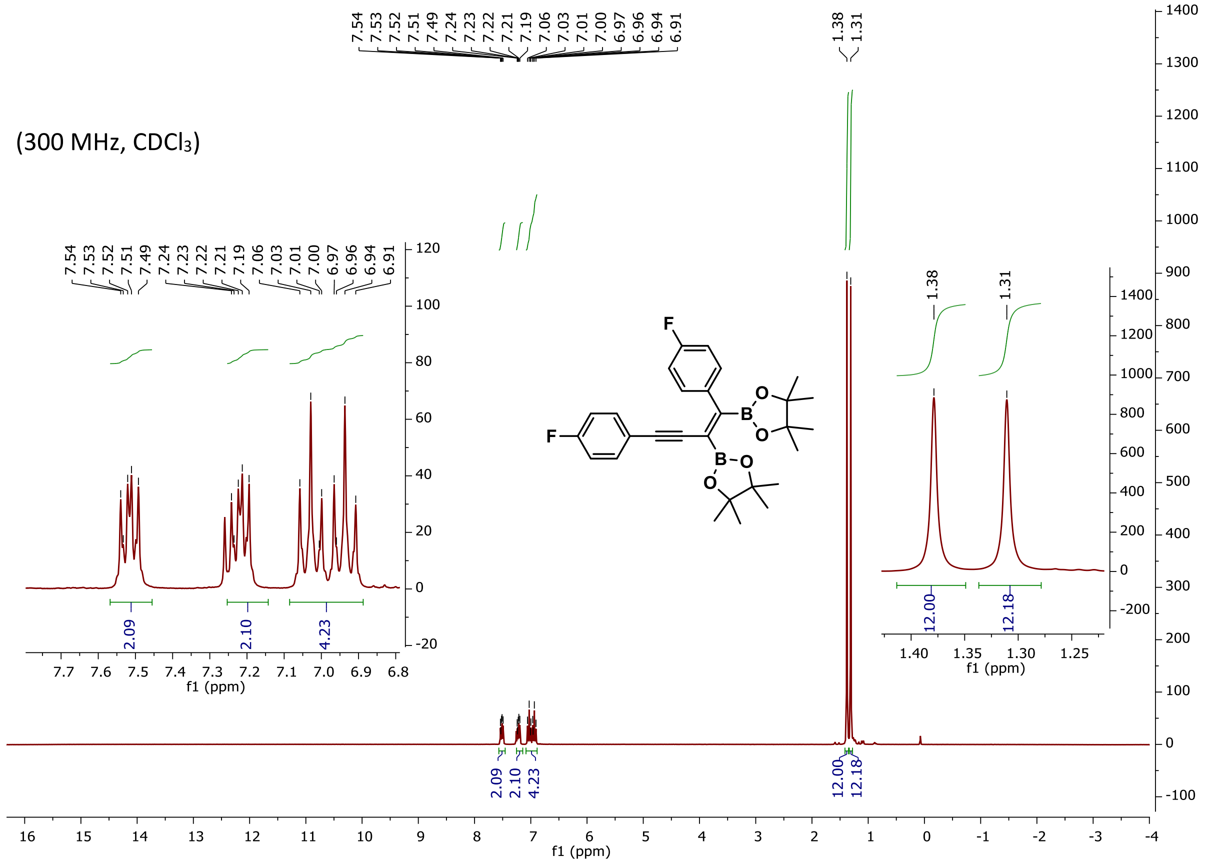


**Figure S83**. $^{1}$H NMR spectrum of **3h**.

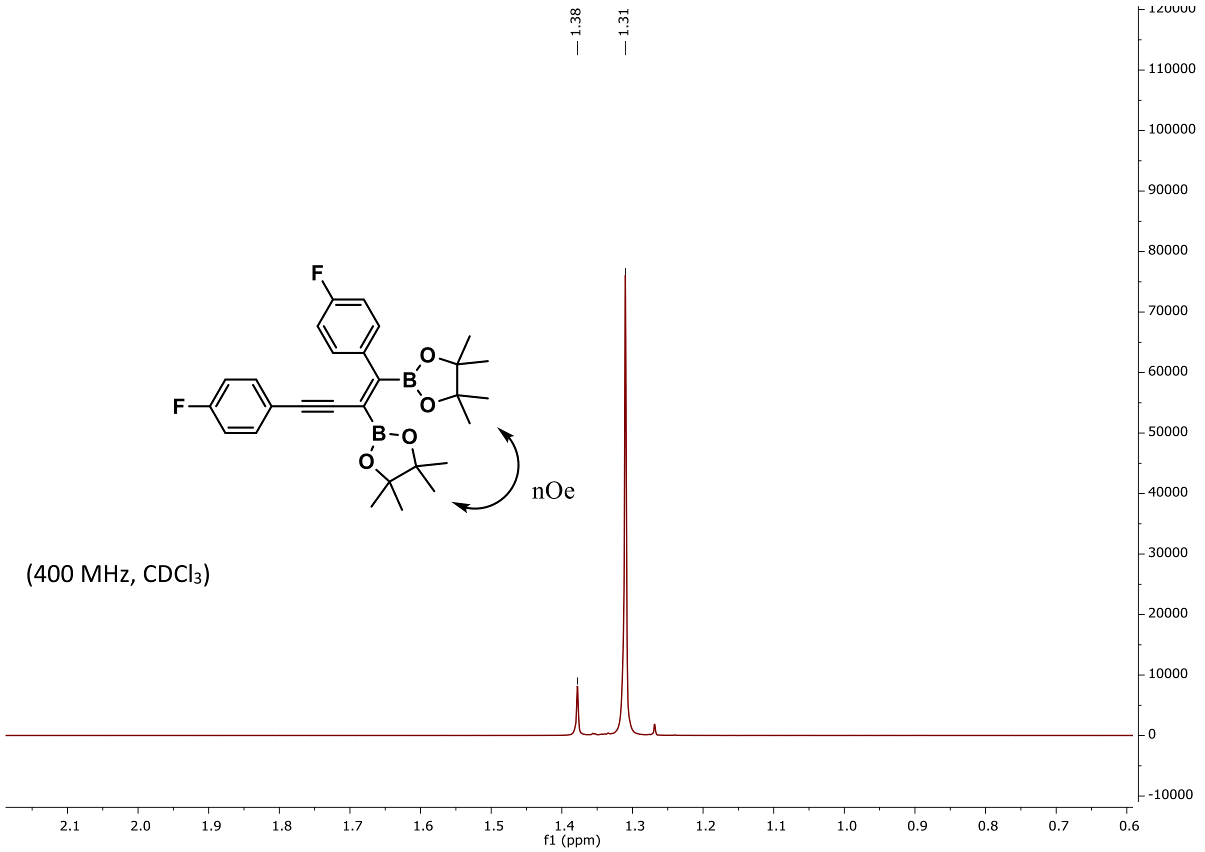


**Figure S84**. 1D selective gradient NOESY of **3h** (freq. 1.33 ppm)

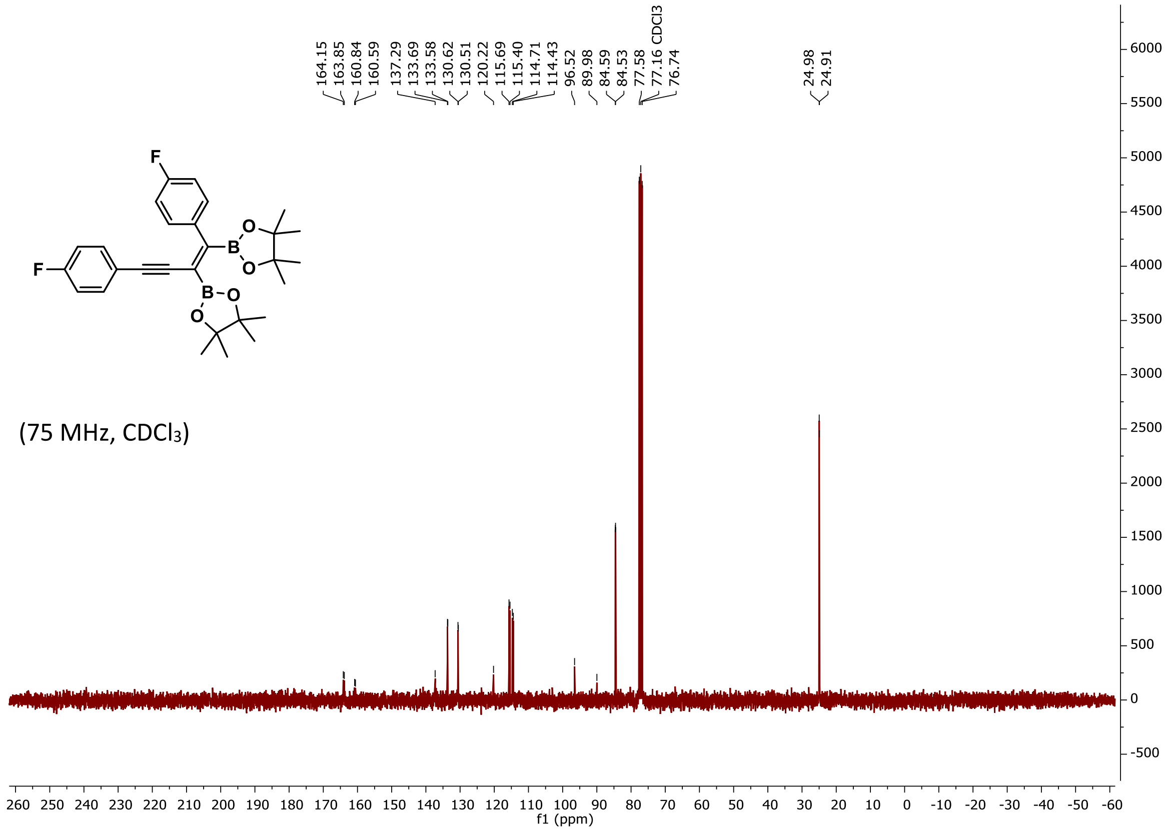


**Figure S85**. $^{13}$C NMR spectrum of **3h**.

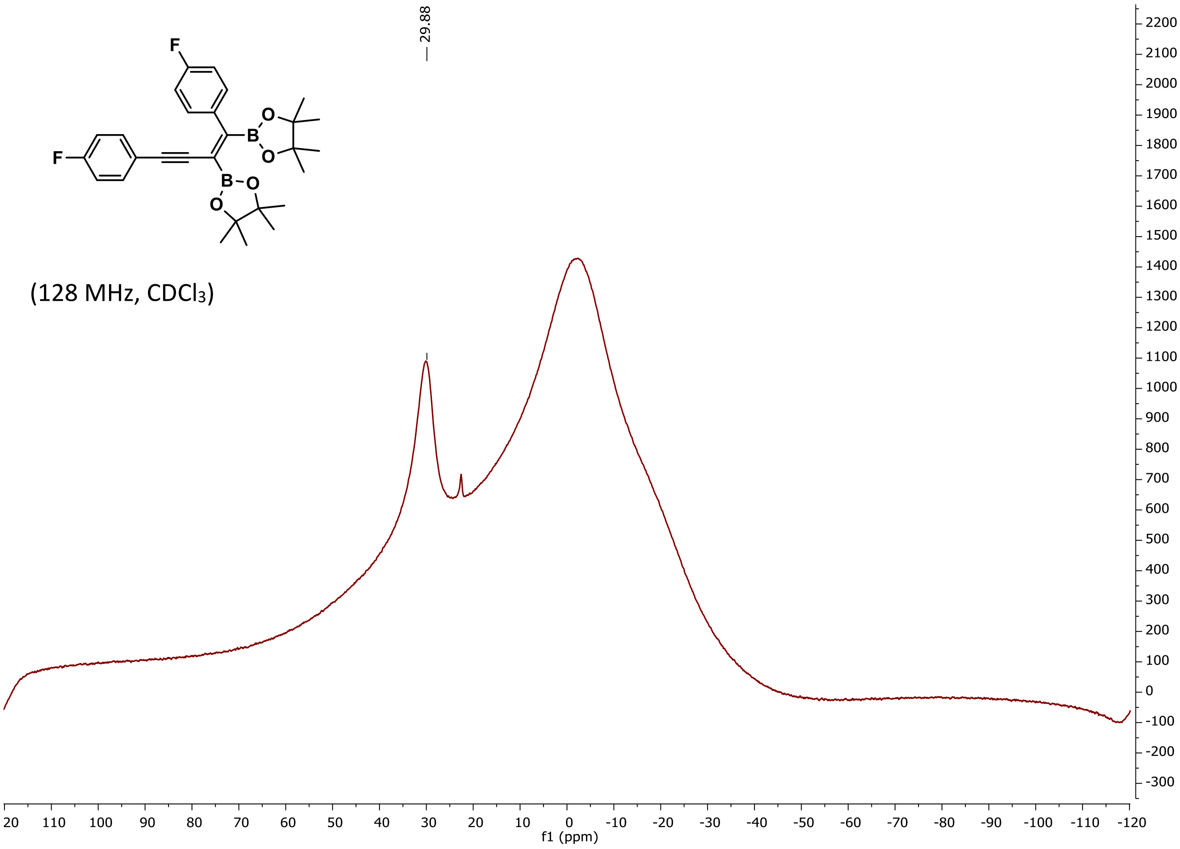


**Figure S86**. $^{11}$B NMR spectrum of **3h**.

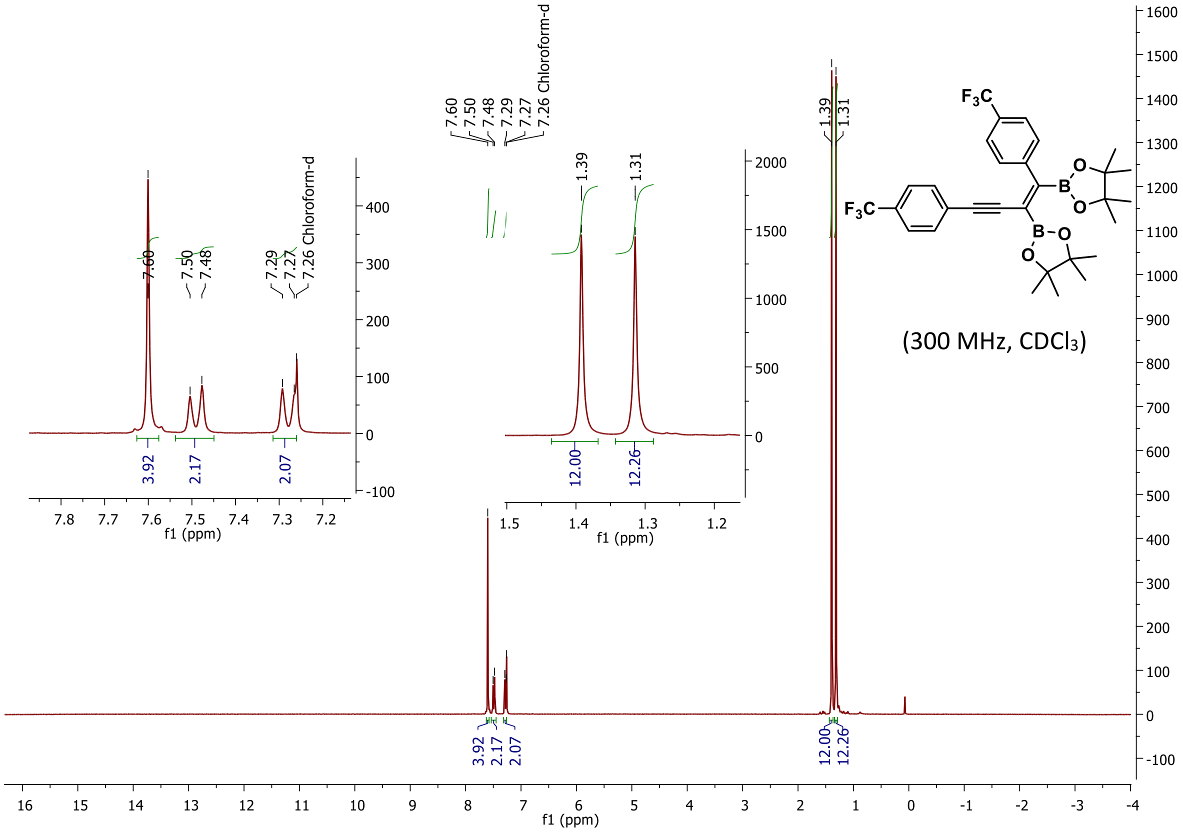


**Figure S87**. [13]H NMR spectrum of **3i**.

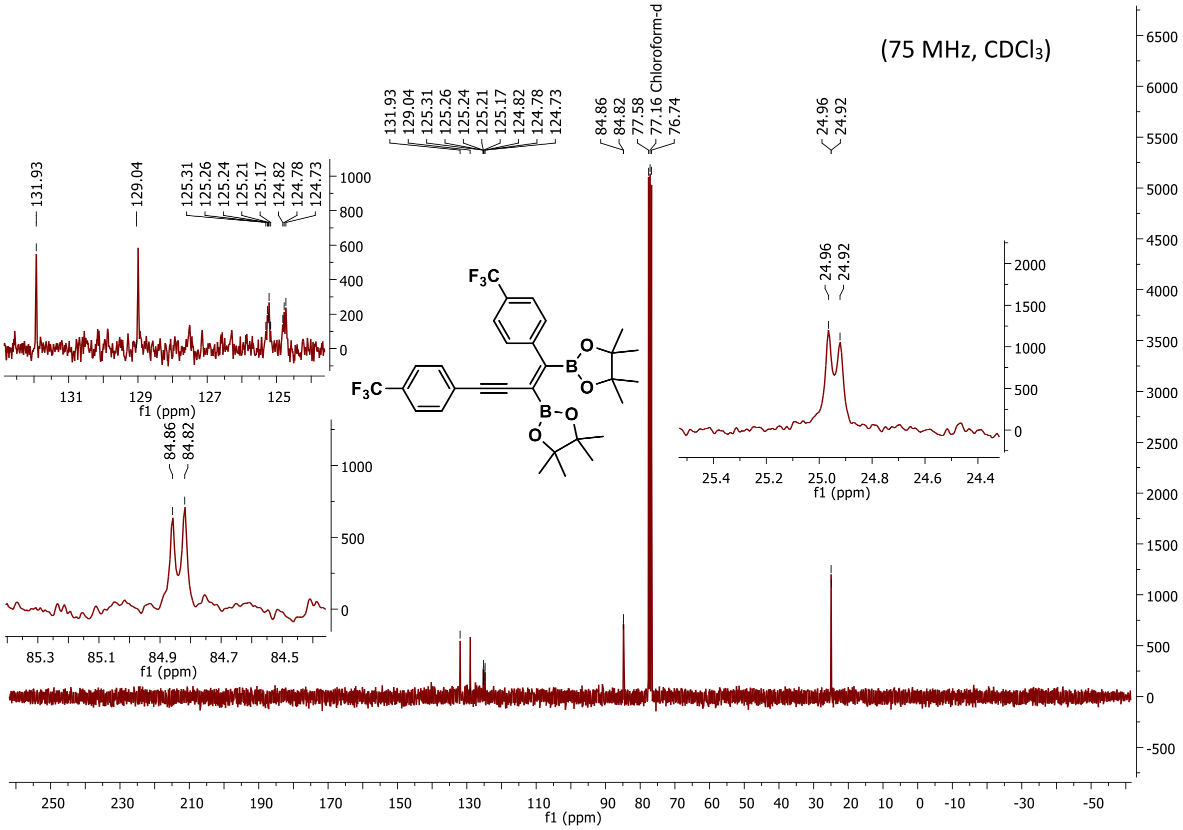


**Figure S88**. $^{13}$C NMR spectrum of **3i**.

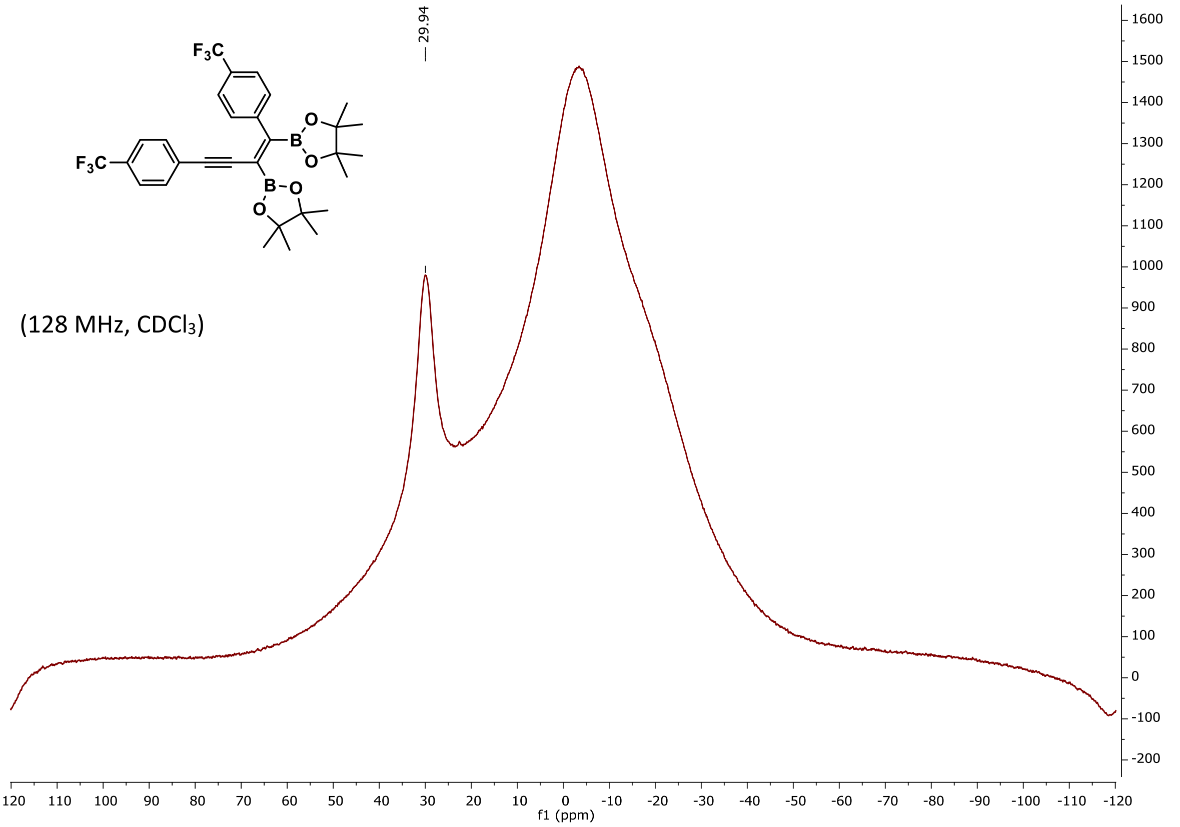

**Figure S89**. $^{11}$B NMR spectrum of **3i**.

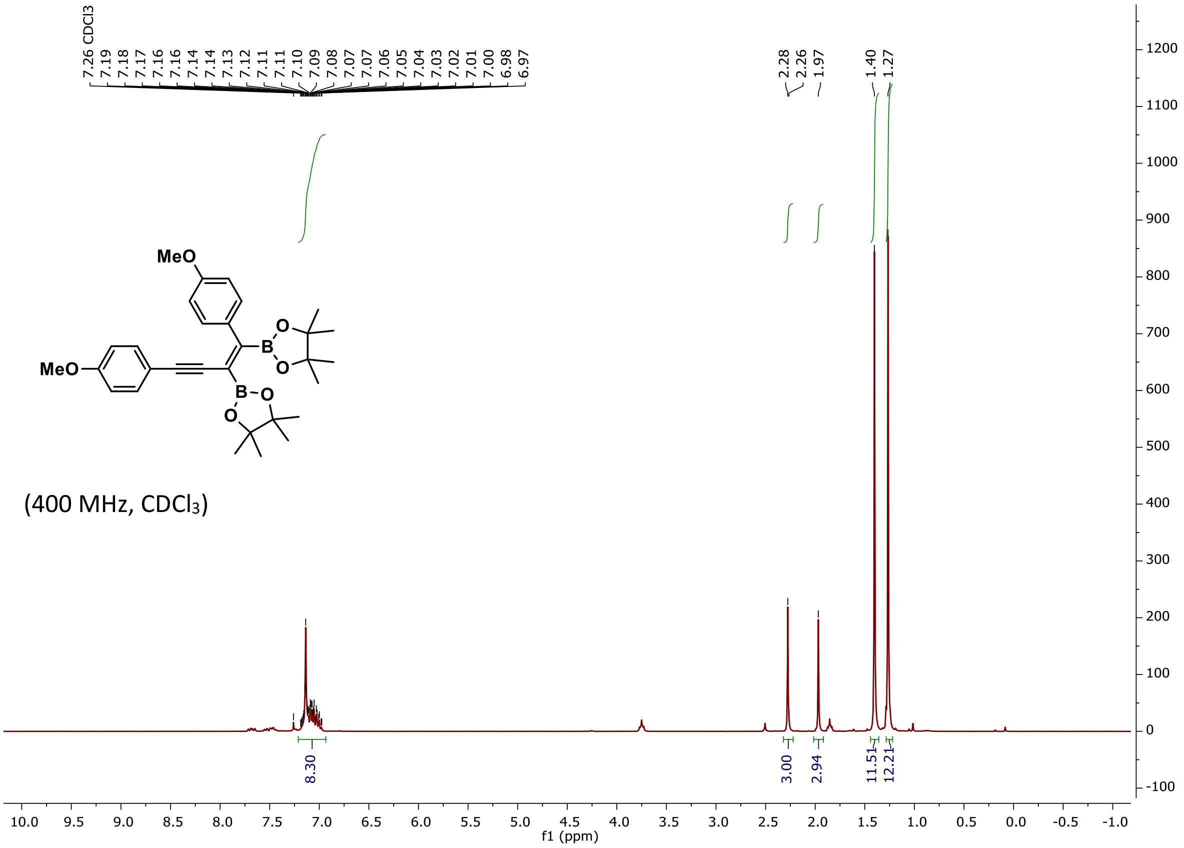

**Figure S90**. $^{1}$H NMR spectrum of **3j**.

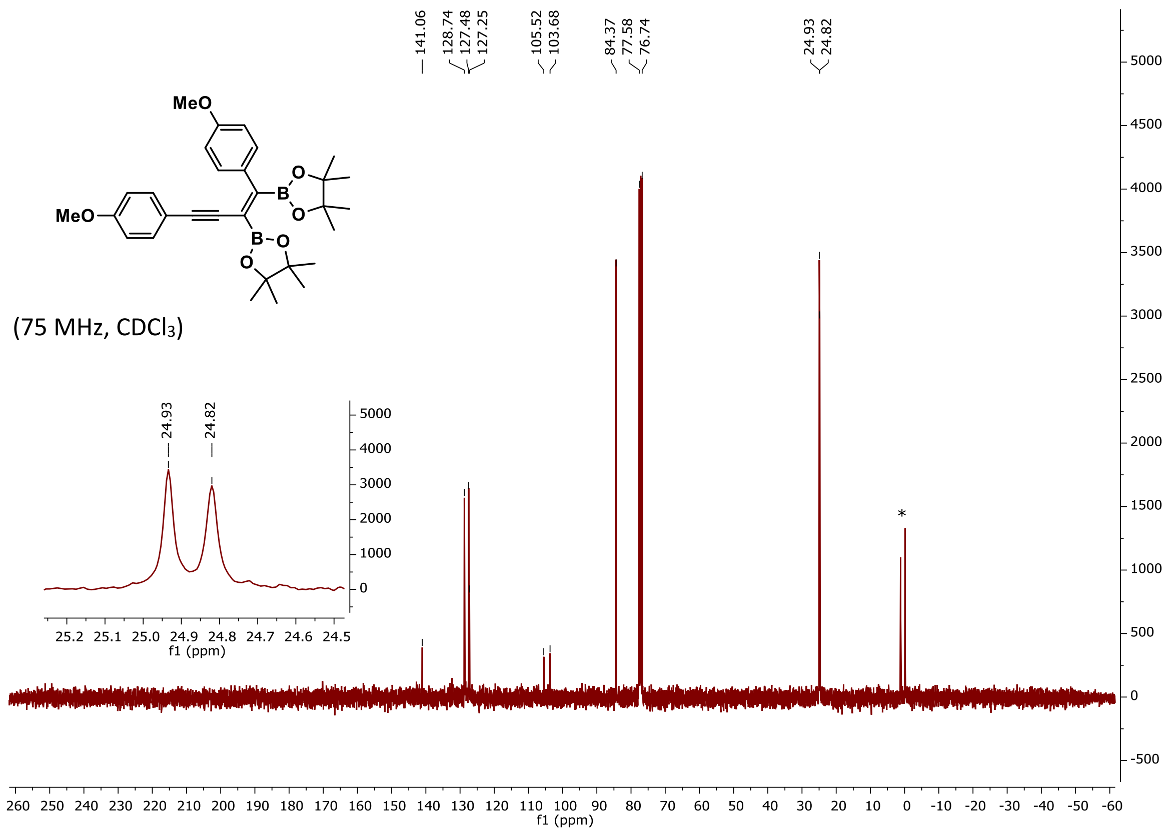


**Figure S91**. $^{13}C$ NMR spectrum of **3j**.

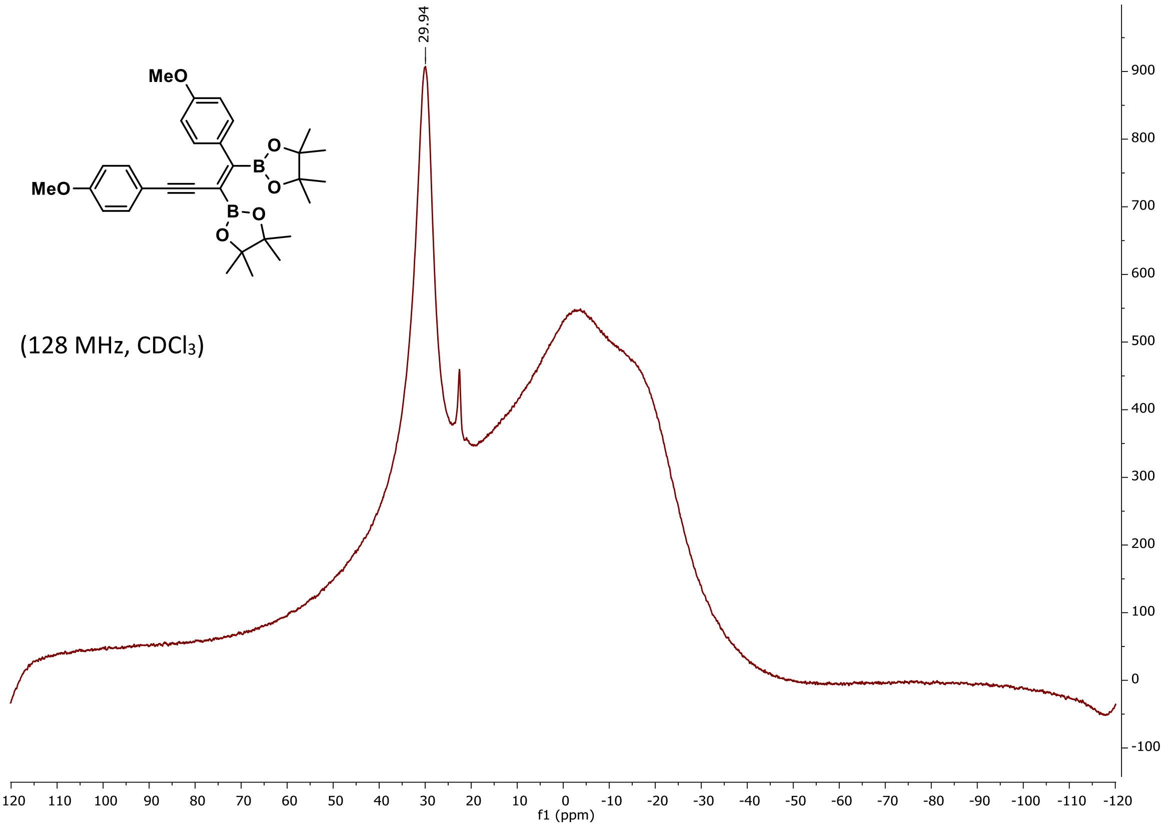


**Figure S92**. $^{11}B$ NMR spectrum of **3j**.

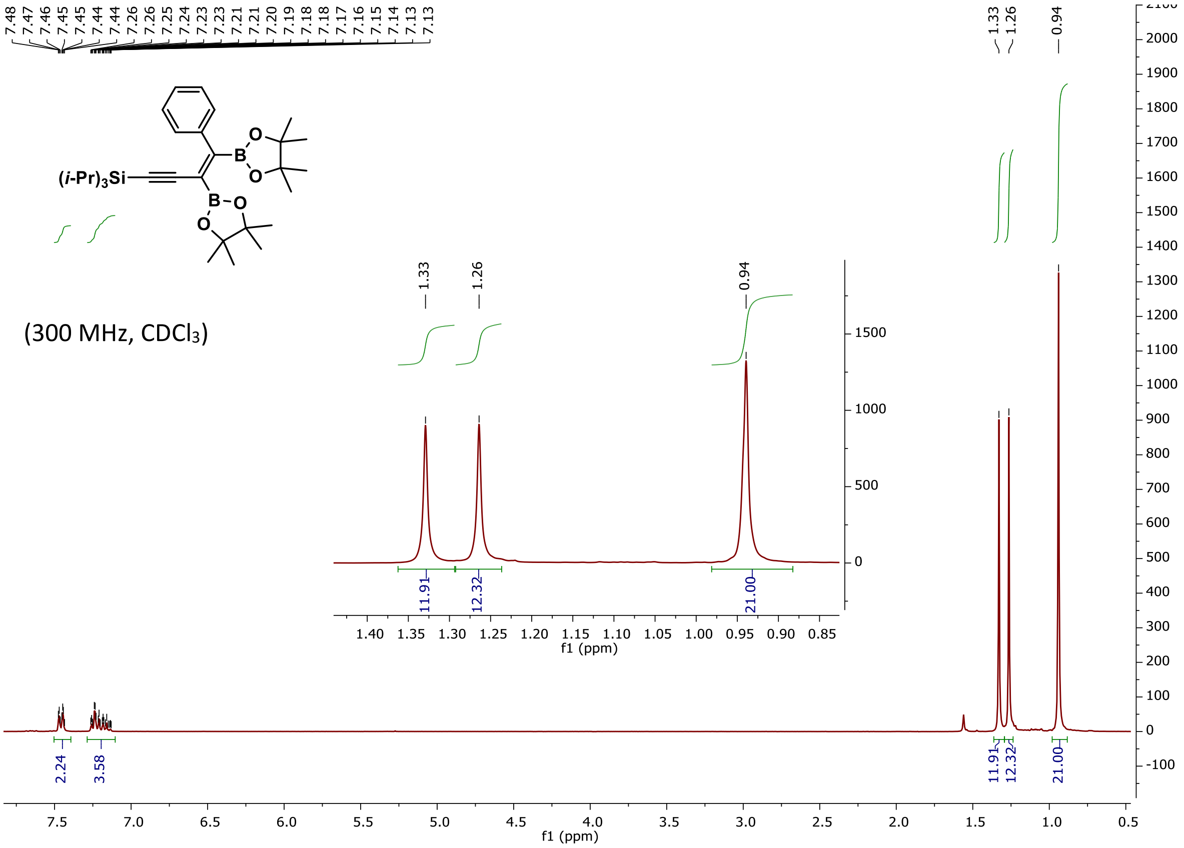


**Figure S93**. $^{1}$H NMR spectrum of **3m**.

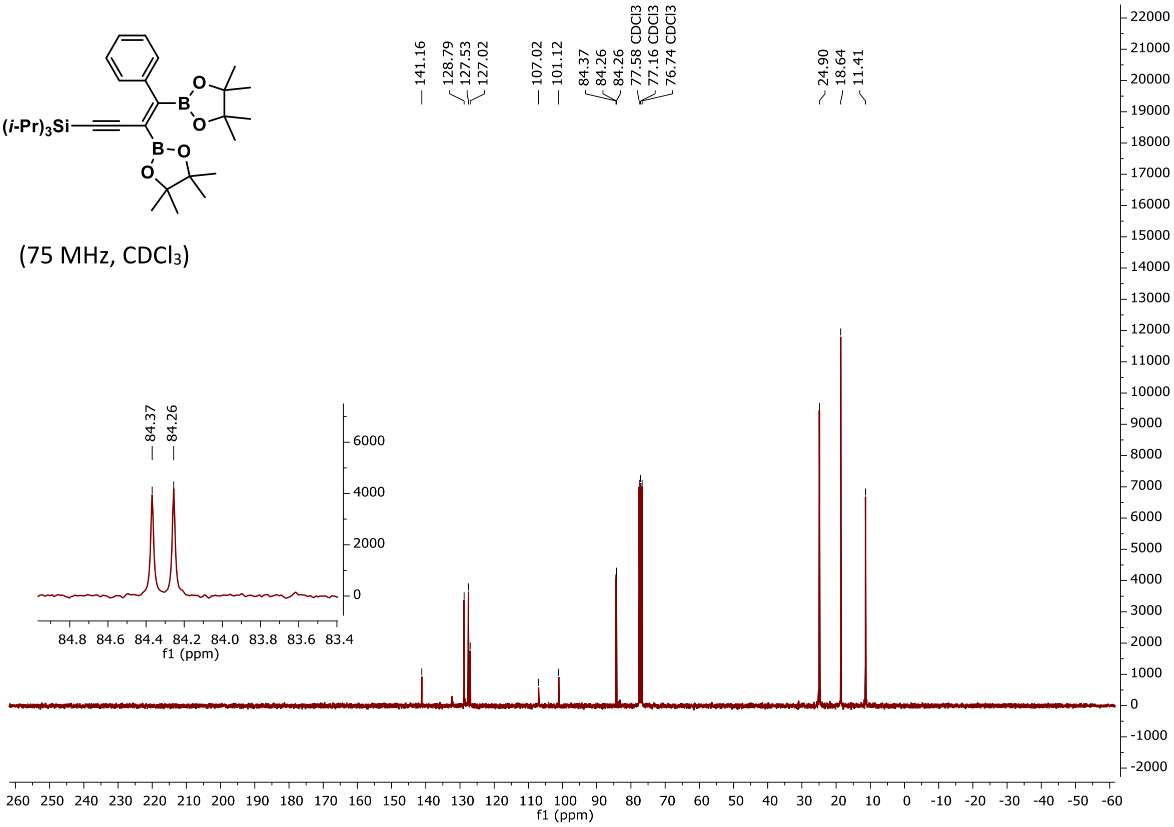


**Figure S94**. $^{13}$C NMR spectrum of **3m**.

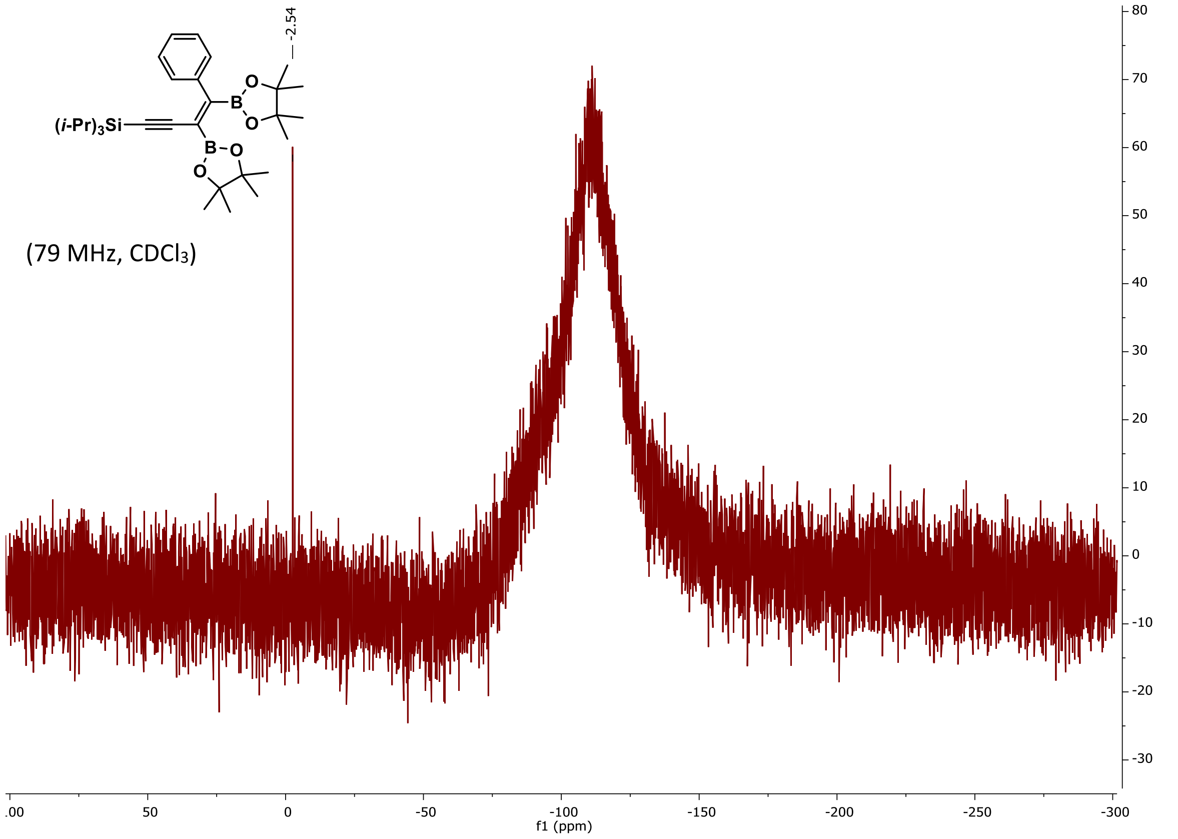


**Figure S95**. $^{29}$Si NMR spectrum of **3m**.

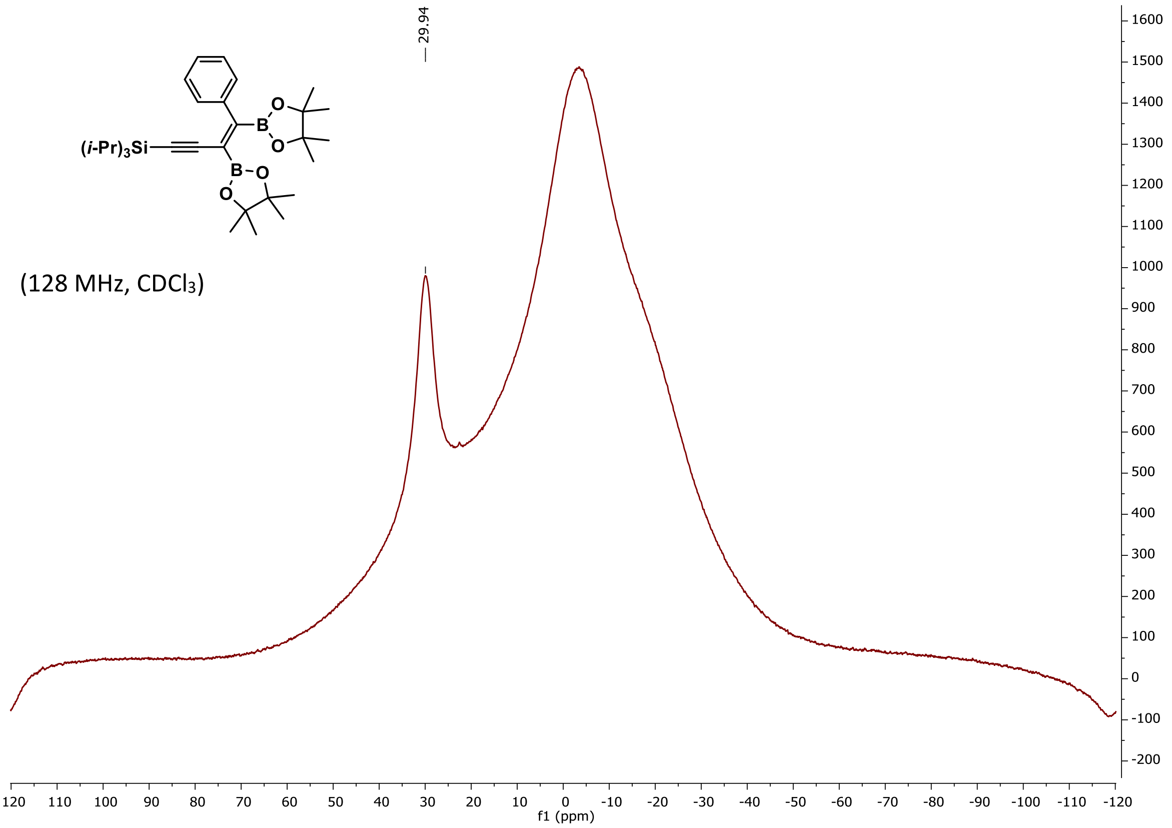


**Figure S96**. $^{11}$B NMR spectrum of **3m**.

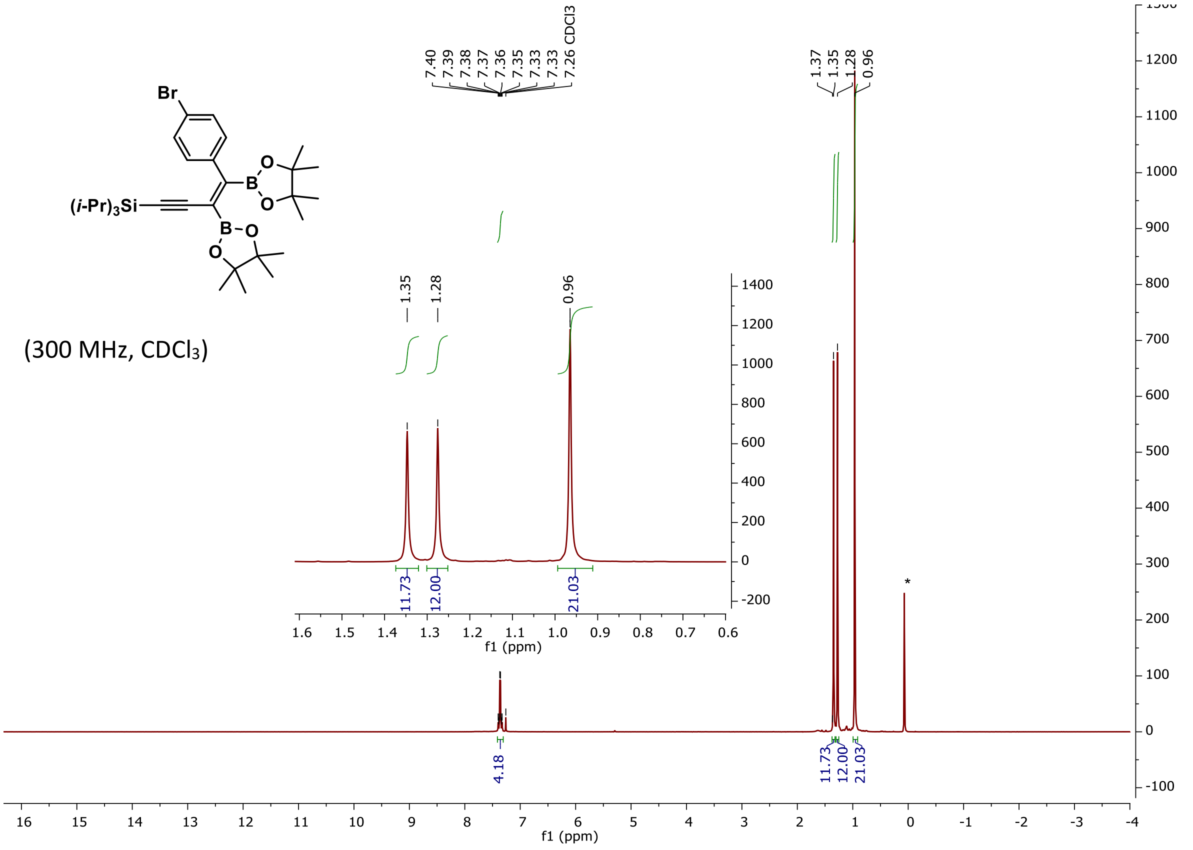

**Figure S97**. $^{1}$H NMR spectrum of **3n**. *-Grease

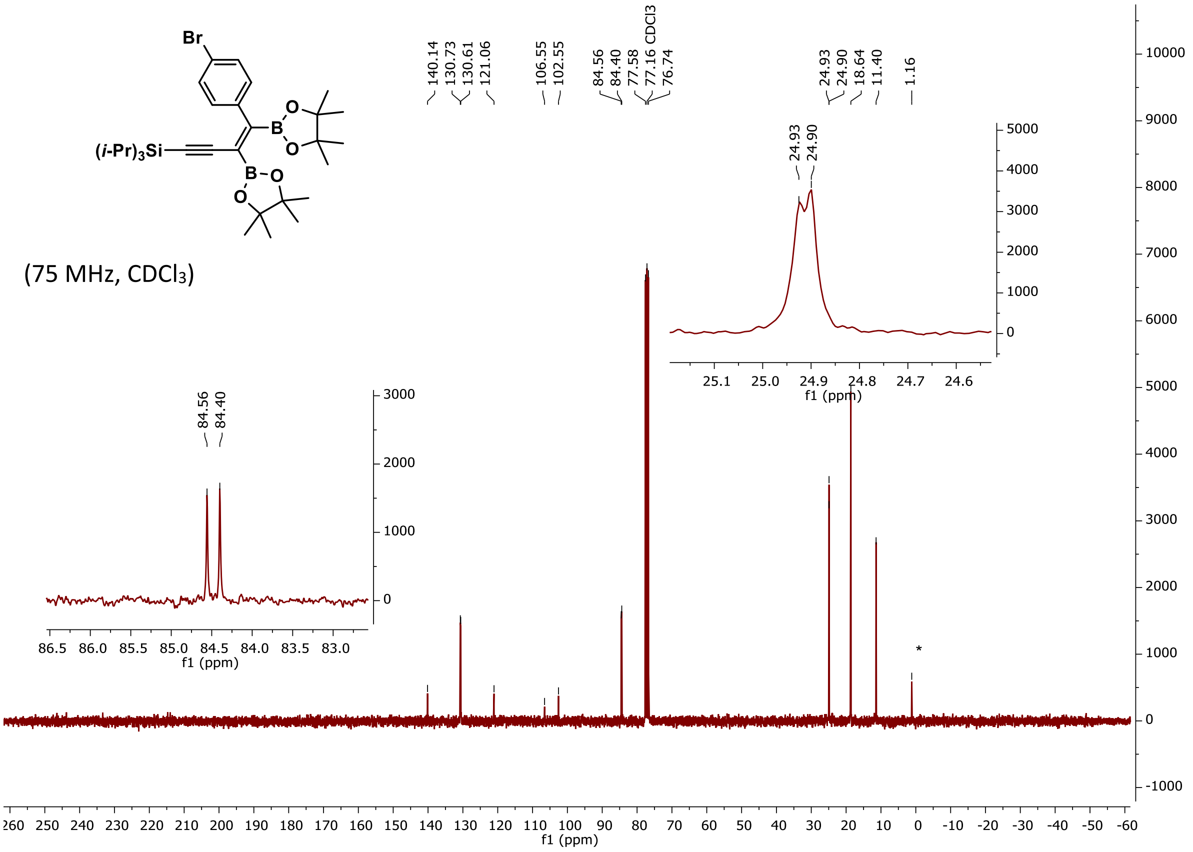

**Figure S98**. $^{13}$C NMR spectrum of **3n**. *-Grease

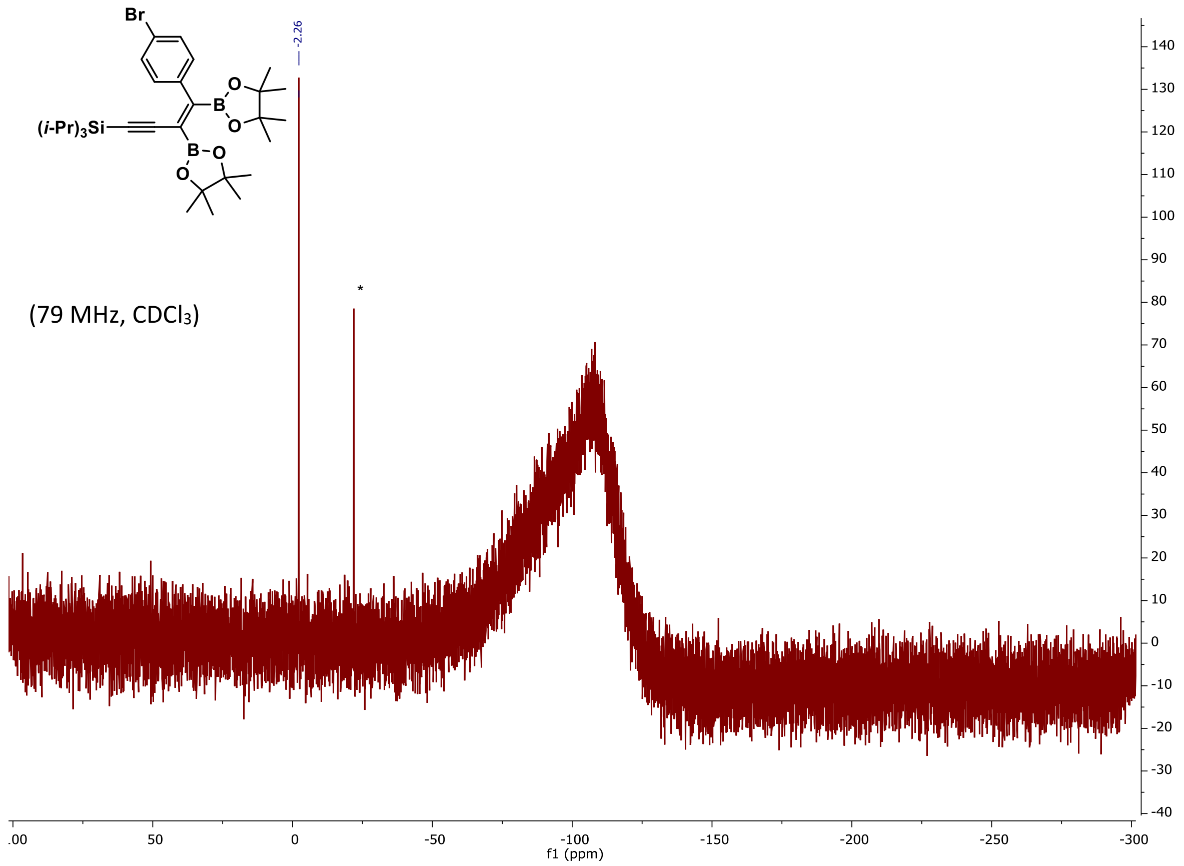


**Figure S99**. $^{29}$Si NMR spectrum of **3n**. *-Grease

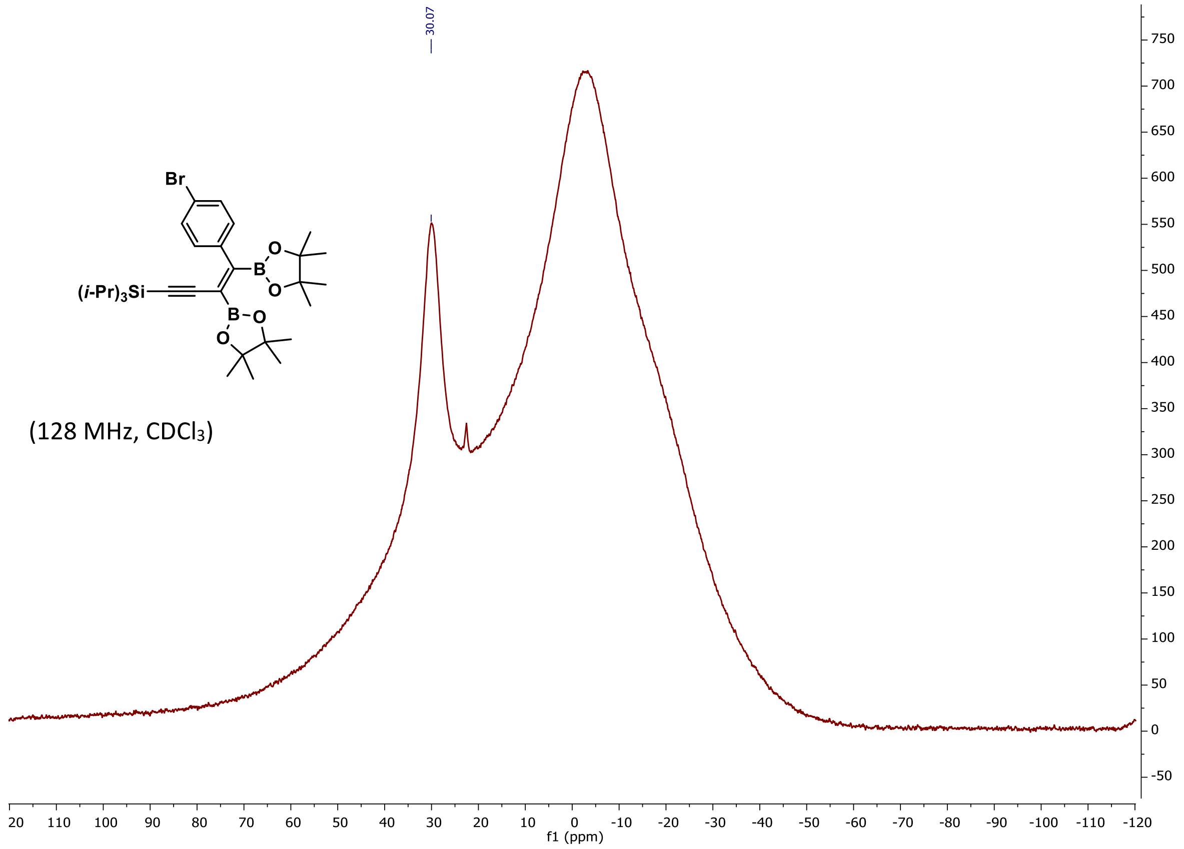


**Figure S100**. $^{11}$B NMR spectrum of **3n**.

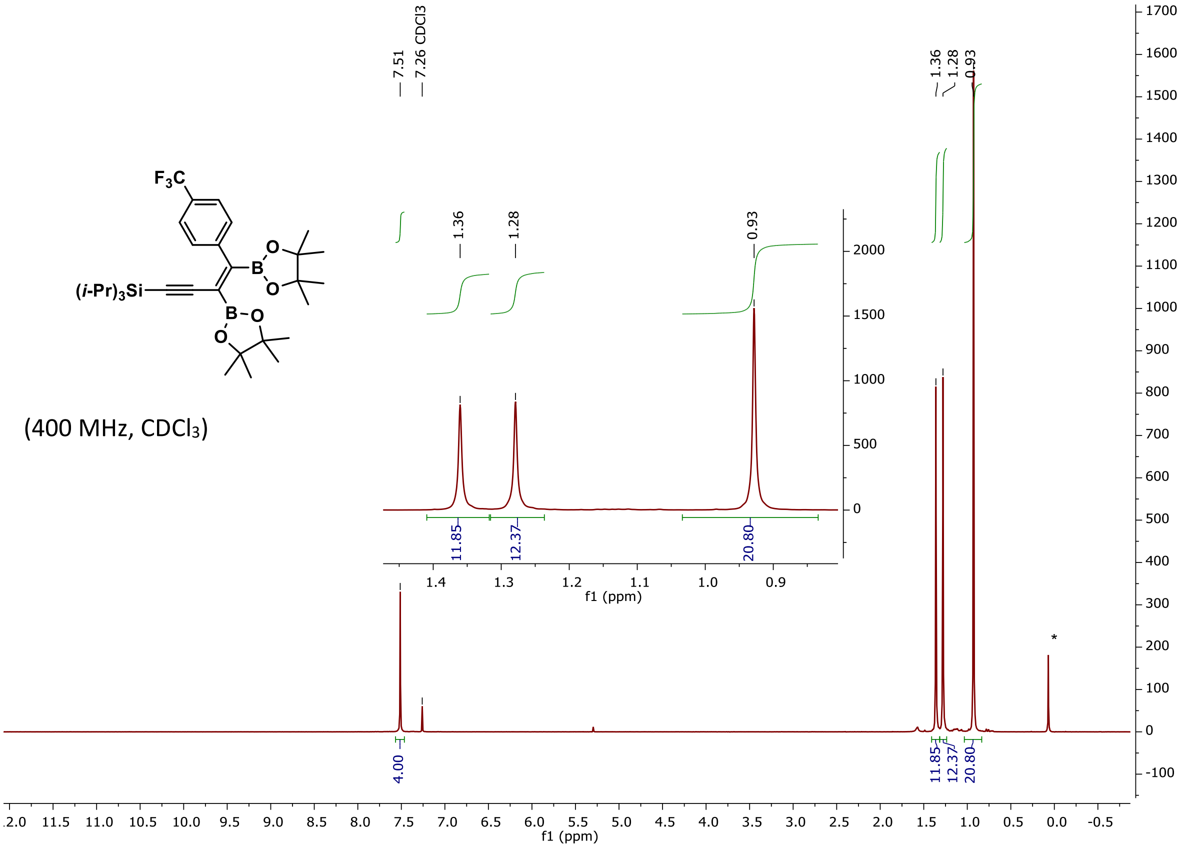


**Figure S101**. $^{1}$H NMR spectrum of **3o**. *-Grease.

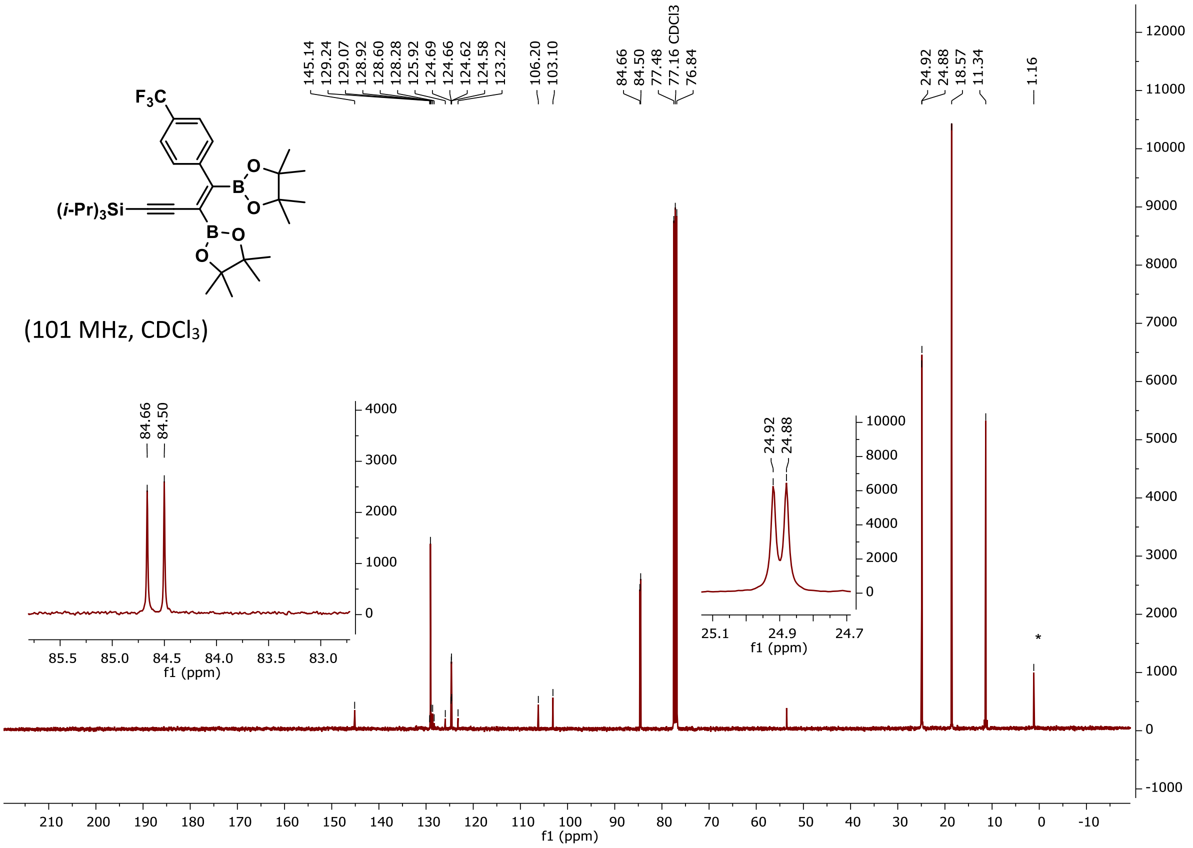


**Figure S102**. $^{13}$C NMR spectrum of **3o**. *-Grease.

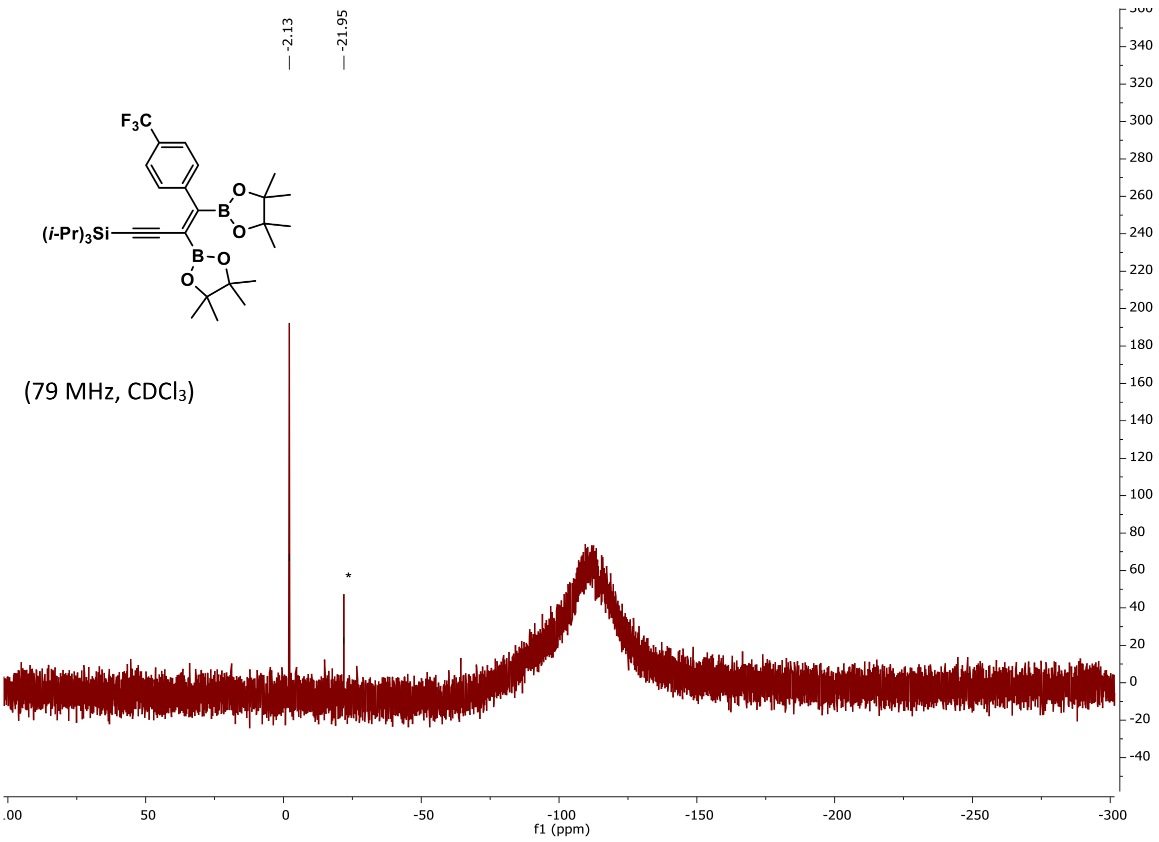


**Figure S103**. $^{29}$Si NMR spectrum of **3o**. *-Grease

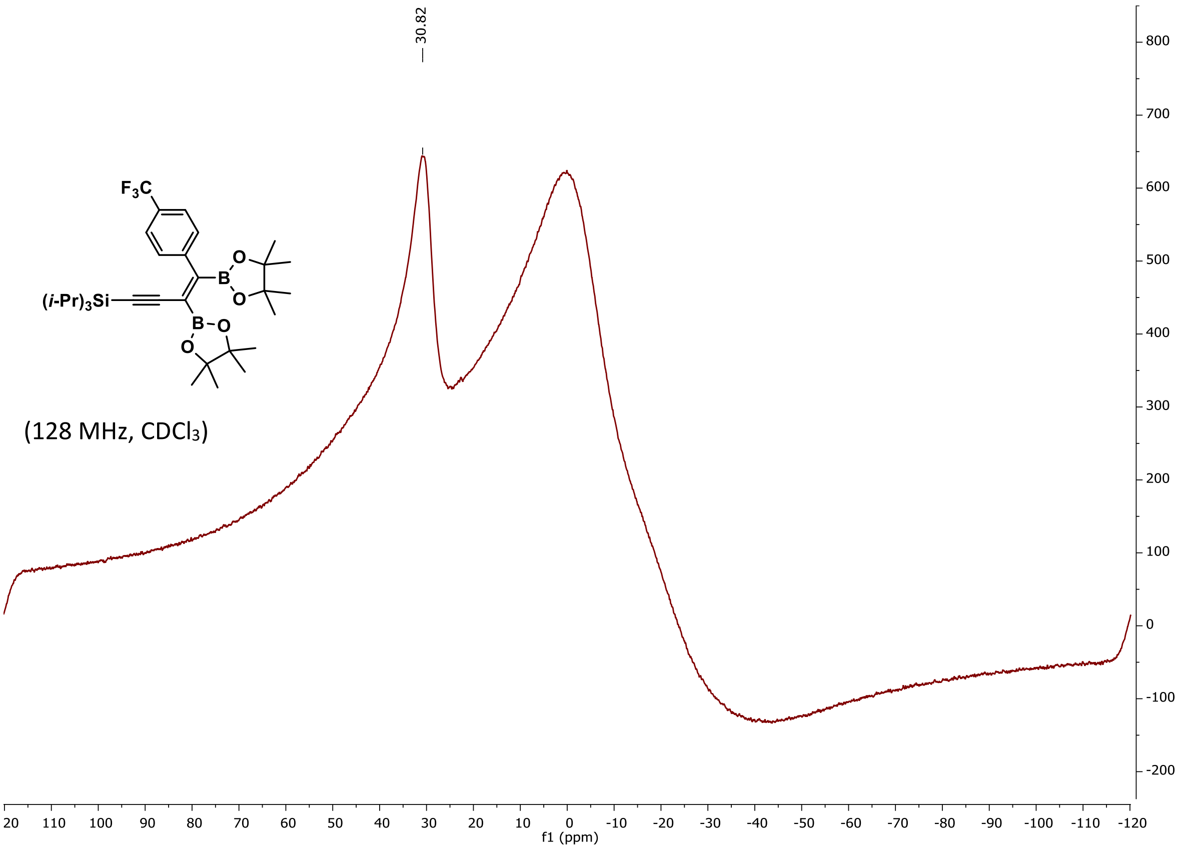


**Figure S104**. $^{11}$B NMR spectrum of **3o**.

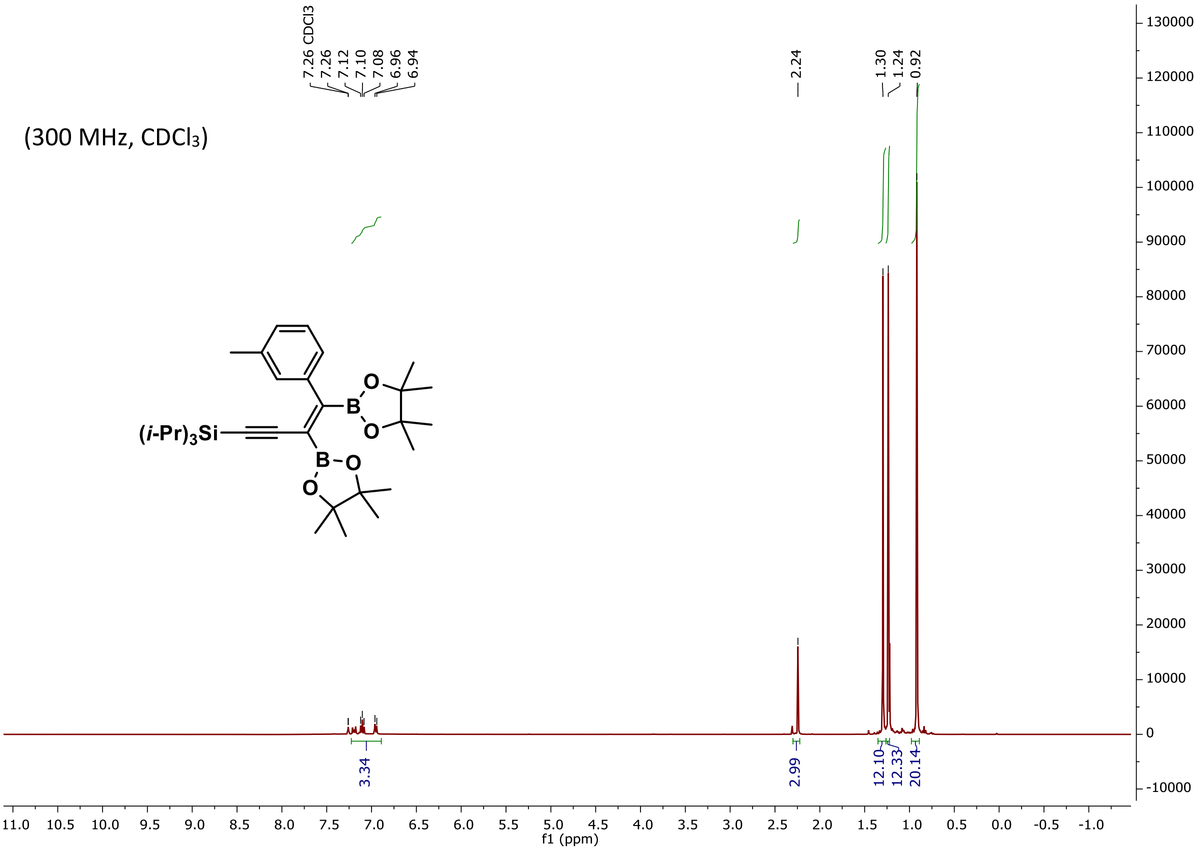


**Figure S105**. $^{1}$H NMR spectrum of (**3p**)

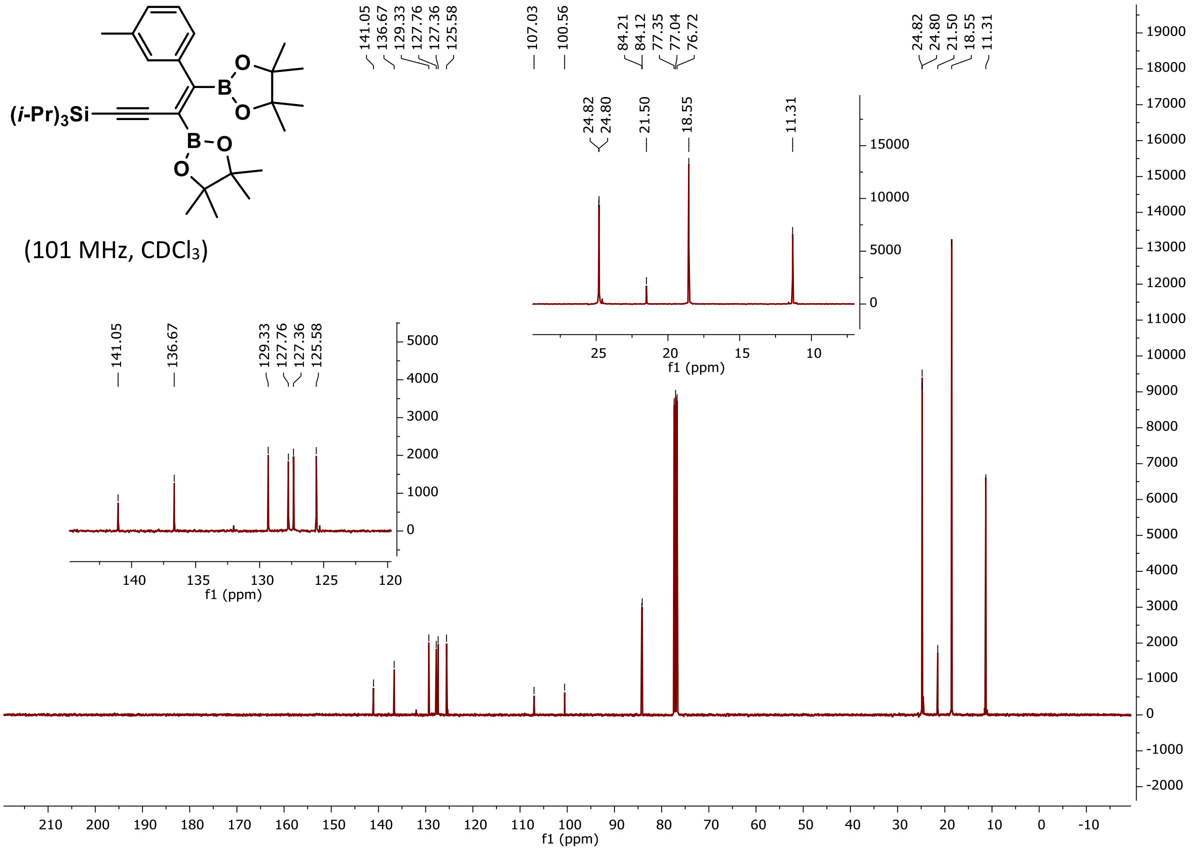


**Figure S106**. $^{11}$C NMR spectrum of (**3p**)

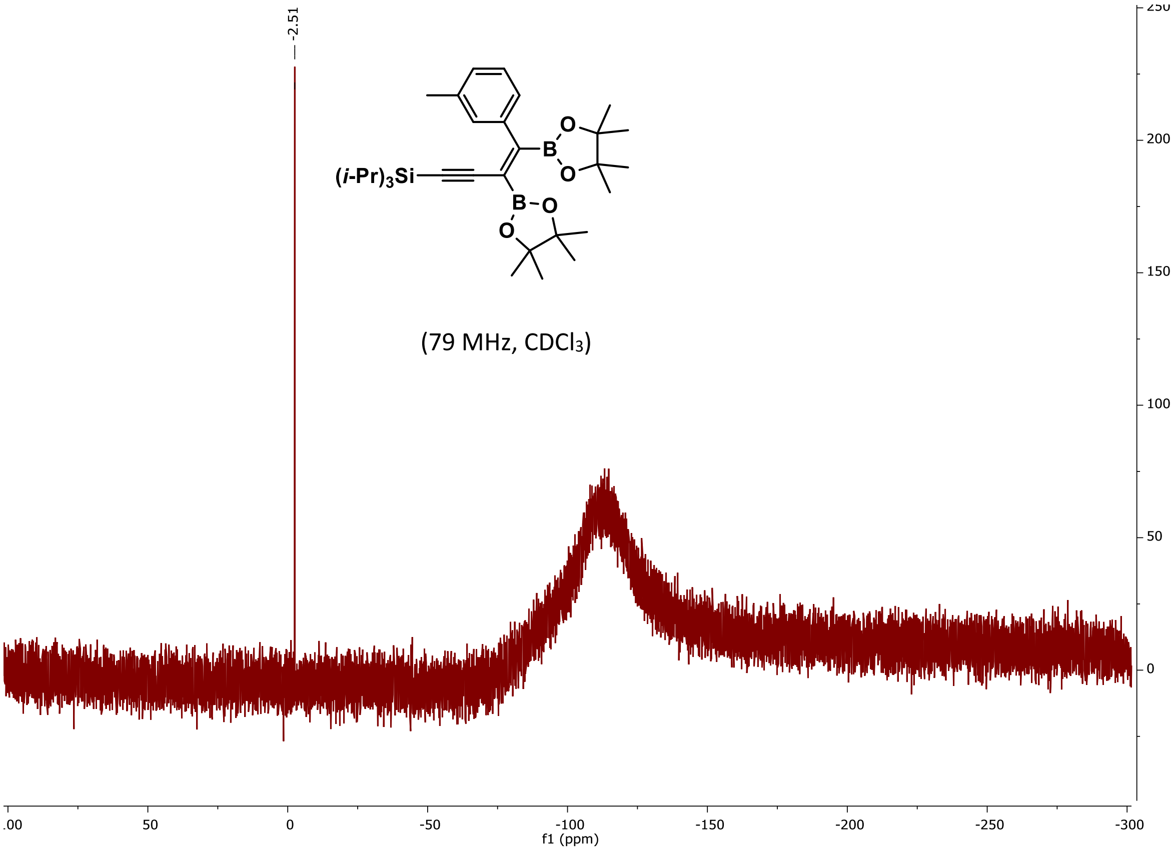


**Figure S107**. $^{29}$Si NMR spectrum of (**3p**)

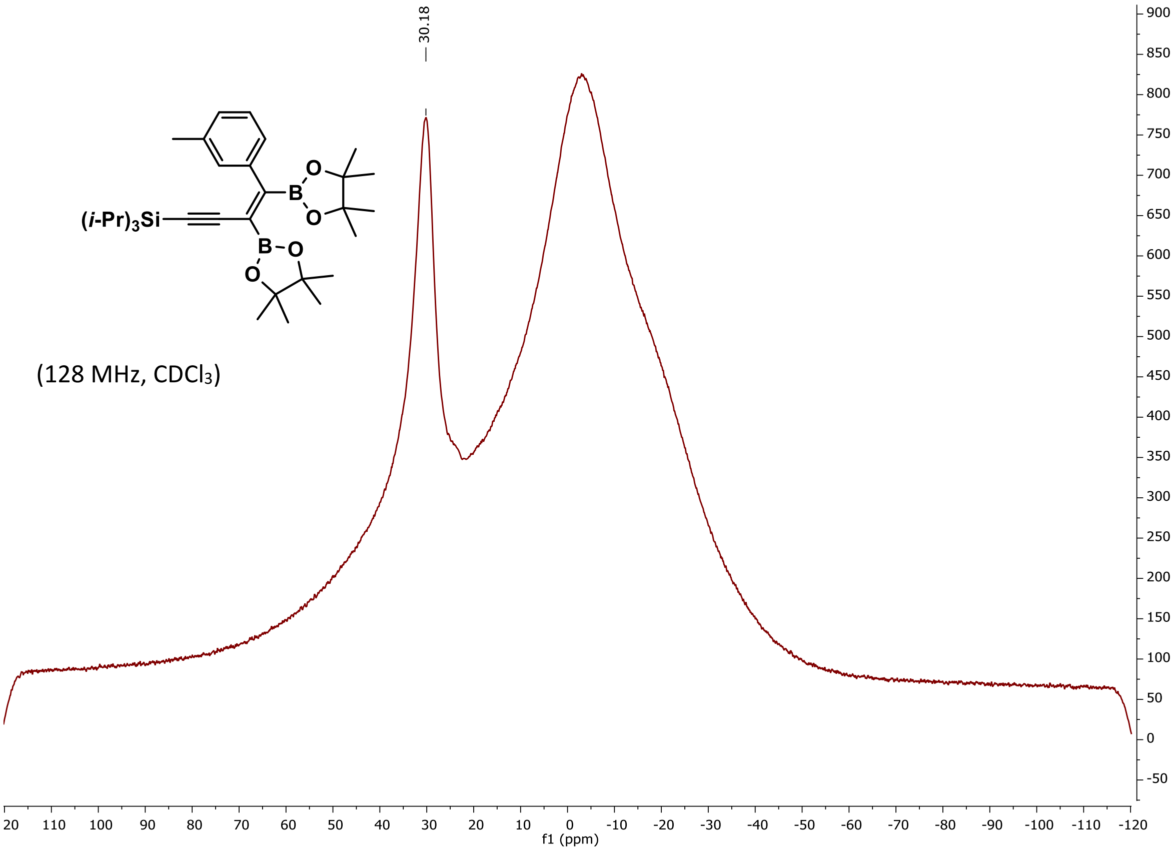


**Figure S108**. $^{11}$B NMR spectrum of (**3p**)

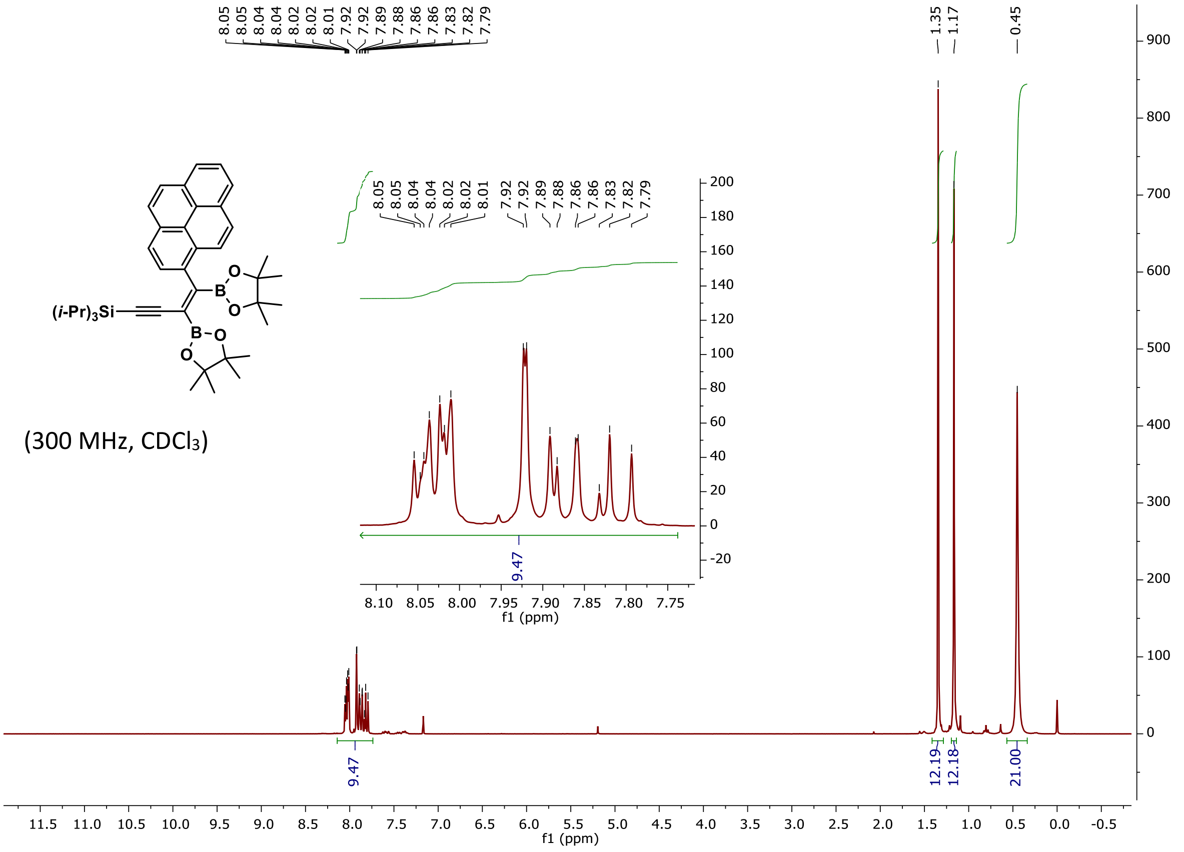


**Figure S109**. $^{1}$H NMR spectrum of **3q**.

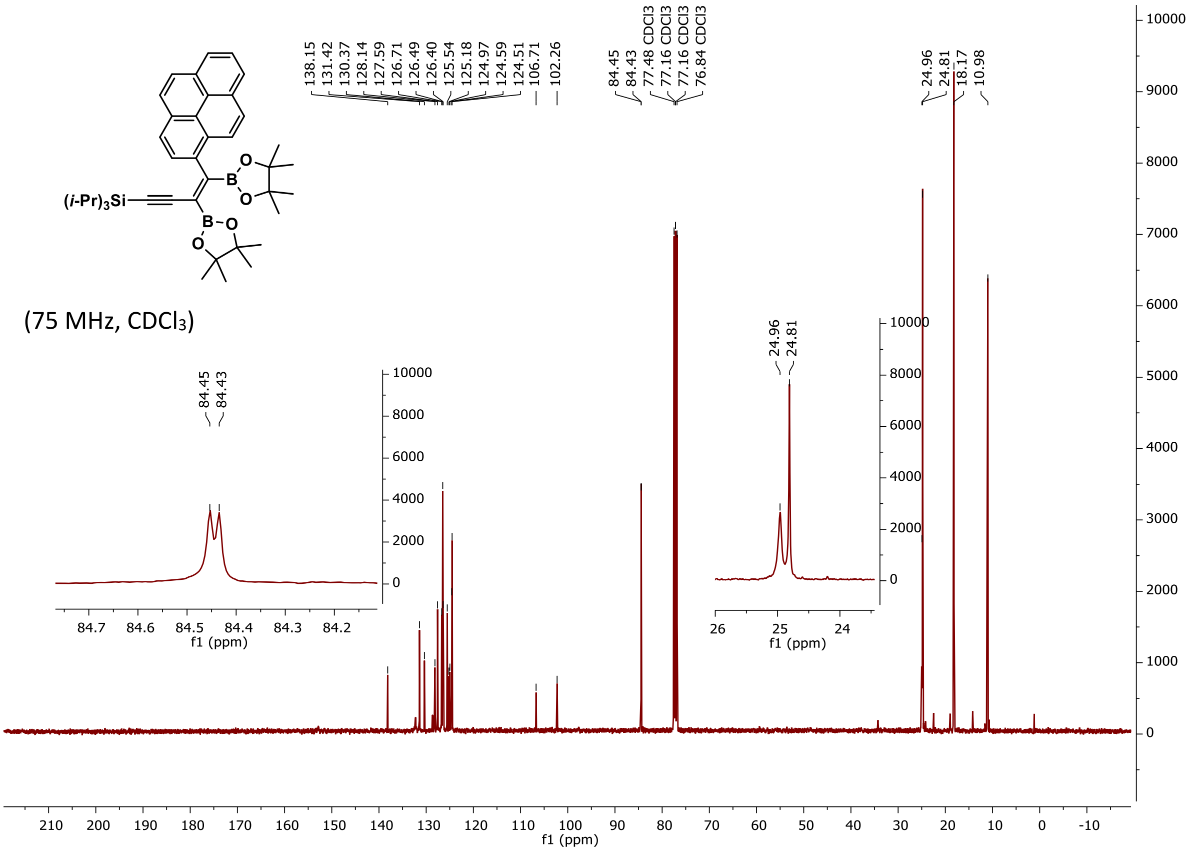


**Figure S110**. $^{13}$C NMR spectrum of **3q**.

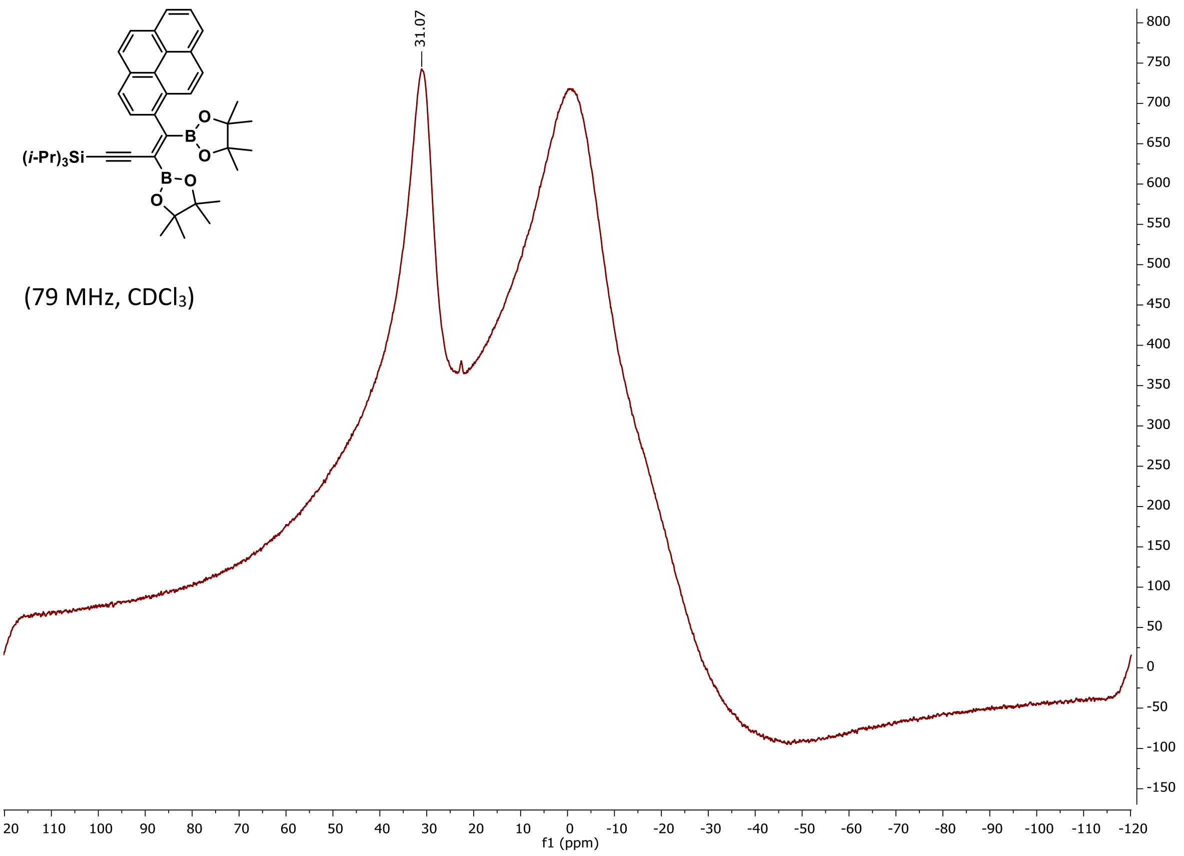


**Figure S111**. $^{11}B$ NMR spectrum of **3q**.

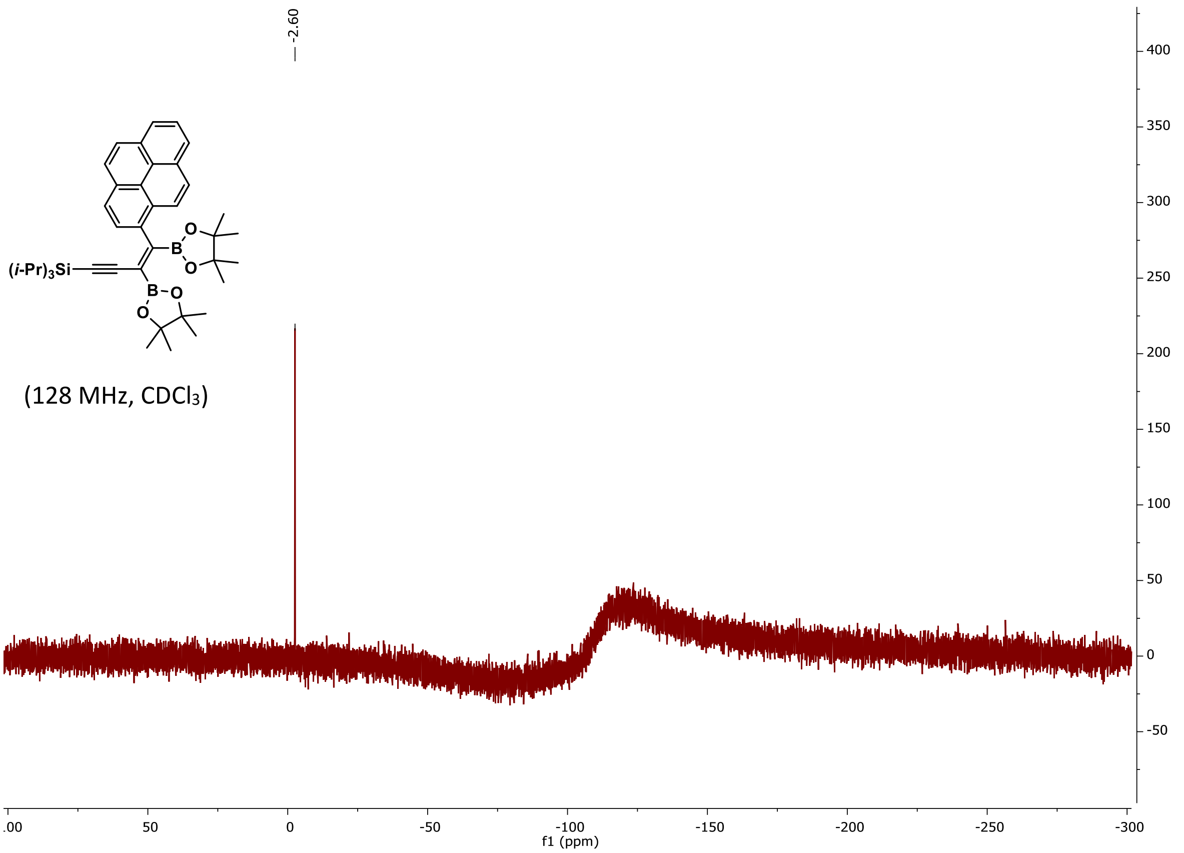


**Figure S112**. $^{29}Si$ NMR spectrum of **3q**.

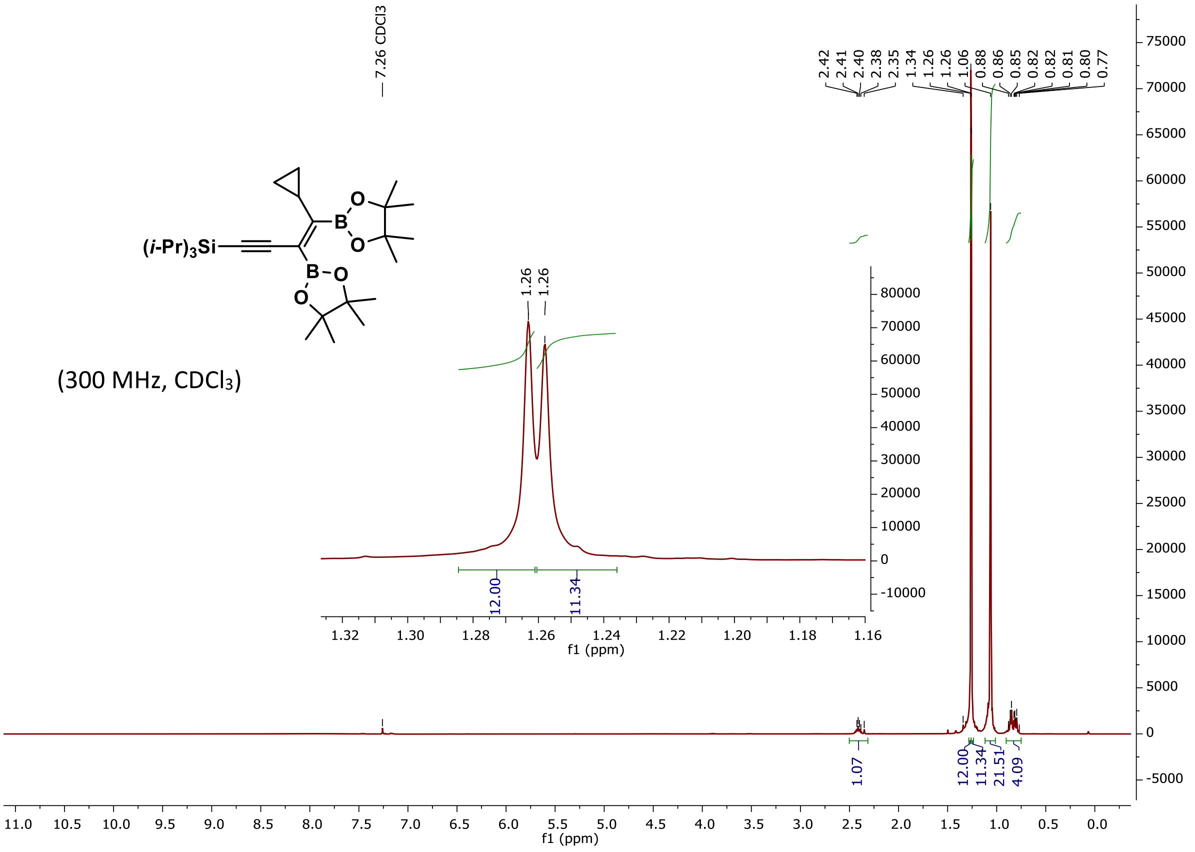


**Figure S113**. $^{1}$H NMR spectrum of (**3r**)

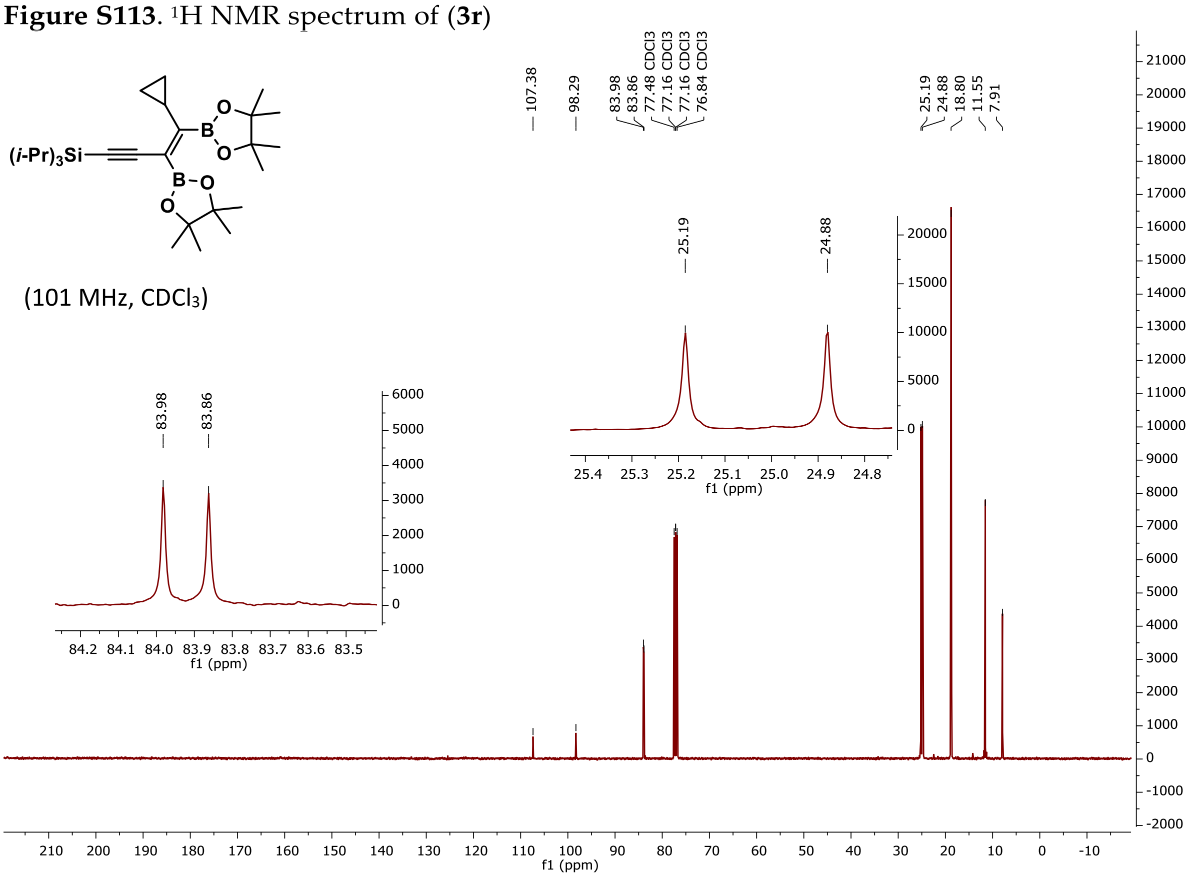


**Figure S114**. $^{11}$C NMR spectrum of (**3r**)

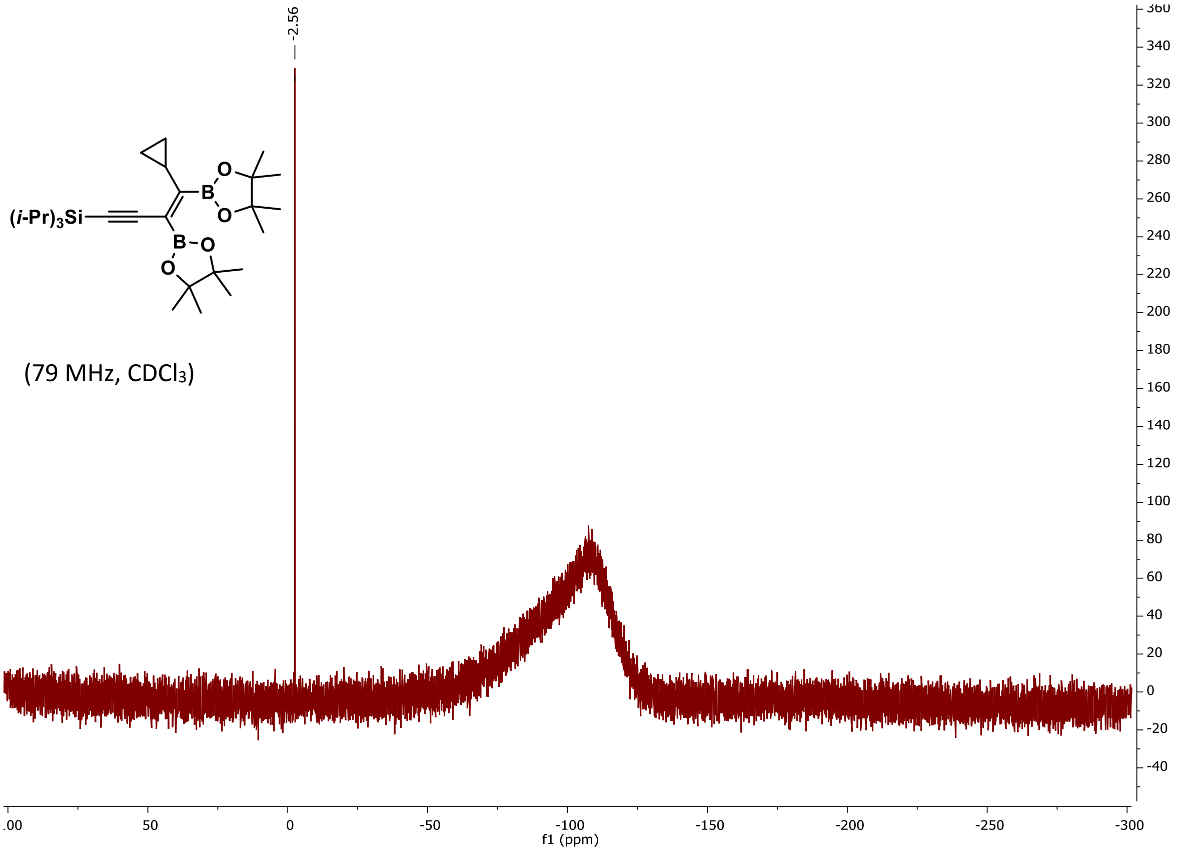


**Figure S115**. [11]C NMR spectrum of (**3r**)

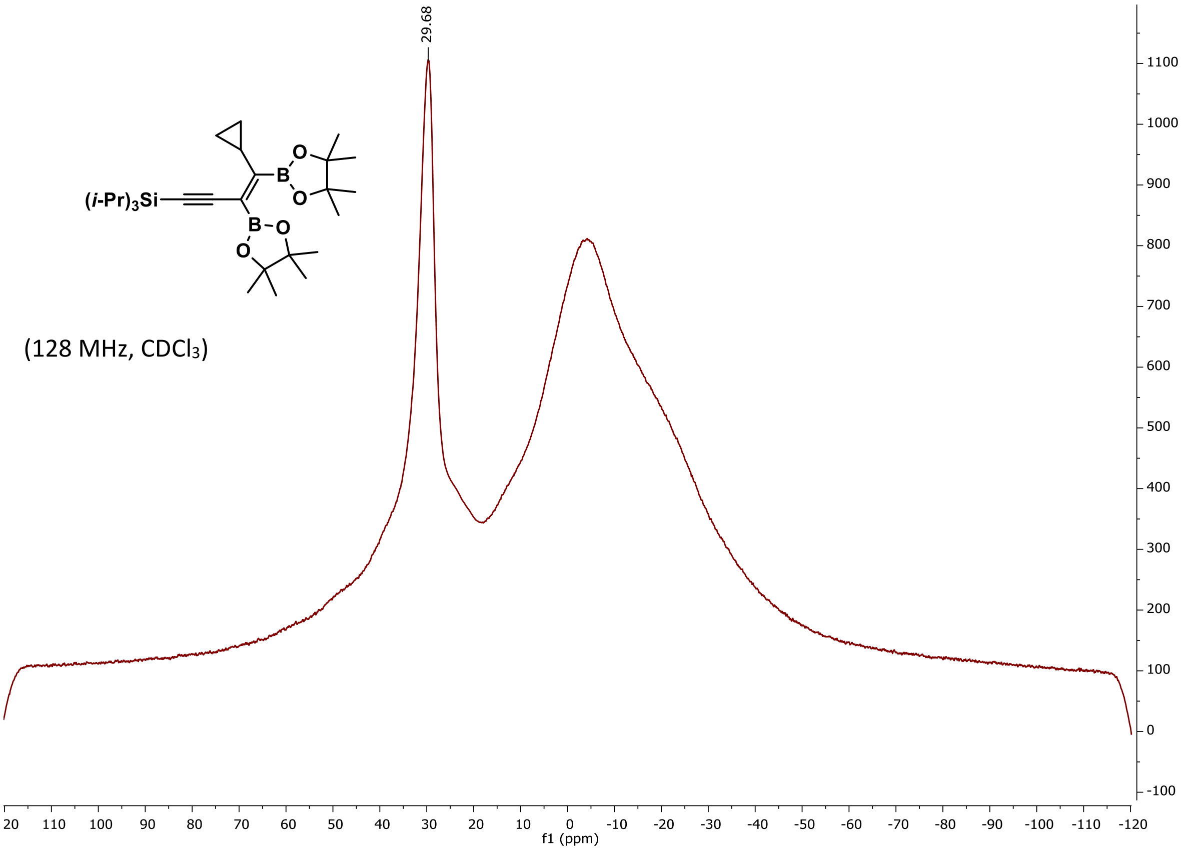


**Figure S116**. $^{29}$Si NMR spectrum of (**3r**)

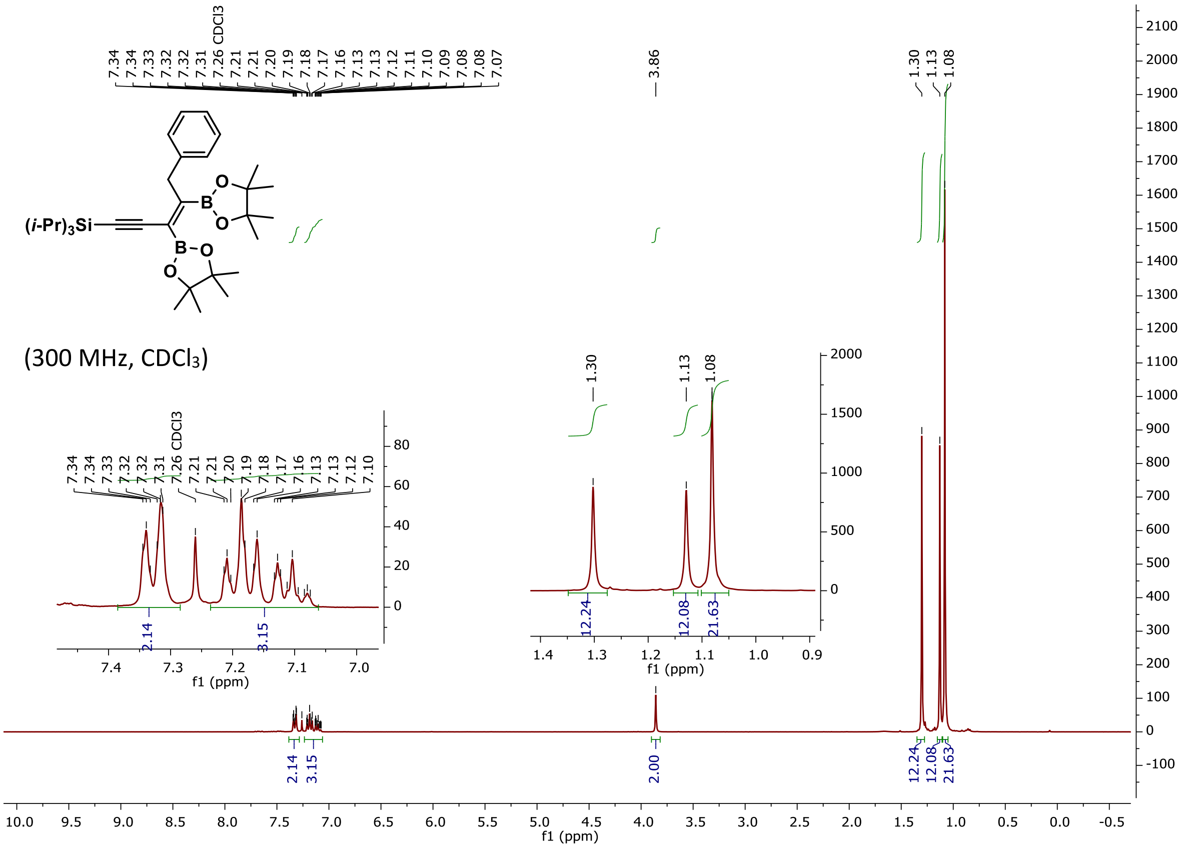


**Figure S117**. $^{1}$H NMR spectrum of **3s**.

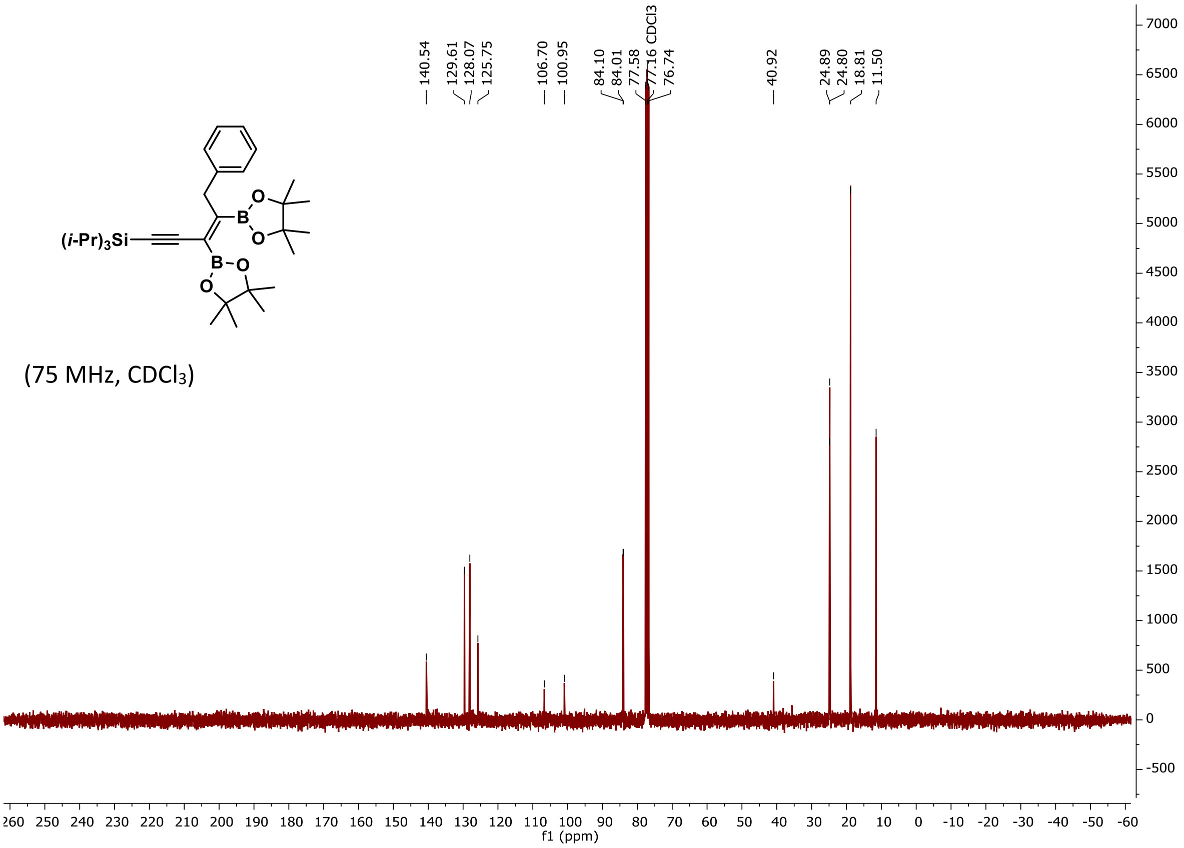


**Figure S118**. $^{11}$C NMR spectrum of **3s**.

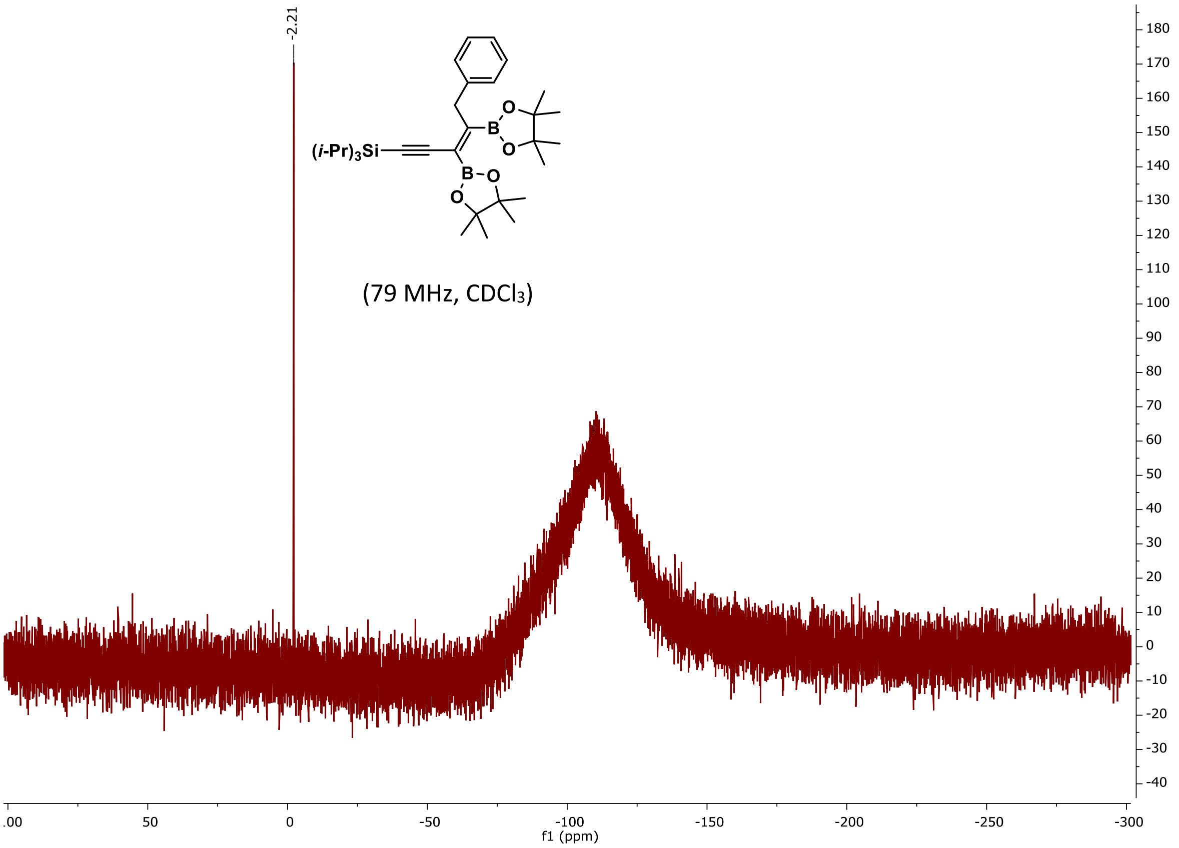


**Figure S119**. $^{29}$Si NMR spectrum of **3s**.

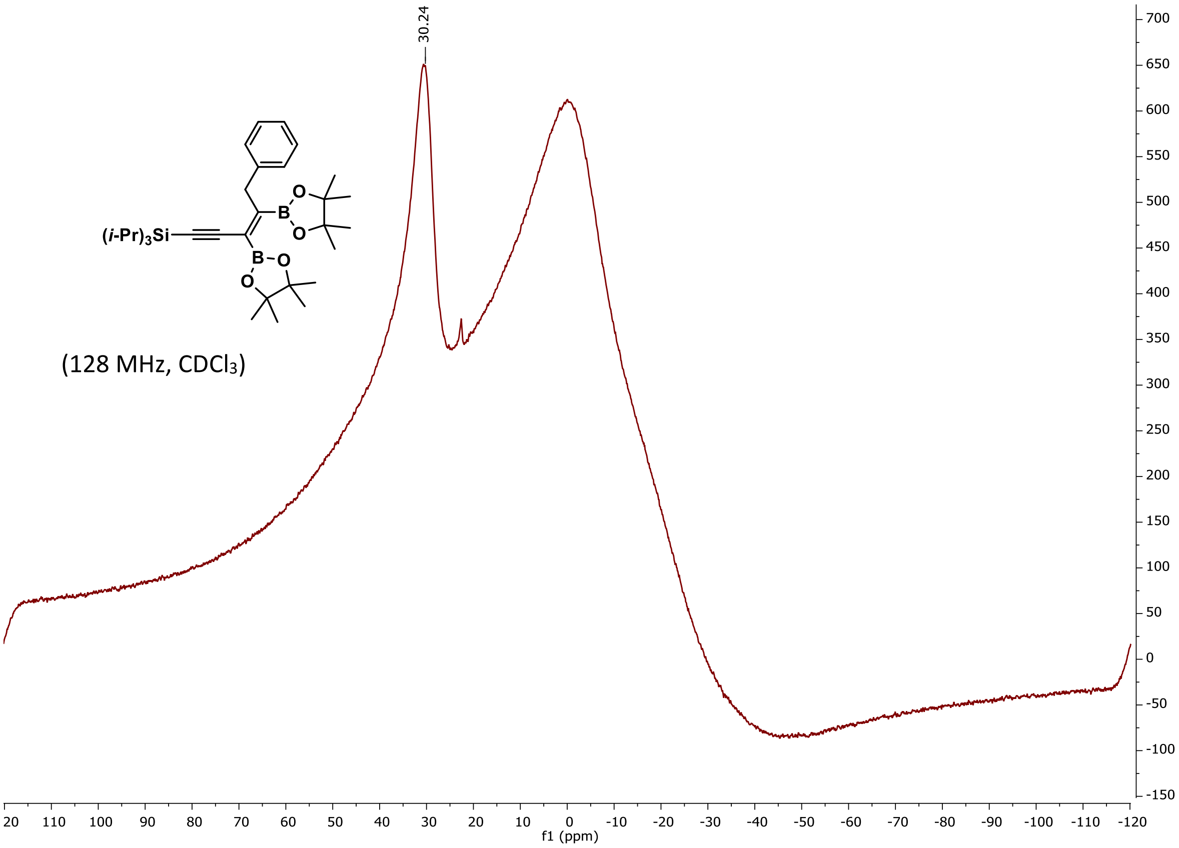


**Figure S120**. $^{11}$B NMR spectrum of **3s**.

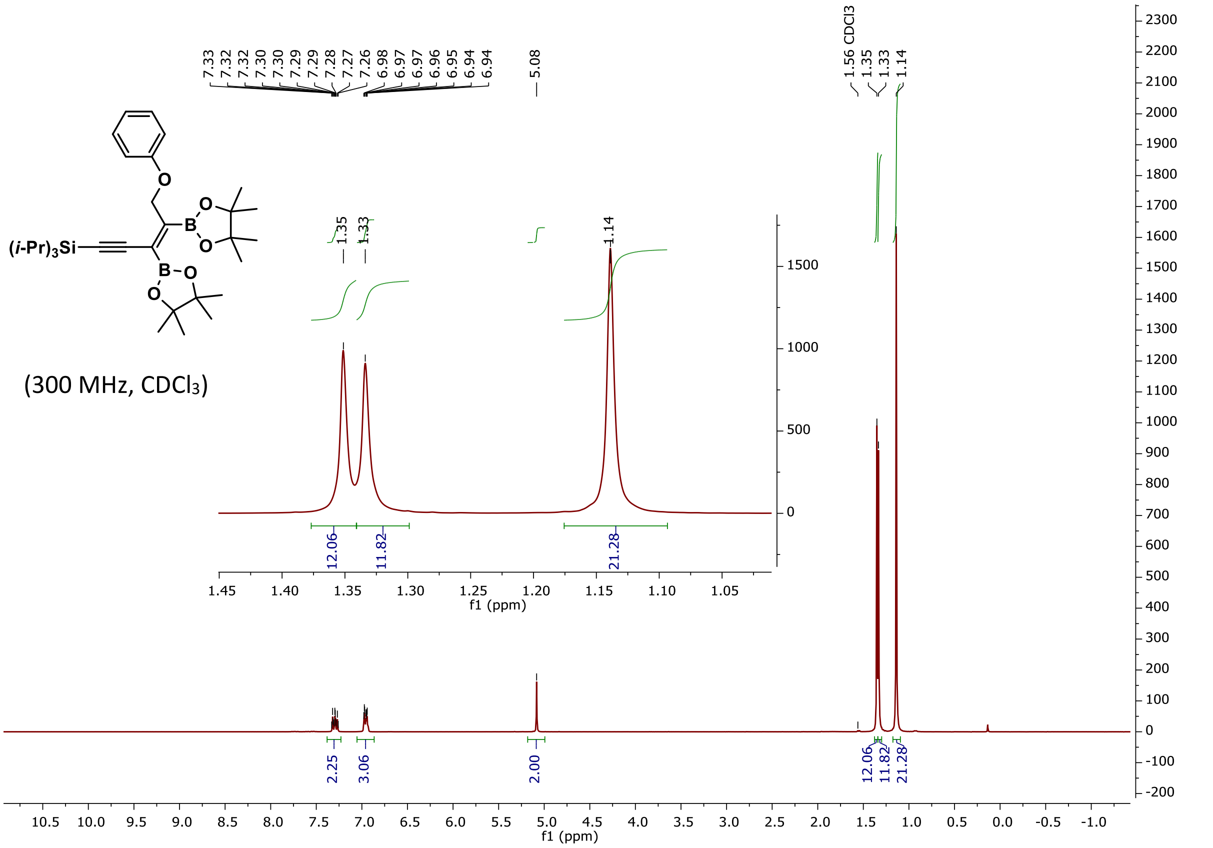


**Figure S121**. $^{1}$H NMR spectrum of **3t**.

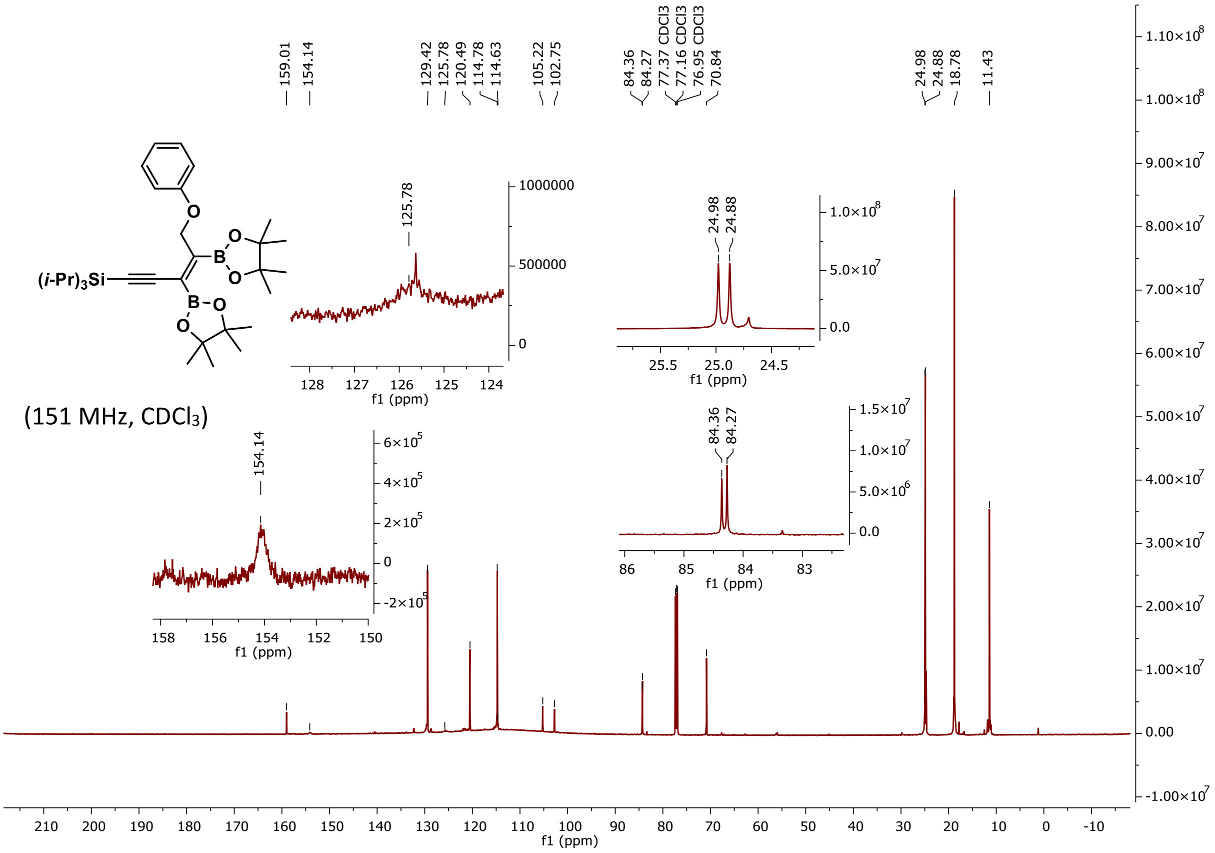


**Figure S122**. [11]C NMR spectrum of **3t**.

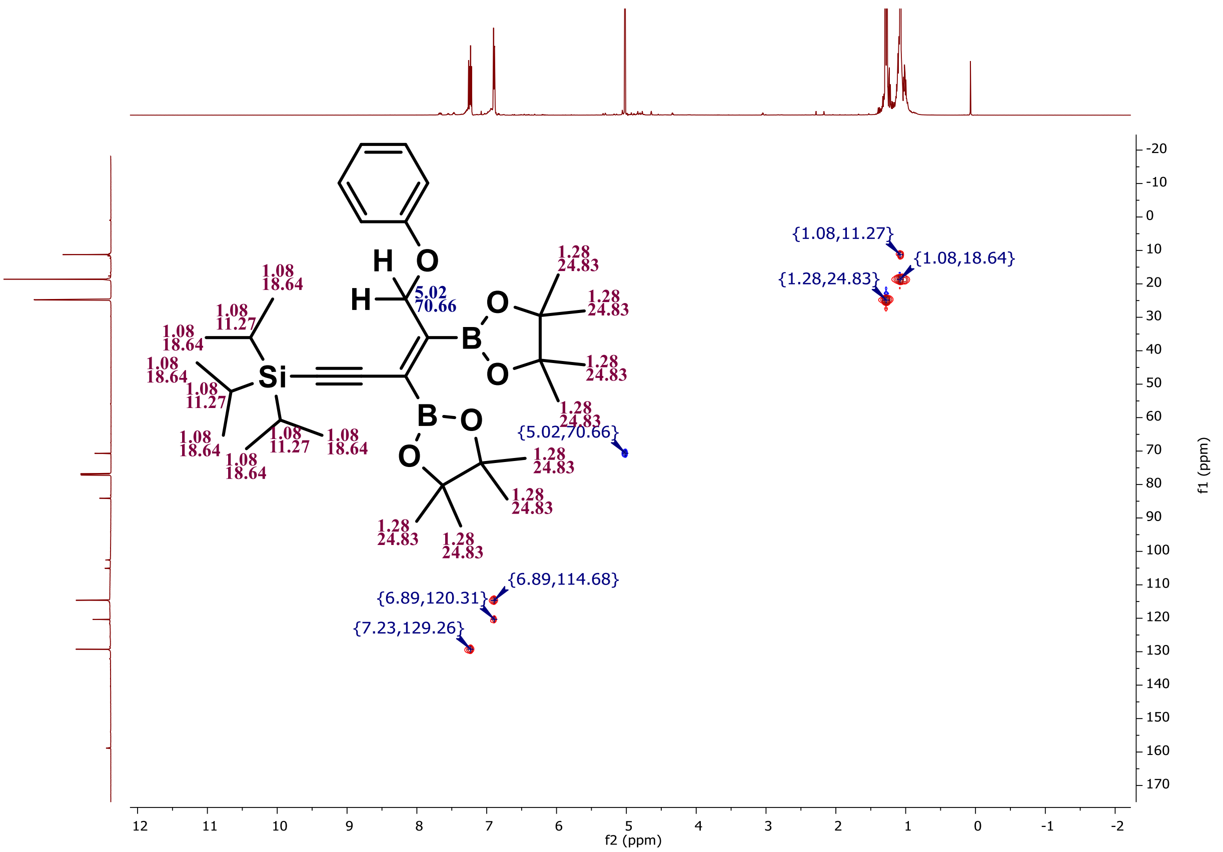


**Figure S123**. $^{1}$H-$^{13}$C HSQC NMR spectrum of **3t**.

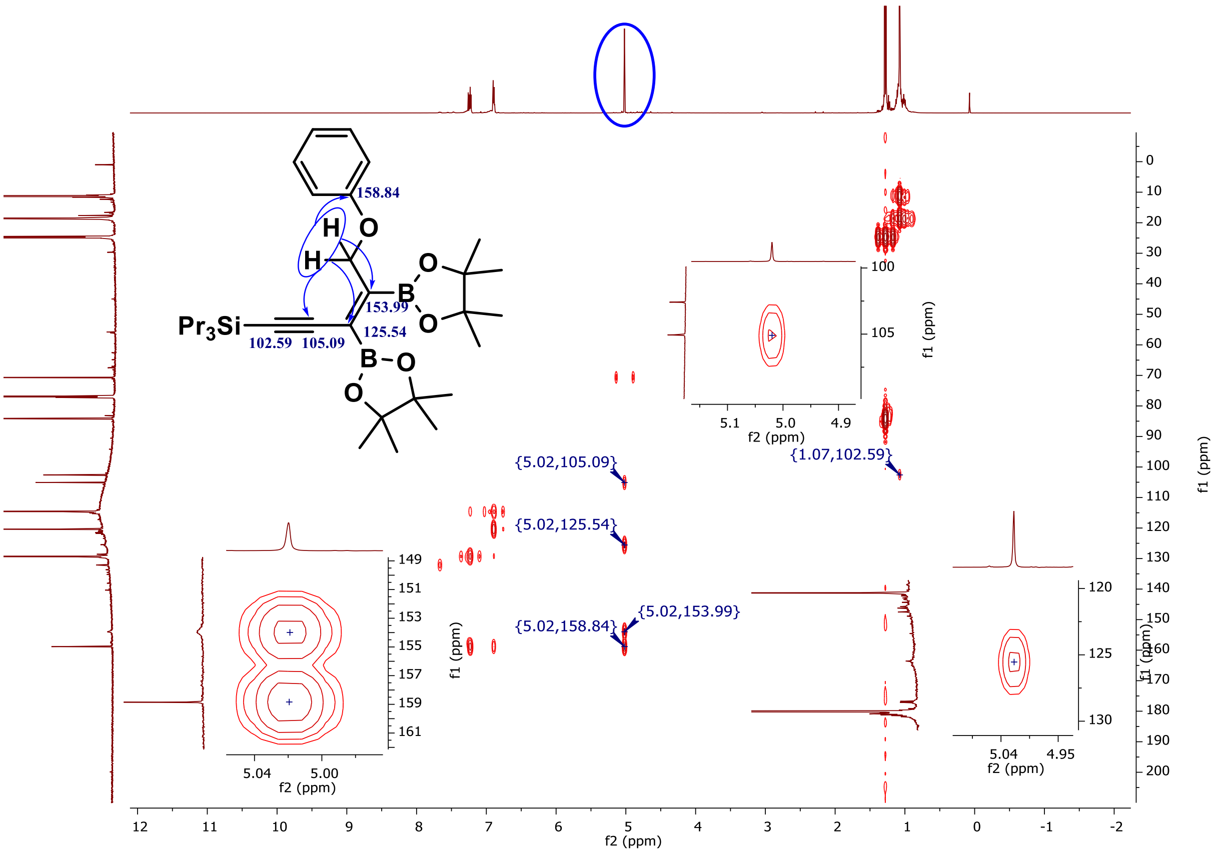


**Figure S124**. $^{1}$H-$^{13}$C HMBC NMR spectrum of **3t**.

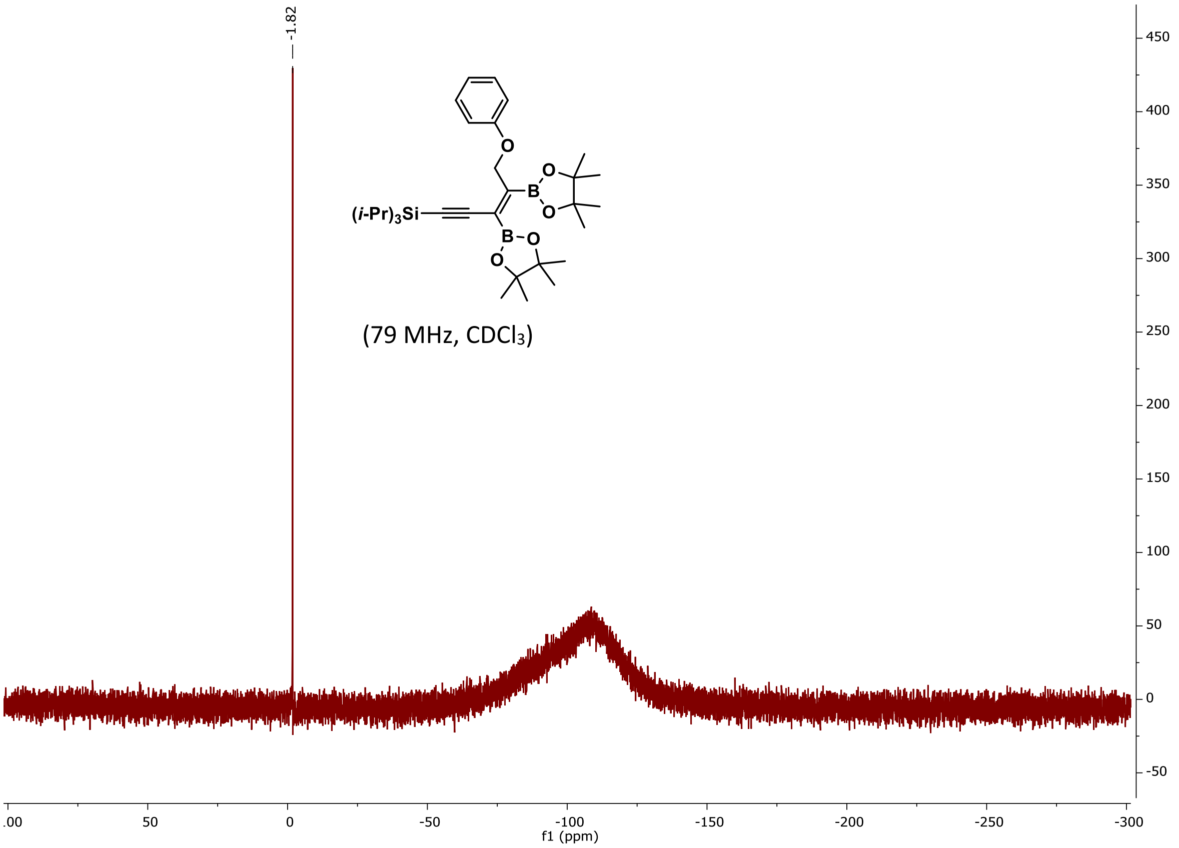


**Figure S125**. $^{29}$Si NMR spectrum of **3t**.

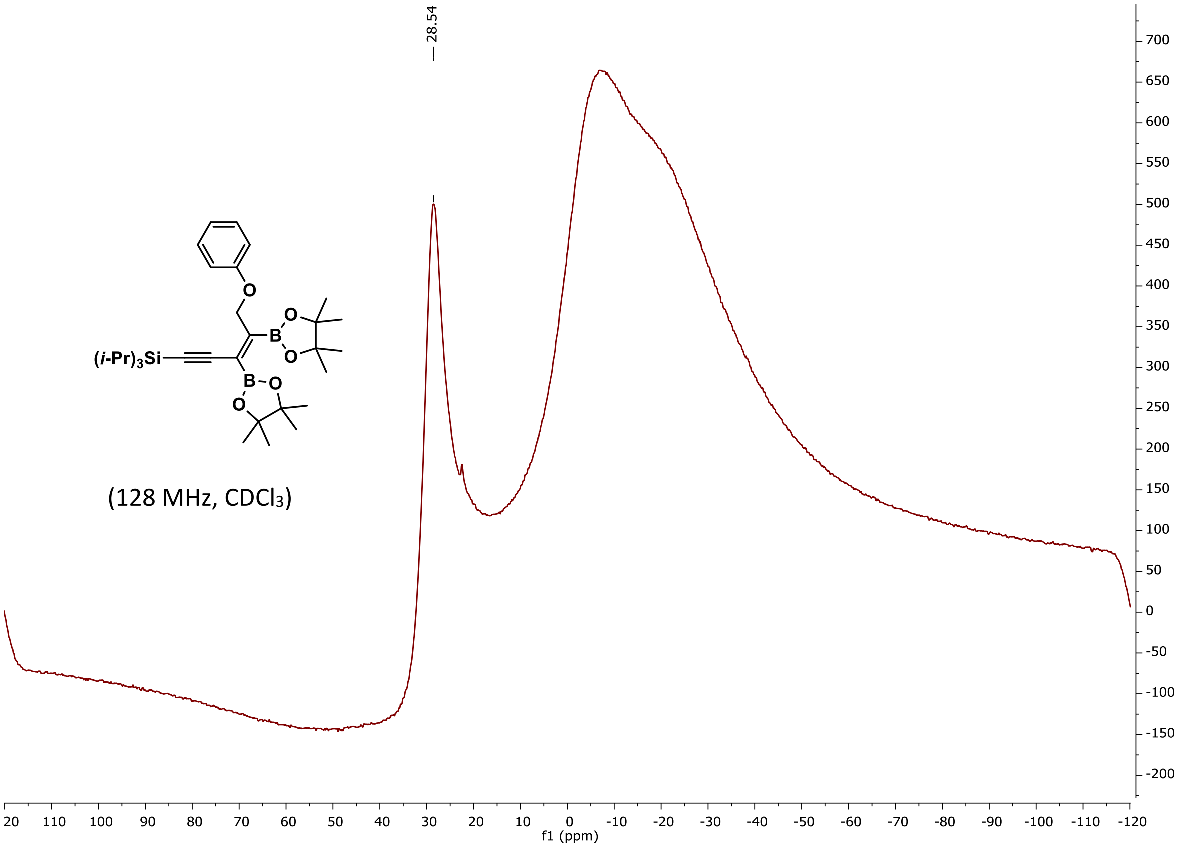


**Figure S126**. $^{11}$B NMR spectrum of **3t**.

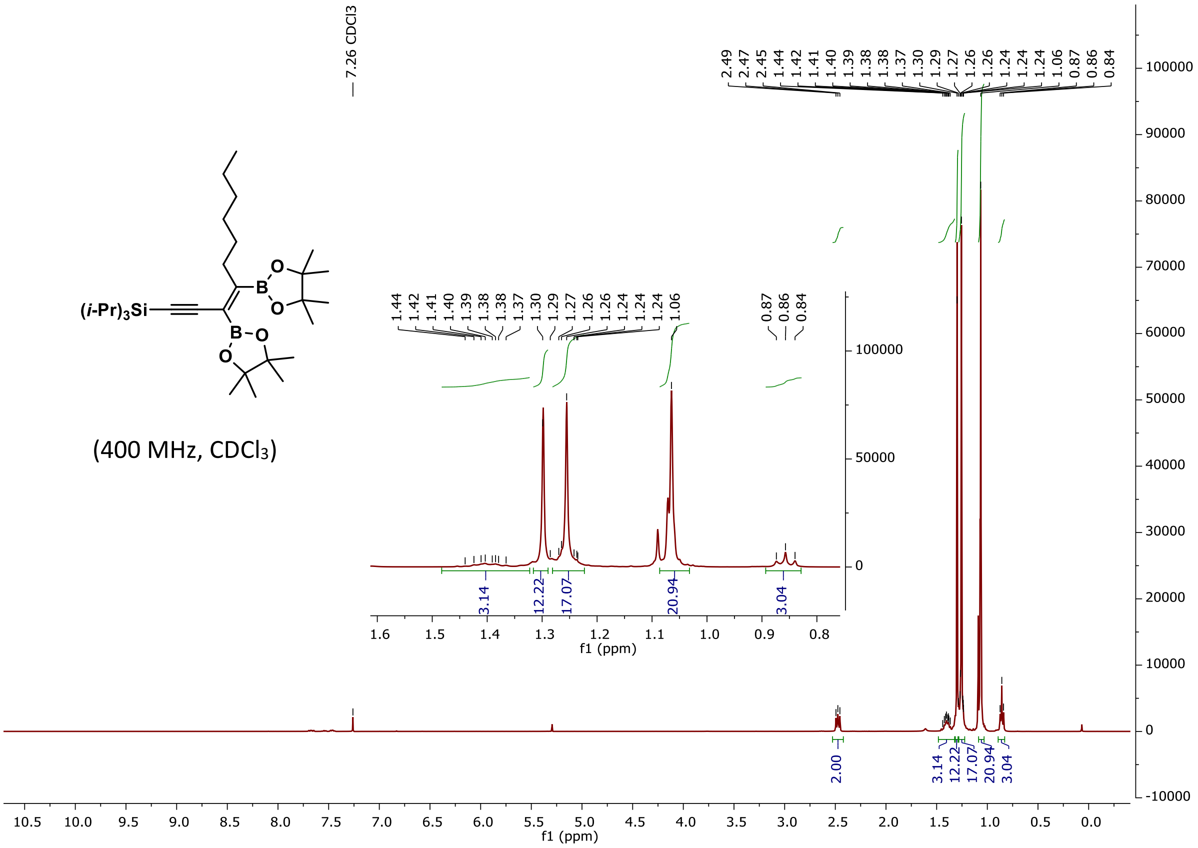


**Figure S127**. $^{1}$H NMR spectrum of **3u**.

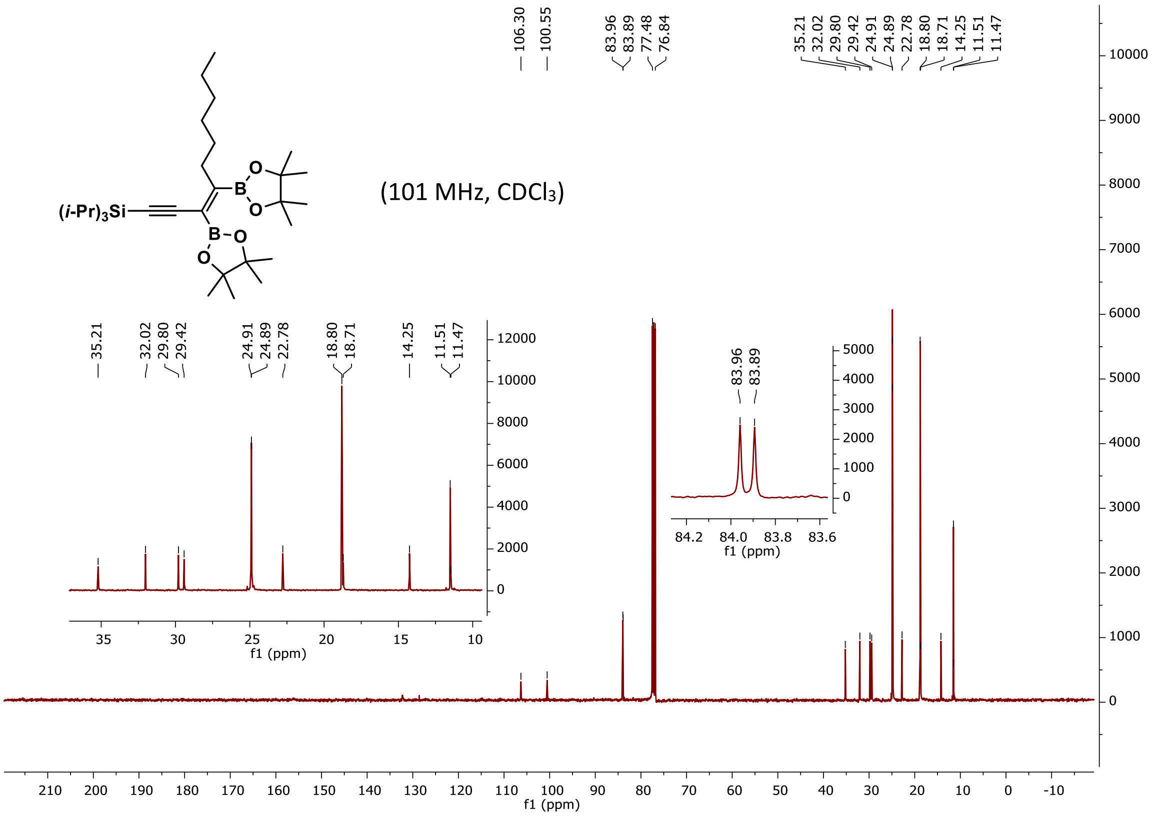


**Figure S128**. $^{13}$C NMR spectrum of **3u**.

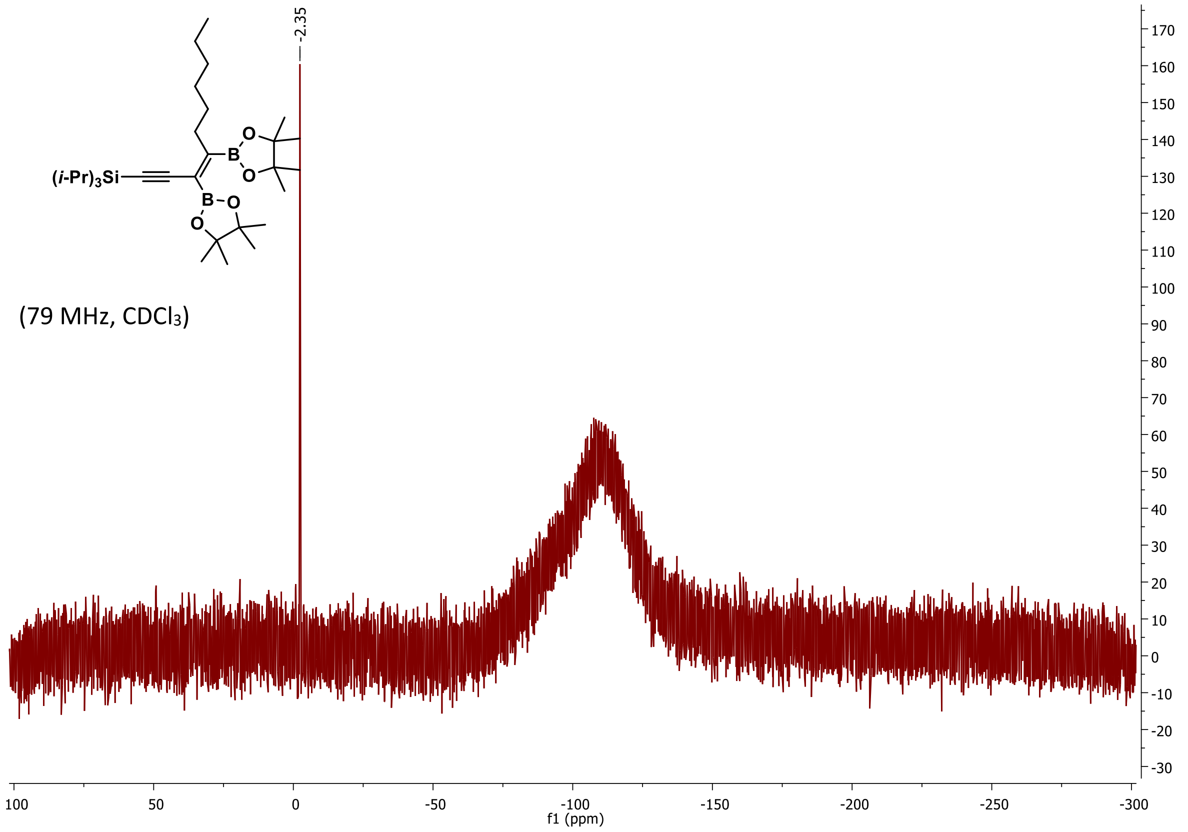


**Figure S129**. $^{29}$Si NMR spectrum of **3u**.

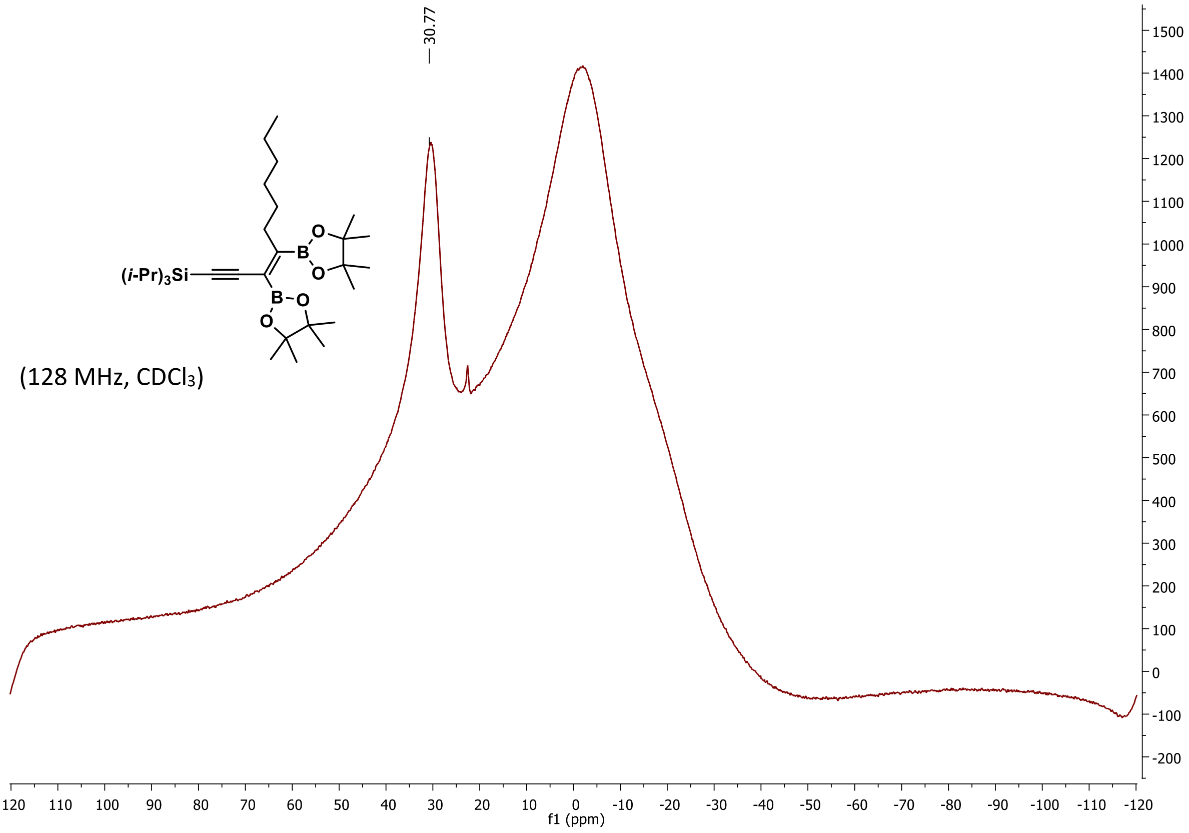


**Figure S130**. $^{11}$B NMR spectrum of **3u**.

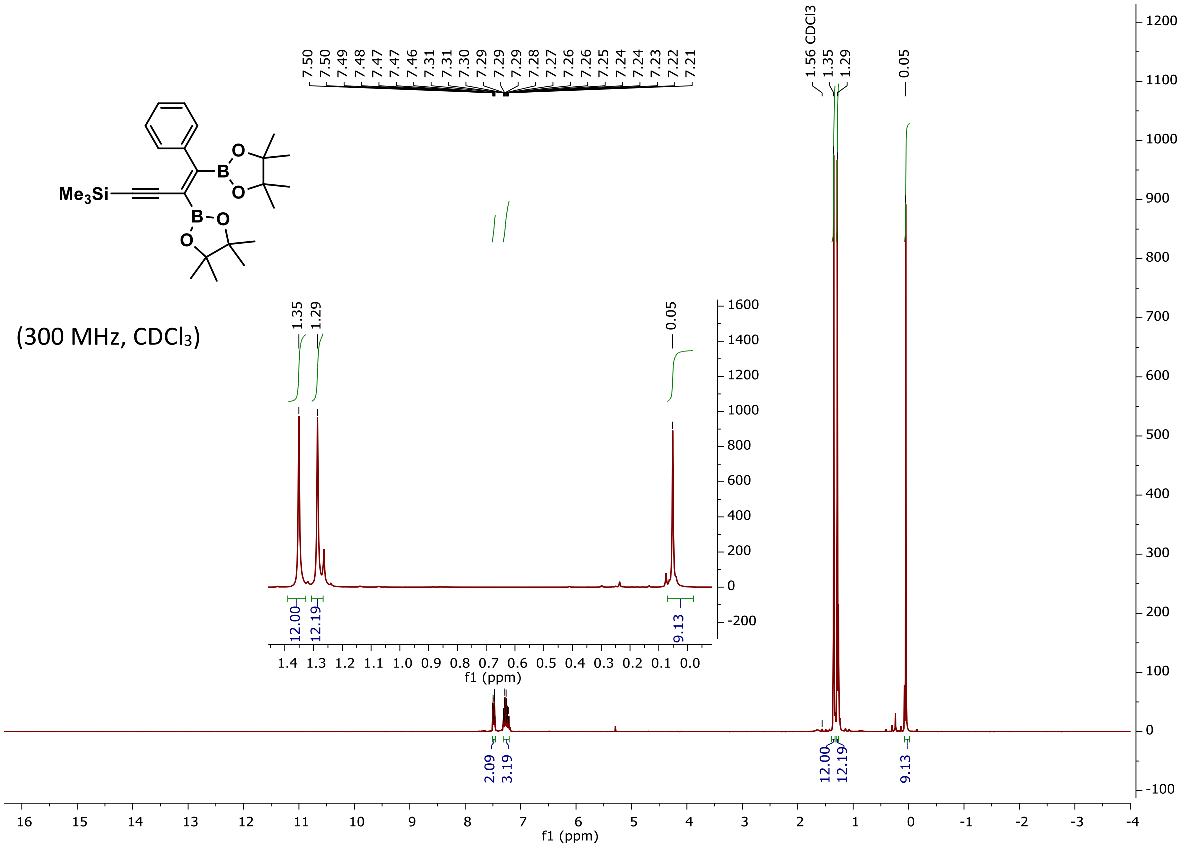


**Figure S131**. $^{1}$H NMR spectrum of **3v**.

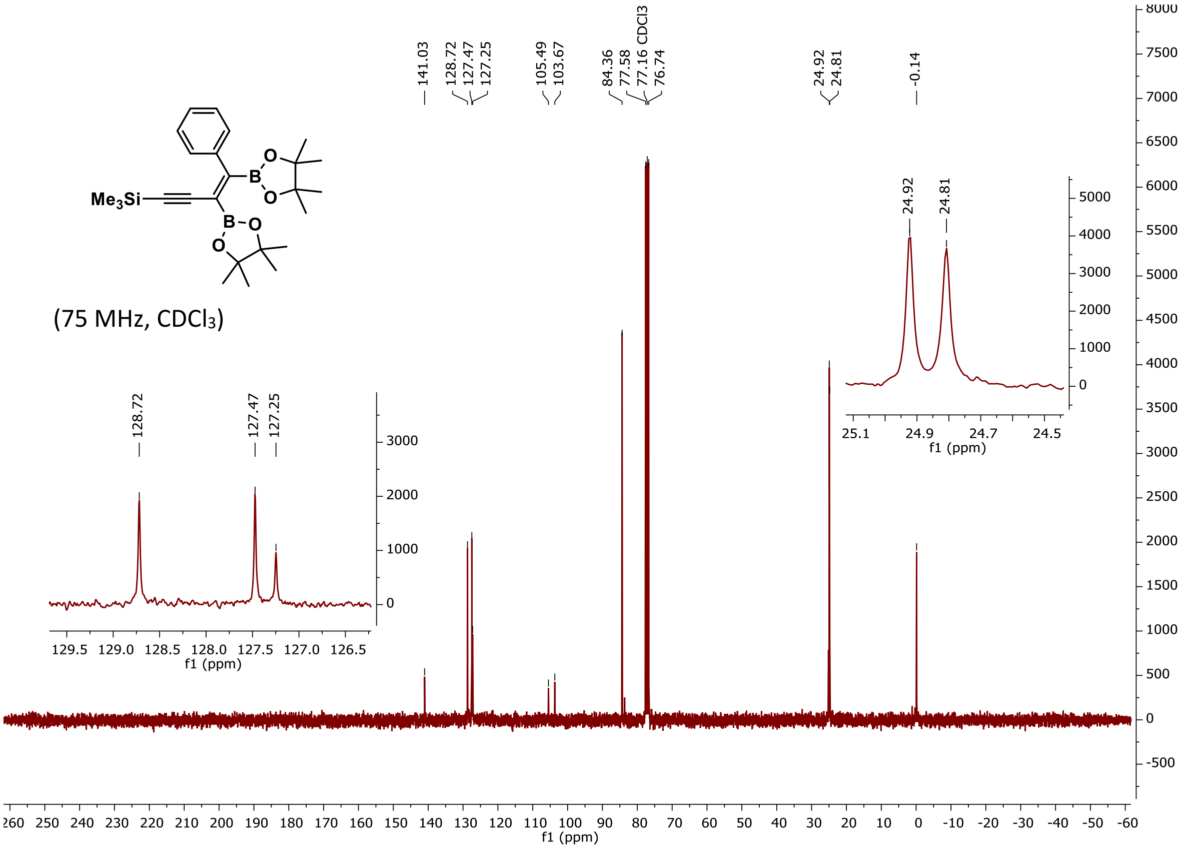


**Figure S132**. $^{13}$C NMR spectrum of **3v**.

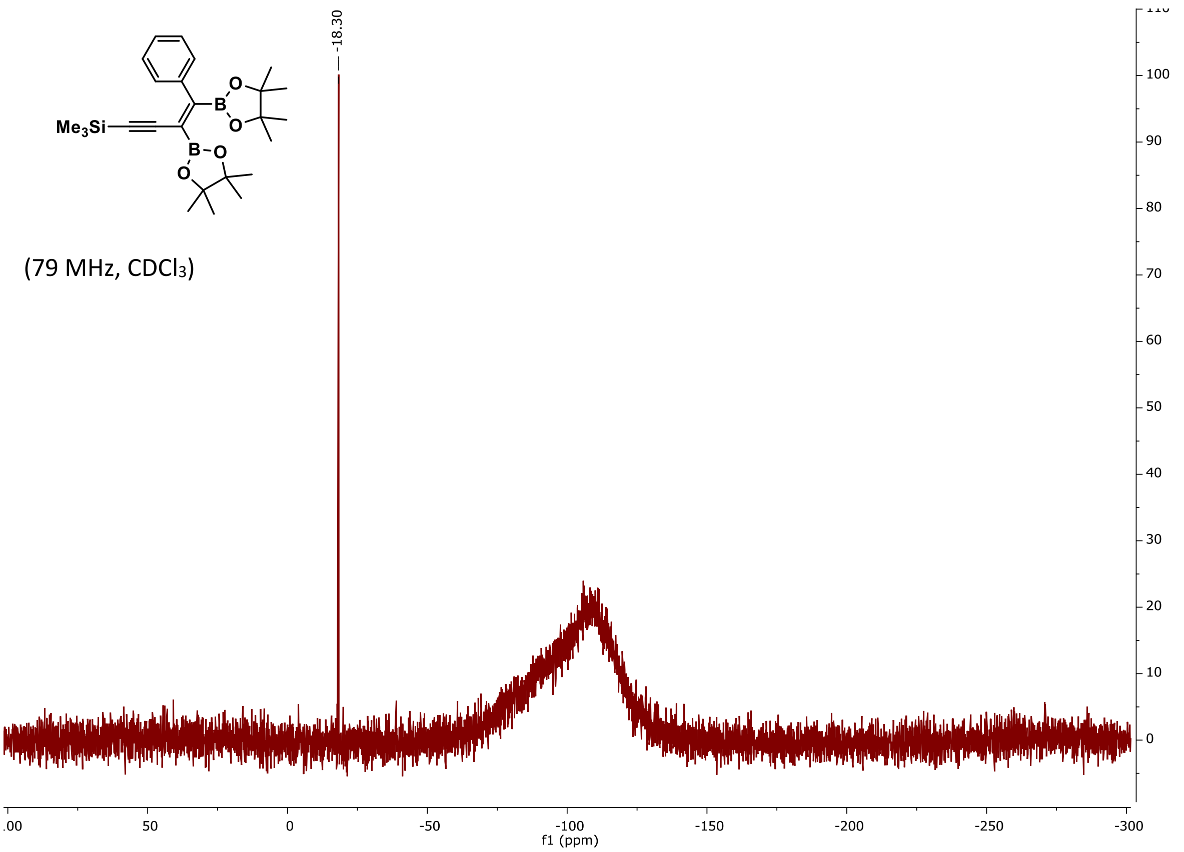


**Figure S133**. $^{29}$Si NMR spectrum of **3v**.

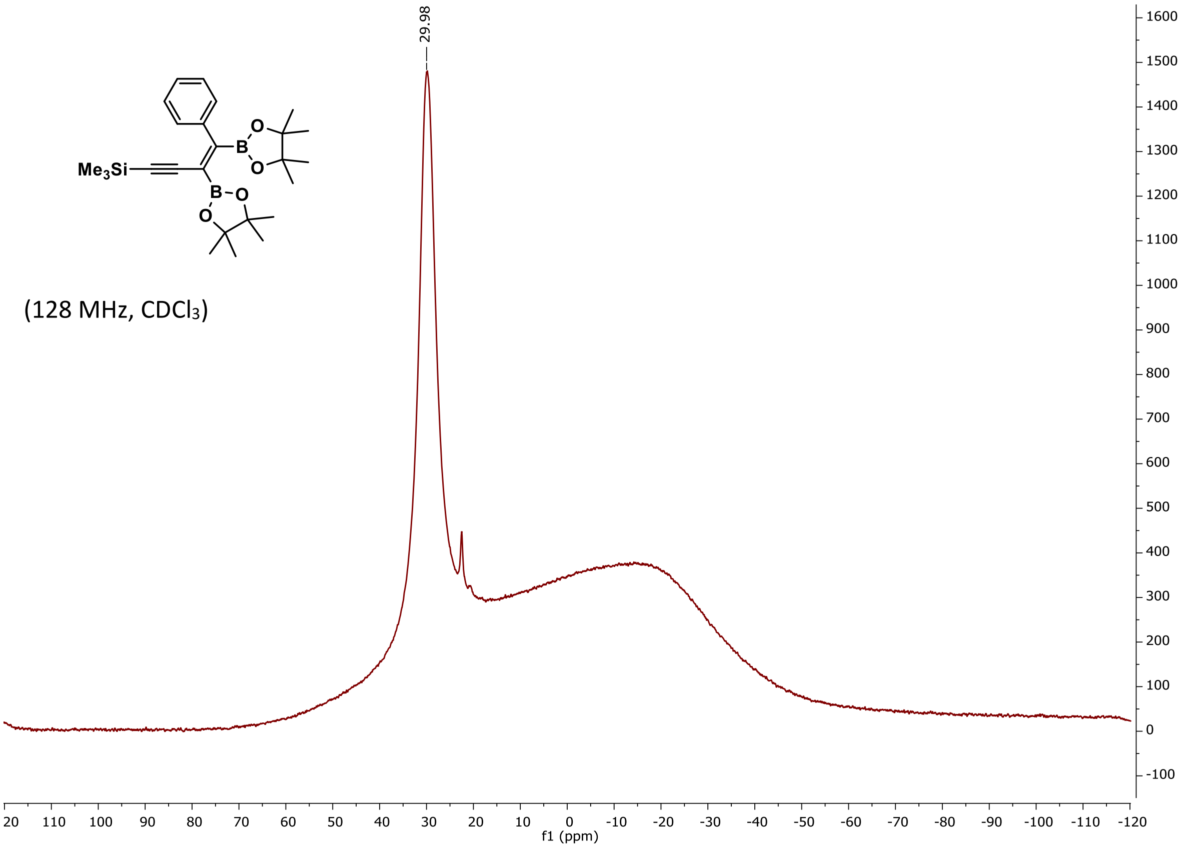


**Figure S134**. $^{11}$B NMR spectrum of **3v**.

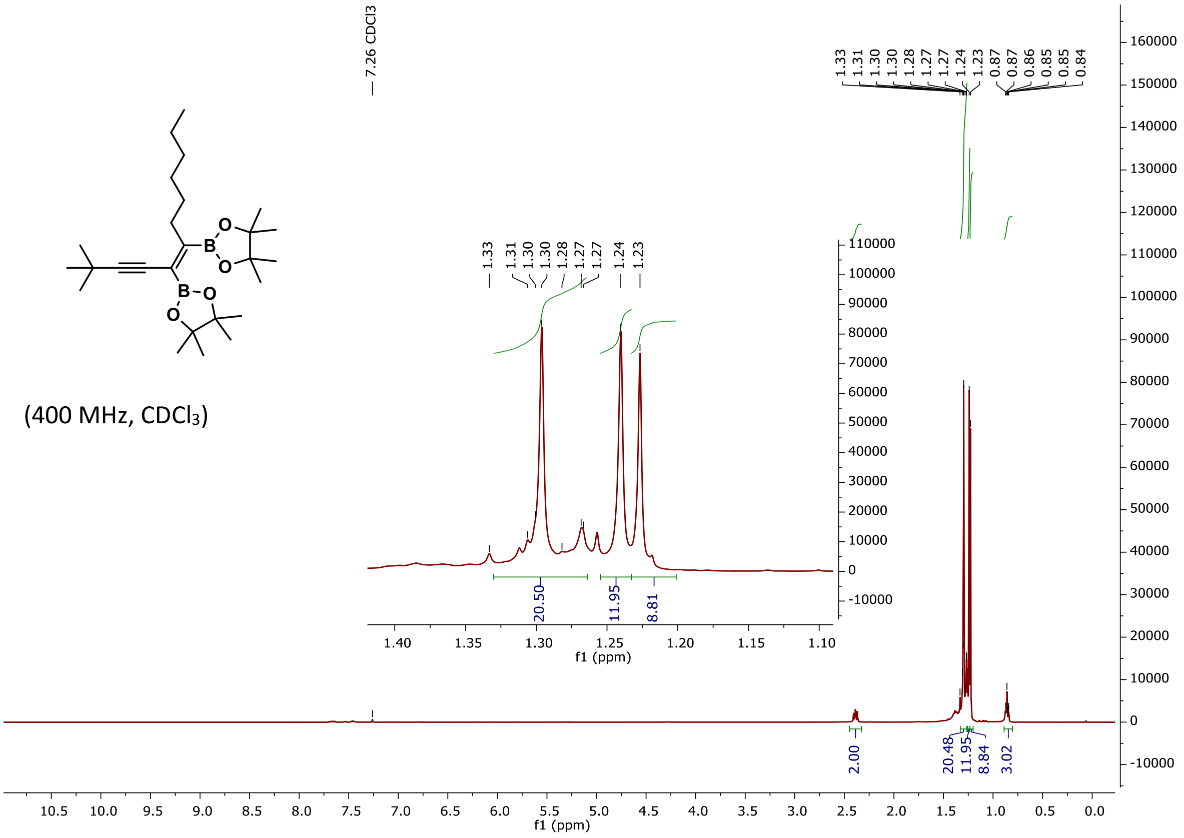


**Figure S135**. $^{1}$H NMR spectrum of **3w**.

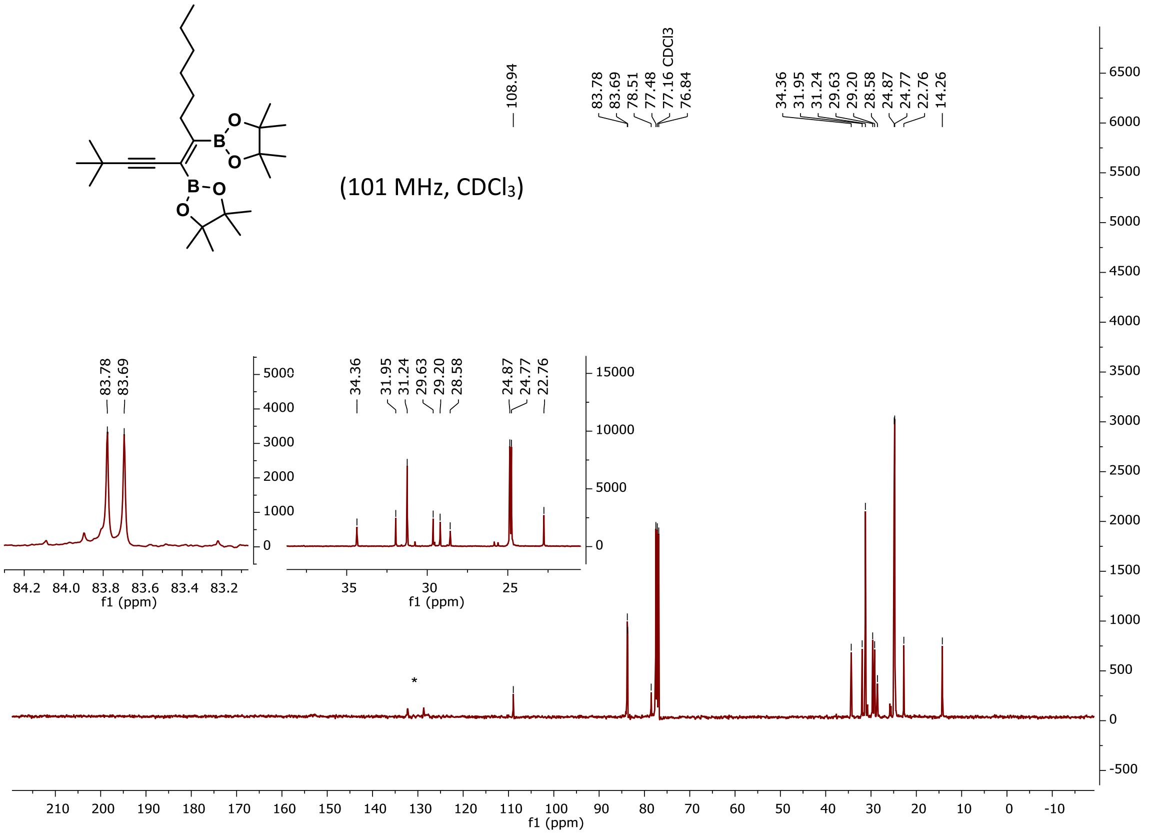


**Figure S136**. $^{13}$C NMR spectrum of **3w**. * - traces of $PPh_3$

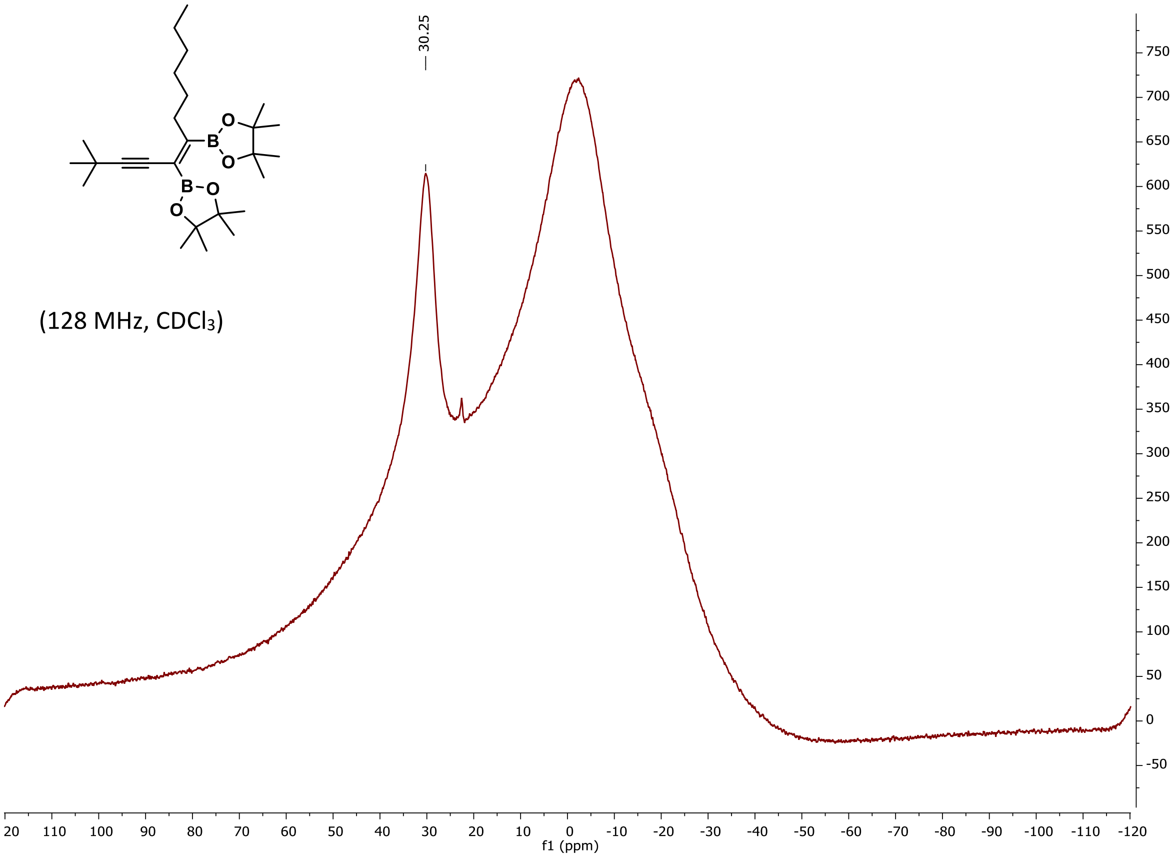


**Figure S137**. ¹¹B NMR spectrum of **3w**.

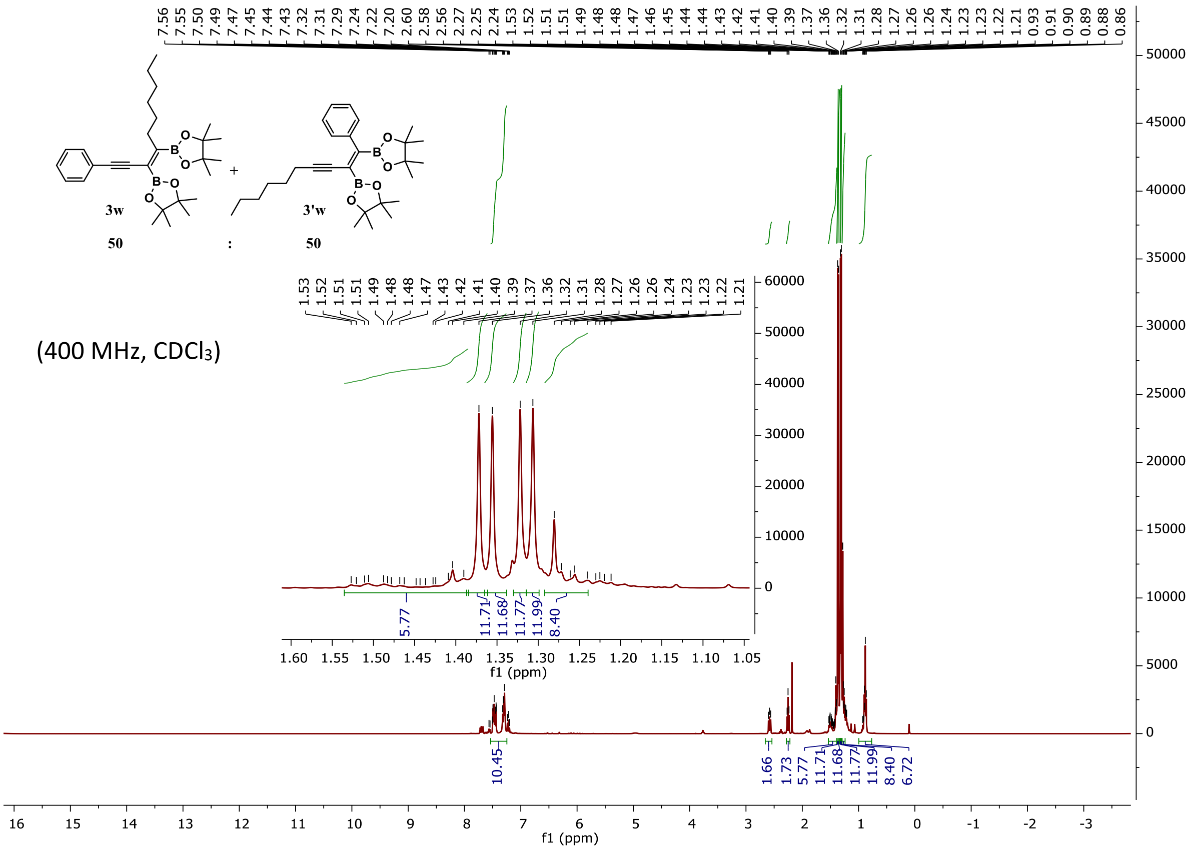


**Figure S138**. ¹H NMR spectrum of **3x/3’x**.

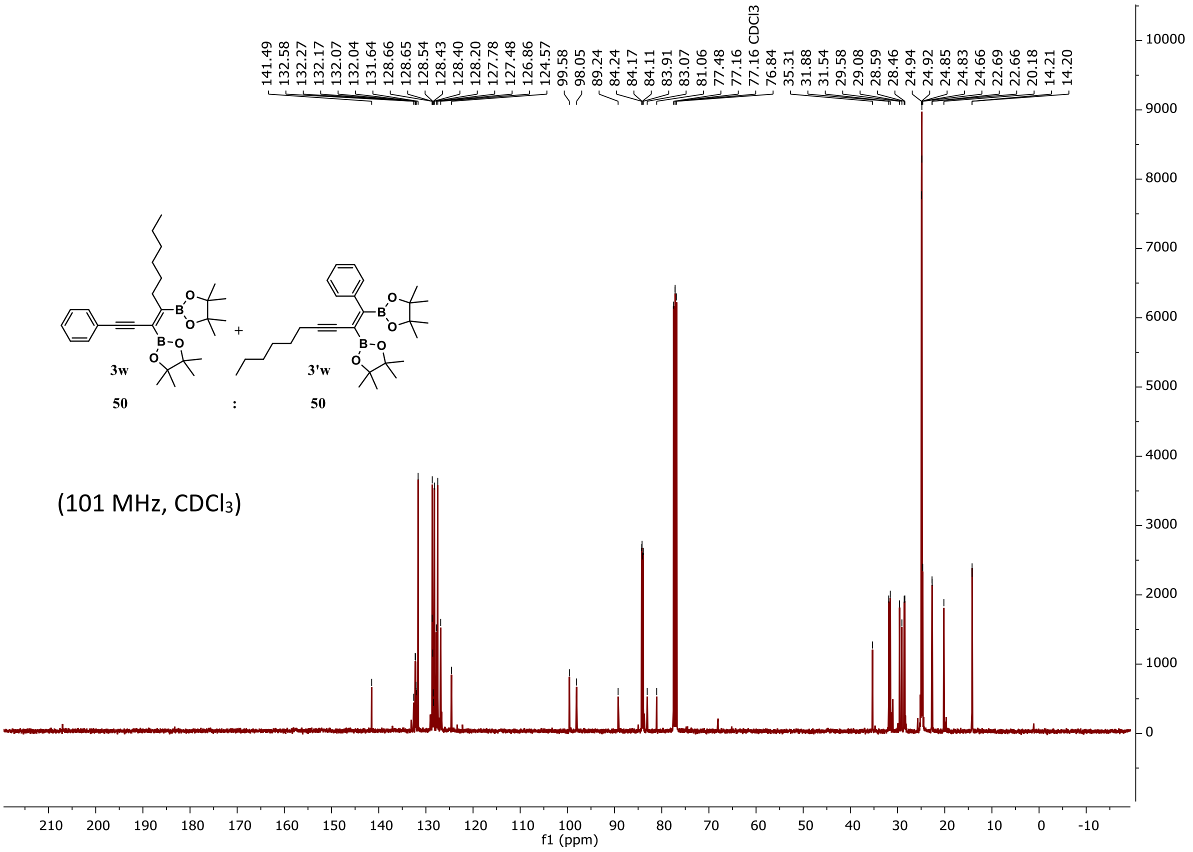

**Figure S139**. $^{13}$C NMR spectrum of **3x/3′x**.

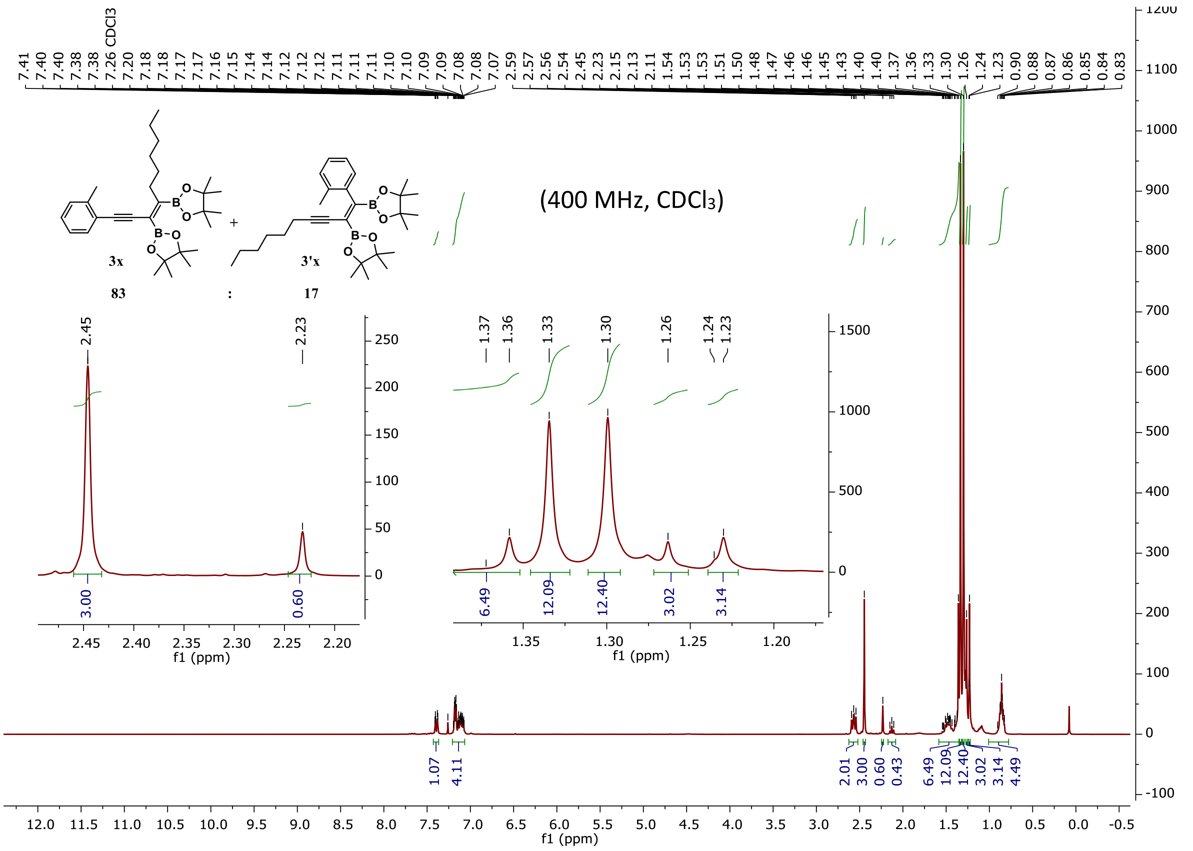

**Figure S140**. $^{1}$H NMR spectrum of **3y/3′y**.

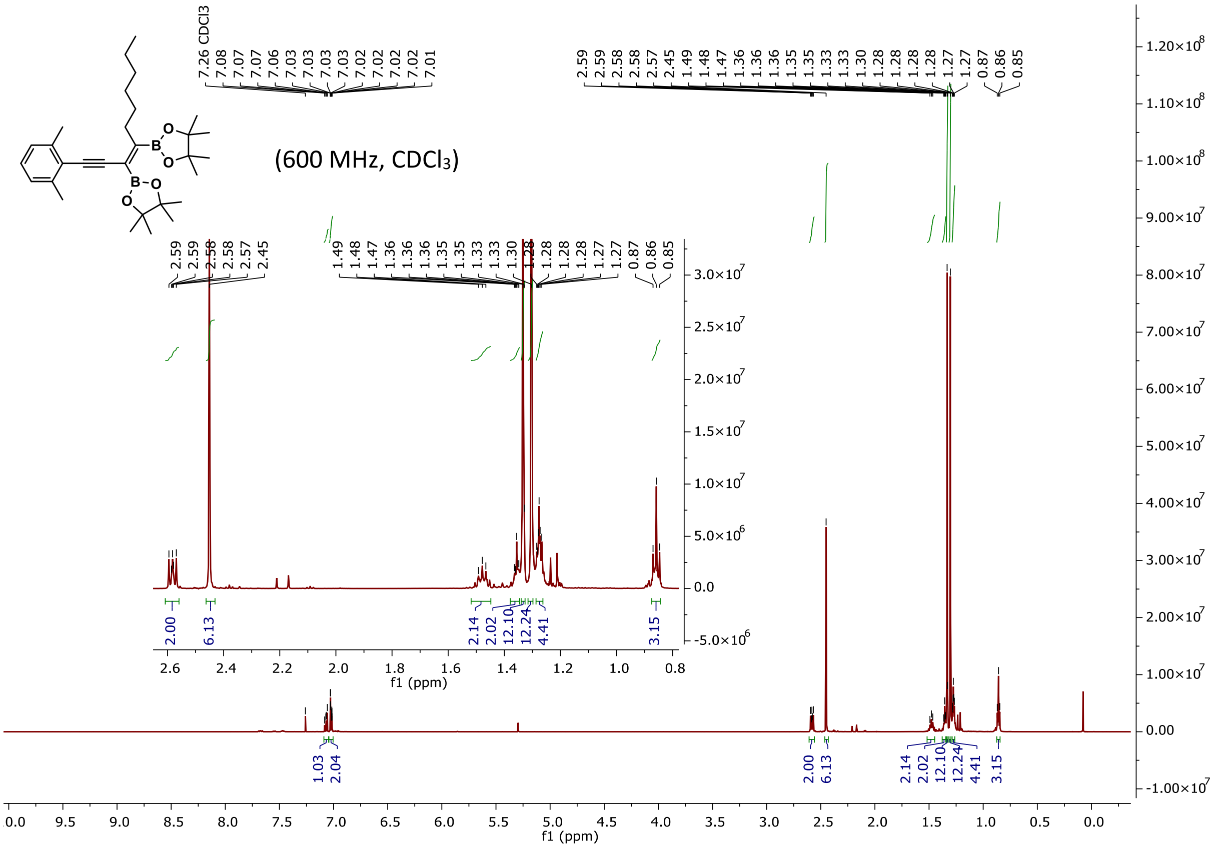

**Figure S141**. $^{1}$H NMR spectrum of **3z.**

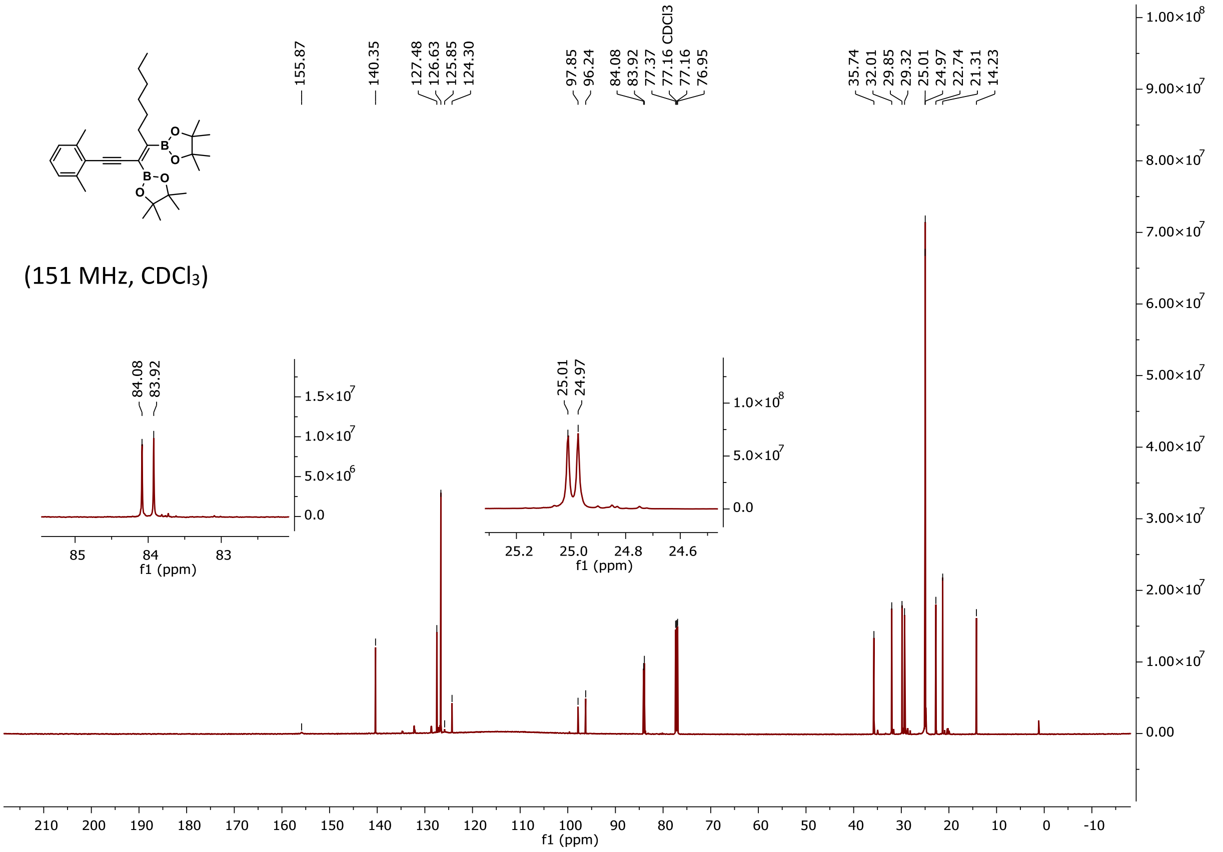

**Figure S142**. $^{13}$C NMR spectrum of **3z.**

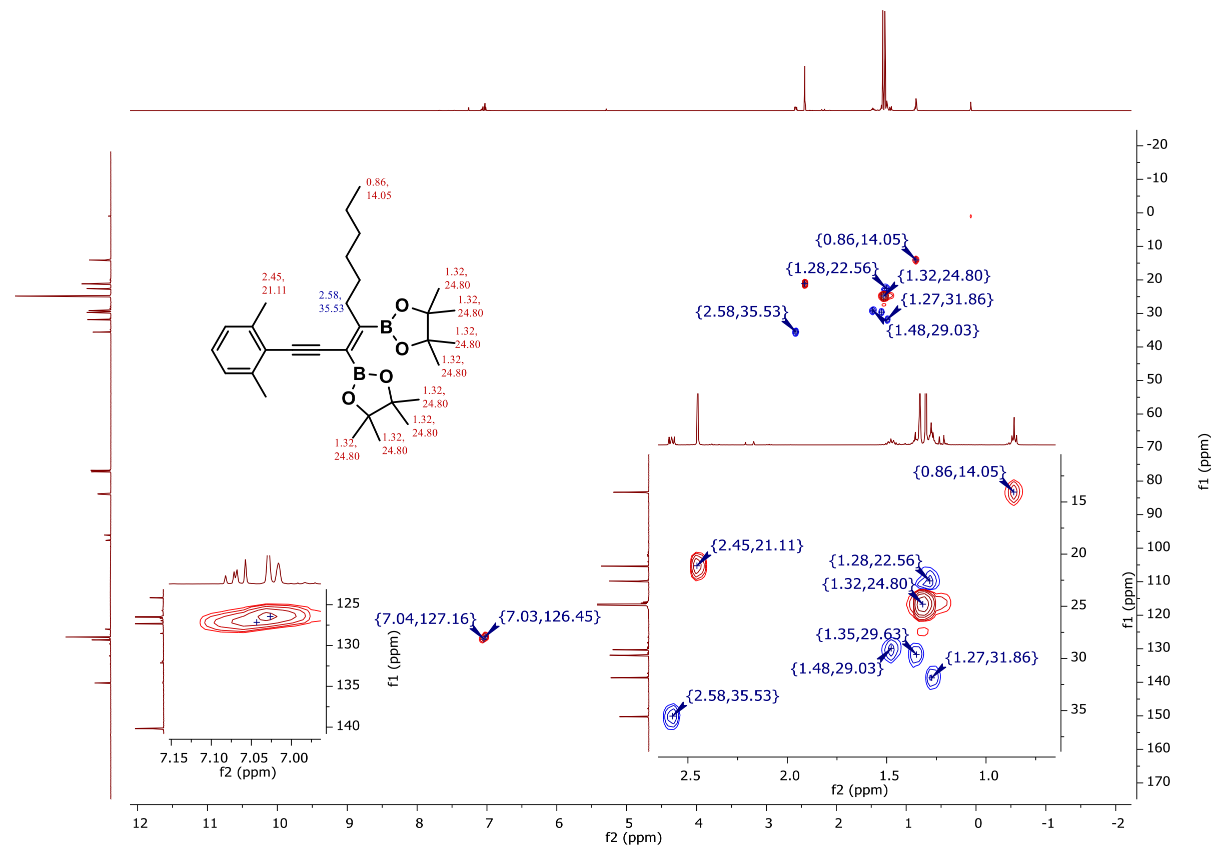

**Figure S143**. ¹H-¹³C HSQC NMR spectrum of **3z.**

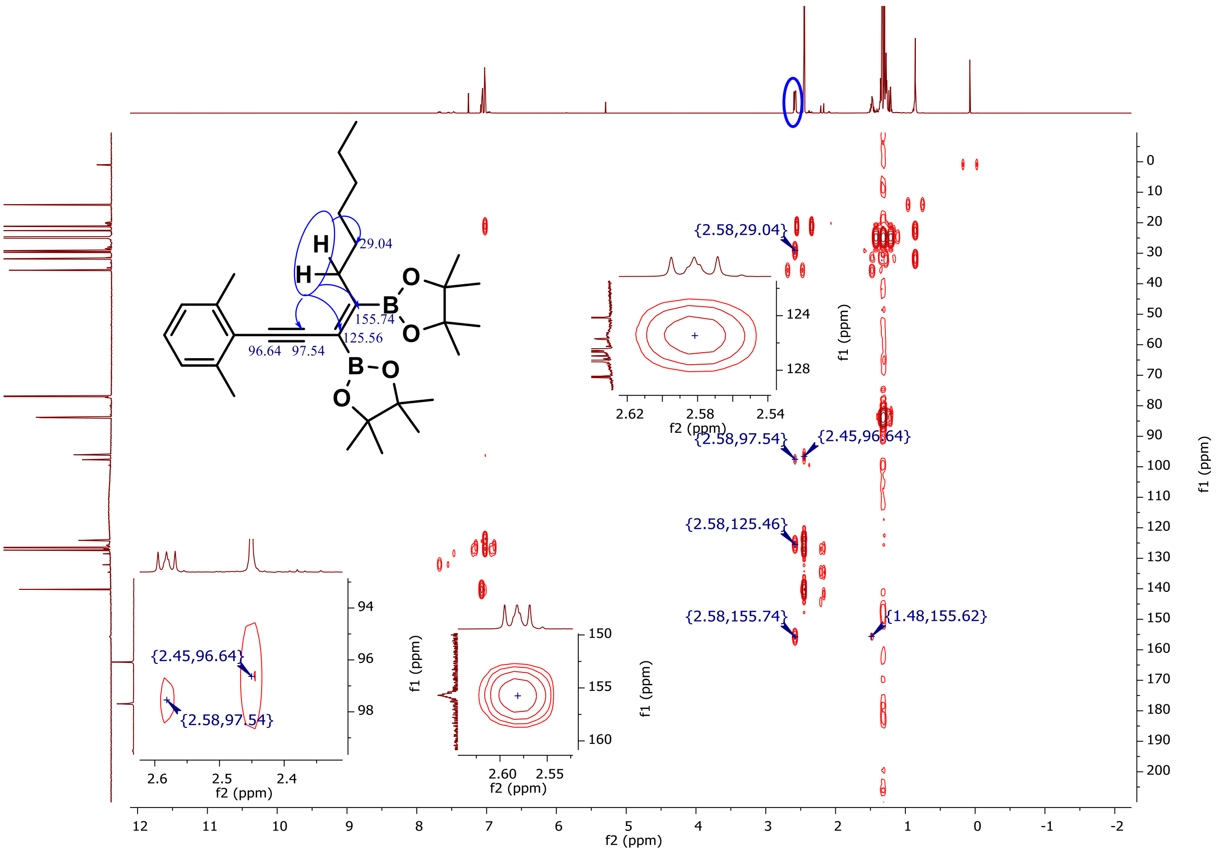

**Figure S144**. ¹H-¹³C HMBC NMR spectrum of **3z.**

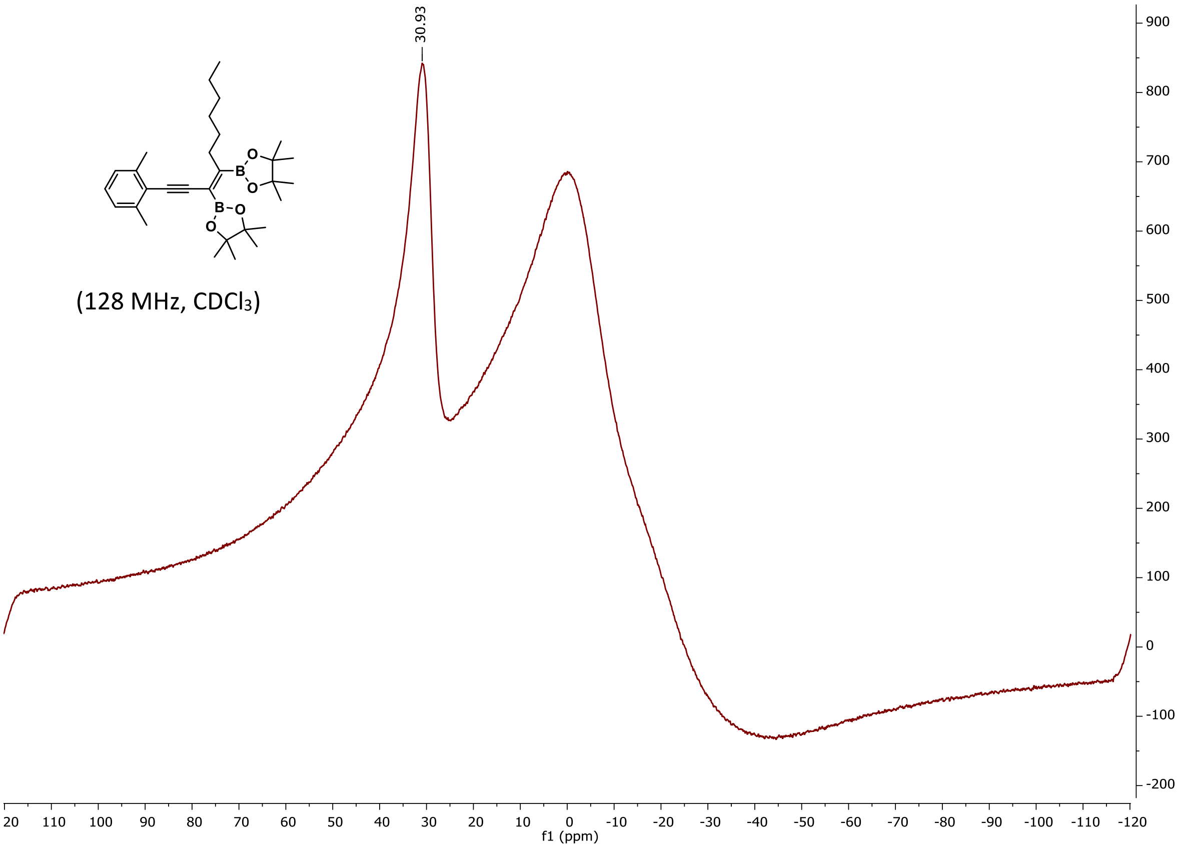


**Figure S145**. $^{11}$B NMR spectrum of **3z.**

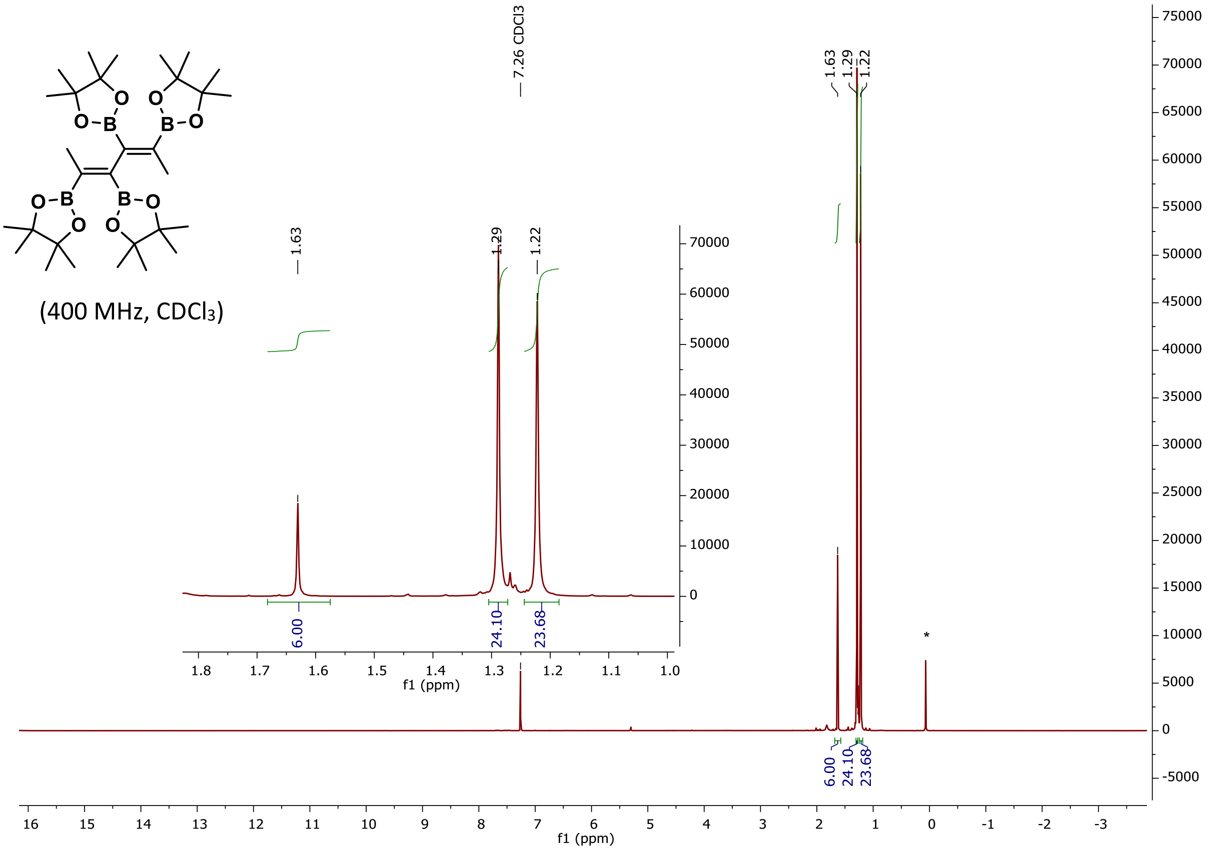


**Figure S146**. $^{1}$H NMR spectrum of **4d.** * - Grease

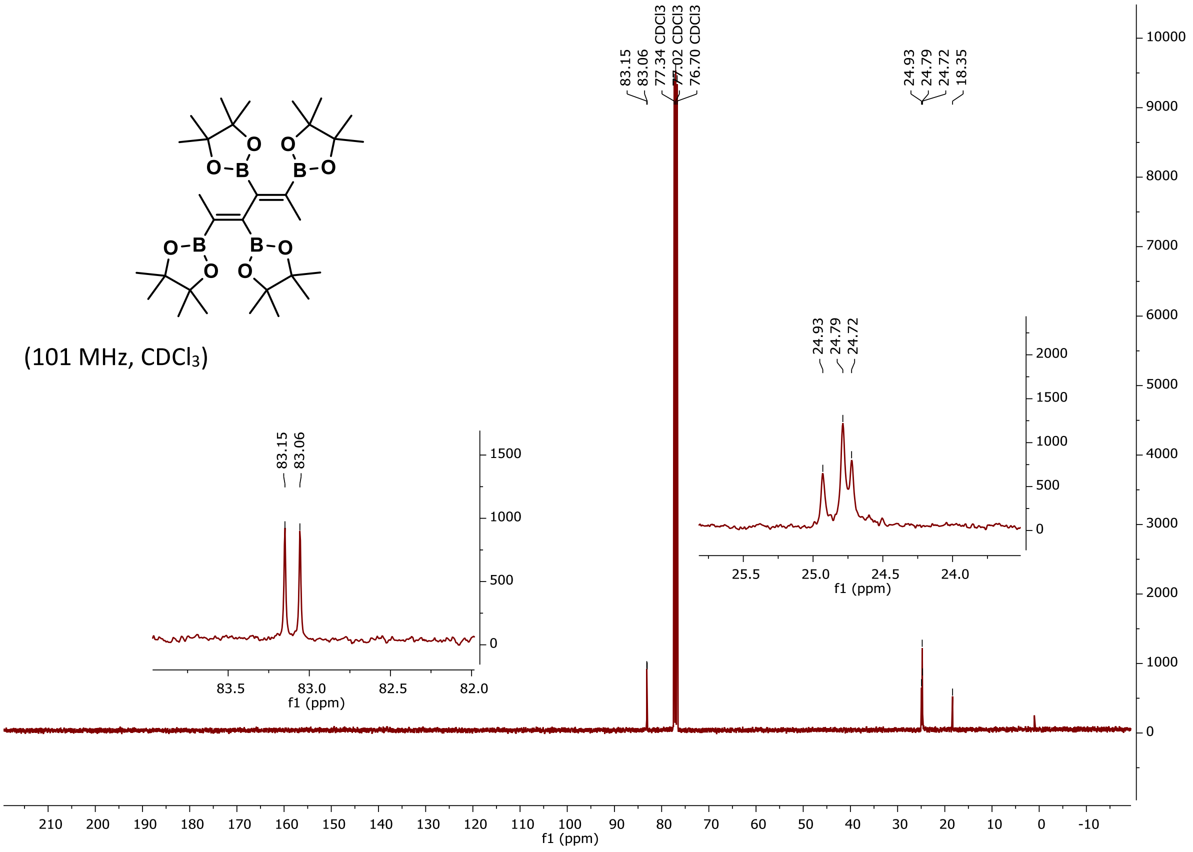


**Figure S147**. $^{13}$C NMR spectrum of **4d.**

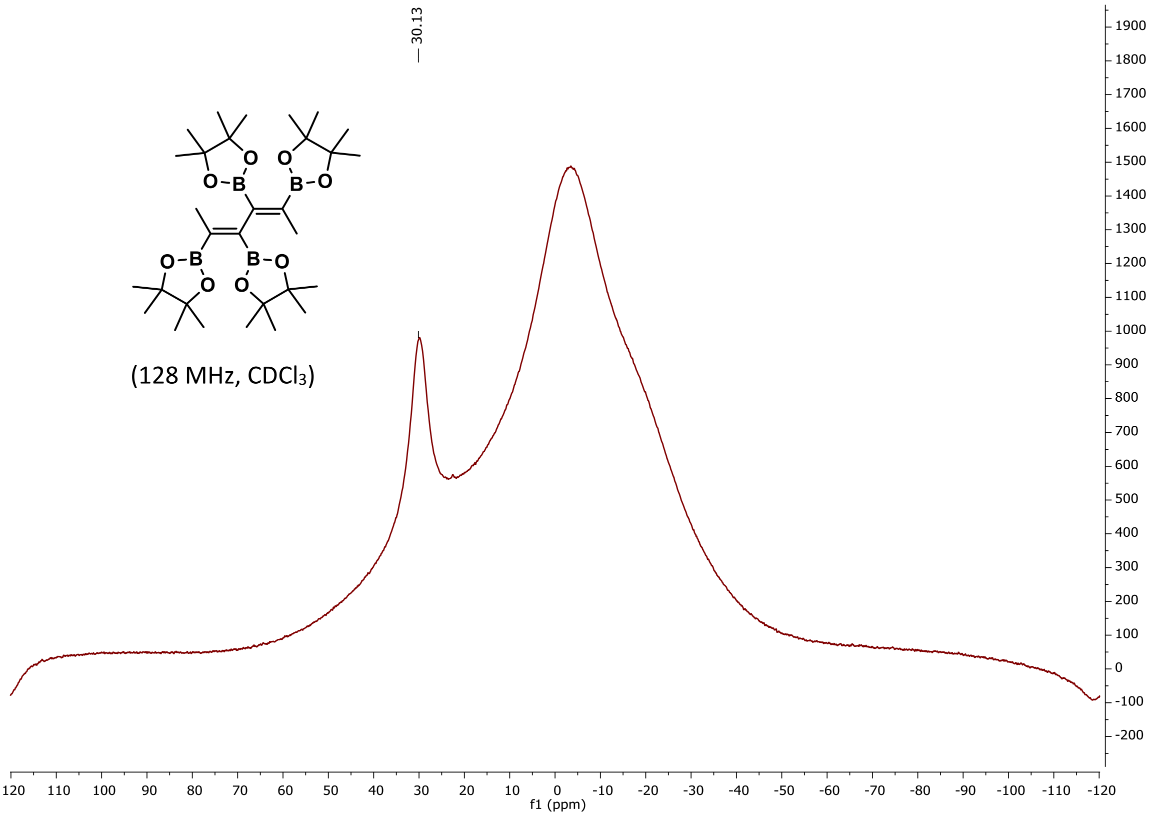


**Figure S148**. $^{11}$B NMR spectrum of **4d.**

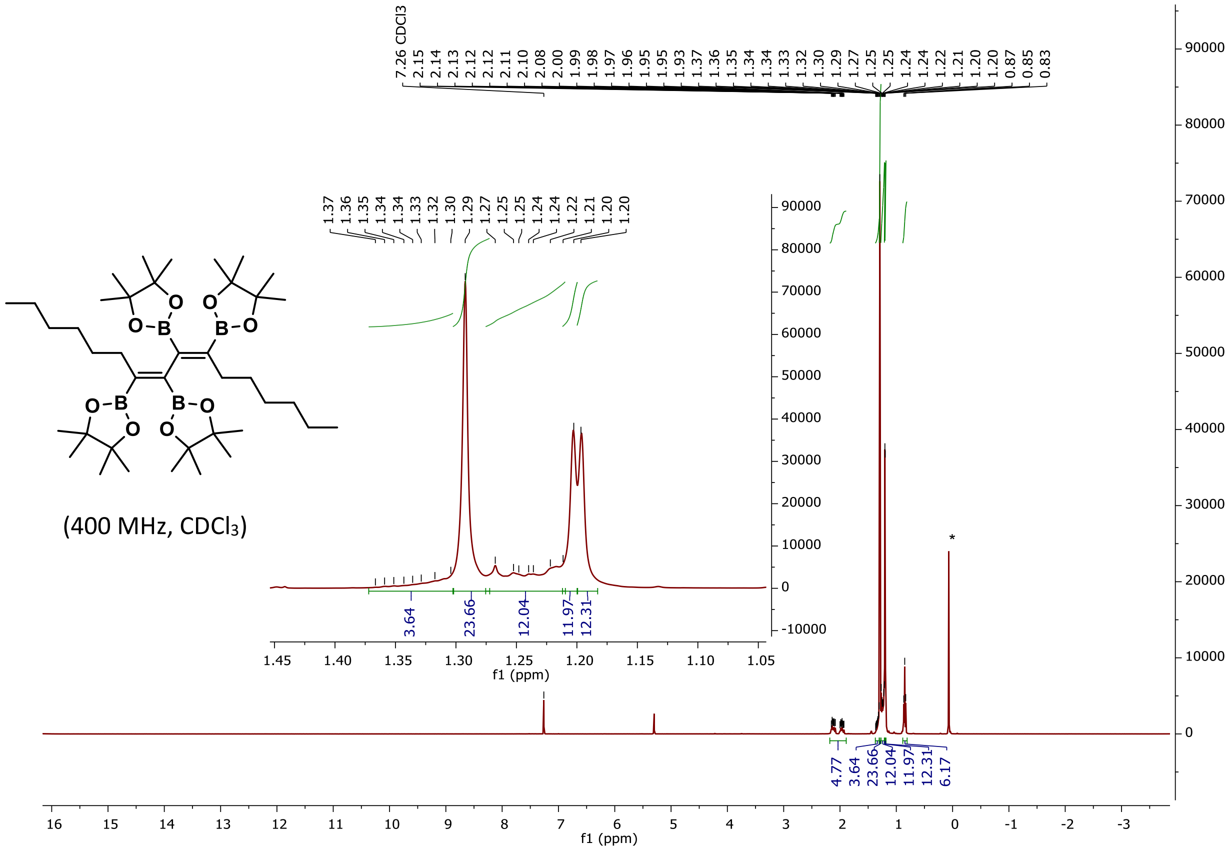


**Figure S149**. $^{1}$H NMR spectrum of **4e.** * - Grease

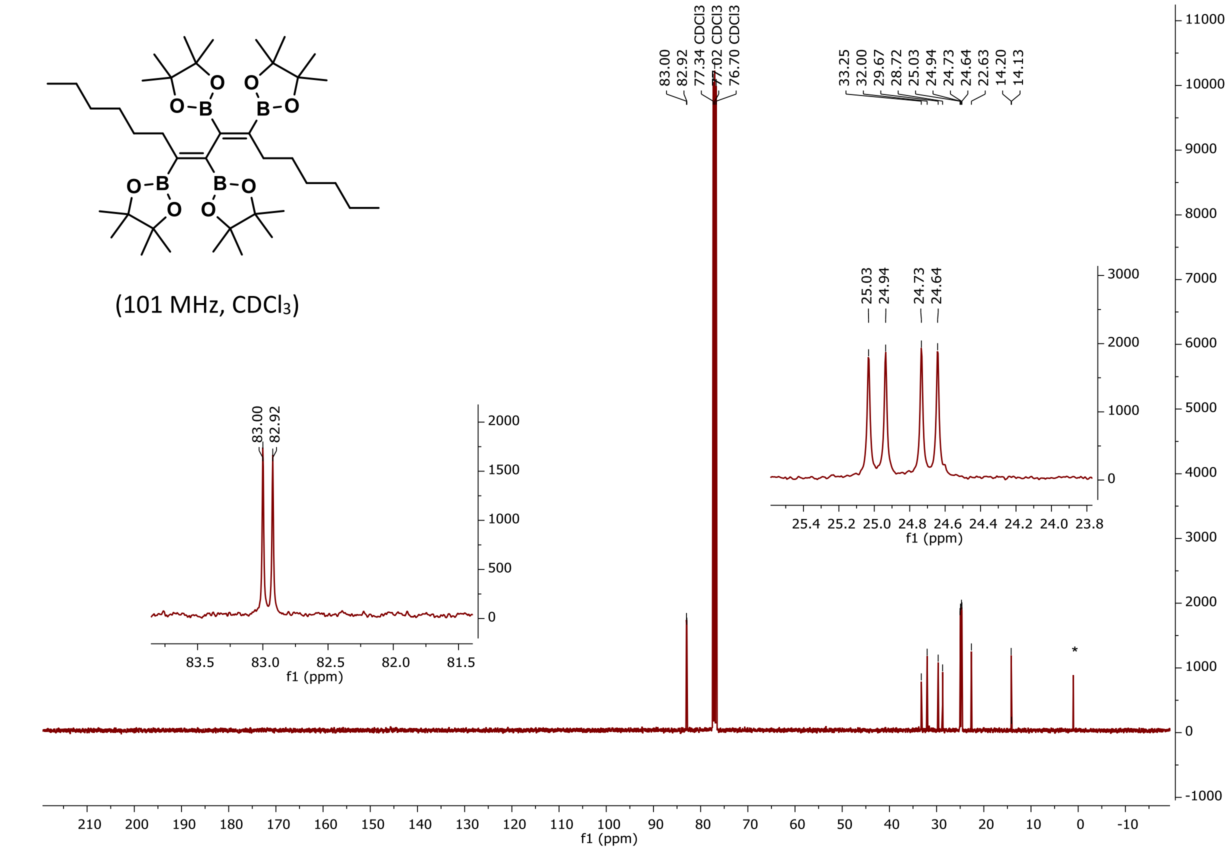


**Figure S150**. $^{13}$C NMR spectrum of **4d.** * - Grease

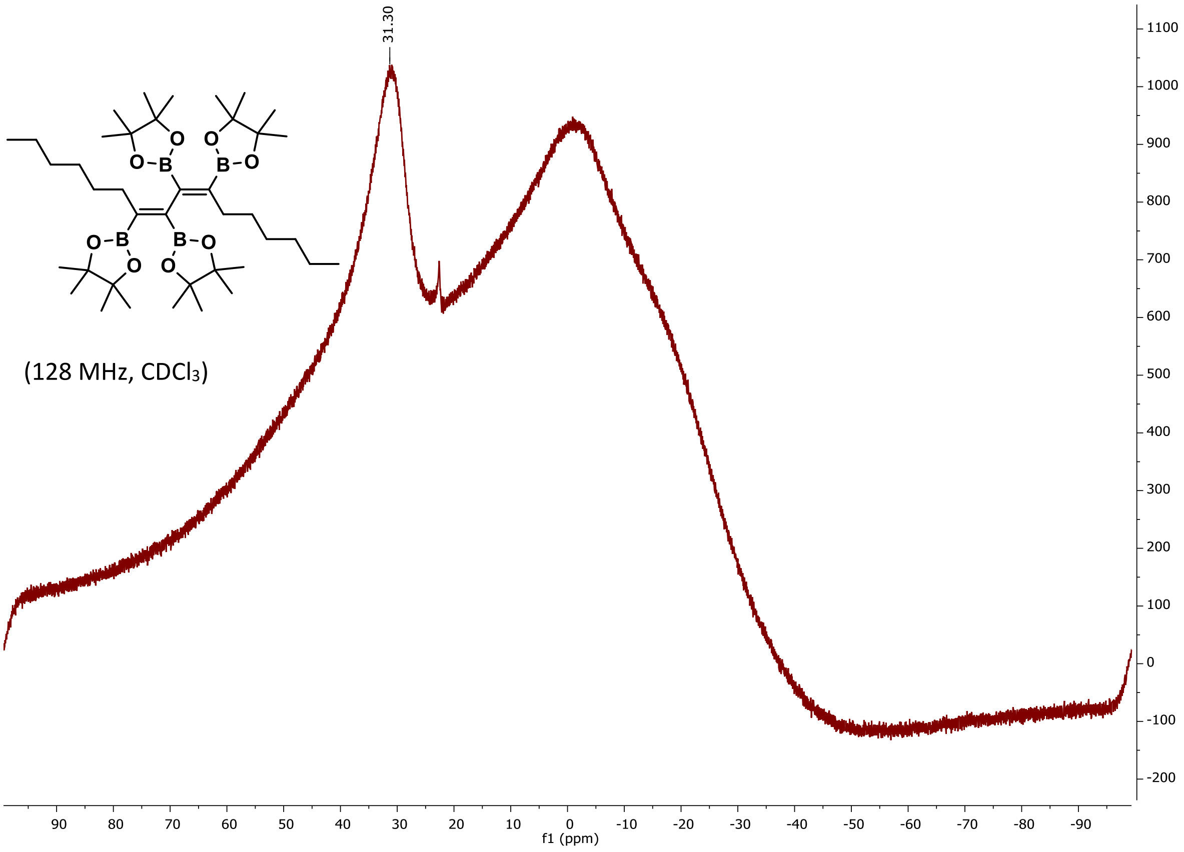

**Figure S151**. $^{11}$B NMR spectrum of **4d.**

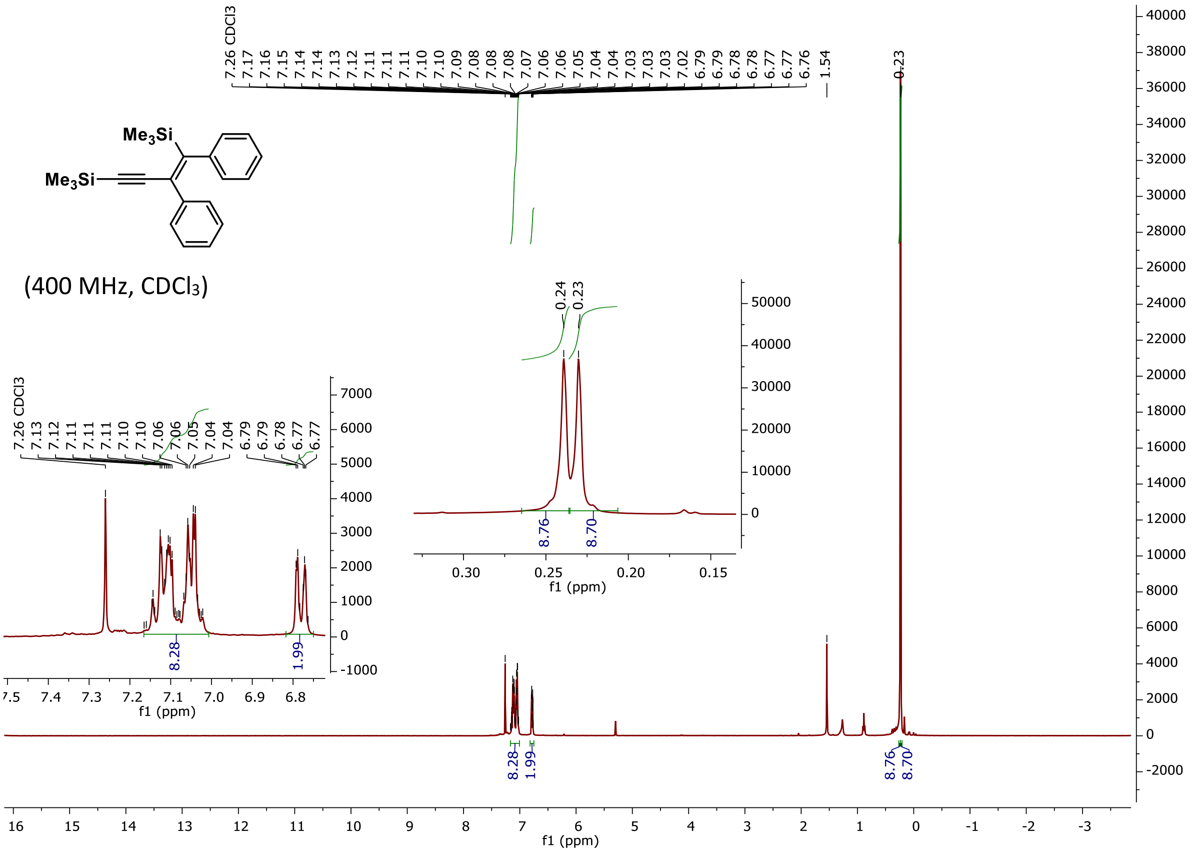

**Figure S152**. $^{1}$H NMR spectrum of **6.**

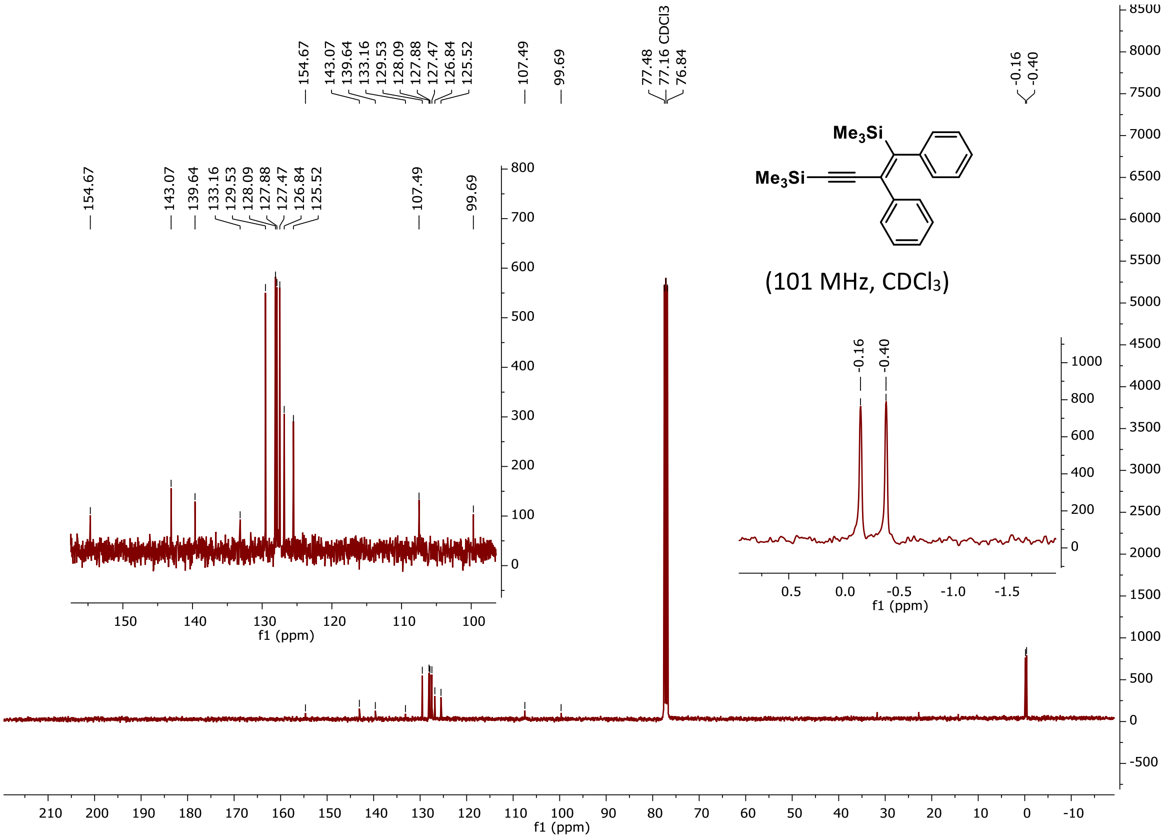


**Figure S153**. $^{13}C$ NMR spectrum of **6.**

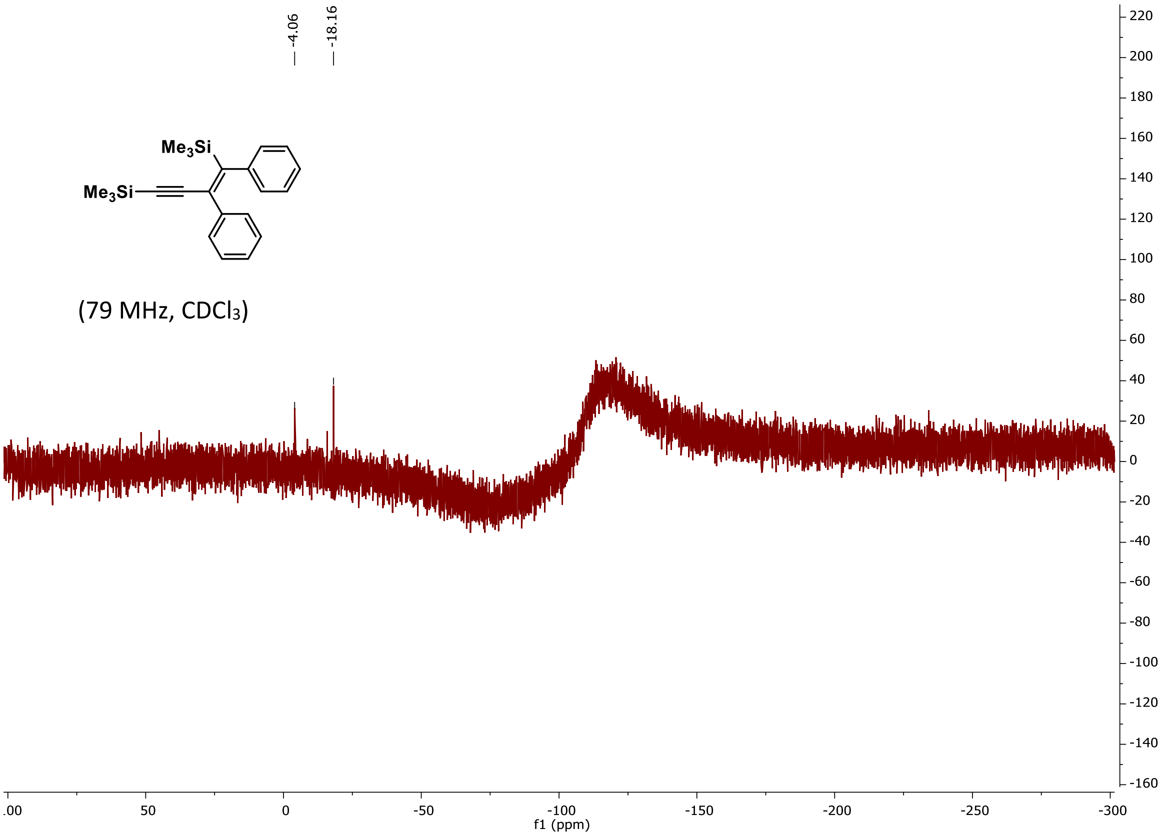


**Figure S154**. $^{29}Si$ NMR spectrum of **6.**

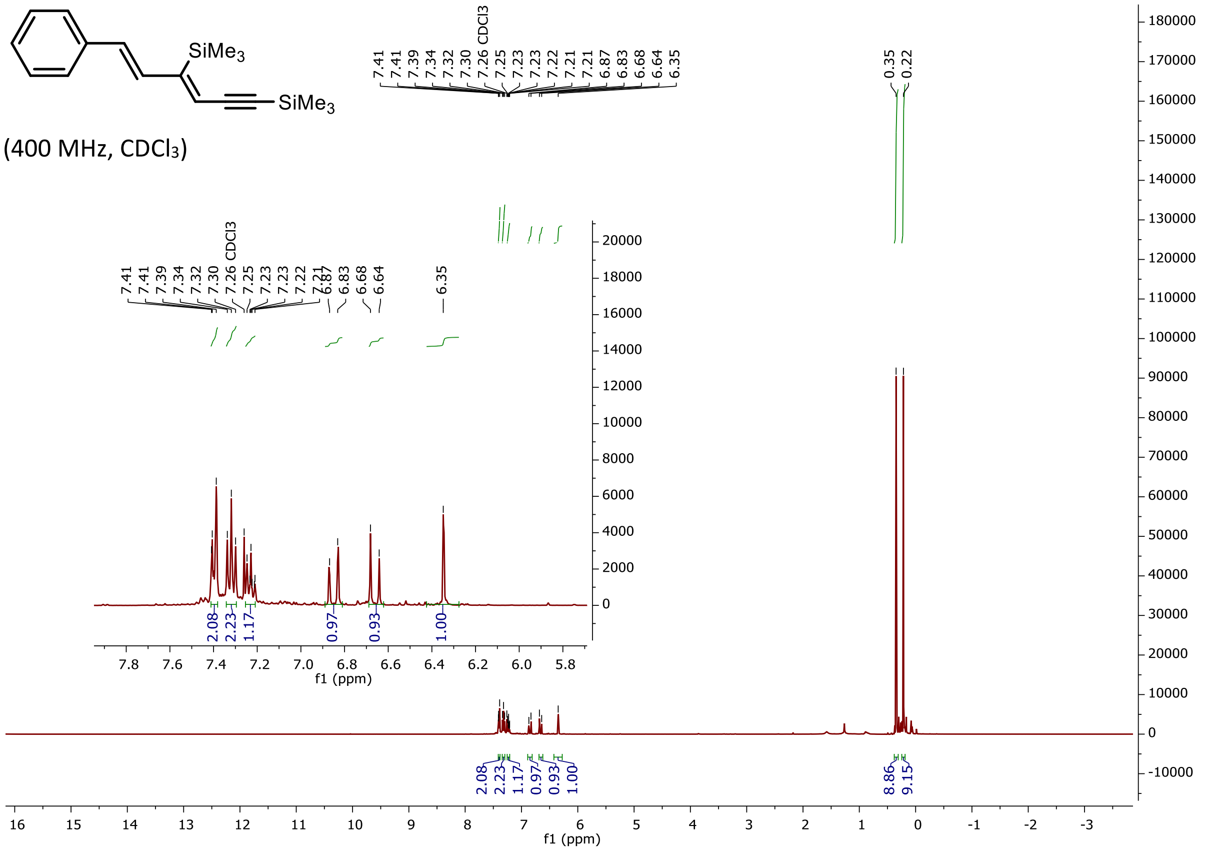


**Figure S155**. $^{1}$H NMR spectrum of **7a.**

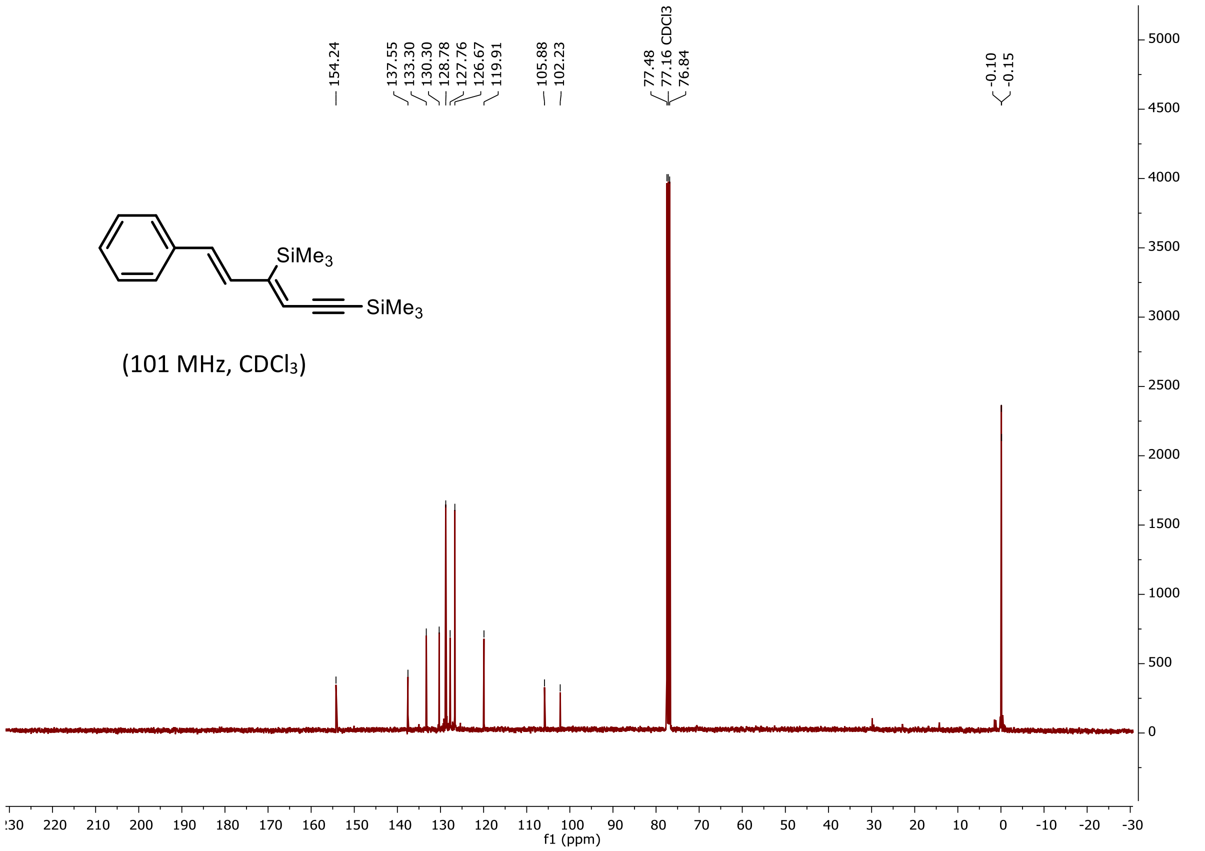


**Figure S156**. $^{13}$C NMR spectrum of **7a.**

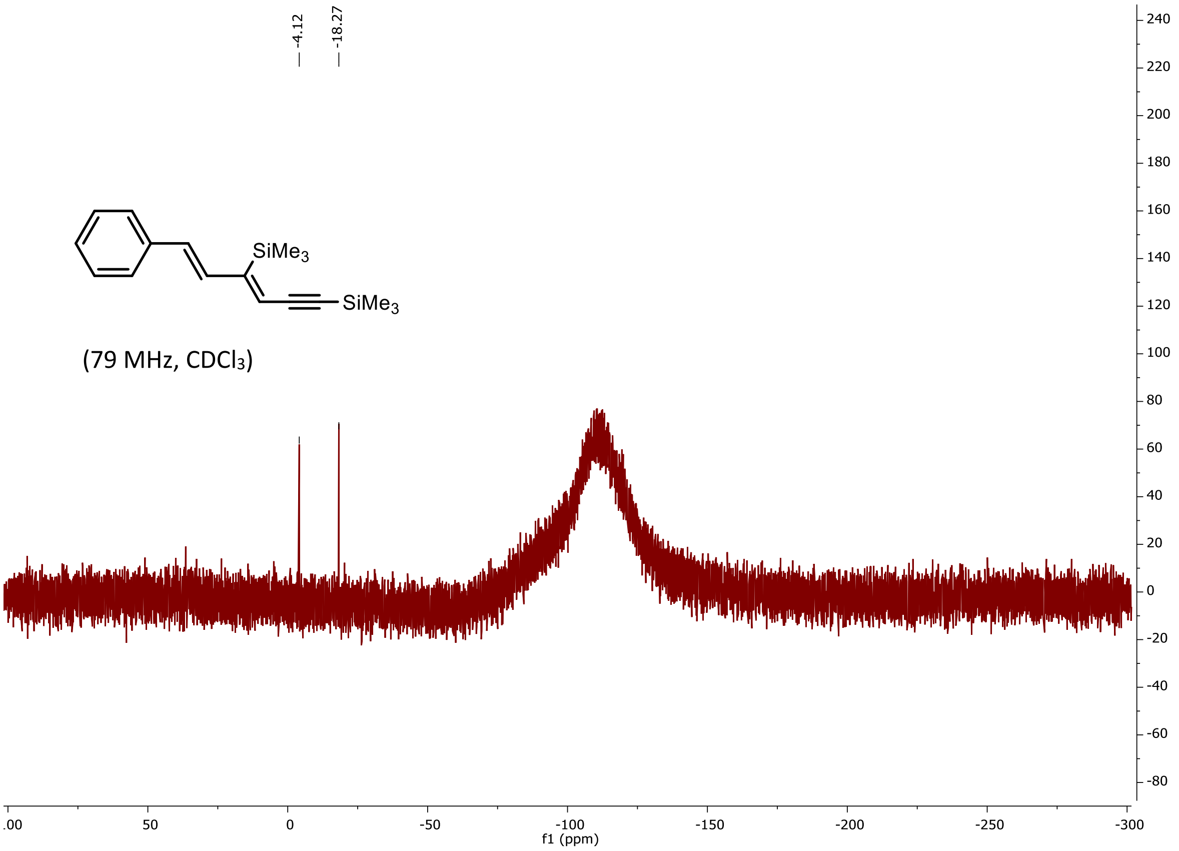


**Figure S157**. $^{29}$Si NMR spectrum of **7a.**

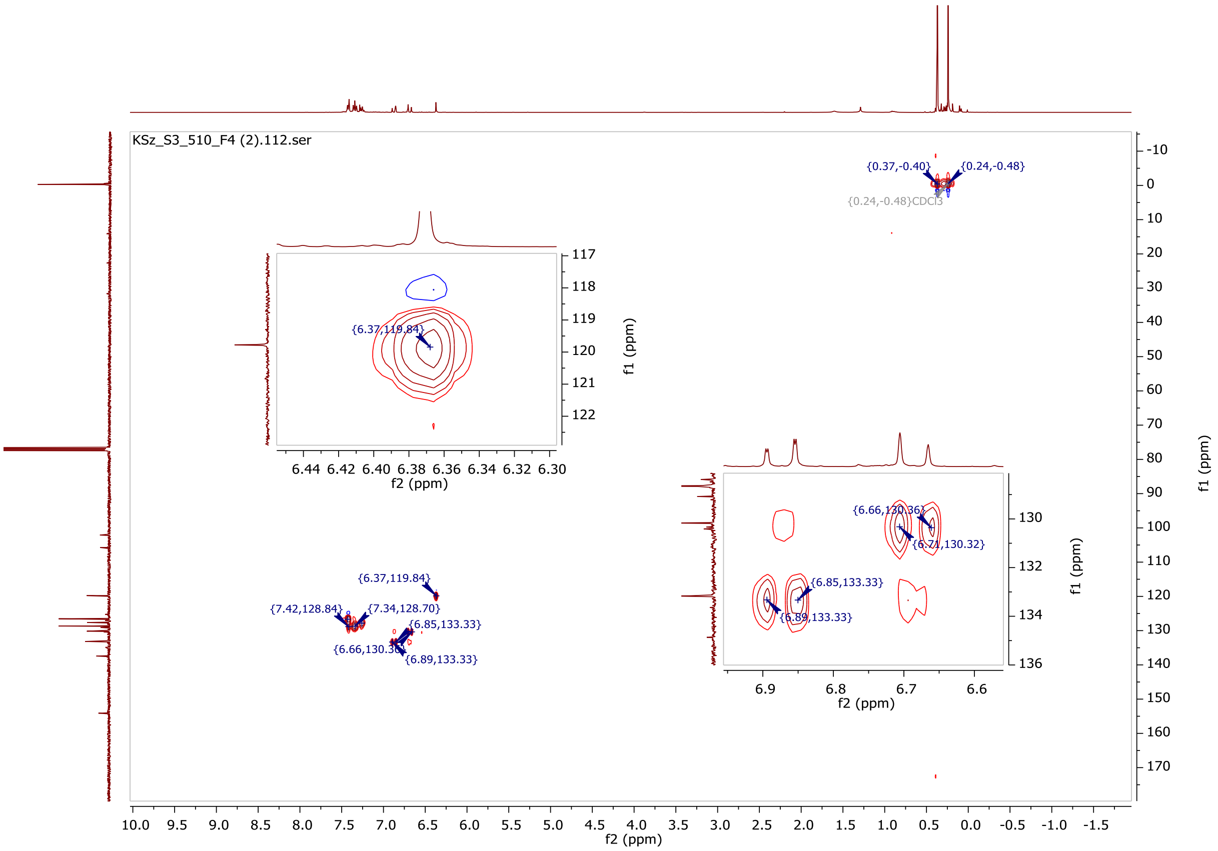


**Figure S158**. $^{1}$H-$^{13}$C HSQC NMR spectrum of **7a.**

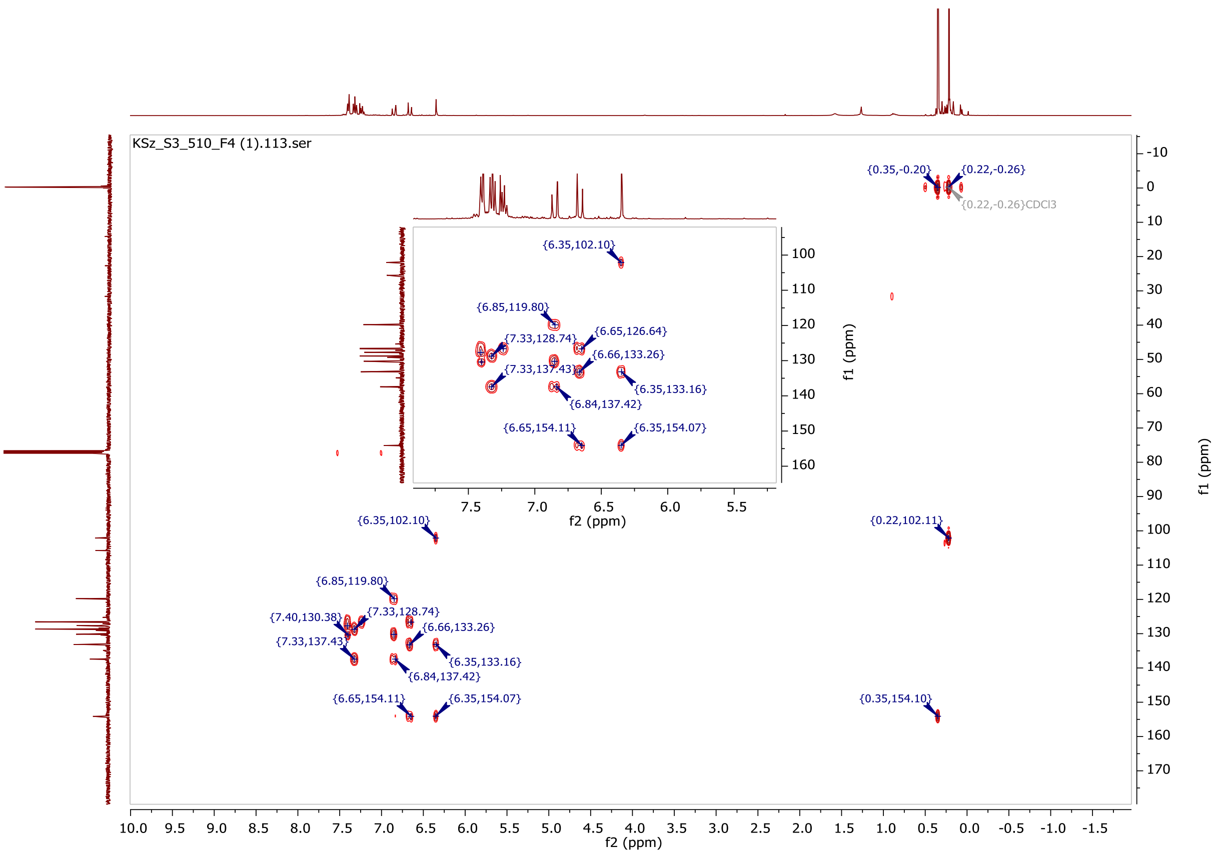


**Figure S159**. $^{1}H$-$^{13}C$ HMBC NMR spectrum of **7a.**

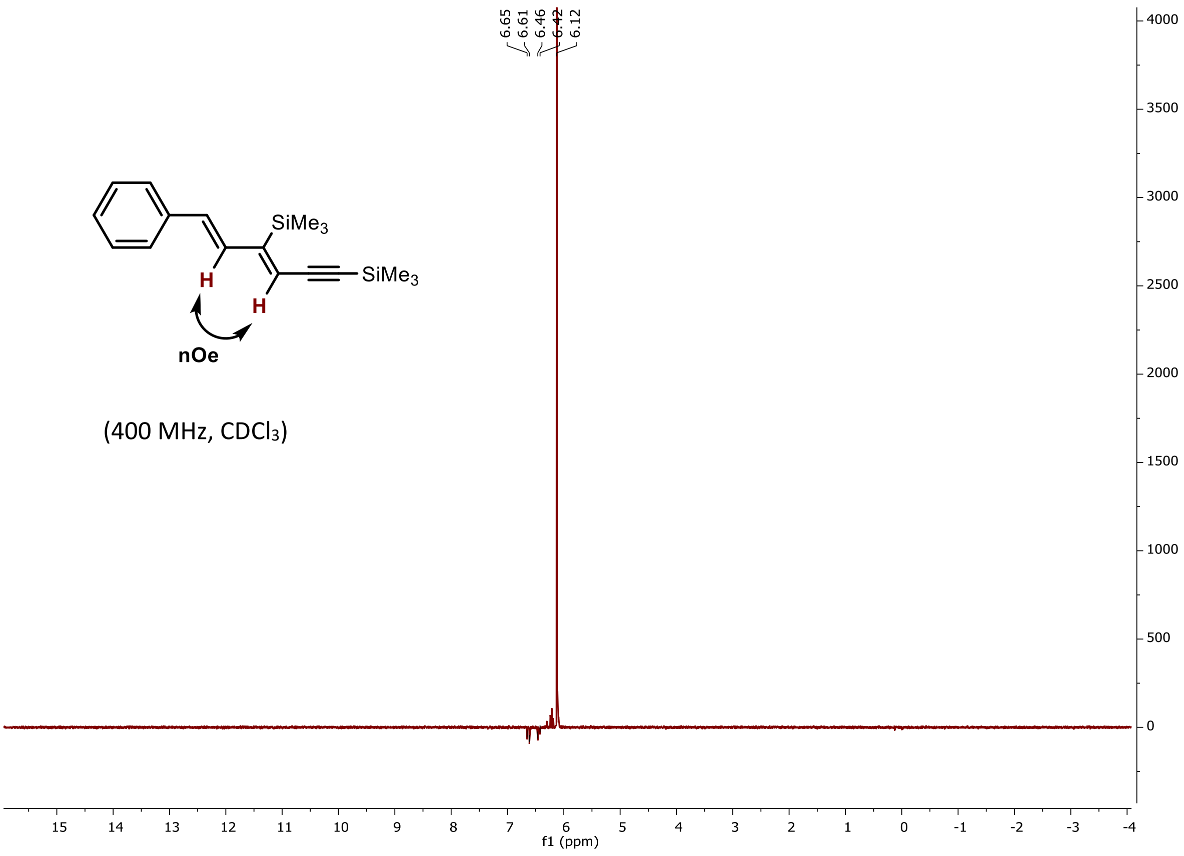


**Figure S160**. Selective 1D NOESY spectrum of **7a** directed at the singlet at 6.12 ppm.

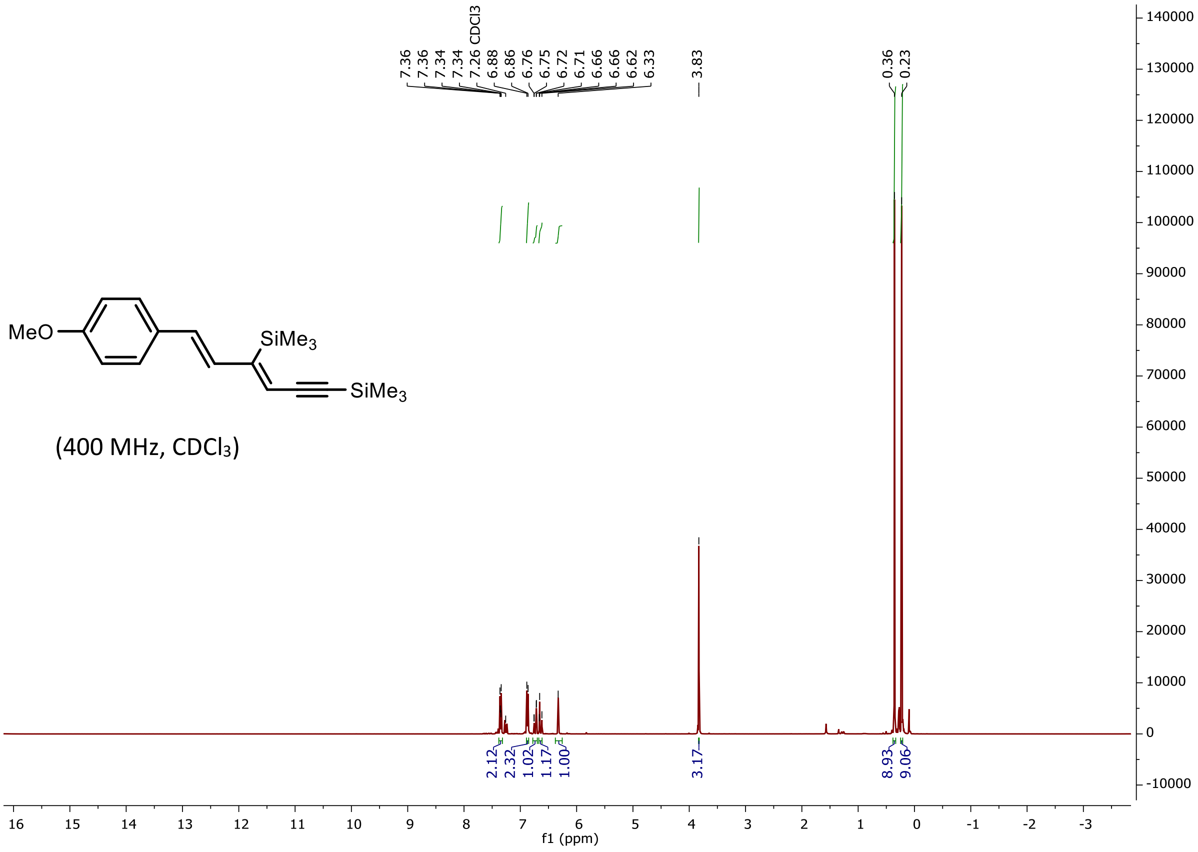


**Figure S161**. $^{1}$H NMR spectrum of **7b.**

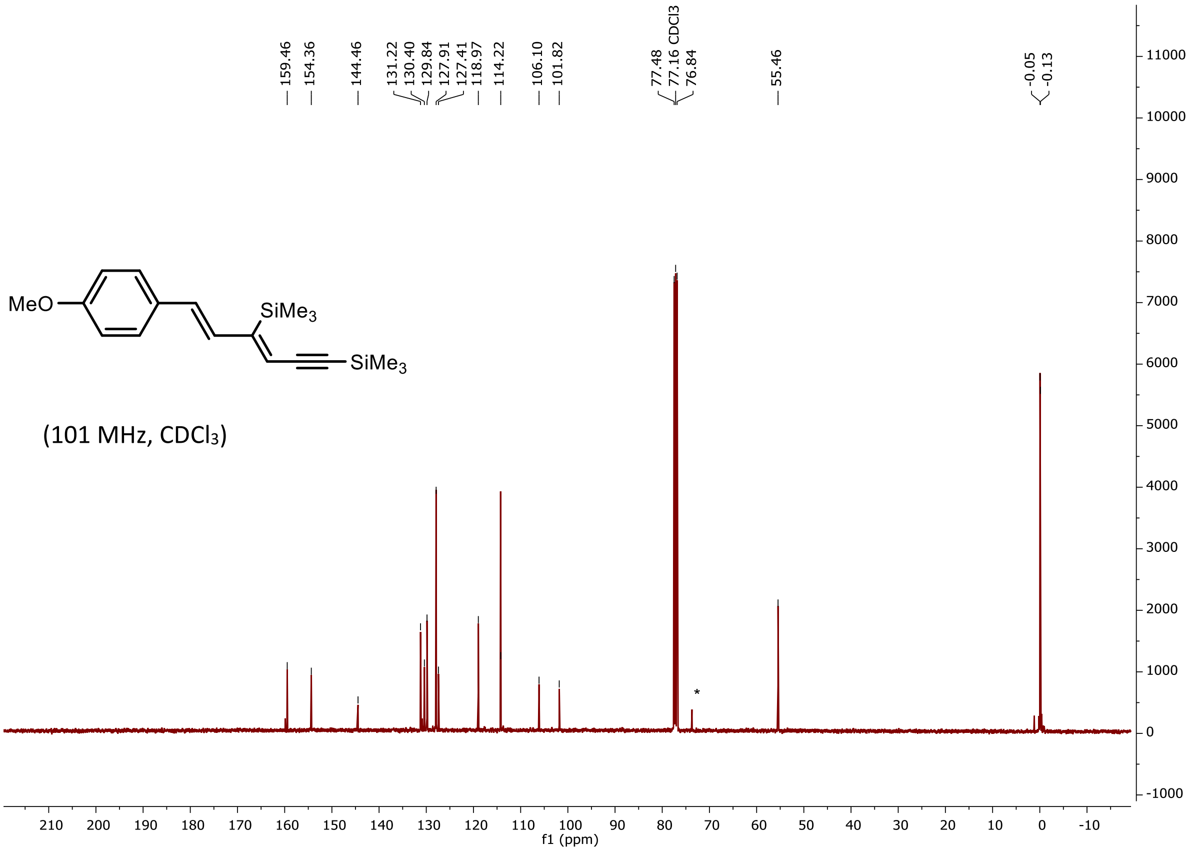


**Figure S162**. $^{13}$C NMR spectrum of **7b.** * - Unknown impurities.

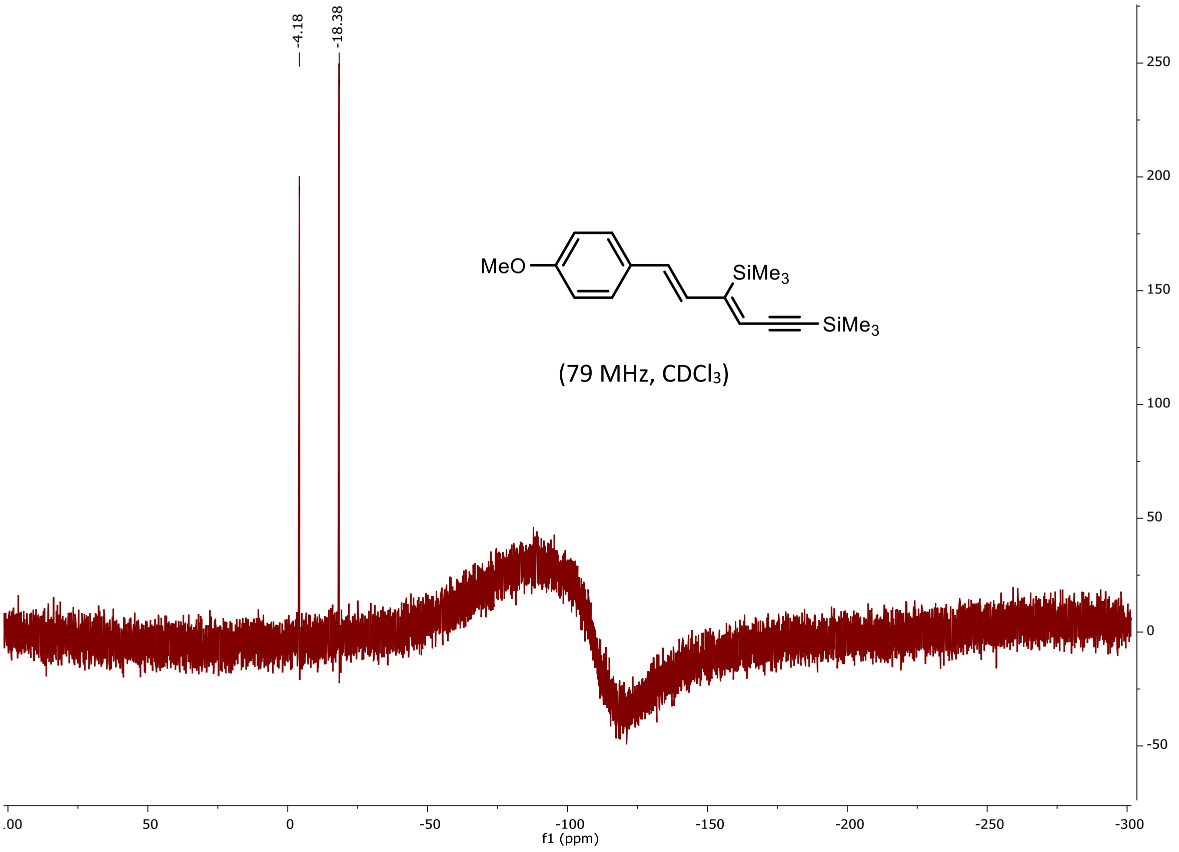


**Figure S163**. $^{29}$Si NMR spectrum of **7b.**

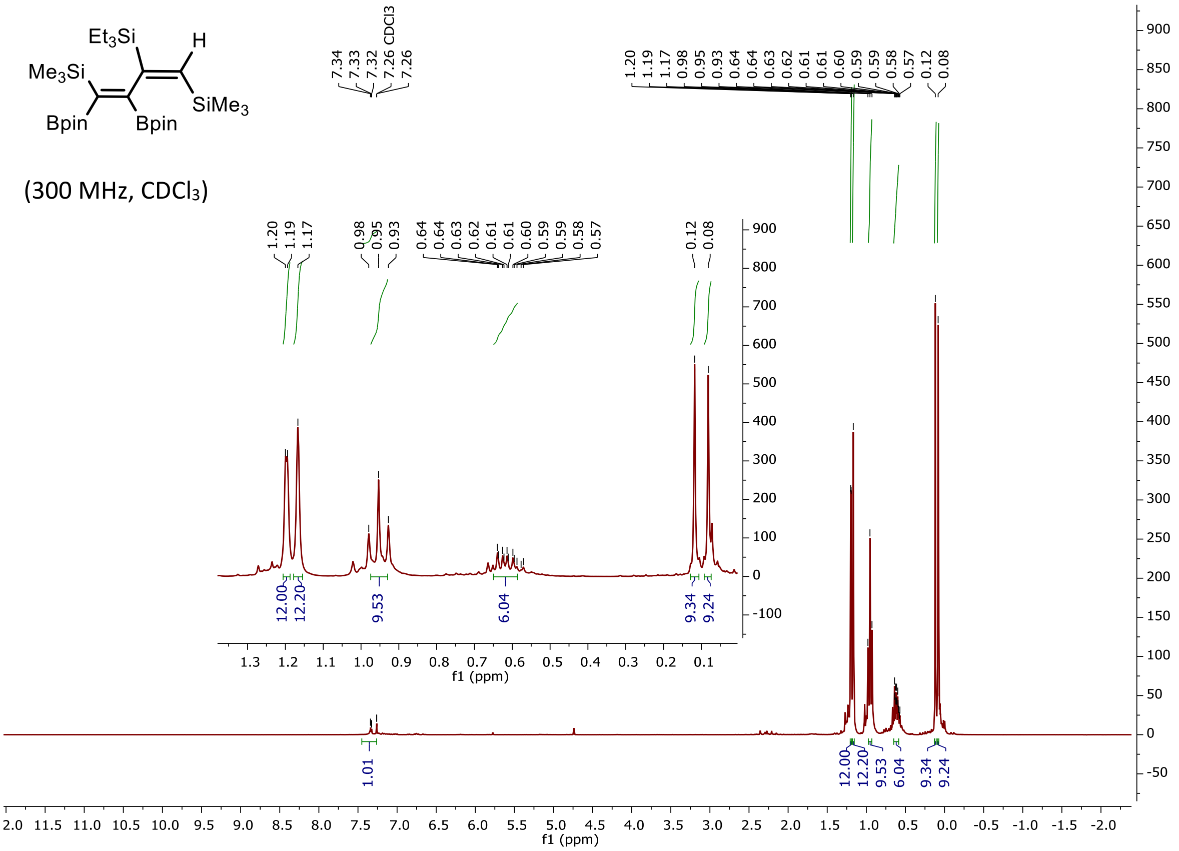


**Figure S164**. $^{1}$H NMR spectrum of **8.**

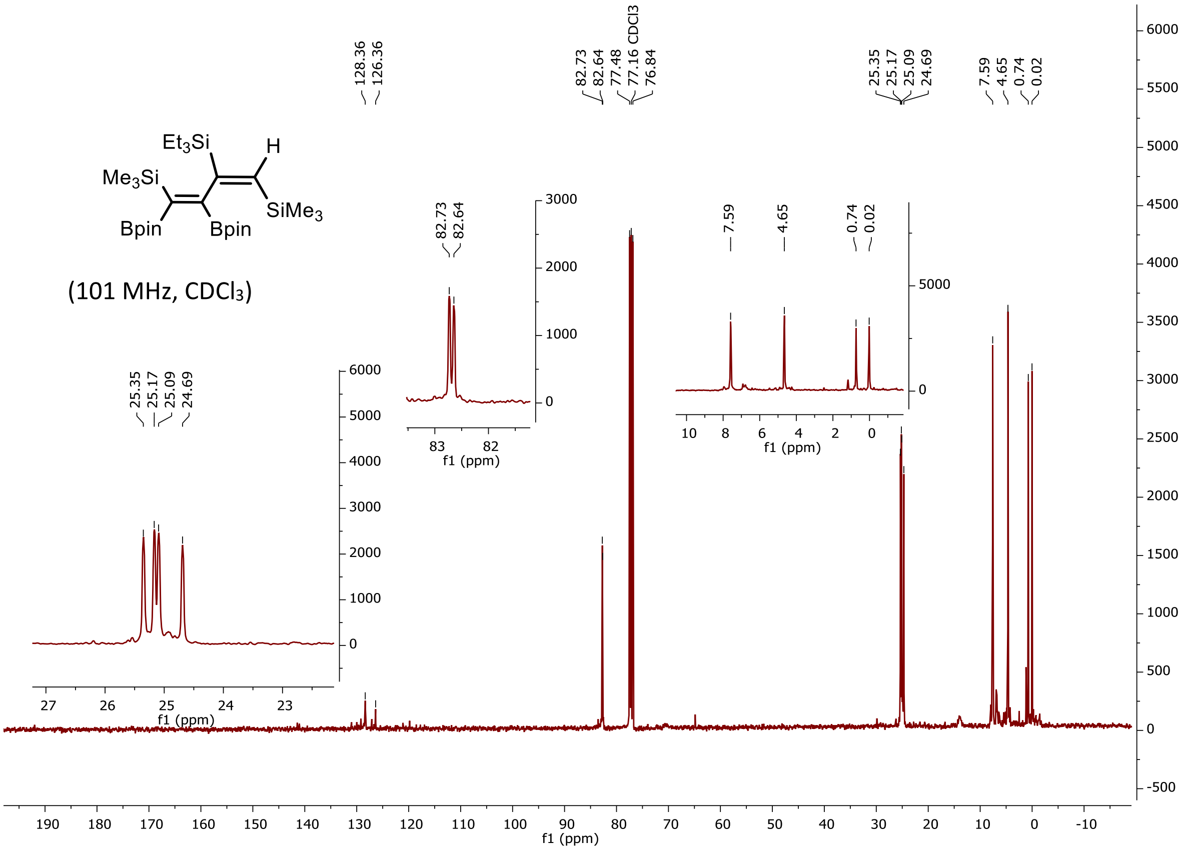


**Figure S165**. $^{13}$C NMR spectrum of **8.**

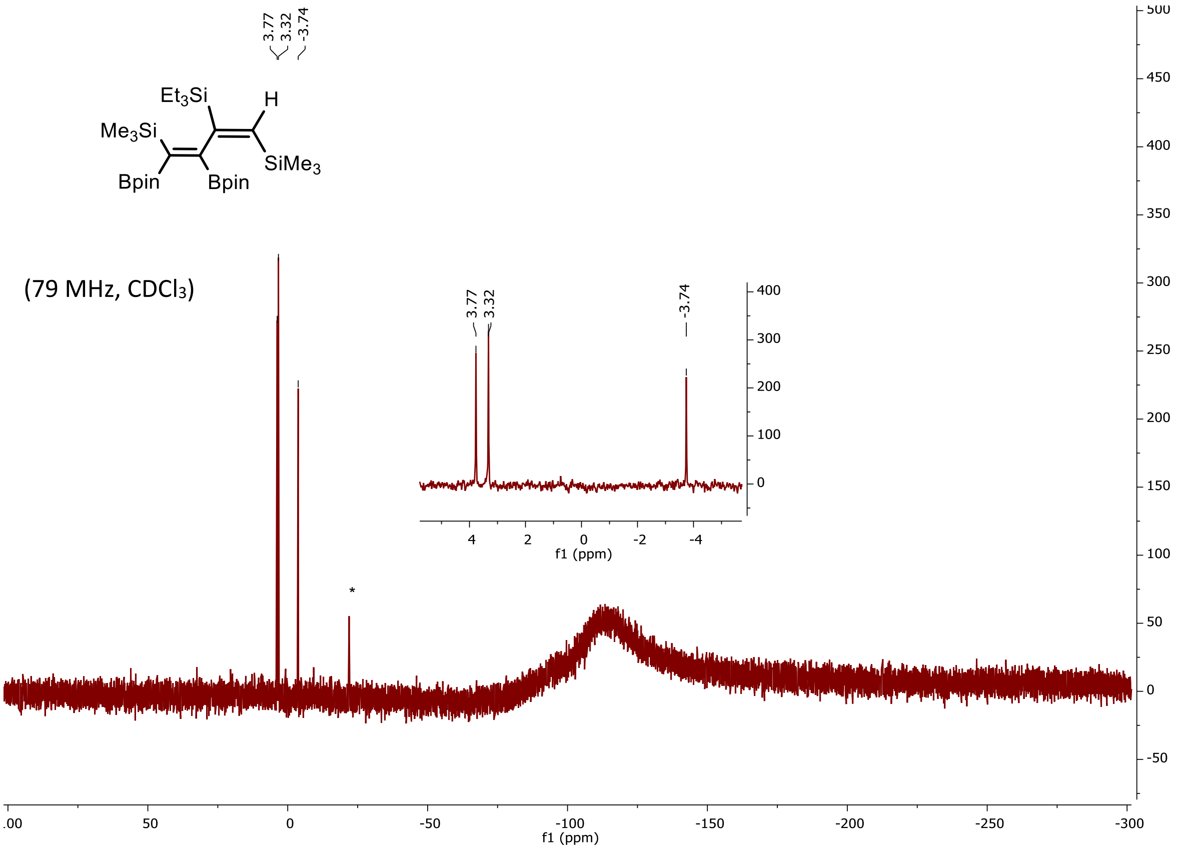


**Figure S166**. $^{29}$Si NMR spectrum of **8.** * - Grease

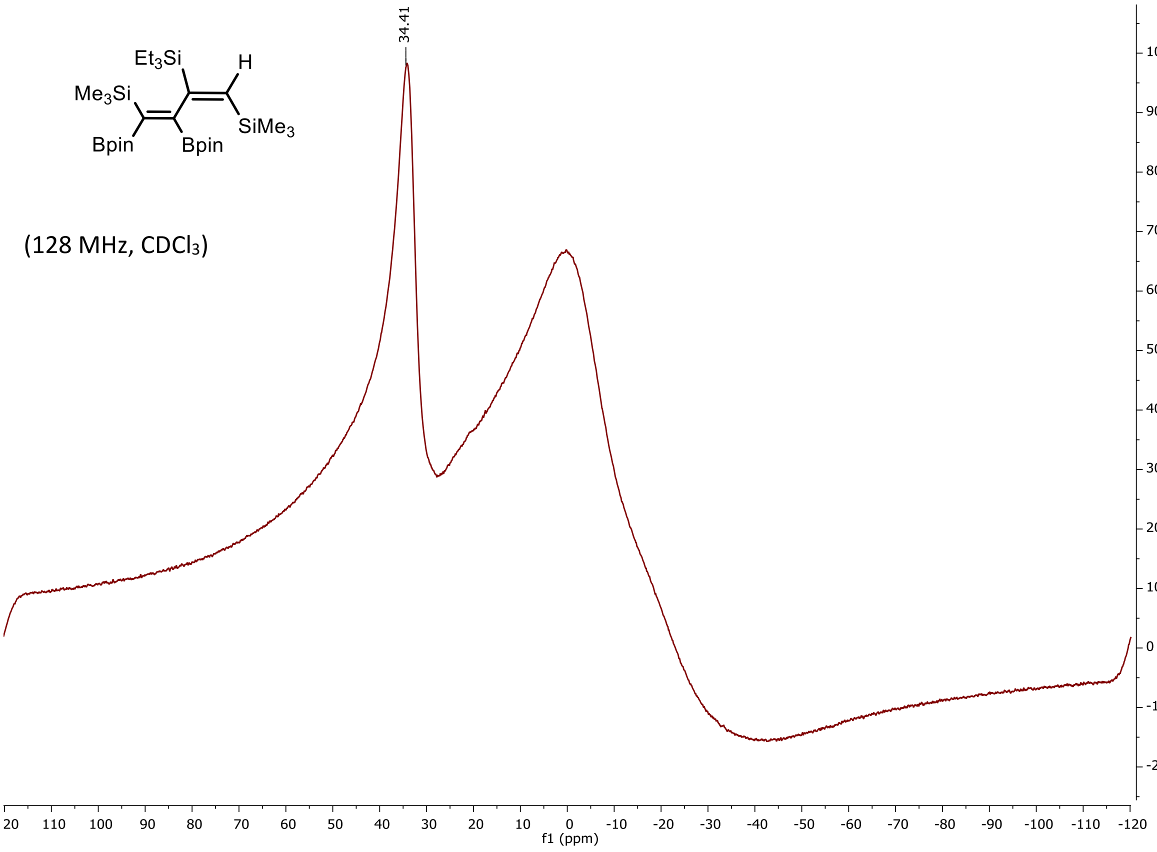


**Figure S167**. $^{11}$B NMR spectrum of **8.**

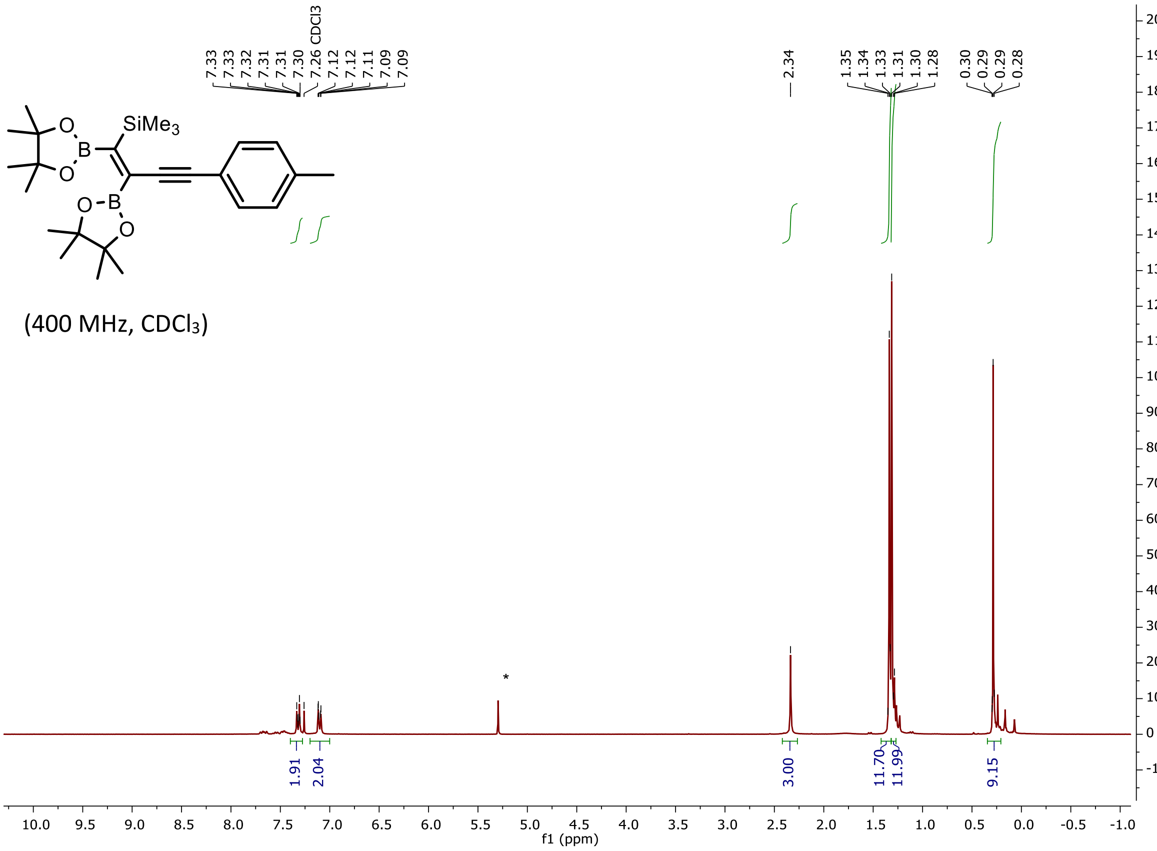


**Figure S168**. $^{1}$H NMR spectrum of **9.**

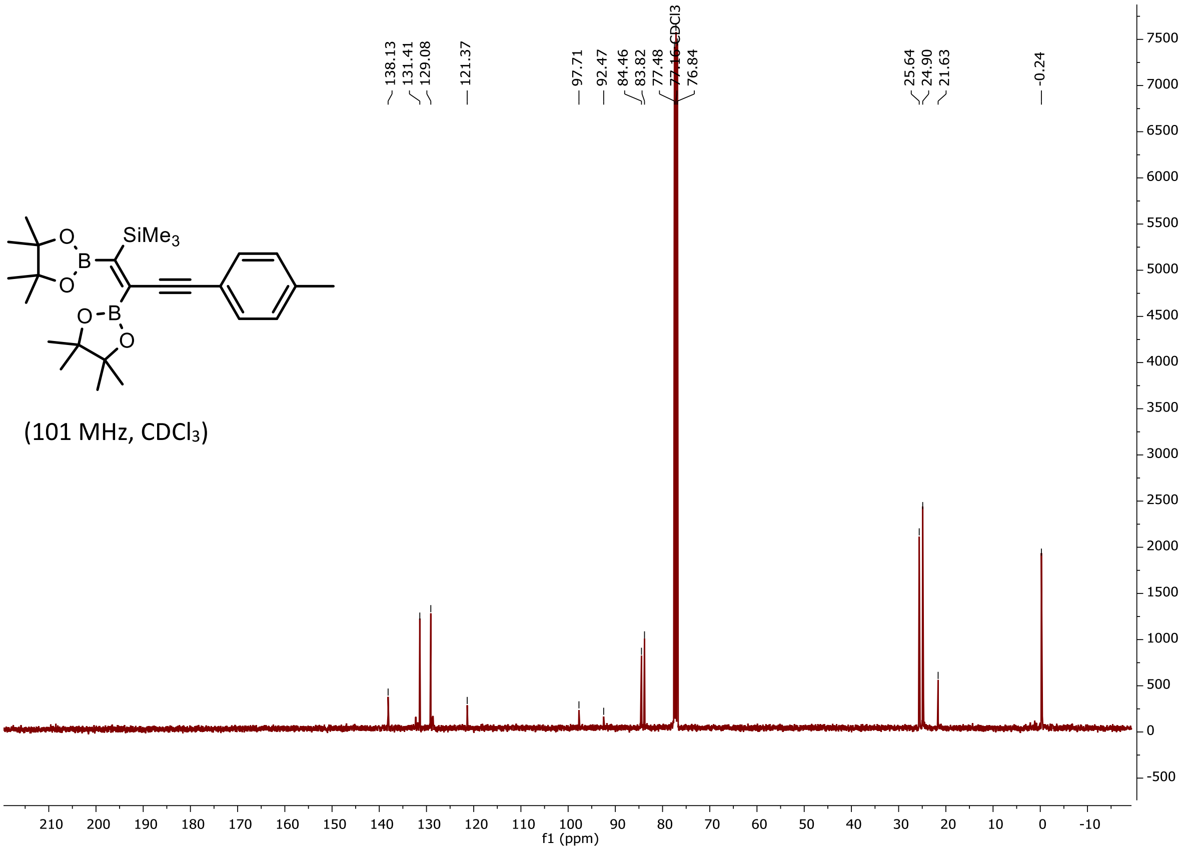


**Figure S169**. $^{13}C$ NMR spectrum of **9.**

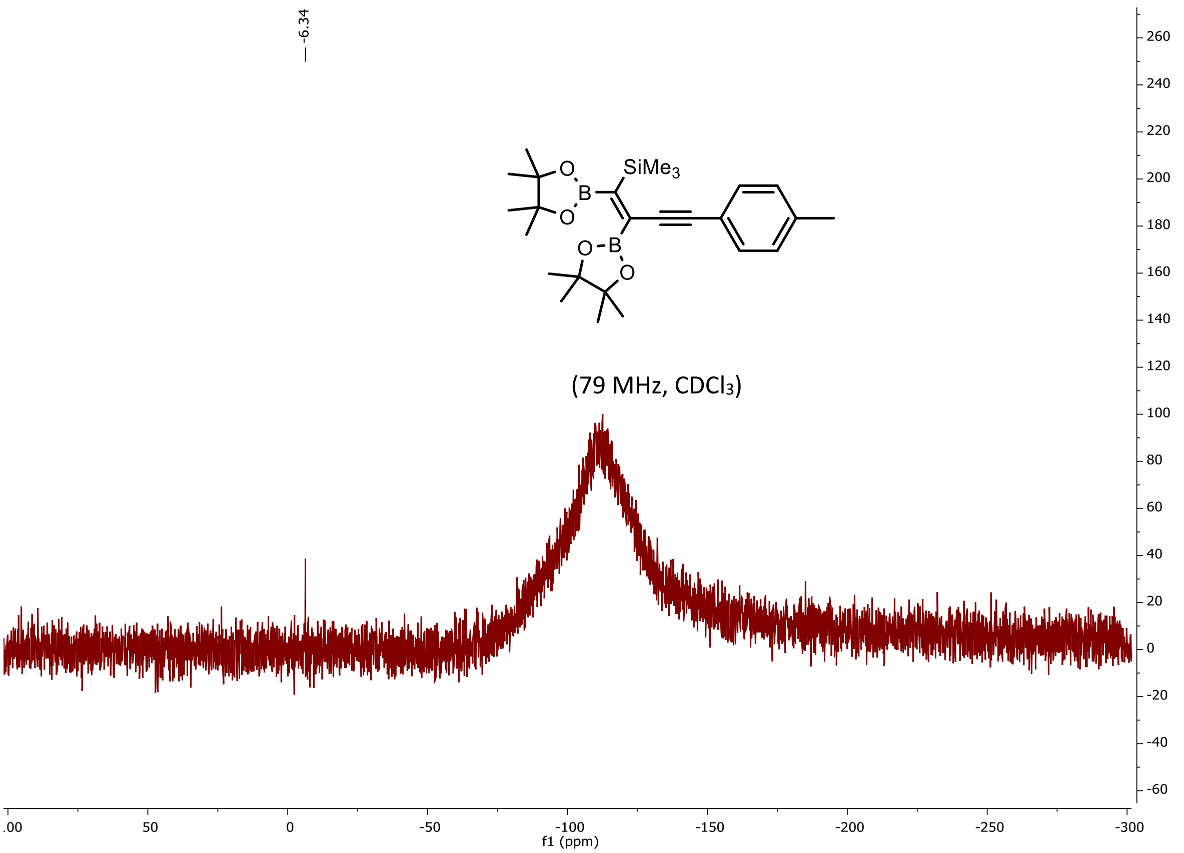


**Figure S170**. $^{29}Si$ NMR spectrum of **9.**

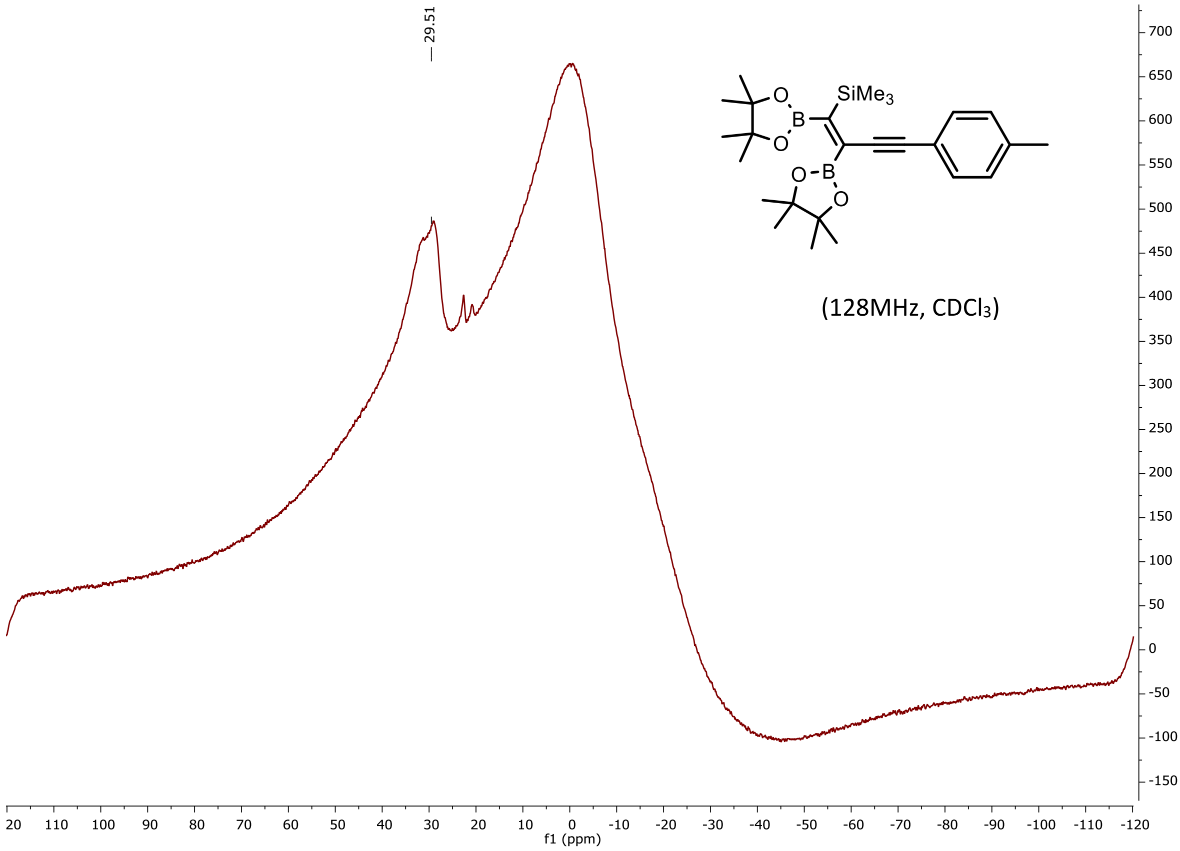


**Figure S171**. ^11B NMR spectrum of **9.**

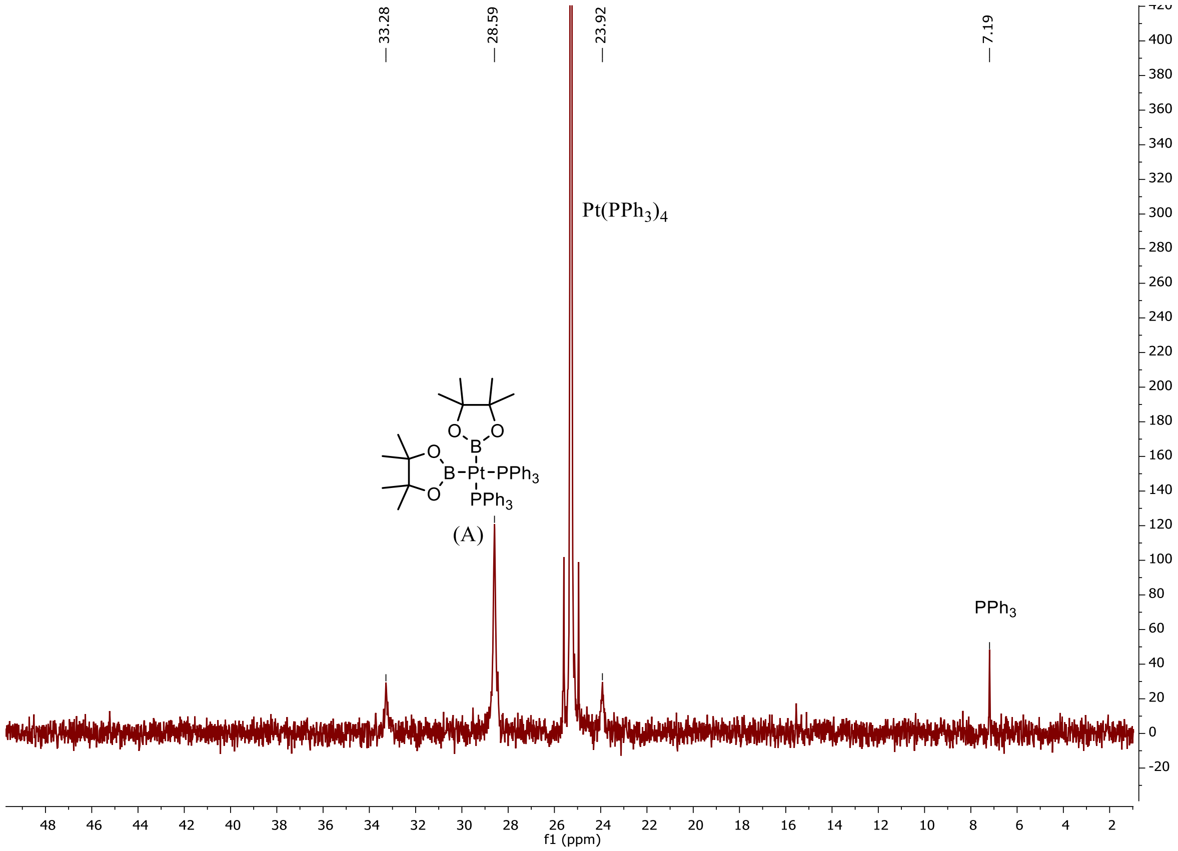


**Figure S172**. Formation of a new (**A**) specie via addition of $B_2pin_2$ to $[Pt(PPh_3)_4]$.

## 6. References


1. Il'inich, G. N.; Zudin, V. N.; Nosov, A. V.; Rogov, V. A.; Likholobov, V. A., The mechanism of the catalytic behaviour of platinum triphenylphosphine complexes in the ethylene hydrocarbonylation. *Journal of Molecular Catalysis A: Chemical* **1995,** *101* (3), 221-235.
2. Yamagishi, M.; Nishigai, K.; Hata, T.; Urabe, H., Nucleophilic addition of sulfonamides to bromoacetylenes: facile preparation of pyrroles. *Organic letters* **2011,** *13* (18), 4873-4875.
3. Lutter, F. H.; Grokenberger, L.; Spieß, P.; Hammann, J. M.; Karaghiosoff, K.; Knochel, P., Cobalt-Catalyzed Cross-Coupling of Functionalized Alkylzinc Reagents with (Hetero)Aryl Halides. *Angewandte Chemie International Edition* **2020,** *59* (14), 5546-5550.
4. Arisawa, M.; Tagami, Y.; Yamaguchi, M., Two types of rhodium-catalyzed CS/CS metathesis reactions: formation of CS/CS bonds and CC/SS bonds. *Tetrahedron Letters* **2008,** *49* (10), 1593-1597.
5. Adam, R.; Mon, M.; Greco, R.; Kalinke, L. H. G.; Vidal-Moya, A.; Fernandez, A.; Winpenny, R. E. P.; Doménech-Carbó, A.; Leyva-Pérez, A.; Armentano, D.; Pardo, E.; Ferrando-Soria, J., Self-Assembly of Catalytically Active Supramolecular Coordination Compounds within Metal–Organic Frameworks. *Journal of the American Chemical Society* **2019,** *141* (26), 10350-10360.
6. Chen, X.; Zhang, H.; Chen, J.; Gong, H., A Mild CuCl-catalyzed Glaser-type Homocoupling Reaction: Air Oxidation and Room Temperature. *Chemistry Letters* **2015,** *44* (2), 129-131.
7. Adimurthy, S.; Malakar, C. C.; Beifuss, U., Influence of Bases and Ligands on the Outcome of the Cu(I)-Catalyzed Oxidative Homocoupling of Terminal Alkynes to 1,4-Disubstituted 1,3-Diynes Using Oxygen as an Oxidant. *The Journal of Organic Chemistry* **2009,** *74* (15), 5648-5651.
8. Pérez, J. M.; Cano, R.; Yus, M.; Ramón, D. J., Copper-Impregnated Magnetite as a Heterogeneous Catalyst for the Homocoupling of Terminal Alkynes. *Synthesis* **2013,** *45* (10), 1373-1379.
9. Schwarz, H.; Köppel, C.; Bohlmann, F., Elektronenstossinduzierte fragmentierung von acetylenverbindungen—XII: Umlagerungen von bis-(trimethylsilyl)-äthern ungesättigter α,ω-diole und massenspektrometrische identifizierung isomerer phenole. *Tetrahedron* **1974,** *30* (5), 689-693.
10. Schörgenhumer, J.; Waser, M., Transition metal-free coupling of terminal alkynes and hypervalent iodine-based alkyne-transfer reagents to access unsymmetrical 1,3-diynes. *Organic & Biomolecular Chemistry* **2018,** *16* (41), 7561-7563.
11. Stefanowska, K.; Szyling, J.; Walkowiak, J. d.; Franczyk, A., Alkenyl-Functionalized Open-Cage Silsesquioxanes (RSiMe2O) 3R′ 7Si7O9: A Novel Class of Building Nanoblocks. *Inorganic Chemistry* **2021**.
12. Banerjee, S.; Patil, N. T., Exploiting the dual role of ethynylbenziodoxolones in gold-catalyzed C(sp)–C(sp) cross-coupling reactions. *Chemical Communications* **2017,** *53* (56), 7937-7940.
13. Takahashi, K.; Geib, S. J.; Maeda, K.; Curran, D. P.; Taniguchi, T., Radical trans-Hydroboration of Substituted 1, 3-Diynes with an N-Heterocyclic Carbene Borane. *Organic letters* **2021,** *23* (3), 1071-1075.
14. Smela, M. P.; Hoye, T. R., A Traceless Tether Strategy for Achieving Formal Intermolecular Hexadehydro-Diels–Alder Reactions. *Organic letters* **2018,** *20* (17), 5502-5505.
15. Pelter, A.; Hughes, R.; Smith, K.; Tabata, M., A new synthesis of unsymmetrical conjugated diynes. *Tetrahedron Letters* **1976,** *17* (48), 4385-4388.
16. Li, X.; Liu, X.; Chen, H.; Wu, W.; Qi, C.; Jiang, H., Copper-Catalyzed Aerobic Oxidative Transformation of Ketone-Derived N-Tosyl Hydrazones: An Entry to Alkynes. *Angewandte Chemie* **2014,** *126* (52), 14713-14717.
